# A Database on Mycorrhizal Traits of Chinese Medicinal Plants

**DOI:** 10.3389/fpls.2022.840343

**Published:** 2022-03-01

**Authors:** Menghan Zhang, Zhaoyong Shi, Shan Zhang, Jiakai Gao

**Affiliations:** ^1^College of Agriculture, Henan University of Science and Technology, Luoyang, China; ^2^Henan Engineering Research Center for Rural Human Settlement, Luoyang, China; ^3^Luoyang Key Laboratory of Symbiotic Microorganism and Green Development, Luoyang, China

**Keywords:** arbuscular mycorrhizal (AM), ectomycorrhiza (ECM), ericoid mycorrhiza (ERM), orchid mycorrhiza (ORM), mycorrhizal type, mycorrhizal status, Chinese medicinal plants

## Abstract

The mycorrhizal traits of plants have been widely reported based on different scales or plant functional groups. To better utilize mycorrhizae to improve the cultivation yield and active ingredient accumulation of medicinal plants, a database of medicinal plant mycorrhizal characteristics is needed. A database on mycorrhizal traits including mycorrhizal type or status of Chinese medicinal plant species was assembled. In this study, the mycorrhizal type or status of a total of 3,230 medicinal plants was presented. Among them, the mycorrhizal traits of 1,321 species were ascertained. These medicinal plants had three mycorrhizal statuses, both single mycorrhiza (SM) and multi-mycorrhiza (MM) contained four mycorrhizal types. The majority of medicinal plants were obligatorily symbiotic with mycorrhizal fungi with 926 (70.10%) species. The most widespread mycorrhizal type is AM, which is associated with 842 medicinal plant species (90.93% of mycorrhiza has an obligatorily symbiotic relationship with Chinese medicinal plants). Another broadly studied mycorrhizal type is ECM, which is associated with 15 medicinal plant species. This study is the first exclusive database on mycorrhizal traits of medicinal plants, which provides both mycorrhizal type and status. This database provides valuable resources for identifying the mycorrhizal information of medicinal plants and enriching the theory of mycorrhizal traits, which will greatly benefit the production or management of medicinal plants.

## Introduction

One of the key innovations in land plants is forming associations with mycorrhizal fungi (Soudzilovskaia et al., 2020). Mycorrhizae are associations between mycorrhizal fungi and plant roots, which are widely found in terrestrial ecosystems (Davison et al., 2015; Sizonenko et al., 2020). A well-established function of mycorrhizae is to transmit nutrients and water to the plant and provide protection against biotic or abiotic stresses (Smith and Read, 2008). Depending on the presence or absence of mycorrhizae, mycorrhizal statuses are classified into obligatorily mycorrhizal (OM), facultatively mycorrhizal (FM), and non-mycorrhizal (NM). Species that can form mycorrhizae in all habitats are called obligatorily mycorrhizal, whereas species that can form mycorrhizae in one habitat but not in another are called facultatively mycorrhizal, and species that cannot form mycorrhizae in all habitats are called non-mycorrhizal (Wang and Qiu, 2006). Mycorrhizal types are usually classified as arbuscular mycorrhiza (AM), ectomycorrhiza (ECM), ericoid mycorrhiza (ERM), and orchid mycorrhiza (ORM) based on the types of colonization of mycorrhizal fungi in plants (Brundrett and Tedersoo, 2018). In addition, some plants have multiple mycorrhizal types (Smith and Read, 2008; Soudzilovskaia et al., 2020).

Plant mycorrhizal traits have attracted attention including different scales, plant functional groups, or communities (Davison et al., 2015; Rillig et al., 2019; Shi et al., 2021). Different mycorrhizal traits of plant nutrient acquisition strategies (Kuga et al., 2014; van der Heijden et al., 2015; Brundrett, 2017; Tedersoo and Bahram, 2019) will affect plant growth response (Bingham and Simard, 2011; Zeng et al., 2013; Zhang et al., 2020) and stress resistance (Huang et al., 2004; Chen et al., 2007; Maffei et al., 2014), and ultimately affect plant community composition (van der Heijden et al., 1998; Klironomos et al., 2011; Hempel et al., 2013), ecological diversity (van der Heijden et al., 1998), and productivity (Yang et al., 2014; Amanifar and Toghranegar, 2020; Wu et al., 2021). In addition, the economic spectral traits of leaves (Shi et al., 2020) and the nutrient distribution of organs (Yang et al., 2021) were different with different mycorrhizal traits. Mycorrhizal traits were classified into mycorrhizal status or type. Plants in different mycorrhizal statuses had different abilities to adapt to the environment. OM has been reported to make plants more competitive in new environments, while FM species were more adaptable to the environment (Hempel et al., 2013). Different mycorrhizal types affected soil carbon and nitrogen cycling differently (Veresoglou et al., 2012a,b; Soudzilovskaia et al., 2015; Averill and Hawkes, 2016). It has been hypothesized that mycorrhizae can serve as a predictive framework for carbon and nitrogen cycling within ecosystems (Wurzburger and Brookshire, 2017). Mycorrhizal type or status information is crucial for understanding the relationship of plant–fungi association (van der Heijden and Horton, 2009; Kraft et al., 2015; Valverde-Barrantes et al., 2017), and it provides a reference for the application of mycorrhizal research.

Using mycorrhizae to improve the active ingredients and cultivation yield of medicinal plants is one of the hot issues in current research (Zeng et al., 2013; Li et al., 2021; Sun et al., 2021). China has abundant resources of medicinal plants, with approximately 3,000 species (Huang et al., 2005; Zhao, 2017; Que et al., 2018). It is also one of the earliest countries to use medicinal plants for curing diseases of humans and animals in the world (Petrovska, 2012). It is recorded that the Chinese had taken medicinal plants for treating many diseases *circa* 2500 BC (Wiart and Read, 2006). At the same time, the excellent curative effect of medicinal plants had been recognized worldwide (Kalauokalani et al., 2001; Borba Nascimento et al., 2018; Wasana et al., 2021). It is well known that artemisinin is extracted from the medicinal plant (Price, 2000). The discoverer Professor Tu Youyou was awarded Nobel Prize in 2015. According to the WHO, 22.395 million people were cured by artemisinin in 2018 (World Health Organization [WHO], 2019). It is noteworthy that medicinal plant extract compounds, such as quercetin, luteolin, kaempferol, and acacetin, have also played an outstanding role in curing COVID-19 by enhancing the immunity of patients significantly, reducing the likelihood of patients becoming critical stage, and shortening the average of hospitalization day for patients by 2 days (Luo et al., 2020; Khadka et al., 2021). The issue of how to improve the active ingredients and cultivation yield of medicinal plants has received extensive attention from related scholars (Borba Nascimento et al., 2018; Wang et al., 2020; Khadka et al., 2021; Wasana et al., 2021). Mycorrhizae have acquired a lot of attention from medicinal botanists for their ability to improve plant nutrient or water transport and induce physiological or secondary metabolic activities (Zeng et al., 2013; Li et al., 2021). Studies have shown that different mycorrhizal traits have different effects on different medicinal plants (Zeng et al., 2013; Zhang et al., 2020). AM can promote the accumulation of medicinally active components of the *Eclipta prostrata* (Duc et al., 2020). ECM can improve the resistance of *Pinus tabulaeformis* to drought (Huang et al., 2004). The understanding of mycorrhizal traits is very important to improve the yield of medicinal plants and the study of medicinal ingredients.

At present, many databases describing mycorrhizal traits have been established. Soudzilovskaia et al. (2020) established the global database of plant mycorrhizal types. Mycorrhizal type or status databases have also been established in several regions, such as the United Kingdom (Harley and Harley, 1987; Fitter, 2005), the former Soviet Union (Akhmetzhanova et al., 2012), Central Europe (Hempel et al., 2013), and California, United States (Pringle et al., 2009). Previous studies are a great resource for understanding the relationship of plant symbiosis with mycorrhizae. However, a comprehensive and exclusive database on mycorrhizal traits of Chinese medicinal plants is lacking, which may restrict the study of mycorrhizal symbiosis.

Our objective is to explore the mycorrhizal type or status of all medicinal plants in China and to provide fundamental information for the study of mycorrhizal traits associated with medicinal plants. To thoroughly evaluate the mycorrhizal type or status of all medicinal plants in China, we searched a large number of recent publications. Among them, several important publications support the extraction of Chinese medicinal plants’ mycorrhizal information (Harley and Harley, 1987; Wang and Qiu, 2006; Akhmetzhanova et al., 2012; Hempel et al., 2013; Soudzilovskaia et al., 2020). This is the most comprehensive database on medicinal plant mycorrhizae to date, including all medicinal plants in China. This database provides valuable resources for identifying the mycorrhizal information of medicinal plants, and enriching the theory of mycorrhizal traits will greatly benefit the production or management of medicinal plants.

## Materials and Methods

### The Acquisition of Medical Plant Species in China

A total of 3,230 species of Chinese medicinal plants were obtained, which was from the Chinese Academy of Sciences digitized China’s most authoritative vascular plant summary of “The Flora of China^1^.” Besides, Chinese medicinal plants were classified at the level of phyla and families according to “The flora of China.”

### Assembly of the Database on Mycorrhizal Traits of Chinese Medicinal Plants

We searched peer-reviewed articles related to mycorrhizal information of Chinese medicinal plants using the China National Knowledge Infrastructure (CNKI) and Web of Science (Supplementary Data Sheet 4). We used the method employed by Wang and Qiu (2006), Averill et al. (2019), Shi et al. (2020), and Yang et al. (2021) to ascertain the mycorrhizal traits of Chinese medicinal plants.

The mycorrhizae of Chinese medicinal plants were ascertained according to the published records based on the method employed by Harley and Harley (1987); Wang and Qiu (2006), Akhmetzhanova et al. (2012), and Hempel et al. (2013). We classified the mycorrhizal types of Chinese medicinal plants as follows: the mycorrhizal type of species obligatorily associated with typical arbuscular structure was classified as AM type, and the mycorrhizal type of species facultatively associated with AM was classified as AM + NM type, according to the method employed by Akhmetzhanova et al. (2012) in a study on a mycorrhizal intensity database of 3,000 vascular plant species from the former Soviet Union and Wang and Qiu (2006) in a study on the phylogenetic distribution and evolution of mycorrhizae in land plants. Others were classified in the same way. The mycorrhizae of species were classified as AM + ECM type if they form AM or ECM. Others were classified in the same way (Supplementary Data Sheet 2).

The mycorrhizal types of species were classified into single mycorrhiza (SM) type and multi-mycorrhiza (MM) type in Chinese medicinal plants according to the study by Smith and Read (2008); Koele et al. (2012), Brundrett and Tedersoo (2018), and Soudzilovskaia et al. (2020). If the Chinese medicinal plant species are found to form one mycorrhiza type, we classified it as SM, and if the species is found to form two or more types of mycorrhizae, we classified it as MM. For example, we classified the mycorrhizae of Chinese medicinal plants with AM and AM + NM as SM type, and AM + ECM was classified as MM type. Others were classified in the same way.

According to the method of Wang and Qiu (2006) for phylogenetic distribution and evolution in land plants mycorrhizae, we classified the mycorrhizal status of the Chinese medicinal plant obligatorily associated with AM, ECM, and AM + ECM as OM and the species facultatively associated with AM, ECM, and AM + ECM (i.e., AM + NM, ECM + NM, and AM + ECM + NM) as FM, and the species cannot form mycorrhizal NM (Supplementary Data Sheet 3).

### Statistical Data

We classified and summarized the information of Chinese medicinal plants and their corresponding mycorrhizal traits using Office 2010. In total, 3,230 species of Chinese medicinal plants including three plant phyla and 209 families was researched. We analyzed the distribution of mycorrhizal type or status at different taxonomic levels of Chinese medicinal plants and provided a reference for mycorrhizal traits of Chinese medicinal plants at the species level.

## Results

### Mycorrhizal Statuses or Types of Chinese Medicinal Plants

**FIGURE 1:**
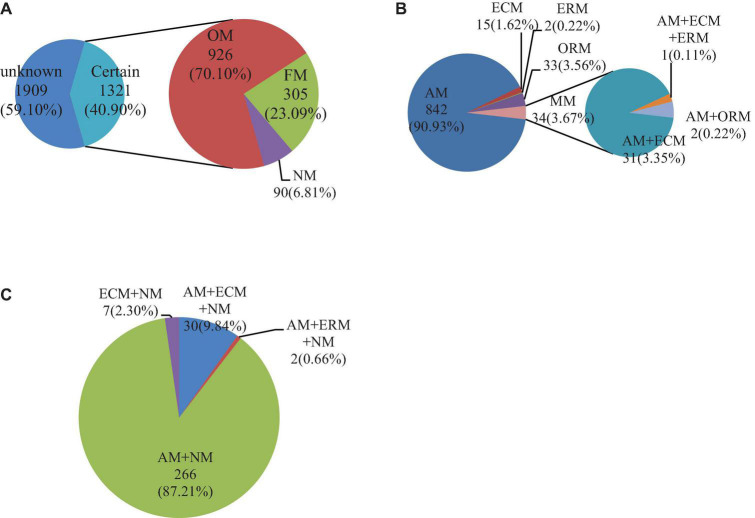
Quantitative results of mycorrhizal status or type distribution of Chinese medicinal plants. Mycorrhizal statuses or types of Chinese medicinal plants **(A)**, mycorrhizal types of Chinese medicinal plants in OM **(B)**, mycorrhizal types of Chinese medicinal plants in FM **(C)**. The certain in the figure is the Chinese medicinal plants with mycorrhizal traits recorded in our search, while unknown stands for none. Obligatorily mycorrhizal (OM), facultatively mycorrhizal (FM), and non-mycorrhizal (NM) are the three different mycorrhizal statuses. Arbuscular mycorrhizal (AM), ectomycorrhizal (ECM), ericoid mycorrhizal (ERM), and orchid mycorrhizal (ORM) are the four different mycorrhizal types, and MM is multi-mycorrhizal. The number represents the amount of medicinal plant species, and the percentage represents the proportion of this part.

The mycorrhizal information of a total of 3,230 medicinal plants was searched in China (Figure 1A). Among them, the mycorrhizal information of 40.90% (1,321 species) of Chinese medicinal plants was recorded (Supplementary Data Sheet 1). There were three mycorrhizal statuses (Supplementary Data Sheet 3) and 11 mycorrhizal types (Supplementary Data Sheet 1) in the 1,321 species of Chinese medicinal plants. Among the mycorrhizal statuses, OM had the highest proportion in the three mycorrhizal statuses, with 926 species accounting for 70.10%. NM had the least proportion in the three mycorrhizal statuses, with only 90 species accounting for 6.81%. Next, FM accounted for 23.09% (305 species). Among the 11 mycorrhizal types, AM was the highest proportion of mycorrhizal types, with 857 species. Additionally, AM + NM was the second-largest mycorrhizal type in plants, with 264 species. The number of other types (nine types of mycorrhizal Chinese medicinal plants) was less than 100 species. There were 15 species of ECM and 7 species of ECM + NM. AM + ECM + ERM had the least number of plants, with only one Chinese medicinal plant species. In total, most Chinese medicinal plants had mycorrhizae, and AM species was the main mycorrhizal type of Chinese medicinal plants.

Figures 1B,C show the mycorrhizal types of 926 OM and 305 FM species, respectively. The OM had more mycorrhizal types than FM. Among the OM, SM was the main mycorrhizal type, accounting for 96.36%. AM was the most common mycorrhizal type, making up 90.93% in OM. The proportion of ECM, ERM, and ORM species was small, accounting for less than 5% of all OM. Among them, ERM had the least, only 0.21%. The proportion of MM in OM was 3.64%. Among them, AM + ECM accounted for the largest proportion, 91.18% in MM. AM + ECM + ERM and AM + ORM species accounted for the same proportion, both accounting for less than 10%. In the FM of medicinal plants in China, AM + NM was the main mycorrhizal type, accounting for 87.21%. Although the proportion of the other three mycorrhizal types accounted for less than 10%, there were still some differences. In the three mycorrhizal types, AM + ECM + NM took the most, accounting for nearly 10%, and AM + ERM + NM was the smallest, accounting for only 0.66%. Among the FM, only ECM + NM did not contain AM, but ECM + NM only had 2.30%. On the whole, AM or associated probably with AM species were always the vast majority in Chinese medicinal plants.

### Mycorrhizal Statuses or Types of Chinese Medicinal Plant Phyla

**FIGURE 2:**
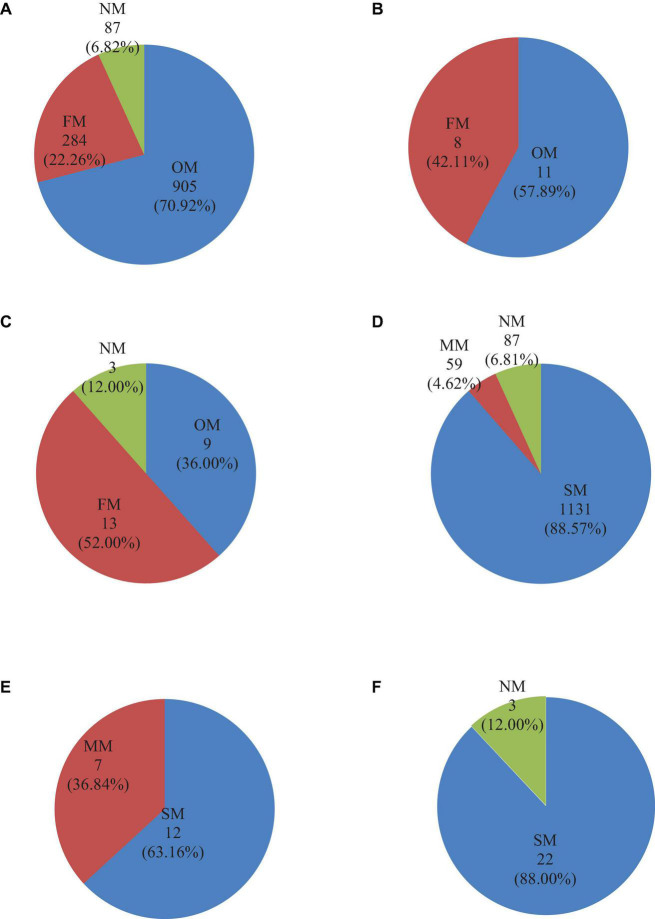
Quantitative results of mycorrhizal status or type distribution of Chinese medicinal plants at phylum levels. Quantitative results of mycorrhizal status or type distribution of Chinese medicinal plants: **(A)** mycorrhizal status of angiosperms, **(B)** mycorrhizal status of gymnosperms, **(C)** mycorrhizal status of pteridophytes, **(D)** mycorrhizal type of angiosperms, **(E)** mycorrhizal type of gymnosperms, and **(F)** mycorrhizal type of pteridophytes. Single mycorrhizal (SM) included AM (AM + NM), ECM (ECM + NM), ERM, ORM. MM included AM + ECM (AM + ECM + NM), AM + ORM, AM + ERM + NM and AM + ECM + ERM. The number represents the amount of medicinal plant species, and the percentage represents the proportion of this part.

To investigate how obvious mycorrhizal statuses were distributed, we divided medicinal plants into three phyla: angiosperm, gymnosperm, and pteridophytes (Figure 2). The results showed that gymnosperm species do not have NM but only have mycorrhizal statuses of OM and FM. The ratio of OM:FM was close to 6:4. In angiosperm species, OM species occupied the dominant position of Chinese medicinal plants, with OM:FM:NM ratio close to 7:2:1. Additionally, in the phylum of pteridophyte species, the FM occupied the dominant position of Chinese medicinal plants, with OM:FM:NM ratio close to 4:5:1. In the study of mycorrhizal types of Chinese medicinal plants in three phyla, we found that SM had the largest proportion. The largest proportion of SM was found in angiosperms, accounting for 88.57%. Even in gymnosperms with the fewest SM, SM still accounted for more than 50%. Among the three plant phyla, only the pteridophyte species contained SM, and the proportion of SM species accounted for 88%. Furthermore, gymnosperm species were more likely to form MM than SM in Chinese medicinal plant species. The result showed that although the mycorrhizal statuses or types of Chinese medicinal plants vary among phyla, all the medicinal plants in different phyla obligatorily tended to form an SM.

### Mycorrhizal Statuses of Chinese Medicinal Plant Families

Mycorrhizal statuses of families in Chinese medicinal plants were presented in Table 1. The mycorrhizal information of 3,230 species of Chinese medicinal plants from 209 families was searched, and the information of 1,321 species from 183 families was recorded. Among them, Ranunculaceae species had the largest number of Chinese medicinal plants, with 219 species. However, only 51 species of Ranunculaceae plants had mycorrhizal information. In the 51 species of Ranunculaceae, OM accounted for 76% (41 species), while NM accounted for less than 10%, with only five species. Leguminosae species was the most recorded mycorrhizal information in Chinese medicinal plants, with 109 species. Of them, OM accounted for more than 80% (94 species) in our recorded Leguminosae species, and the NM accounted for less than 5%. It is worth noting that mycorrhizae must be formed in 69 families of Chinese medicinal plants, and no mycorrhizae were found in 6 families of Chinese medicinal plants. In general, Chinese medicinal plants tend to associate with mycorrhizae.

**TABLE 1:** Mycorrhizal statuses at family level of Chinese medicinal plants.

Family	The ratio of research species and total	The percentage of species in OM/FM/NM
Acanthaceae	6/18	83/17/0
Actinidiaceae	1/2	0/100/0
Adiantaceae	3/3	0/100/0
Aizoaceae	3/3	33/33/33
Alangiaceae	3/5	67/33/0
Alismataceae	3/4	33/33/33
Amaranthaceae	14/22	29/71/0
Amaryllidaceae	6/6	67/33/0
Anacardiaceae	7/10	71/29/0
Annonaceae	4/11	50/25/25
Apocynaceae	18/53	78/22/0
Aquifoliaceae	4/9	75/25/0
Araceae	18/71	56/22/22
Araliaceae	8/34	88/13/0
Araucariaceae	1/1	100/0/0
Aristolochiaceae	5/46	100/0/0
Asclepiadaceae	14/83	79/21/0
Aspleniaceae	1/1	0/100/0
Balsaminaceae	2/13	50/50/0
Basellaceae	1/3	0/100/0
Begoniaceae	1/2	100/0/0
Berberidaceae	8/40	75/25/0
Bignoniaceae	8/20	88/13/0
Bixaceae	1/1	100/0/0
Bombacaceae	1/1	100/0/0
Boraginaceae	5/16	80/20/0
Botrychiaceae	2/3	100/0/0
Burseraceae	2/4	100/0/0
Cactaceae	2/2	100/0/0
Calycanthaceae	2/2	100/0/0
Campanulaceae	1/11	100/0/0
Cannaceae	1/1	100/0/0
Capparaceae	2/9	0/100/0
Caprifoliaceae	3/23	100/0/0
Caricaceae	1/1	100/0/0
Caryophyllaceae	16/41	19/50/31
Casuarinaceae	1/1	100/0/0
Celastraceae	3/7	67/33/0
Cephalotaxaceae	3/6	100/0/0
Ceratophyllaceae	1/1	0/100/0
Chenopodiaceae	7/8	29/29/43
Chloranthaceae	5/11	80/20/0
Combretaceae	3/5	100/0/0
Commelinaceae	6/11	0/67/33
Compositae	108/214	81/17/3
Connaraceae	1/1	100/0/0
Convolvulaceae	16/34	56/31/13
Coriariaceae	1/1	100/0/0
Cornaceae	2/7	50/50/0
Crassulaceae	5/19	100/0/0
Cruciferae	29/43	14/59/28
Cucurbitaceae	13/27	85/15/0
Cupressaceae	4/4	25/75/0
Cycadaceae	1/1	100/0/0
Cyperaceae	1/3	0/100/0
Davalliaceae	1/2	100/0/0
Dioscoreaceae	9/22	89/11/0
Dipsacaceae	2/6	100/0/0
Dipterocarpaceae	2/2	100/0/0
Droseraceae	3/4	0/33/67
Drynariaceae	1/3	0/100/0
Dryopteridaceae	1/2	100/0/0
Ebenaceae	5/8	80/20/0
Elaeagnaceae	6/15	83/17/0
Elaeocarpaceae	1/1	100/0/0
Empetraceae	1/1	0/100/0
Ephedraceae	4/6	100/0/0
Ericaceae	7/20	86/14/0
Eucommiaceae	1/1	100/0/0
Euphorbiaceae	34/60	71/24/6
Fagaceae	3/3	0/100/0
Flagellariaceae	1/1	100/0/0
Gentianaceae	1/12	0/100/0
Geraniaceae	4/8	100/0/0
Gesneriaceae	6/42	100/0/0
Ginkgoaceae	1/1	0/100/0
Gramineae	23/33	57/39/4
Guttiferae	9/13	67/22/11
Haloragidaceae	1/2	0/100/0
Hamamelidaceae	2/6	0/100/0
Hippocastanaceae	1/2	100/0/0
Hippocrateaceae	1/1	100/0/0
Icacinaceae	1/4	100/0/0
Iridaceae	4/14	100/0/0
Juglandaceae	2/3	0/100/0
Juncaceae	1/2	0/100/0
Labiatae	58/176	81/12/7
Lardizabalaceae	3/10	100/0/0
Lauraceae	28/44	89/7/4
Lecythidaceae	1/1	100/0/0
Leguminosae	109/197	86/11/3
Lemnaceae	2/2	0/50/50
Liliaceae	32/64	81/13/6
Linaceae	3/4	67/33/0
Loganiaceae	6/19	83/17/0
Loranthaceae	1/14	100/0/0
Lygodiaceae	1/1	0/100/0
Lythraceae	7/7	14/86/0
Magnoliaceae	15/39	93/7/0
Malvaceae	14/19	64/36/0
Marantaceae	2/2	100/0/0
Marsileaceae	1/1	0/100/0
Melastomataceae	2/27	50/50/0
Meliaceae	4/6	50/25/25
Menispermaceae	5/19	100/0/0
Moraceae	12/13	83/17/0
Musaceae	1/3	100/0/0
Myricaceae	1/1	100/0/0
Myristicaceae	1/1	100/0/0
Myrsinaceae	10/35	90/10/0
Myrtaceae	2/4	50/50/0
Nephrolepidaceae	1/1	100/0/0
Nyctaginaceae	2/4	0/100/0
Nymphaeaceae	2/2	0/50/50
Nyssaceae	1/1	100/0/0
Oleaceae	13/24	54/38/8
Onagraceae	7/9	43/57/0
Ophioglossaceae	2/2	100/0/0
Orchidaceae	7/23	100/0/0
Oxalidaceae	6/7	33/67/0
Palmae	5/7	80/20/0
Pandanaceae	2/3	0/50/50
Papaveraceae	11/57	64/27/9
Parkeriaceae	1/2	0/100/0
Passifloraceae	3/7	100/0/0
Pedaliaceae	1/1	100/0/0
Philydraceae	1/1	0/100/0
Phrymaceae	1/1	100/0/0
Phytolaccaceae	2/2	50/50/0
Pinaceae	4/5	0/100/0
Piperaceae	7/13	71/14/14
Plumbaginaceae	3/10	100/0/0
Polygalaceae	5/23	100/0/0
Polygonaceae	19/30	53/42/5
Polypodiaceae	2/6	0/100/0
Pontederiaceae	1/1	0/100/0
Portulacaceae	5/5	20/40/40
Potamogetonaceae	1/1	0/0/100
Primulaceae	8/25	100/0/0
Pteridaceae	3/3	33/67/0
Pteridiaceae	2/2	0/100/0
Punicaceae	1/1	100/0/0
Pyrolaceae	1/2	100/0/0
Ranunculaceae	51/219	76/14/10
Rhamnaceae	13/22	46/46/8
Rhizophoraceae	2/2	0/100/0
Rosaceae	65/124	75/22/3
Rubiaceae	16/59	56/31/13
Rutaceae	15/31	80/20/0
Sabiaceae	1/4	100/0/0
Salicaceae	5/6	40/60/0
Salviniaceae	1/1	0/100/0
Santalaceae	2/6	50/50/0
Sapindaceae	7/12	43/43/14
Sapotaceae	4/5	100/0/0
Saururaceae	1/3	100/0/0
Saxifragaceae	2/32	100/0/0
Scrophulariaceae	9/23	56/33/11
Simaroubaceae	2/3	50/50/0
Sinopteridaceae	2/2	50/50/0
Solanaceae	13/19	54/38/8
Sparganiaceae	1/2	100/0/0
Staphyleaceae	1/1	100/0/0
Stemonaceae	2/5	100/0/0
Sterculiaceae	3/7	100/0/0
Symplocaceae	7/10	100/0/0
Taccaceae	1/2	0/100/0
Tamaricaceae	1/1	100/0/0
Taxaceae	1/1	100/0/0
Theaceae	1/2	100/0/0
Thymelaeaceae	3/12	100/0/0
Tiliaceae	2/3	100/0/0
Typhaceae	3/5	0/100/0
Ulmaceae	6/7	33/33/33
Umbelliferae	25/86	68/20/12
Urticaceae	8/44	75/13/13
Valerianaceae	5/9	80/20/0
Verbenaceae	18/60	72/28/0
Violaceae	10/19	80/20/0
Vitaceae	6/12	100/0/0
Zingiberaceae	24/43	92/4/4
Zygophyllaceae	4/4	50/25/25

*Mycorrhizal statuses: OM, obligatorily mycorrhizal; FM, facultatively mycorrhizal; NM, non-mycorrhizal.*

## Discussion

The database was first established in this study to provide information on the mycorrhizal type or status of Chinese medicinal plants. This study is the first systematic summary and exploration of the mycorrhizal type or status of all Chinese medicinal plants. These data were urgently needed because of their ultimate importance in exploring the symbiosis relationship between mycorrhizae and Chinese medicinal plants (Long et al., 2010; Penuelas et al., 2013; Song et al., 2019). At the same time, these data are urgently needed because they are extremely important for utilizing mycorrhizae to enhance the cultivation yield and active ingredients of medicinal plants. We have enriched the mycorrhizal database through the search of the existing published literature information. Previous studies on mycorrhizal database provide great support for our study (Harley and Harley, 1987; Wang and Qiu, 2006; Akhmetzhanova et al., 2012; Hempel et al., 2013; Soudzilovskaia et al., 2020). The data list of this database contains 209 families with 3,000 species of Chinese medicinal plants. Even though FungalRoot (Soudzilovskaia et al., 2020) is the largest database in the world, it still does not contain more than two-thirds of species information in our database. The database provides a reference for the identification of medicinal plant mycorrhizal traits, enriches mycorrhizal traits theory, and provides a reference for understanding the symbiotic relationship between plant and mycorrhiza.

The database can provide information on the mycorrhizal type or status of each medicinal plant that has been studied. At present, there are many databases about the status of plant mycorrhizal plants (Harley and Harley, 1987; Wang and Qiu, 2006; Akhmetzhanova et al., 2012; Hempel et al., 2013; Soudzilovskaia et al., 2020). Compared with this data information, our database confirms the earlier claims that more than 70% of plants have mycorrhizae (Wang and Qiu, 2006), and less than 10% do not form mycorrhizae (Brundrett and Tedersoo, 2018). Our database also confirms previous studies that the majority of Chinese medicinal plants with mycorrhizae are AM (842 species in our database, which accounts for 71.10% of OM), while ECM species (15 species in our database) account for only a small proportion of all Chinese medicinal plant species (Soudzilovskaia et al., 2020). In addition, ERM species accounted for a very small part of the Chinese medicinal plants in our database, and most of them were of Ericaceae plant species. For the special distribution of ERM, we believe that this may be due to the specific symbiosis produced by the mutual selection of ERM and Rhododendron plants in the process of mycorrhizal associations (Zhalnina et al., 2018; Genre et al., 2020). Considering that our data only represent the information on mycorrhizal traits of Chinese medicinal plants (1,321 species) and cannot accurately represent the information on mycorrhizal traits of all Chinese medicinal plants (3,230 species), our findings need to be treated with caution. Therefore, further study is needed to obtain the true distribution of mycorrhizal traits of medicinal plants.

Some interesting phenomena were found in studying the distribution of mycorrhizal statuses in different plant phyla. Only OM and FM existed in the Chinese medicinal plants with known mycorrhizal information in gymnosperms. Wang and Qiu (2006) also showed that gymnosperms only contained OM and FM. Among the medicinal plants of pteridophytes, there are few species of medicinal plants in OM status. This may be related to the growth of most ferns in a humid environment, which is not conducive to the formation of mycorrhizal associations (Jayachandran and Shetty, 2003; Ipsilantis and Sylvia, 2007). An interesting question is which aspects of the medicinal plants’ ecological taxonomy and mycorrhizal traits cause the different distribution of mycorrhizal status in different phyla. This information will enhance our understanding of plant mycorrhizal symbiotic systems, given that most ecological models of plants and mycorrhizae are based on mycorrhizal traits.

The database explored the distribution of single and multiple mycorrhizae in Chinese medicinal plants by obtaining detailed mycorrhizal type information about each medicinal plant. We detected that the vast majority of plants had SM, and only infrequent plants had MM (Figure 2). MM colonization of plants has been well documented (Smith and Read, 2008; Brundrett and Tedersoo, 2018; Soudzilovskaia et al., 2020). Double mycorrhizal types of plants were first reported in the global mycorrhizal database (Soudzilovskaia et al., 2020). Previous studies have found that species that may be symbiotic with AM can also be symbiotic with ECM and ERM fungi, with a total of 64 species in our database (Smith and Read, 2008; Brundrett and Tedersoo, 2018). Furthermore, the database statistics showed that although SM was the main mycorrhizal type of medicinal plants, medicinal plants in both OM and FM statuses had MM type, with OM having more types and numbers of MM.

By providing detailed information on the mycorrhizal traits of Chinese medicinal plants in different families, our database explores the relationship between the mycorrhizal symbiosis status of medicinal plants and the biological taxonomy of medicinal plants. Our mycorrhizal information differs from previous records in the Ranunculaceae with the most species list and the legume with the most mycorrhizal records (Harley and Harley, 1987; Wang and Qiu, 2006). In legumes, we have more abundant records, but a lower proportion of OM, and in Ranunculaceae, we have more abundant records, and at the same time, the proportion of OM is higher. This may be due to insufficient studies of plant-mycorrhizal relationships. To obtain more accurate mycorrhizal traits information, further exploration is required.

At present, there have been many studies on the use of mycorrhizae to improve the cultivation yield and active ingredients of medicinal plants (Zeng et al., 2013; Li et al., 2021; Sun et al., 2021). This part of the research shows that different types of mycorrhizae can, respectively, affect seed germination and seedling growth (Sharma et al., 2007; Zhang et al., 2020) and increase the accumulation of active ingredients of medicinal plants (Zeng et al., 2013). However, there is a lack of research on which medicinal plants can mycorrhizae associate with and which types of mycorrhizae can associate with which medicinal plants. To overcome these problems, we constructed this database by searching for published articles. However, we must admit that our database has certain limitations. When the ecosystem has succession or exhibits extreme nutrient levels or climatic conditions, the resulting mycorrhizal traits may be different from the information provided by the database. In this case and for medicinal plant species not included in the database, we recommend *in situ* determination of mycorrhizal traits.

In summary, the unique content of this database will supplement medicinal plants and mycorrhizae studies. These data will be used to help understand and analyze the mutualistic symbiosis relationship between medicinal plants and mycorrhizae, which was very important for the development of medicinal botany and mycorrhizae. Our database provided information of mycorrhizal type or status in Chinese medicinal plants that have been published and provided Chinese medicinal plants and the Latin names, which will be convenient for others to search. This provides great convenience for the study of using mycorrhizae to improve the cultivation yield and active ingredients of medicinal plants and will provide great benefits for the production and management of medicinal plants.

## Conclusion

Medicinal plants have rich mycorrhizal traits. The mycorrhizal traits of all Chinese medicinal plants were studied, and a database of mycorrhizal statuses or types of medicinal plants was established. The mycorrhizal traits of 1,321 medicinal plants from 183 families of three phyla were identified. These medicinal plants have three mycorrhizal statuses and four single and four multiple mycorrhizal types. This database is the first exclusive database to describe the mycorrhizal traits of medicinal plants, which provides valuable resources for the identification of medicinal plant mycorrhizal information and enriches the theory of mycorrhizal traits.

## Data Availability Statement

The datasets presented in this study can be found in online repositories. The names of the repository/repositories and accession number(s) can be found in the article/Supplementary Material.

## Author Contributions

MZ and ZS: conceptualization, software, data curation, and visualization. ZS: methodology, resources, supervision, project administration, and funding acquisition. MZ, JG, and ZS: validation and writing – original draft preparation. MZ, SZ, and ZS: formal analysis, writing, review, and editing. MZ: investigation. All authors contributed to the article and approved the submitted version.

## Conflict of Interest

The authors declare that the research was conducted in the absence of any commercial or financial relationships that could be construed as a potential conflict of interest.

## Publisher’s Note

All claims expressed in this article are solely those of the authors and do not necessarily represent those of their affiliated organizations, or those of the publisher, the editors and the reviewers. Any product that may be evaluated in this article, or claim that may be made by its manufacturer, is not guaranteed or endorsed by the publisher.

## Footnotes

1
http://www.iplant.cn/frps/jingji/2
