# Supplementary material for: A Database on Mycorrhizal Traits of Chinese Medicinal Plants
Source: Front Plant Sci. 2022 Mar 1;13:840343. doi: 10.3389/fpls.2022.840343 (PMC8921535; doi:10.3389/fpls.2022.840343)
Supplement: Supplementary file 1 [file Data_Sheet_1.PDF]

Y: yes; N: no

| Species | Family | Phylum | Mycorrhizae or not(Y/N) |
|---|---|---|---|
| *Adhatoda vasica* | Acanthaceae | Angiospermae | Y |
| *Andrographis paniculata* | Acanthaceae | Angiospermae | Y |
| *Asystasia chelonoides* | Acanthaceae | Angiospermae | N |
| *Asystasiella neesiana* | Acanthaceae | Angiospermae | N |
| *Baphicacanthus cusia* | Acanthaceae | Angiospermae | N |
| *Barleria cristata* | Acanthaceae | Angiospermae | Y |
| *Barleria lupulina* | Acanthaceae | Angiospermae | Y |
| *Championella sarcorrhiza* | Acanthaceae | Angiospermae | N |
| *Dicliptera chinensis* | Acanthaceae | Angiospermae | N |
| *Gendarussa ventricosa* | Acanthaceae | Angiospermae | N |
| *Gendarussa vulgaris* | Acanthaceae | Angiospermae | N |
| *Hygrophila salicifolia* | Acanthaceae | Angiospermae | Y |
| *Hypoestes purpurea* | Acanthaceae | Angiospermae | N |
| *Lepidagathis incurva* | Acanthaceae | Angiospermae | N |
| *Peristrophe japonica* | Acanthaceae | Angiospermae | N |
| *Rhinacanthus nasutus* | Acanthaceae | Angiospermae | Y |
| *Rostellularia procumbens* | Acanthaceae | Angiospermae | N |
| *Rungia pectinata* | Acanthaceae | Angiospermae | N |
| *Actinidia polygama* | Actinidiaceae | Angiospermae | Y |
| *Saurauia tristyla var. tristyla* | Actinidiaceae | Angiospermae | N |
| *Mollugo cerviana* | Aizoaceae | Angiospermae | Y |
| *Mollugo stricta* | Aizoaceae | Angiospermae | Y |
| *Tetragonia tetragonioides* | Aizoaceae | Angiospermae | Y |
| *Alangium chinense* | Alangiaceae | Angiospermae | Y |
| *Alangium faberi* | Alangiaceae | Angiospermae | N |
| *Alangium faberi var. platyphyllum* | Alangiaceae | Angiospermae | N |
| *Alangium platanifolium* | Alangiaceae | Angiospermae | Y |
| *Alangium salviifolium* | Alangiaceae | Angiospermae | Y |
| *Alisma canaliculatum* | Alismataceae | Angiospermae | Y |
| *Alisma orinentale* | Alismataceae | Angiospermae | N |
| *Alisma plantago-aquatica* | Alismataceae | Angiospermae | Y |
| *Sagittaria pygmaea* | Alismataceae | Angiospermae | Y |
| *Achyranthes aspera* | Amaranthacea | Angiospermae | Y |
| *Achyranthes bidentata* | Amaranthacea | Angiospermae | Y |
| *Achyranthes longifolia* | Amaranthacea | Angiospermae | N |
| *Achyranthes longifolia f. rubra* | Amaranthacea | Angiospermae | N |
| *Achyranthes ogatai* | Amaranthacea | Angiospermae | N |
| *Aerva sanguinolenta* | Amaranthacea | Angiospermae | Y |
| *Alternanthera philoxeroides* | Amaranthacea | Angiospermae | Y |
| *Alternanthera sessilis* | Amaranthacea | Angiospermae | Y |
| *Amaranthus caudatus* | Amaranthacea | Angiospermae | Y |
| *Amaranthus lividus* | Amaranthacea | Angiospermae | Y |
| *Amaranthus retroflexus* | Amaranthacea | Angiospermae | Y |
| *Amaranthus spinosus* | Amaranthacea | Angiospermae | Y |
| *Amaranthus tricolor* | Amaranthacea | Angiospermae | Y |
| *Amaranthus viridis* | Amaranthacea | Angiospermae | Y |
| *Celosia argentea* | Amaranthacea | Angiospermae | Y |
| *Celosia cristata* | Amaranthacea | Angiospermae | Y |

| | | | |
|---|---|---|---|
| *Celosia swinhoei* | Amaranthacea | Angiospermae | N |
| *Cladostachys frutescens* | Amaranthacea | Angiospermae | N |
| *Cyathula capitata* | Amaranthacea | Angiospermae | N |
| *Cyathula officinalis* | Amaranthacea | Angiospermae | N |
| *Cyathula prostrata* | Amaranthacea | Angiospermae | Y |
| *Iresine herbstii* | Amaranthacea | Angiospermae | N |
| *Agave americana* | Amaryllidacea | Angiospermae | Y |
| *Agave sisalana* | Amaryllidacea | Angiospermae | Y |
| *Crinum asiaticum* | Amaryllidacea | Angiospermae | Y |
| *Crinum asiaticum var. sinicum* | Amaryllidacea | Angiospermae | Y |
| *Curculigo orchioides* | Amaryllidacea | Angiospermae | Y |
| *Narcissus tazetta var. chinensis* | Amaryllidacea | Angiospermae | Y |
| *Anacardium occidentale* | Anacardiaceae | Angiospermae | Y |
| *Choerospondias axillaris var. axillar* | Anacardiaceae | Angiospermae | Y |
| *Dobinea delavayi* | Anacardiaceae | Angiospermae | N |
| *Dracontomelon duperreanum* | Anacardiaceae | Angiospermae | N |
| *Mangifera indica* | Anacardiaceae | Angiospermae | Y |
| *Pistacia vera* | Anacardiaceae | Angiospermae | Y |
| *Pistacia weinmannifolia* | Anacardiaceae | Angiospermae | Y |
| *Rhus chinensis* | Anacardiaceae | Angiospermae | Y |
| *Toxicodendron succedaneum* | Anacardiaceae | Angiospermae | Y |
| *Toxicodendron vernicifluum* | Anacardiaceae | Angiospermae | N |
| *Annona squamosa* | Annonaceae | Angiospermae | Y |
| *Artabotrys hexapetalus* | Annonaceae | Angiospermae | Y |
| *Desmos chinensis* | Annonaceae | Angiospermae | Y |
| *Fissistigma bracteolatum* | Annonaceae | Angiospermae | N |
| *Fissistigma glaucescens* | Annonaceae | Angiospermae | Y |
| *Fissistigma oldhamii* | Annonaceae | Angiospermae | N |
| *Fissistigma polyanthum* | Annonaceae | Angiospermae | N |
| *Fissistigma uonicum* | Annonaceae | Angiospermae | N |
| *Goniothalamus donnaiensis* | Annonaceae | Angiospermae | N |
| *Uvaria microcarpa* | Annonaceae | Angiospermae | N |
| *Uvaria tonkinensis* | Annonaceae | Angiospermae | N |
| *Angelica decusiva* | Apiaceae | Angiospermae | N |
| *Aganosma kwangsiensis* | Apocynaceae | Angiospermae | N |
| *Aganosma schlechteriana* | Apocynaceae | Angiospermae | N |
| *Alstonia mairei* | Apocynaceae | Angiospermae | N |
| *Alstonia scholaris* | Apocynaceae | Angiospermae | Y |
| *Alstonia yunnanensis* | Apocynaceae | Angiospermae | N |
| *Alyxia levinei* | Apocynaceae | Angiospermae | N |
| *Alyxia sinensis* | Apocynaceae | Angiospermae | N |
| *Amsonia sinensis* | Apocynaceae | Angiospermae | N |
| *Apocynum venetum* | Apocynaceae | Angiospermae | Y |
| *Apocynum venetum var. ellipticifoliu* | Apocynaceae | Angiospermae | Y |
| *Beaumontia grandiflora* | Apocynaceae | Angiospermae | N |
| *Catharanthus roseus* | Apocynaceae | Angiospermae | Y |
| *Cerbera manghas* | Apocynaceae | Angiospermae | Y |
| *Chonemorpha eriostylis* | Apocynaceae | Angiospermae | N |
| *Chonemorpha megacalyx* | Apocynaceae | Angiospermae | N |
| *Chonemorpha valvata* | Apocynaceae | Angiospermae | N |

| | | | |
|---|---|---|---|
| *Ecdysanthera rosea* | Apocynaceae | Angiospermae | Y |
| *Ecdysanthera utilis* | Apocynaceae | Angiospermae | N |
| *Ervatamia hainanensis* | Apocynaceae | Angiospermae | N |
| *Ervatamia kwangsiensis* | Apocynaceae | Angiospermae | N |
| *Ervatamia officinalis* | Apocynaceae | Angiospermae | N |
| *Holarrhena antidysenterica* | Apocynaceae | Angiospermae | N |
| *Ichnocarpus frutescens* | Apocynaceae | Angiospermae | Y |
| *Ichnocarpus oliganthus* | Apocynaceae | Angiospermae | N |
| *Kopsia officinalis* | Apocynaceae | Angiospermae | N |
| *Melodinus fusiformis* | Apocynaceae | Angiospermae | Y |
| *Melodinus henryi* | Apocynaceae | Angiospermae | N |
| *Melodinus suaveolens* | Apocynaceae | Angiospermae | N |
| *Nerium oleander* | Apocynaceae | Angiospermae | Y |
| *Parabarium huaitingii* | Apocynaceae | Angiospermae | N |
| *Parabarium micranthum* | Apocynaceae | Angiospermae | N |
| *Pottsia laxiflora* | Apocynaceae | Angiospermae | N |
| *Rauvolfia latifrons* | Apocynaceae | Angiospermae | N |
| *Rauvolfia serpentina* | Apocynaceae | Angiospermae | Y |
| *Rauvolfia tetraphylla* | Apocynaceae | Angiospermae | Y |
| *Rauvolfia verticillata* | Apocynaceae | Angiospermae | Y |
| *Rauvolfia verticillata var. hainanens* | Apocynaceae | Angiospermae | Y |
| *Rauvolfia vomitoria* | Apocynaceae | Angiospermae | Y |
| *Rauvolfia yunnanensis* | Apocynaceae | Angiospermae | Y |
| *Sindechites henryi* | Apocynaceae | Angiospermae | N |
| *Strophanthus divaricatus* | Apocynaceae | Angiospermae | N |
| *Strophanthus gratus* | Apocynaceae | Angiospermae | N |
| *Strophanthus hispidus* | Apocynaceae | Angiospermae | N |
| *Strophanthus sarmentosus* | Apocynaceae | Angiospermae | N |
| *Trachelospermum dunnii* | Apocynaceae | Angiospermae | N |
| *Trachelospermum jasminoides* | Apocynaceae | Angiospermae | Y |
| *Trachelospermum jasminoides var. h* | Apocynaceae | Angiospermae | Y |
| *Vallaris indecora* | Apocynaceae | Angiospermae | N |
| *Wrightia laevis* | Apocynaceae | Angiospermae | N |
| *Wrightia pubescens* | Apocynaceae | Angiospermae | Y |
| *Tylophora koi Merr.* | Apocynaceae | Angiospermae | N |
| *Tylophora ovata (Lindl.) Hook. ex St* | Apocynaceae | Angiospermae | N |
| *Wrightia annamensis* | Apocynaceae | Angiospermae | N |
| *Ilex aculeolata* | Aquifoliaceae | Angiospermae | N |
| *Ilex asprella* | Aquifoliaceae | Angiospermae | Y |
| *Ilex cornuta* | Aquifoliaceae | Angiospermae | Y |
| *Ilex latifolia* | Aquifoliaceae | Angiospermae | Y |
| *Ilex macrocarpa* | Aquifoliaceae | Angiospermae | N |
| *Ilex macrocarpa var. longipeduncula* | Aquifoliaceae | Angiospermae | N |
| *Ilex microcca f. pilosa* | Aquifoliaceae | Angiospermae | Y |
| *Ilex pernyi* | Aquifoliaceae | Angiospermae | N |
| *Ilex rotunda* | Aquifoliaceae | Angiospermae | N |
| *Acorus calamus var. verus* | Araceae | Angiospermae | Y |
| *Acorus gramineus* | Araceae | Angiospermae | Y |
| *Acorus rumphianus* | Araceae | Angiospermae | N |
| *Acorus tatarinowii* | Araceae | Angiospermae | Y |

| | | | |
|---|---|---|---|
| *Aglaonema modestum* | Araceae | Angiospermae | Y |
| *Aglaonema tenuipes* | Araceae | Angiospermae | Y |
| *Alocasia cucullata* | Araceae | Angiospermae | N |
| *Alocasia macrorrhiza* | Araceae | Angiospermae | Y |
| *Amorphophallus mairei* | Araceae | Angiospermae | N |
| *Amorphophallus rivieri* | Araceae | Angiospermae | Y |
| *Amorphophallus sinensis* | Araceae | Angiospermae | N |
| *Amydrium sinense* | Araceae | Angiospermae | N |
| *Arisaema amurense var. serratum* | Araceae | Angiospermae | N |
| *Arisaema angustatum* | Araceae | Angiospermae | N |
| *Arisaema angustatum var. peninsula* | Araceae | Angiospermae | N |
| *Arisaema asperatum* | Araceae | Angiospermae | N |
| *Arisaema austro-yunnanense* | Araceae | Angiospermae | N |
| *Arisaema bathycoleum* | Araceae | Angiospermae | N |
| *Arisaema calcareum* | Araceae | Angiospermae | N |
| *Arisaema decipiens* | Araceae | Angiospermae | N |
| *Arisaema du-bois-reymondiae* | Araceae | Angiospermae | N |
| *Arisaema elephas* | Araceae | Angiospermae | N |
| *Arisaema erubescens* | Araceae | Angiospermae | Y |
| *Arisaema fargesii* | Araceae | Angiospermae | N |
| *Arisaema flavum* | Araceae | Angiospermae | Y |
| *Arisaema franchetianum* | Araceae | Angiospermae | N |
| *Arisaema heterophyllum* | Araceae | Angiospermae | N |
| *Arisaema inkiangense var. maculatu* | Araceae | Angiospermae | N |
| *Arisaema lichiangense* | Araceae | Angiospermae | N |
| *Arisaema lingyunense* | Araceae | Angiospermae | N |
| *Arisaema lobatum* | Araceae | Angiospermae | N |
| *Arisaema nepenthoides* | Araceae | Angiospermae | N |
| *Arisaema prazeri* | Araceae | Angiospermae | N |
| *Arisaema rhizomatum var. nudum* | Araceae | Angiospermae | N |
| *Arisaema rhombiforme* | Araceae | Angiospermae | N |
| *Arisaema sikokianum var. henryanur* | Araceae | Angiospermae | N |
| *Arisaema yunnanense* | Araceae | Angiospermae | N |
| *Caladium bicolor* | Araceae | Angiospermae | Y |
| *Colocasia antiquorum* | Araceae | Angiospermae | Y |
| *Colocasia esculenta* | Araceae | Angiospermae | Y |
| *Colocasia gigantea* | Araceae | Angiospermae | N |
| *Cryptocoryne yunnanensis* | Araceae | Angiospermae | N |
| *Epipremnum pinnatum* | Araceae | Angiospermae | N |
| *Gonatanthus pumilus* | Araceae | Angiospermae | N |
| *Homalomena occulta* | Araceae | Angiospermae | N |
| *Lasia spinosa* | Araceae | Angiospermae | Y |
| *Pinellia cordata* | Araceae | Angiospermae | N |
| *Pinellia integrifolia* | Araceae | Angiospermae | N |
| *Pinellia pedatisecta* | Araceae | Angiospermae | N |
| *Pinellia ternata* | Araceae | Angiospermae | Y |
| *Pistia stratiotes* | Araceae | Angiospermae | Y |
| *Pothos chinensis var. lotienensis* | Araceae | Angiospermae | N |
| *Pothos kerrii* | Araceae | Angiospermae | N |
| *Pothos pilulifer* | Araceae | Angiospermae | N |

| | | | |
|---|---|---|---|
| *Pothos repens* | Araceae | Angiospermae | N |
| *Pothos scandens* | Araceae | Angiospermae | N |
| *Remusatia vivipara* | Araceae | Angiospermae | N |
| *Rhaphidophora decursiva* | Araceae | Angiospermae | Y |
| *Rhaphidophora hongkongensis* | Araceae | Angiospermae | Y |
| *Rhaphidophora hookeri* | Araceae | Angiospermae | N |
| *Rhaphidophora peepla* | Araceae | Angiospermae | N |
| *Schismatoglottis calyptrata* | Araceae | Angiospermae | Y |
| *Typhonium albidinervum* | Araceae | Angiospermae | N |
| *Typhonium divaricatum* | Araceae | Angiospermae | N |
| *Typhonium flagelliforme* | Araceae | Angiospermae | N |
| *Typhonium giganteum* | Araceae | Angiospermae | N |
| *Typhonium roxburgii* | Araceae | Angiospermae | N |
| *Typhonium trifoliatum* | Araceae | Angiospermae | N |
| *Typhonium trilobatum* | Araceae | Angiospermae | N |
| *Steudnera colocasiifolia* | Araceae | Angiospermae | N |
| *Acanthopanax evodiaefolius var. ferr* | Araliaceae | Angiospermae | N |
| *Acanthopanax gracilistylus* | Araliaceae | Angiospermae | Y |
| *Acanthopanax lasiogyne* | Araliaceae | Angiospermae | N |
| *Acanthopanax senticosus* | Araliaceae | Angiospermae | Y |
| *Acanthopanax sessiliflorus* | Araliaceae | Angiospermae | Y |
| *Acanthopanax trifoliatus* | Araliaceae | Angiospermae | N |
| *Acanthopanax verticillatus* | Araliaceae | Angiospermae | N |
| *Aralia armata* | Araliaceae | Angiospermae | N |
| *Aralia chinensis* | Araliaceae | Angiospermae | N |
| *Aralia cordata* | Araliaceae | Angiospermae | Y |
| *Aralia decaisneana* | Araliaceae | Angiospermae | N |
| *Aralia fargesii* | Araliaceae | Angiospermae | N |
| *Dendropanax dentiger* | Araliaceae | Angiospermae | N |
| *Dendropanax proteus* | Araliaceae | Angiospermae | N |
| *Hedera nepalensis var. sinensis* | Araliaceae | Angiospermae | Y |
| *Heteropanax brevipedicellatus* | Araliaceae | Angiospermae | N |
| *Heteropanax chinensis* | Araliaceae | Angiospermae | N |
| *Heteropanax fragrans* | Araliaceae | Angiospermae | N |
| *Kalopanax septemlobus* | Araliaceae | Angiospermae | Y |
| *Macropanax rosthornii* | Araliaceae | Angiospermae | N |
| *Nothopanax davidii* | Araliaceae | Angiospermae | N |
| *Nothopanax delavayi* | Araliaceae | Angiospermae | N |
| *Panax ginseng* | Araliaceae | Angiospermae | Y |
| *Schefflera arboricola* | Araliaceae | Angiospermae | N |
| *Schefflera delavayi* | Araliaceae | Angiospermae | N |
| *Schefflera kwangsiensis* | Araliaceae | Angiospermae | N |
| *Schefflera octophylla* | Araliaceae | Angiospermae | N |
| *Schefflera venulosa* | Araliaceae | Angiospermae | N |
| *Tetrapanax papyrifer* | Araliaceae | Angiospermae | Y |
| *Tupidanthus calyptratus* | Araliaceae | Angiospermae | N |
| *Panax japonicus var. bipinnatifidus* | Araliaceae | Angiospermae | N |
| *Panax pseudo-ginseng var. elegantio* | Araliaceae | Angiospermae | N |
| *Panax pseudo-ginseng var. japonicu:* | Araliaceae | Angiospermae | N |
| *Panax pseudo-ginseng var. notogins* | Araliaceae | Angiospermae | N |

| | | | |
|---|---|---|---|
| *Aristolochia cathcartii* | Aristolochiace | Angiospermae | N |
| *Aristolochia championii* | Aristolochiace | Angiospermae | N |
| *Aristolochia contorta* | Aristolochiace | Angiospermae | N |
| *Aristolochia debilis* | Aristolochiace | Angiospermae | Y |
| *Aristolochia elegans* | Aristolochiace | Angiospermae | N |
| *Aristolochia fangchi* | Aristolochiace | Angiospermae | N |
| *Aristolochia fordiana* | Aristolochiace | Angiospermae | N |
| *Aristolochia foveolata* | Aristolochiace | Angiospermae | N |
| *Aristolochia hainanensis* | Aristolochiace | Angiospermae | N |
| *Aristolochia howii* | Aristolochiace | Angiospermae | N |
| *Aristolochia impresinervis* | Aristolochiace | Angiospermae | N |
| *Aristolochia kwangsiensis* | Aristolochiace | Angiospermae | N |
| *Aristolochia manshuriensis* | Aristolochiace | Angiospermae | N |
| *Aristolochia mollissima* | Aristolochiace | Angiospermae | Y |
| *Aristolochia obliqua* | Aristolochiace | Angiospermae | N |
| *Aristolochia tagala* | Aristolochiace | Angiospermae | N |
| *Aristolochia thwaitesii* | Aristolochiace | Angiospermae | N |
| *Aristolochia tuberosa* | Aristolochiace | Angiospermae | N |
| *Aristolochia tubiflora* | Aristolochiace | Angiospermae | N |
| *Aristolochia versicolor* | Aristolochiace | Angiospermae | N |
| *Asarum caudigerellum* | Aristolochiace | Angiospermae | N |
| *Asarum caudigerum* | Aristolochiace | Angiospermae | N |
| *Asarum caulescens* | Aristolochiace | Angiospermae | Y |
| *Asarum debile* | Aristolochiace | Angiospermae | N |
| *Asarum delavayi* | Aristolochiace | Angiospermae | N |
| *Asarum forbesii* | Aristolochiace | Angiospermae | N |
| *Asarum fukienense* | Aristolochiace | Angiospermae | N |
| *Asarum geophilum* | Aristolochiace | Angiospermae | N |
| *Asarum himalaicum* | Aristolochiace | Angiospermae | N |
| *Asarum ichangense* | Aristolochiace | Angiospermae | N |
| *Asarum inflatum* | Aristolochiace | Angiospermae | N |
| *Asarum longerhizomatosum* | Aristolochiace | Angiospermae | N |
| *Asarum magnificum* | Aristolochiace | Angiospermae | N |
| *Asarum magnificum var. dinghuense* | Aristolochiace | Angiospermae | N |
| *Asarum maximum* | Aristolochiace | Angiospermae | N |
| *Asarum petelotii* | Aristolochiace | Angiospermae | N |
| *Asarum porphyronotum var. atrovire* | Aristolochiace | Angiospermae | N |
| *Asarum pulchellum* | Aristolochiace | Angiospermae | N |
| *Asarum renicordatum* | Aristolochiace | Angiospermae | N |
| *Asarum sagittarioides* | Aristolochiace | Angiospermae | N |
| *Asarum sieboldii* | Aristolochiace | Angiospermae | Y |
| *Asarum sieboldii f. seoulense* | Aristolochiace | Angiospermae | Y |
| *Asarum splendens* | Aristolochiace | Angiospermae | N |
| *Asarum wulingense* | Aristolochiace | Angiospermae | N |
| *Saruma henryi* | Aristolochiace | Angiospermae | N |
| *Aristolochia arborea* | Aristolochiace | Angiospermae | N |
| *Asclepias curassavica* | Asclepiadacea | Angiospermae | Y |
| *Brachystelma edule* | Asclepiadacea | Angiospermae | N |
| *Brachystelma kerrii* | Asclepiadacea | Angiospermae | N |
| *Calotropis gigantea* | Asclepiadacea | Angiospermae | Y |

| | | | |
|---|---|---|---|
| *Calotropis procera* | Asclepiadacea | Angiospermae | Y |
| *Ceropegia mairei* | Asclepiadacea | Angiospermae | N |
| *Ceropegia trichantha* | Asclepiadacea | Angiospermae | N |
| *Cryptolepis buchananii* | Asclepiadacea | Angiospermae | Y |
| *Cryptolepis sinensis* | Asclepiadacea | Angiospermae | N |
| *Cynanchum amplexicaule var. castar* | Asclepiadacea | Angiospermae | N |
| *Cynanchum atratum* | Asclepiadacea | Angiospermae | N |
| *Cynanchum auriculatum* | Asclepiadacea | Angiospermae | Y |
| *Cynanchum bungei* | Asclepiadacea | Angiospermae | N |
| *Cynanchum chekiangense* | Asclepiadacea | Angiospermae | N |
| *Cynanchum corymbosum* | Asclepiadacea | Angiospermae | N |
| *Cynanchum dubium* | Asclepiadacea | Angiospermae | N |
| *Cynanchum forrestii* | Asclepiadacea | Angiospermae | N |
| *Cynanchum glaucescens* | Asclepiadacea | Angiospermae | N |
| *Cynanchum inamoenum* | Asclepiadacea | Angiospermae | N |
| *Cynanchum mooreanum* | Asclepiadacea | Angiospermae | N |
| *Cynanchum officinale* | Asclepiadacea | Angiospermae | N |
| *Cynanchum otophyllum* | Asclepiadacea | Angiospermae | N |
| *Cynanchum paniculatum* | Asclepiadacea | Angiospermae | N |
| *Cynanchum sibiricum* | Asclepiadacea | Angiospermae | Y |
| *Cynanchum stauntonii* | Asclepiadacea | Angiospermae | N |
| *Cynanchum versicolor* | Asclepiadacea | Angiospermae | N |
| *Cynanchum wilfordii* | Asclepiadacea | Angiospermae | N |
| *Dischidanthus urceolatus* | Asclepiadacea | Angiospermae | N |
| *Dischidia australis* | Asclepiadacea | Angiospermae | N |
| *Dischidia chinensis* | Asclepiadacea | Angiospermae | N |
| *Dolichopetalum kwangsiense* | Asclepiadacea | Angiospermae | N |
| *Dregea sinensis* | Asclepiadacea | Angiospermae | N |
| *Dregea volubilis* | Asclepiadacea | Angiospermae | N |
| *Gomphocarpus fruticosus* | Asclepiadacea | Angiospermae | Y |
| *Gongronema nepalense* | Asclepiadacea | Angiospermae | N |
| *Graphistemma pictum* | Asclepiadacea | Angiospermae | N |
| *Gymnema longiretinaculatum* | Asclepiadacea | Angiospermae | N |
| *Gymnema sylvestre* | Asclepiadacea | Angiospermae | Y |
| *Gymnema tingens* | Asclepiadacea | Angiospermae | N |
| *Heterostemma alatum* | Asclepiadacea | Angiospermae | N |
| *Heterostemma oblongifolium* | Asclepiadacea | Angiospermae | N |
| *Holostemma annulare* | Asclepiadacea | Angiospermae | N |
| *Hoya carnosa* | Asclepiadacea | Angiospermae | Y |
| *Hoya carnosa var. marmorata* | Asclepiadacea | Angiospermae | Y |
| *Hoya formosana* | Asclepiadacea | Angiospermae | N |
| *Hoya fungii* | Asclepiadacea | Angiospermae | N |
| *Hoya lancilimba* | Asclepiadacea | Angiospermae | N |
| *Hoya lancilimba f. tsoi* | Asclepiadacea | Angiospermae | N |
| *Hoya pottsii* | Asclepiadacea | Angiospermae | N |
| *Hoya pottsii var. angustifolia* | Asclepiadacea | Angiospermae | N |
| *Marsdenia griffithii* | Asclepiadacea | Angiospermae | N |
| *Marsdenia longipes* | Asclepiadacea | Angiospermae | N |
| *Marsdenia officinalis* | Asclepiadacea | Angiospermae | N |
| *Marsdenia sinensis* | Asclepiadacea | Angiospermae | N |

| | | | |
|---|---|---|---|
| *Marsdenia tenacissima* | Asclepiadacea | Angiospermae | N |
| *Marsdenia tomentosa* | Asclepiadacea | Angiospermae | Y |
| *Metaplexis hemsleyana* | Asclepiadacea | Angiospermae | N |
| *Metaplexis japonica* | Asclepiadacea | Angiospermae | Y |
| *Myriopteron extensum* | Asclepiadacea | Angiospermae | N |
| *Oxystelma esculentum* | Asclepiadacea | Angiospermae | N |
| *Pentasacme championii* | Asclepiadacea | Angiospermae | N |
| *Periploca calophylla* | Asclepiadacea | Angiospermae | N |
| *Periploca forrestii* | Asclepiadacea | Angiospermae | N |
| *Periploca sepium* | Asclepiadacea | Angiospermae | Y |
| *Sarcostemma acidum* | Asclepiadacea | Angiospermae | N |
| *Secamone lanceolata* | Asclepiadacea | Angiospermae | N |
| *Secamone sinica* | Asclepiadacea | Angiospermae | N |
| *Stelmatocrypton khasianum* | Asclepiadacea | Angiospermae | N |
| *Stephanotis chunii* | Asclepiadacea | Angiospermae | N |
| *Streptocaulon griffithii* | Asclepiadacea | Angiospermae | N |
| *Telosma cordata* | Asclepiadacea | Angiospermae | N |
| *Toxocarpus wightianus* | Asclepiadacea | Angiospermae | N |
| *Tylophora arenicola* | Asclepiadacea | Angiospermae | Y |
| *Tylophora atrofolliculata* | Asclepiadacea | Angiospermae | N |
| *Tylophora floribunda* | Asclepiadacea | Angiospermae | N |
| *Tylophora kerrii* | Asclepiadacea | Angiospermae | N |
| *Tylophora koi* | Asclepiadacea | Angiospermae | N |
| *Tylophora mollissima* | Asclepiadacea | Angiospermae | N |
| *Tylophora ovata* | Asclepiadacea | Angiospermae | N |
| *Tylophora renchangii* | Asclepiadacea | Angiospermae | N |
| *Tylophora secamonoides* | Asclepiadacea | Angiospermae | N |
| *Tylophora trichophylla* | Asclepiadacea | Angiospermae | N |
| *Tylophora yunnanensis* | Asclepiadacea | Angiospermae | N |
| *Smilacina nokomoticola* | Asparagaceae | Angiospermae | N |
| *Dendranthema morii* | Asteraceae | Angiospermae | N |
| *Inula aspera* | Asteraceae | Angiospermae | N |
| *Inula simonii* | Asteraceae | Angiospermae | N |
| *Inula verrucosa* | Asteraceae | Angiospermae | N |
| *Balanophora fargesii* | Balanophorace | Angiospermae | N |
| *Balanophora formosana* | Balanophorace | Angiospermae | N |
| *Balanophora indica* | Balanophorace | Angiospermae | N |
| *Balanophora involucrata* | Balanophorace | Angiospermae | N |
| *Balanophora laxiflora* | Balanophorace | Angiospermae | N |
| *Balanophora parvior* | Balanophorace | Angiospermae | N |
| *Impatiens apalophylla* | Balsaminaceae | Angiospermae | N |
| *Impatiens arguta* | Balsaminaceae | Angiospermae | N |
| *Impatiens balsamina* | Balsaminaceae | Angiospermae | Y |
| *Impatiens chinensis* | Balsaminaceae | Angiospermae | Y |
| *Impatiens chlorosepala* | Balsaminaceae | Angiospermae | N |
| *Impatiens claviger* | Balsaminaceae | Angiospermae | N |
| *Impatiens cyanantha* | Balsaminaceae | Angiospermae | N |
| *Impatiens lasiophyton* | Balsaminaceae | Angiospermae | N |
| *Impatiens leptocaulon* | Balsaminaceae | Angiospermae | N |
| *Impatiens loulanensis* | Balsaminaceae | Angiospermae | N |

| | | | |
|---|---|---|---|
| *Impatiens pritzelii* | Balsaminaceae | Angiospermae | N |
| *Impatiens siculifer* | Balsaminaceae | Angiospermae | N |
| *Impatiens uliginosa* | Balsaminaceae | Angiospermae | N |
| *Anredera cordifolia* | Basellaceae | Angiospermae | N |
| *Anredera scandens* | Basellaceae | Angiospermae | N |
| *Basella alba* | Basellaceae | Angiospermae | Y |
| *Begonia argenteo-guttata* | Begoniaceae | Angiospermae | N |
| *Begonia maculata* | Begoniaceae | Angiospermae | Y |
| *Berberis aggregata* | Berberidaceae | Angiospermae | N |
| *Berberis amurensis* | Berberidaceae | Angiospermae | Y |
| *Berberis brachypoda* | Berberidaceae | Angiospermae | N |
| *Berberis circumserrata* | Berberidaceae | Angiospermae | N |
| *Berberis dasystachya* | Berberidaceae | Angiospermae | N |
| *Berberis dielsiana* | Berberidaceae | Angiospermae | N |
| *Berberis ferdinandi-coburgii* | Berberidaceae | Angiospermae | N |
| *Berberis henryana* | Berberidaceae | Angiospermae | N |
| *Berberis hsuyunensis* | Berberidaceae | Angiospermae | N |
| *Berberis julianae* | Berberidaceae | Angiospermae | N |
| *Berberis lempergiana* | Berberidaceae | Angiospermae | N |
| *Berberis poiretii* | Berberidaceae | Angiospermae | Y |
| *Berberis polyantha* | Berberidaceae | Angiospermae | N |
| *Berberis pruinosa var. pruinosa* | Berberidaceae | Angiospermae | N |
| *Berberis sargentiana* | Berberidaceae | Angiospermae | Y |
| *Berberis sibirica* | Berberidaceae | Angiospermae | Y |
| *Berberis thunbergii* | Berberidaceae | Angiospermae | Y |
| *Berberis vernae* | Berberidaceae | Angiospermae | N |
| *Berberis verruculosa* | Berberidaceae | Angiospermae | N |
| *Berberis virgetorum* | Berberidaceae | Angiospermae | N |
| *Berberis wilsonae* | Berberidaceae | Angiospermae | N |
| *Berberis xinganensis* | Berberidaceae | Angiospermae | N |
| *Caulophyllum robustum* | Berberidaceae | Angiospermae | N |
| *Diphylleia sinensis* | Berberidaceae | Angiospermae | N |
| *Dysosma aurantiocaulis* | Berberidaceae | Angiospermae | N |
| *Dysosma difformis* | Berberidaceae | Angiospermae | N |
| *Dysosma majorensis* | Berberidaceae | Angiospermae | N |
| *Dysosma pleiantha* | Berberidaceae | Angiospermae | N |
| *Dysosma veitchii* | Berberidaceae | Angiospermae | N |
| *Dysosma versipellis* | Berberidaceae | Angiospermae | Y |
| *Epimedium acuminatum* | Berberidaceae | Angiospermae | N |
| *Epimedium boreali-guizhouense* | Berberidaceae | Angiospermae | N |
| *Epimedium brevicornu* | Berberidaceae | Angiospermae | N |
| *Epimedium koreanum* | Berberidaceae | Angiospermae | N |
| *Epimedium pubescens* | Berberidaceae | Angiospermae | N |
| *Epimedium sagittatum* | Berberidaceae | Angiospermae | N |
| *Gymnospermium kiangnanense* | Berberidaceae | Angiospermae | N |
| *Nandina domestica* | Berberidaceae | Angiospermae | Y |
| *Plagiorhegma dubia* | Berberidaceae | Angiospermae | N |
| *Sinopodophyllum hexandrum* | Berberidaceae | Angiospermae | Y |
| *Betula chinensis* | Betulaceae | Angiospermae | N |
| *Campsis grandiflora* | Bignoniaceae | Angiospermae | Y |

| | | | |
|---|---|---|---|
| *Campsis radicans* | Bignoniaceae | Angiospermae | N |
| *Catalpa bungei* | Bignoniaceae | Angiospermae | Y |
| *Catalpa fargesii* | Bignoniaceae | Angiospermae | N |
| *Catalpa fargesii f. duclouxii* | Bignoniaceae | Angiospermae | N |
| *Catalpa ovata* | Bignoniaceae | Angiospermae | N |
| *Incarvillea arguta* | Bignoniaceae | Angiospermae | Y |
| *Incarvillea compacta* | Bignoniaceae | Angiospermae | N |
| *Incarvillea delavayi* | Bignoniaceae | Angiospermae | N |
| *Incarvillea lutea* | Bignoniaceae | Angiospermae | N |
| *Incarvillea mairei* | Bignoniaceae | Angiospermae | N |
| *Incarvillea mairei var. grandiflora* | Bignoniaceae | Angiospermae | N |
| *Incarvillea younghusbandii* | Bignoniaceae | Angiospermae | Y |
| *Kigelia africana* | Bignoniaceae | Angiospermae | Y |
| *Millingtonia hortensis* | Bignoniaceae | Angiospermae | Y |
| *Oroxylum indicum* | Bignoniaceae | Angiospermae | Y |
| *Radermachera hainanensis* | Bignoniaceae | Angiospermae | N |
| *Radermachera pentandra* | Bignoniaceae | Angiospermae | N |
| *Radermachera sinica* | Bignoniaceae | Angiospermae | Y |
| *Radermachera yunnanensis* | Bignoniaceae | Angiospermae | N |
| *Bixa orellana* | Bixaceae | Angiospermae | Y |
| *Woodwardia cochi-chinensis* | Blechnaceae | Angiospermae | N |
| *Bombax malabaricum* | Bombacaceae | Angiospermae | Y |
| *Antiotrema dunnianum* | Boraginaceae | Angiospermae | N |
| *Arnebia euchroma* | Boraginaceae | Angiospermae | N |
| *Arnebia guttata* | Boraginaceae | Angiospermae | N |
| *Cordia dichotoma* | Boraginaceae | Angiospermae | N |
| *Cynoglossum amabile var. amabile* | Boraginaceae | Angiospermae | N |
| *Cynoglossum divaricatum* | Boraginaceae | Angiospermae | N |
| *Cynoglossum officinale* | Boraginaceae | Angiospermae | Y |
| *Cynoglossum zeylanicum* | Boraginaceae | Angiospermae | Y |
| *Heliotropium indicum* | Boraginaceae | Angiospermae | Y |
| *Lappula heteracantha* | Boraginaceae | Angiospermae | N |
| *Lithospermum erythrorhizon* | Boraginaceae | Angiospermae | N |
| *Lithospermum officinale* | Boraginaceae | Angiospermae | Y |
| *Lithospermum zollingeri* | Boraginaceae | Angiospermae | N |
| *Thyrocarpus sampsonii* | Boraginaceae | Angiospermae | N |
| *Trigonotis macrophylla var. verrucos* | Boraginaceae | Angiospermae | N |
| *Trigonotis peduncularis* | Boraginaceae | Angiospermae | Y |
| *Canarium album* | Burseraceae | Angiospermae | Y |
| *Canarium pimela* | Burseraceae | Angiospermae | Y |
| *Commiphora myrrha* | Burseraceae | Angiospermae | N |
| *Boswellia carteri* | Burseraceae | Angiospermae | N |
| *Opuntia monacantha* | Cactaceae | Angiospermae | Y |
| *Opuntia stricta var. dillenii* | Cactaceae | Angiospermae | Y |
| *Chimonanthus nitens* | Calycanthacea | Angiospermae | Y |
| *Chimonanthus praecox* | Calycanthacea | Angiospermae | Y |
| *Campanula colorata* | Campanulacea | Angiospermae | N |
| *Campanumoea javanica* | Campanulacea | Angiospermae | N |
| *Campanumoea lancifolia* | Campanulacea | Angiospermae | N |
| *Lobelia chinensis* | Campanulacea | Angiospermae | N |

| | | | |
|---|---|---|---|
| *Lobelia davidii var. davidii* | Campanulacea | Angiospermae | N |
| *Lobelia melliana* | Campanulacea | Angiospermae | N |
| *Lobelia sequinii* | Campanulacea | Angiospermae | N |
| *Lobelia sessilifolia* | Campanulacea | Angiospermae | N |
| *Platycodon grandiflorus* | Campanulacea | Angiospermae | Y |
| *Pratia nummularia* | Campanulacea | Angiospermae | N |
| *Wahlenbergia marginata* | Campanulacea | Angiospermae | N |
| *Canna indica* | Cannaceae | Angiospermae | Y |
| *Capparis acutifolia* | Capparaceae | Angiospermae | N |
| *Capparis bodinieri* | Capparaceae | Angiospermae | N |
| *Capparis cantoniensis* | Capparaceae | Angiospermae | N |
| *Capparis chingiana* | Capparaceae | Angiospermae | N |
| *Capparis masaikai* | Capparaceae | Angiospermae | N |
| *Capparis versicolor* | Capparaceae | Angiospermae | N |
| *Capparis yunnanensis* | Capparaceae | Angiospermae | N |
| *Cleome gynandra* | Capparaceae | Angiospermae | Y |
| *Cleome viscosa* | Capparaceae | Angiospermae | Y |
| *Leycesteria formosa* | Caprifoliaceae | Angiospermae | N |
| *Leycesteria formosa var. stenosepala* | Caprifoliaceae | Angiospermae | N |
| *Lonicera acuminata* | Caprifoliaceae | Angiospermae | N |
| *Lonicera buchananii* | Caprifoliaceae | Angiospermae | N |
| *Lonicera confusa* | Caprifoliaceae | Angiospermae | Y |
| *Lonicera crassifolia* | Caprifoliaceae | Angiospermae | N |
| *Lonicera dasystyla* | Caprifoliaceae | Angiospermae | N |
| *Lonicera hispida* | Caprifoliaceae | Angiospermae | Y |
| *Lonicera hypoglauca* | Caprifoliaceae | Angiospermae | N |
| *Lonicera hypoglauca subsp. nudiflor* | Caprifoliaceae | Angiospermae | N |
| *Lonicera inodora* | Caprifoliaceae | Angiospermae | N |
| *Lonicera macranthoides* | Caprifoliaceae | Angiospermae | N |
| *Lonicera pampaninii* | Caprifoliaceae | Angiospermae | N |
| *Lonicera rhytidophylla* | Caprifoliaceae | Angiospermae | N |
| *Lonicera similis* | Caprifoliaceae | Angiospermae | N |
| *Lonicera similis var. omeiensis* | Caprifoliaceae | Angiospermae | N |
| *Lonicera tragophylla* | Caprifoliaceae | Angiospermae | N |
| *Sambucus adnata* | Caprifoliaceae | Angiospermae | Y |
| *Sambucus chinensis* | Caprifoliaceae | Angiospermae | N |
| *Viburnum cylindricum* | Caprifoliaceae | Angiospermae | N |
| *Viburnum odoratissimum var. odora* | Caprifoliaceae | Angiospermae | N |
| *Viburnum odoratissinum* | Caprifoliaceae | Angiospermae | N |
| *Viburnum subalpinum* | Caprifoliaceae | Angiospermae | N |
| *Carica papaya* | Caricaceae | Angiospermae | Y |
| *Agrostemma githago* | Caryophyllace | Angiospermae | Y |
| *Arenaria brevipetala* | Caryophyllace | Angiospermae | N |
| *Arenaria juncea* | Caryophyllace | Angiospermae | N |
| *Arenaria melanandra* | Caryophyllace | Angiospermae | N |
| *Arenaria przewalskii* | Caryophyllace | Angiospermae | N |
| *Arenaria serpyllifolia* | Caryophyllace | Angiospermae | Y |
| *Brachystemma calycinum* | Caryophyllace | Angiospermae | N |
| *Cucubalus baccifer* | Caryophyllace | Angiospermae | N |
| *Dianthus chinensis* | Caryophyllace | Angiospermae | Y |

| | | | |
|---|---|---|---|
| *Dianthus chinensis var. longisquama* | Caryophyllace | Angiospermae | Y |
| *Dianthus chinensis var. versicolor* | Caryophyllace | Angiospermae | Y |
| *Dianthus superbus* | Caryophyllace | Angiospermae | Y |
| *Drymaria diandra* | Caryophyllace | Angiospermae | N |
| *Gypsophila davurica* | Caryophyllace | Angiospermae | N |
| *Gypsophila oldhamiana* | Caryophyllace | Angiospermae | N |
| *Gypsophila pacifica* | Caryophyllace | Angiospermae | N |
| *Gypsophila paniculata* | Caryophyllace | Angiospermae | Y |
| *Lepyrodiclis holosteoides* | Caryophyllace | Angiospermae | N |
| *Lychnis coronata* | Caryophyllace | Angiospermae | N |
| *Lychnis senno* | Caryophyllace | Angiospermae | N |
| *Myosoton aquaticum* | Caryophyllace | Angiospermae | Y |
| *Psammosilene tunicoides* | Caryophyllace | Angiospermae | N |
| *Pseudostellaria heterophylla* | Caryophyllace | Angiospermae | Y |
| *Sagina japonica* | Caryophyllace | Angiospermae | Y |
| *Saponaria officinalis* | Caryophyllace | Angiospermae | Y |
| *Silene aprica* | Caryophyllace | Angiospermae | N |
| *Silene conoidea* | Caryophyllace | Angiospermae | Y |
| *Silene fortunei* | Caryophyllace | Angiospermae | Y |
| *Silene gracilicaulis* | Caryophyllace | Angiospermae | N |
| *Silene gracilicaulis var. rubescens* | Caryophyllace | Angiospermae | N |
| *Silene jenisseensis* | Caryophyllace | Angiospermae | N |
| *Silene viscidula* | Caryophyllace | Angiospermae | Y |
| *Stellaria chinensis* | Caryophyllace | Angiospermae | N |
| *Stellaria dichotoma var. lanceolata* | Caryophyllace | Angiospermae | N |
| *Stellaria henryi* | Caryophyllace | Angiospermae | N |
| *Stellaria media* | Caryophyllace | Angiospermae | Y |
| *Stellaria neglecta* | Caryophyllace | Angiospermae | N |
| *Stellaria uliginosa* | Caryophyllace | Angiospermae | Y |
| *Stellaria vestita* | Caryophyllace | Angiospermae | N |
| *Stellaria yunnanensis* | Caryophyllace | Angiospermae | N |
| *Vaccaria segetalis* | Caryophyllace | Angiospermae | N |
| *Casuarina equisetifolia* | Casuarinaceae | Angiospermae | Y |
| *Celastrus orbiculatus* | Celastraceae | Angiospermae | Y |
| *Celastrus paniculatus* | Celastraceae | Angiospermae | N |
| *Celastrus rosthornianus* | Celastraceae | Angiospermae | N |
| *Euonymus alatus* | Celastraceae | Angiospermae | Y |
| *Euonymus laxiflorus* | Celastraceae | Angiospermae | Y |
| *Maytenus confertiflorus* | Celastraceae | Angiospermae | N |
| *Maytenus guangxiensis* | Celastraceae | Angiospermae | N |
| *Ceratophyllum demersum* | Ceratophyllace | Angiospermae | Y |
| *Anabasis aphylla* | Chenopodiace | Angiospermae | Y |
| *Atriplex centralasiatica* | Chenopodiace | Angiospermae | Y |
| *Chenopodium album* | Chenopodiace | Angiospermae | Y |
| *Chenopodium ambrosioides* | Chenopodiace | Angiospermae | Y |
| *Chenopodium aristatum* | Chenopodiace | Angiospermae | N |
| *Chenopodium hybridum* | Chenopodiace | Angiospermae | Y |
| *Kochia scoparia* | Chenopodiace | Angiospermae | Y |
| *Salsola collina* | Chenopodiace | Angiospermae | Y |
| *Chloranthus fortunei* | Chloranthacea | Angiospermae | N |

| | | | |
|---|---|---|---|
| *Chloranthus henryi* | Chloranthacea | Angiospermae | N |
| *Chloranthus henryi var. hupehensis* | Chloranthacea | Angiospermae | N |
| *Chloranthus holostegius* | Chloranthacea | Angiospermae | N |
| *Chloranthus japonicus* | Chloranthacea | Angiospermae | Y |
| *Chloranthus multistachys* | Chloranthacea | Angiospermae | Y |
| *Chloranthus serratus* | Chloranthacea | Angiospermae | Y |
| *Chloranthus sessilifolius* | Chloranthacea | Angiospermae | N |
| *Chloranthus spicatus* | Chloranthacea | Angiospermae | Y |
| *Sarcandra glabra* | Chloranthacea | Angiospermae | Y |
| *Sarcandra hainanensis* | Chloranthacea | Angiospermae | N |
| *Calycopteris floribunda* | Combretaceae | Angiospermae | N |
| *Quisqualis indica* | Combretaceae | Angiospermae | N |
| *Terminalia bellirica* | Combretaceae | Angiospermae | Y |
| *Terminalia catappa* | Combretaceae | Angiospermae | Y |
| *Terminalia chebula* | Combretaceae | Angiospermae | Y |
| *Commelina bengalensis* | Commelinacea | Angiospermae | Y |
| *Commelina communis* | Commelinacea | Angiospermae | Y |
| *Commelina diffusa* | Commelinacea | Angiospermae | Y |
| *Commelina paludosa* | Commelinacea | Angiospermae | N |
| *Cyanotis arachnoidea* | Commelinacea | Angiospermae | N |
| *Cyanotis cristata* | Commelinacea | Angiospermae | Y |
| *Floscopa scandens* | Commelinacea | Angiospermae | N |
| *Murdannia nudiflora* | Commelinacea | Angiospermae | Y |
| *Murdannia triquetra* | Commelinacea | Angiospermae | N |
| *Pollia japonica* | Commelinacea | Angiospermae | Y |
| *Pollia miranda* | Commelinacea | Angiospermae | N |
| *Ageratum conyzoides* | Compositae | Angiospermae | Y |
| *Ageratum houstonianum* | Compositae | Angiospermae | Y |
| *Ainsliaea elegans var. strigosa* | Compositae | Angiospermae | N |
| *Ainsliaea fragrans* | Compositae | Angiospermae | N |
| *Ainsliaea pertyoides var. albo-tomen* | Compositae | Angiospermae | N |
| *Ajania variifolia* | Compositae | Angiospermae | N |
| *Anaphalis bulleyana* | Compositae | Angiospermae | N |
| *Anaphalis flavescens var. lanata* | Compositae | Angiospermae | N |
| *Anaphalis lactea* | Compositae | Angiospermae | Y |
| *Anaphalis nepalensis* | Compositae | Angiospermae | N |
| *Anaphalis porphyrolepis* | Compositae | Angiospermae | N |
| *Antennaria dioica* | Compositae | Angiospermae | Y |
| *Artemisia absinthium* | Compositae | Angiospermae | Y |
| *Artemisia anethifolia* | Compositae | Angiospermae | Y |
| *Artemisia anethoides* | Compositae | Angiospermae | N |
| *Artemisia angustissima* | Compositae | Angiospermae | N |
| *Artemisia annua* | Compositae | Angiospermae | Y |
| *Artemisia anomala* | Compositae | Angiospermae | N |
| *Artemisia anomala var. tomentella* | Compositae | Angiospermae | N |
| *Artemisia argyi* | Compositae | Angiospermae | Y |
| *Artemisia argyi var. gracilis* | Compositae | Angiospermae | Y |
| *Artemisia brachyloba* | Compositae | Angiospermae | Y |
| *Artemisia capillaris* | Compositae | Angiospermae | Y |
| *Artemisia carvifolia* | Compositae | Angiospermae | Y |

| | | | |
|---|---|---|---|
| *Artemisia conaensis* | Compositae | Angiospermae | N |
| *Artemisia dracunculus* | Compositae | Angiospermae | Y |
| *Artemisia dracunculus var. turkestan* | Compositae | Angiospermae | Y |
| *Artemisia dubia* | Compositae | Angiospermae | Y |
| *Artemisia dubia var. subdigitata* | Compositae | Angiospermae | Y |
| *Artemisia edgeworthii* | Compositae | Angiospermae | N |
| *Artemisia edgeworthii var. diffusa* | Compositae | Angiospermae | N |
| *Artemisia eriopoda* | Compositae | Angiospermae | Y |
| *Artemisia frigida* | Compositae | Angiospermae | Y |
| *Artemisia gilvescens* | Compositae | Angiospermae | N |
| *Artemisia giraldii* | Compositae | Angiospermae | Y |
| *Artemisia hedinii* | Compositae | Angiospermae | N |
| *Artemisia igniaria* | Compositae | Angiospermae | N |
| *Artemisia japonica* | Compositae | Angiospermae | Y |
| *Artemisia japonica var. hainanensis* | Compositae | Angiospermae | Y |
| *Artemisia keiskeana* | Compositae | Angiospermae | N |
| *Artemisia lactiflora* | Compositae | Angiospermae | N |
| *Artemisia lactiflora var. incisa* | Compositae | Angiospermae | N |
| *Artemisia lavandulaefolia* | Compositae | Angiospermae | Y |
| *Artemisia leucophylla* | Compositae | Angiospermae | Y |
| *Artemisia littoricola* | Compositae | Angiospermae | Y |
| *Artemisia macrocephala* | Compositae | Angiospermae | N |
| *Artemisia manshurica* | Compositae | Angiospermae | N |
| *Artemisia mongolica* | Compositae | Angiospermae | Y |
| *Artemisia moorcroftiana* | Compositae | Angiospermae | N |
| *Artemisia ordosica* | Compositae | Angiospermae | Y |
| *Artemisia orientali-hengduangensis* | Compositae | Angiospermae | N |
| *Artemisia parviflora* | Compositae | Angiospermae | N |
| *Artemisia princeps* | Compositae | Angiospermae | Y |
| *Artemisia qinlingensis* | Compositae | Angiospermae | N |
| *Artemisia roxburghiana* | Compositae | Angiospermae | Y |
| *Artemisia rubripes* | Compositae | Angiospermae | Y |
| *Artemisia rupestris* | Compositae | Angiospermae | Y |
| *Artemisia sacrorum* | Compositae | Angiospermae | Y |
| *Artemisia sacrorum var. incana* | Compositae | Angiospermae | Y |
| *Artemisia sacrorum var. messerschm* | Compositae | Angiospermae | Y |
| *Artemisia selengensis* | Compositae | Angiospermae | N |
| *Artemisia sieversiana* | Compositae | Angiospermae | Y |
| *Artemisia sphaerocephala* | Compositae | Angiospermae | Y |
| *Artemisia tournefortiana* | Compositae | Angiospermae | N |
| *Artemisia verbenacea* | Compositae | Angiospermae | N |
| *Artemisia verlotorum* | Compositae | Angiospermae | Y |
| *Artemisia vestita* | Compositae | Angiospermae | Y |
| *Artemisia vulgaris* | Compositae | Angiospermae | Y |
| *Artemisia wellbyi* | Compositae | Angiospermae | Y |
| *Aster ageratoides* | Compositae | Angiospermae | Y |
| *Aster ageratoides var. lasiocladus* | Compositae | Angiospermae | Y |
| *Aster alatipes* | Compositae | Angiospermae | N |
| *Aster auriculatus* | Compositae | Angiospermae | N |
| *Aster auriculatus f. crenatus* | Compositae | Angiospermae | N |

| | | | |
|---|---|---|---|
| *Aster batangensis var. staticefolius* | Compositae | Angiospermae | N |
| *Aster flaccidus* | Compositae | Angiospermae | N |
| *Aster likiangensis* | Compositae | Angiospermae | N |
| *Aster likiangensis f. polianthus* | Compositae | Angiospermae | N |
| *Aster oreophilus* | Compositae | Angiospermae | N |
| *Aster oreophilus f. inaequisquamus* | Compositae | Angiospermae | N |
| *Aster oreophilus f. umbrosus* | Compositae | Angiospermae | N |
| *Aster prainii* | Compositae | Angiospermae | N |
| *Aster souliei* | Compositae | Angiospermae | N |
| *Aster turbinatus var. chekiangensis* | Compositae | Angiospermae | N |
| *Atractylodes lancea* | Compositae | Angiospermae | Y |
| *Atractylodes macrocephala* | Compositae | Angiospermae | Y |
| *Bidens bipinnata* | Compositae | Angiospermae | Y |
| *Bidens biternata* | Compositae | Angiospermae | Y |
| *Bidens frondosa* | Compositae | Angiospermae | Y |
| *Bidens parviflora* | Compositae | Angiospermae | Y |
| *Bidens pilosa* | Compositae | Angiospermae | Y |
| *Bidens pilosa var. radiata* | Compositae | Angiospermae | Y |
| *Bidens tripartita* | Compositae | Angiospermae | Y |
| *Blumea balsamifera* | Compositae | Angiospermae | Y |
| *Carpesium abrotanoides* | Compositae | Angiospermae | Y |
| *Carpesium cernuum* | Compositae | Angiospermae | N |
| *Carpesium divaricatum* | Compositae | Angiospermae | Y |
| *Carpesium lipskyi* | Compositae | Angiospermae | Y |
| *Carpesium macrocephalum* | Compositae | Angiospermae | N |
| *Carpesium nepalense var. lanatum* | Compositae | Angiospermae | N |
| *Carthamus tinctorius* | Compositae | Angiospermae | Y |
| *Cavea tanguensis* | Compositae | Angiospermae | N |
| *Cavea tanguensis f. acaulis* | Compositae | Angiospermae | N |
| *Centipeda minima* | Compositae | Angiospermae | Y |
| *Conyza blinii* | Compositae | Angiospermae | Y |
| *Conyza bonariensis* | Compositae | Angiospermae | Y |
| *Conyza canadensis* | Compositae | Angiospermae | Y |
| *Conyza japonica* | Compositae | Angiospermae | Y |
| *Crassocephalum crepidioides* | Compositae | Angiospermae | Y |
| *Crepis phoenix* | Compositae | Angiospermae | N |
| *Dahlia pinnata* | Compositae | Angiospermae | Y |
| *Dendranthema indicum* | Compositae | Angiospermae | Y |
| *Dichrocephala auriculata* | Compositae | Angiospermae | N |
| *Dichrocephala benthamii* | Compositae | Angiospermae | N |
| *Doellingeria marchandii* | Compositae | Angiospermae | N |
| *Doellingeria scaber* | Compositae | Angiospermae | Y |
| *Dolomiaea souliei* | Compositae | Angiospermae | N |
| *Eclipta prostrata* | Compositae | Angiospermae | Y |
| *Elephantopus scaber* | Compositae | Angiospermae | Y |
| *Elephantopus tomentosus* | Compositae | Angiospermae | N |
| *Emilia sonchifolia* | Compositae | Angiospermae | Y |
| *Erigeron annuus* | Compositae | Angiospermae | Y |
| *Erigeron breviscapus* | Compositae | Angiospermae | Y |
| *Eupatorium chinense* | Compositae | Angiospermae | Y |

| | | | |
|---|---|---|---|
| *Eupatorium fortunei* | Compositae | Angiospermae | N |
| *Eupatorium fortunei var. angustilobu* | Compositae | Angiospermae | N |
| *Eupatorium heterophyllum* | Compositae | Angiospermae | N |
| *Eupatorium japonicum* | Compositae | Angiospermae | N |
| *Eupatorium lindleyanum* | Compositae | Angiospermae | N |
| *Eupatorium odoratum* | Compositae | Angiospermae | Y |
| *Farfugium japonicum* | Compositae | Angiospermae | Y |
| *Galinsoga parviflora* | Compositae | Angiospermae | Y |
| *Gerbera delavayi* | Compositae | Angiospermae | N |
| *Gerbera piloselloides* | Compositae | Angiospermae | N |
| *Glossogyne tenuifolia* | Compositae | Angiospermae | Y |
| *Gnaphalium affine* | Compositae | Angiospermae | Y |
| *Helianthus annuus* | Compositae | Angiospermae | Y |
| *Helianthus tuberosus* | Compositae | Angiospermae | Y |
| *Helichrysum arenarium* | Compositae | Angiospermae | Y |
| *Hippolytia delavayi* | Compositae | Angiospermae | N |
| *Hippolytia kennedyi* | Compositae | Angiospermae | N |
| *Inula britannica* | Compositae | Angiospermae | Y |
| *Inula helenium* | Compositae | Angiospermae | Y |
| *Inula helianthus-aquatica* | Compositae | Angiospermae | Y |
| *Inula hookeri* | Compositae | Angiospermae | N |
| *Inula hookeri f. major* | Compositae | Angiospermae | N |
| *Inula japonica* | Compositae | Angiospermae | Y |
| *Inula leariifolia* | Compositae | Angiospermae | N |
| *Inula nervosa* | Compositae | Angiospermae | N |
| *Inula pterocaula* | Compositae | Angiospermae | N |
| *Inula racemosa* | Compositae | Angiospermae | N |
| *Inula salicina* | Compositae | Angiospermae | Y |
| *Ixeridium sonchifolium* | Compositae | Angiospermae | N |
| *Ixeris polycephala* | Compositae | Angiospermae | Y |
| *Kalimeris indica* | Compositae | Angiospermae | Y |
| *Leontopodium andersonii* | Compositae | Angiospermae | N |
| *Leontopodium artemisiifolium* | Compositae | Angiospermae | N |
| *Leontopodium calocephalum var. uli* | Compositae | Angiospermae | N |
| *Leontopodium japonicum* | Compositae | Angiospermae | Y |
| *Leontopodium leontopodioides* | Compositae | Angiospermae | Y |
| *Ligularia hodgsonii* | Compositae | Angiospermae | N |
| *Ligularia sibirica* | Compositae | Angiospermae | Y |
| *Matricaria recutita* | Compositae | Angiospermae | Y |
| *Microglossa pyrifolia* | Compositae | Angiospermae | N |
| *Myriactis nepalensis* | Compositae | Angiospermae | N |
| *Pentanema indicum* | Compositae | Angiospermae | Y |
| *Pentanema indicum var. hypoleucum* | Compositae | Angiospermae | Y |
| *Petasites japonicus* | Compositae | Angiospermae | Y |
| *Petasites tricholobus* | Compositae | Angiospermae | N |
| *Picris japonica* | Compositae | Angiospermae | N |
| *Pulicaria chrysantha* | Compositae | Angiospermae | N |
| *Pulicaria dysenterica* | Compositae | Angiospermae | Y |
| *Pulicaria insignis* | Compositae | Angiospermae | N |
| *Pulicaria prostrata* | Compositae | Angiospermae | N |

| | | | |
|---|---|---|---|
| *Pulicaria uliginosa* | Compositae | Angiospermae | N |
| *Pyrethrum cinerariifolium* | Compositae | Angiospermae | Y |
| *Pyrethrum tatsienense* | Compositae | Angiospermae | N |
| *Rhynchospermum verticillatum* | Compositae | Angiospermae | N |
| *Saussurea costus* | Compositae | Angiospermae | Y |
| *Saussurea gossypiphora* | Compositae | Angiospermae | Y |
| *Saussurea medusa* | Compositae | Angiospermae | N |
| *Saussurea obvallata* | Compositae | Angiospermae | N |
| *Scorzonera divaricata var. sublilacir* | Compositae | Angiospermae | N |
| *Scorzonera muriculata* | Compositae | Angiospermae | Y |
| *Seriphidium brevifolium* | Compositae | Angiospermae | N |
| *Seriphidium cinum* | Compositae | Angiospermae | N |
| *Seriphidium transiliense* | Compositae | Angiospermae | Y |
| *Siegesbeckia orientalis* | Compositae | Angiospermae | Y |
| *Sinosenecio homogyniphyllus* | Compositae | Angiospermae | N |
| *Solidago decurrens* | Compositae | Angiospermae | N |
| *Solidago virgaurea* | Compositae | Angiospermae | Y |
| *Sonchus oleraceus* | Compositae | Angiospermae | Y |
| *Spilanthes callimorpha* | Compositae | Angiospermae | Y |
| *Spilanthes paniculata* | Compositae | Angiospermae | N |
| *Stemmacantha carthamoides* | Compositae | Angiospermae | N |
| *Stemmacantha uniflora* | Compositae | Angiospermae | N |
| *Syneilesis aconitifolia* | Compositae | Angiospermae | N |
| *Taraxacum mongolicum* | Compositae | Angiospermae | Y |
| *Taraxacum platypecidum* | Compositae | Angiospermae | Y |
| *Tussilago farfara* | Compositae | Angiospermae | Y |
| *Vernonia anthelmintica* | Compositae | Angiospermae | Y |
| *Vernonia chingiana* | Compositae | Angiospermae | N |
| *Vernonia cinerea* | Compositae | Angiospermae | N |
| *Vernonia cumingiana* | Compositae | Angiospermae | N |
| *Vernonia esculenta* | Compositae | Angiospermae | Y |
| *Vernonia extensa* | Compositae | Angiospermae | N |
| *Vernonia parishii* | Compositae | Angiospermae | N |
| *Vernonia patula* | Compositae | Angiospermae | N |
| *Vernonia saligna* | Compositae | Angiospermae | N |
| *Vernonia solanifolia* | Compositae | Angiospermae | N |
| *Vernonia squarrosa* | Compositae | Angiospermae | N |
| *Waldheimia glabra* | Compositae | Angiospermae | N |
| *Xanthium sibiricum* | Compositae | Angiospermae | Y |
| *Youngia stenoma* | Compositae | Angiospermae | N |
| *Rourea microphylla* | Connaraceae | Angiospermae | Y |
| *Aniseia biflora* | Convolvulacea | Angiospermae | N |
| *Argyreia acuta* | Convolvulacea | Angiospermae | N |
| *Argyreia capitata* | Convolvulacea | Angiospermae | N |
| *Argyreia obtusifolia* | Convolvulacea | Angiospermae | N |
| *Argyreia pierreana* | Convolvulacea | Angiospermae | N |
| *Argyreia seguinii* | Convolvulacea | Angiospermae | N |
| *Argyreia wallichii* | Convolvulacea | Angiospermae | N |
| *Blinkworthia convolvuloides* | Convolvulacea | Angiospermae | N |
| *Calonyction muricatum* | Convolvulacea | Angiospermae | N |

| | | | |
|---|---|---|---|
| *Calystegia hederacea* | Convolvulacea | Angiospermae | Y |
| *Calystegia sepium* | Convolvulacea | Angiospermae | Y |
| *Convolvulus arvensis* | Convolvulacea | Angiospermae | Y |
| *Cuscuta australis* | Convolvulacea | Angiospermae | Y |
| *Cuscuta campestris* | Convolvulacea | Angiospermae | Y |
| *Cuscuta chinensis* | Convolvulacea | Angiospermae | N |
| *Cuscuta japonica* | Convolvulacea | Angiospermae | N |
| *Cuscuta reflexa var. reflexa* | Convolvulacea | Angiospermae | N |
| *Dichondra repens* | Convolvulacea | Angiospermae | Y |
| *Erycibe obtusifolia* | Convolvulacea | Angiospermae | Y |
| *Erycibe schmidtii* | Convolvulacea | Angiospermae | N |
| *Evolvulus alsinoides* | Convolvulacea | Angiospermae | Y |
| *Evolvulus alsinoides var. decumbens* | Convolvulacea | Angiospermae | Y |
| *Ipomoea aquatica* | Convolvulacea | Angiospermae | Y |
| *Ipomoea cairica* | Convolvulacea | Angiospermae | Y |
| *Ipomoea digitata* | Convolvulacea | Angiospermae | N |
| *Ipomoea pes-caprae* | Convolvulacea | Angiospermae | Y |
| *Merremia hungaiensis var. linifolia* | Convolvulacea | Angiospermae | N |
| *Merremia sibirica* | Convolvulacea | Angiospermae | N |
| *Merremia umbellata subsp. orientali* | Convolvulacea | Angiospermae | Y |
| *Operculina turpethum* | Convolvulacea | Angiospermae | Y |
| *Parasitipomoea formosana* | Convolvulacea | Angiospermae | N |
| *Pharbitis nil* | Convolvulacea | Angiospermae | Y |
| *Porana mairei* | Convolvulacea | Angiospermae | N |
| *Porana racemosa* | Convolvulacea | Angiospermae | Y |
| *Coriaria nepalensis* | Coriariaceae | Angiospermae | Y |
| *Cornus officinalis* | Cornaceae | Angiospermae | Y |
| *Dendrobenthamia capitata* | Cornaceae | Angiospermae | N |
| *Dendrobenthamia melanotricha* | Cornaceae | Angiospermae | N |
| *Helwingia japonica* | Cornaceae | Angiospermae | Y |
| *Swida oblonga* | Cornaceae | Angiospermae | N |
| *Swida paucinervis* | Cornaceae | Angiospermae | N |
| *Toricellia angulata var. intermedia* | Cornaceae | Angiospermae | N |
| *Bryophyllum pinnatum* | Crassulaceae | Angiospermae | N |
| *Hylotelephium erythrostictum* | Crassulaceae | Angiospermae | Y |
| *Hylotelephium mingjinianum* | Crassulaceae | Angiospermae | N |
| *Hylotelephium verticillatum* | Crassulaceae | Angiospermae | N |
| *Kalanchoe laciniata* | Crassulaceae | Angiospermae | N |
| *Orostachys fimbriatus* | Crassulaceae | Angiospermae | Y |
| *Rhodiola algida var. tangutica* | Crassulaceae | Angiospermae | N |
| *Rhodiola dumulosa* | Crassulaceae | Angiospermae | N |
| *Rhodiola henryi* | Crassulaceae | Angiospermae | N |
| *Rhodiola kirilowii* | Crassulaceae | Angiospermae | N |
| *Rhodiola yunnanensis* | Crassulaceae | Angiospermae | N |
| *Sedum aizoon* | Crassulaceae | Angiospermae | Y |
| *Sedum elatinoides* | Crassulaceae | Angiospermae | N |
| *Sedum emarginatum* | Crassulaceae | Angiospermae | N |
| *Sedum lineare* | Crassulaceae | Angiospermae | N |
| *Sedum major* | Crassulaceae | Angiospermae | Y |
| *Sedum odontophyllum* | Crassulaceae | Angiospermae | N |

| | | | |
|---|---|---|---|
| *Sedum sarmentosum* | Crassulaceae | Angiospermae | Y |
| *Sinocrassula indica* | Crassulaceae | Angiospermae | N |
| *Arabis flagellosa* | Cruciferae | Angiospermae | Y |
| *Armoracia rusticana* | Cruciferae | Angiospermae | Y |
| *Brassica campestris* | Cruciferae | Angiospermae | Y |
| *Brassica juncea* | Cruciferae | Angiospermae | Y |
| *Brassica juncea var. gracilis* | Cruciferae | Angiospermae | Y |
| *Capsella bursa-pastoris* | Cruciferae | Angiospermae | Y |
| *Cardamine flexuosa* | Cruciferae | Angiospermae | Y |
| *Cardamine griffithii* | Cruciferae | Angiospermae | Y |
| *Cardamine hirsuta* | Cruciferae | Angiospermae | Y |
| *Cardamine impatiens* | Cruciferae | Angiospermae | Y |
| *Cardamine leucantha* | Cruciferae | Angiospermae | N |
| *Cardamine lyrata* | Cruciferae | Angiospermae | N |
| *Cardamine macrophylla* | Cruciferae | Angiospermae | Y |
| *Cardamine macrophylla var. polyphy* | Cruciferae | Angiospermae | Y |
| *Cardamine scaposa* | Cruciferae | Angiospermae | N |
| *Cardamine tangutorum* | Cruciferae | Angiospermae | N |
| *Cardamine urbaniana* | Cruciferae | Angiospermae | N |
| *Cheiranthus roseus* | Cruciferae | Angiospermae | N |
| *Cochlearia officinalis* | Cruciferae | Angiospermae | Y |
| *Descurainia sophia* | Cruciferae | Angiospermae | Y |
| *Draba oreades* | Cruciferae | Angiospermae | Y |
| *Eruca sativa* | Cruciferae | Angiospermae | Y |
| *Erysimum cheiranthoides* | Cruciferae | Angiospermae | Y |
| *Isatis indigotica* | Cruciferae | Angiospermae | N |
| *Isatis tinctoria* | Cruciferae | Angiospermae | Y |
| *Lepidium apetalum* | Cruciferae | Angiospermae | Y |
| *Lepidium latifolium* | Cruciferae | Angiospermae | Y |
| *Lepidium latifolium var. affine* | Cruciferae | Angiospermae | Y |
| *Lepidium sativum* | Cruciferae | Angiospermae | Y |
| *Lepidium virginicum* | Cruciferae | Angiospermae | Y |
| *Megacarpaea delavayi* | Cruciferae | Angiospermae | N |
| *Nasturtium officinale* | Cruciferae | Angiospermae | Y |
| *Pegaeophyton scapiflorum* | Cruciferae | Angiospermae | N |
| *Pugionium cornutum* | Cruciferae | Angiospermae | N |
| *Raphanus sativus* | Cruciferae | Angiospermae | Y |
| *Raphanus sativus var. longipinnatus* | Cruciferae | Angiospermae | Y |
| *Rorippa dubia* | Cruciferae | Angiospermae | N |
| *Rorippa indica* | Cruciferae | Angiospermae | Y |
| *Sinapis alba* | Cruciferae | Angiospermae | Y |
| *Solms-Laubachia dolichocarpa* | Cruciferae | Angiospermae | N |
| *Solms-Laubachia eurycarpa* | Cruciferae | Angiospermae | N |
| *Solms-Laubachia linearifolia* | Cruciferae | Angiospermae | N |
| *Thlaspi arvense* | Cruciferae | Angiospermae | Y |
| *Actinostemma tenerum* | Cucurbitaceae | Angiospermae | Y |
| *Benincasa hispida* | Cucurbitaceae | Angiospermae | Y |
| *Bolbostemma paniculatum* | Cucurbitaceae | Angiospermae | N |
| *Citrullus lanatus* | Cucurbitaceae | Angiospermae | Y |
| *Cucumis melo* | Cucurbitaceae | Angiospermae | Y |

| | | | |
|---|---|---|---|
| *Cucumis sativus* | Cucurbitaceae | Angiospermae | Y |
| *Cucurbita moschata* | Cucurbitaceae | Angiospermae | Y |
| *Gynostemma pentaphyllum* | Cucurbitaceae | Angiospermae | Y |
| *Hemsleya amabilis* | Cucurbitaceae | Angiospermae | N |
| *Hemsleya graciliflora* | Cucurbitaceae | Angiospermae | N |
| *Lagenaria siceraria* | Cucurbitaceae | Angiospermae | Y |
| *Lagenaria siceraria var. microcarpa* | Cucurbitaceae | Angiospermae | Y |
| *Luffa acutangula* | Cucurbitaceae | Angiospermae | N |
| *Luffa cylindrica* | Cucurbitaceae | Angiospermae | Y |
| *Momordica charantia* | Cucurbitaceae | Angiospermae | Y |
| *Momordica cochinchinensis* | Cucurbitaceae | Angiospermae | N |
| *Siraitia grosvenorii* | Cucurbitaceae | Angiospermae | Y |
| *Solena amplexicaulis* | Cucurbitaceae | Angiospermae | N |
| *Thladiantha hookeri* | Cucurbitaceae | Angiospermae | N |
| *Thladiantha hookeri var. pentadacty* | Cucurbitaceae | Angiospermae | N |
| *Thladiantha sessilifolia* | Cucurbitaceae | Angiospermae | N |
| *Trichosanthes anguina* | Cucurbitaceae | Angiospermae | N |
| *Trichosanthes cucumerina* | Cucurbitaceae | Angiospermae | Y |
| *Trichosanthes jinggangshanica* | Cucurbitaceae | Angiospermae | N |
| *Trichosanthes rosthornii* | Cucurbitaceae | Angiospermae | N |
| *Trichosanthes rosthornii var. multici* | Cucurbitaceae | Angiospermae | N |
| *Zehneria indica* | Cucurbitaceae | Angiospermae | N |
| *Cynomorium songaricum* | Cynomoriacea | Angiospermae | N |
| *Cyperus rotundus* | Cyperaceae | Angiospermae | Y |
| *Heleocharis dulcis* | Cyperaceae | Angiospermae | N |
| *Scirpus yagara* | Cyperaceae | Angiospermae | N |
| *Daphniphyllum calycinum* | Daphniphyllac | Angiospermae | N |
| *Dillenia pentagyna* | Dilleniaceae | Angiospermae | N |
| *Dioscorea alata* | Dioscoreaceae | Angiospermae | Y |
| *Dioscorea althaeoides* | Dioscoreaceae | Angiospermae | N |
| *Dioscorea bulbifera* | Dioscoreaceae | Angiospermae | Y |
| *Dioscorea chingii* | Dioscoreaceae | Angiospermae | N |
| *Dioscorea cirrhosa* | Dioscoreaceae | Angiospermae | N |
| *Dioscorea deltoidea* | Dioscoreaceae | Angiospermae | Y |
| *Dioscorea esquirolii* | Dioscoreaceae | Angiospermae | N |
| *Dioscorea futschauensis* | Dioscoreaceae | Angiospermae | N |
| *Dioscorea glabra* | Dioscoreaceae | Angiospermae | N |
| *Dioscorea gracillima* | Dioscoreaceae | Angiospermae | N |
| *Dioscorea hispida* | Dioscoreaceae | Angiospermae | N |
| *Dioscorea japonica* | Dioscoreaceae | Angiospermae | Y |
| *Dioscorea melanophyma* | Dioscoreaceae | Angiospermae | N |
| *Dioscorea nipponica* | Dioscoreaceae | Angiospermae | Y |
| *Dioscorea opposita* | Dioscoreaceae | Angiospermae | Y |
| *Dioscorea panthaica* | Dioscoreaceae | Angiospermae | N |
| *Dioscorea parviflora* | Dioscoreaceae | Angiospermae | N |
| *Dioscorea septemloba* | Dioscoreaceae | Angiospermae | N |
| *Dioscorea subcalva* | Dioscoreaceae | Angiospermae | N |
| *Dioscorea tokoro* | Dioscoreaceae | Angiospermae | Y |
| *Dioscorea zingiberensis* | Dioscoreaceae | Angiospermae | Y |
| *Ottelia alismoides* | Dioscoreaceae | Angiospermae | Y |

| | | | |
|---|---|---|---|
| *Dipsacus asperoides* | Dipsacaceae | Angiospermae | Y |
| *Dipsacus atropurpureus* | Dipsacaceae | Angiospermae | N |
| *Dipsacus japonicus* | Dipsacaceae | Angiospermae | Y |
| *Morina chinensis* | Dipsacaceae | Angiospermae | N |
| *Pterocephalus hookeri* | Dipsacaceae | Angiospermae | N |
| *Triplostegia grandiflora* | Dipsacaceae | Angiospermae | N |
| *Dipterocarpus retusus* | Dipterocarpace | Angiospermae | Y |
| *Dipterocarpus turbinatus* | Dipterocarpace | Angiospermae | Y |
| *Drosera burmanni* | Droseraceae | Angiospermae | N |
| *Drosera peltata* | Droseraceae | Angiospermae | Y |
| *Drosera peltata var. multisepala* | Droseraceae | Angiospermae | Y |
| *Drosera rotundifolia* | Droseraceae | Angiospermae | Y |
| *Diospyros cathayensis* | Ebenaceae | Angiospermae | Y |
| *Diospyros glaucifolia* | Ebenaceae | Angiospermae | N |
| *Diospyros kaki* | Ebenaceae | Angiospermae | Y |
| *Diospyros lotus* | Ebenaceae | Angiospermae | Y |
| *Diospyros maritima* | Ebenaceae | Angiospermae | Y |
| *Diospyros morrisiana* | Ebenaceae | Angiospermae | Y |
| *Diospyros oleifera* | Ebenaceae | Angiospermae | N |
| *Diospyros siderophylla* | Ebenaceae | Angiospermae | N |
| *Elaeagnus angustifolia* | Elaeagnaceae | Angiospermae | Y |
| *Elaeagnus bockii* | Elaeagnaceae | Angiospermae | N |
| *Elaeagnus glabra* | Elaeagnaceae | Angiospermae | Y |
| *Elaeagnus gonyanthes* | Elaeagnaceae | Angiospermae | N |
| *Elaeagnus henryi* | Elaeagnaceae | Angiospermae | N |
| *Elaeagnus lanceolata* | Elaeagnaceae | Angiospermae | N |
| *Elaeagnus mollis* | Elaeagnaceae | Angiospermae | Y |
| *Elaeagnus multiflora* | Elaeagnaceae | Angiospermae | N |
| *Elaeagnus multiflora var. tenuipes* | Elaeagnaceae | Angiospermae | N |
| *Elaeagnus oxycarpa* | Elaeagnaceae | Angiospermae | N |
| *Elaeagnus pungens* | Elaeagnaceae | Angiospermae | Y |
| *Elaeagnus stellipila* | Elaeagnaceae | Angiospermae | N |
| *Elaeagnus umbellata* | Elaeagnaceae | Angiospermae | Y |
| *Elaeagnus yunnanensis* | Elaeagnaceae | Angiospermae | N |
| *Hippophae thibetana* | Elaeagnaceae | Angiospermae | Y |
| *Elaeocarpus prunifolioides* | Elaeocarpacea | Angiospermae | Y |
| *Empetrum nigrum var. japonicum* | Empetraceae | Angiospermae | Y |
| *Agapetes brandisiana* | Ericaceae | Angiospermae | N |
| *Agapetes mannii* | Ericaceae | Angiospermae | N |
| *Cassiope fastigiata* | Ericaceae | Angiospermae | Y |
| *Craibiodendron stellatum* | Ericaceae | Angiospermae | N |
| *Craibiodendron yunnanense* | Ericaceae | Angiospermae | N |
| *Gaultheria forrestii* | Ericaceae | Angiospermae | Y |
| *Gaultheria leucocarpa* | Ericaceae | Angiospermae | N |
| *Gaultheria leucocarpa var. crenulata* | Ericaceae | Angiospermae | N |
| *Gaultheria leucocarpa var. hirsuta* | Ericaceae | Angiospermae | N |
| *Rhododendron anthopogon* | Ericaceae | Angiospermae | N |
| *Rhododendron anthopogonoides* | Ericaceae | Angiospermae | N |
| *Rhododendron decorum* | Ericaceae | Angiospermae | Y |
| *Rhododendron molle* | Ericaceae | Angiospermae | Y |

| | | | |
|---|---|---|---|
| *Rhododendron naamkwanense var. n* | Ericaceae | Angiospermae | N |
| *Rhododendron ovatum* | Ericaceae | Angiospermae | Y |
| *Rhododendron simsii* | Ericaceae | Angiospermae | Y |
| *Sect. Ponticum* | Ericaceae | Angiospermae | N |
| *Vaccinium dunalianum* | Ericaceae | Angiospermae | N |
| *Vaccinium fragile* | Ericaceae | Angiospermae | N |
| *Vaccinium vitis-idaea* | Ericaceae | Angiospermae | Y |
| *Eriocaulon acutibracteatum* | Eriocaulaceae | Angiospermae | N |
| *Eriocaulon buergerianum* | Eriocaulaceae | Angiospermae | N |
| *Eriocaulon sexangulare* | Eriocaulaceae | Angiospermae | N |
| *Erythroxylum novogranatense* | Erythroxylace | Angiospermae | N |
| *Eucommia ulmoides* | Eucommiaceae | Angiospermae | Y |
| *Alchornea trewioides* | Euphorbiaceae | Angiospermae | Y |
| *Antidesma bunius* | Euphorbiaceae | Angiospermae | Y |
| *Antidesma ghaesembilla* | Euphorbiaceae | Angiospermae | Y |
| *Bischofia javanica* | Euphorbiaceae | Angiospermae | Y |
| *Breynia fruticosa* | Euphorbiaceae | Angiospermae | Y |
| *Breynia retusa* | Euphorbiaceae | Angiospermae | N |
| *Breynia rostrata* | Euphorbiaceae | Angiospermae | N |
| *Breynia vitis-idaea* | Euphorbiaceae | Angiospermae | Y |
| *Bridelia stipularis* | Euphorbiaceae | Angiospermae | N |
| *Bridelia tomentosa* | Euphorbiaceae | Angiospermae | N |
| *Claoxylon indicum* | Euphorbiaceae | Angiospermae | N |
| *Croton crassifolius* | Euphorbiaceae | Angiospermae | N |
| *Croton tiglium* | Euphorbiaceae | Angiospermae | Y |
| *Euphorbia antiquorum* | Euphorbiaceae | Angiospermae | Y |
| *Euphorbia esula* | Euphorbiaceae | Angiospermae | Y |
| *Euphorbia fischeriana* | Euphorbiaceae | Angiospermae | N |
| *Euphorbia griffithii* | Euphorbiaceae | Angiospermae | N |
| *Euphorbia helioscopia* | Euphorbiaceae | Angiospermae | Y |
| *Euphorbia heyneana* | Euphorbiaceae | Angiospermae | N |
| *Euphorbia humifusa* | Euphorbiaceae | Angiospermae | Y |
| *Euphorbia hypericifolia* | Euphorbiaceae | Angiospermae | Y |
| *Euphorbia jolkinii* | Euphorbiaceae | Angiospermae | Y |
| *Euphorbia kansuensis* | Euphorbiaceae | Angiospermae | N |
| *Euphorbia kansui* | Euphorbiaceae | Angiospermae | N |
| *Euphorbia lathylris* | Euphorbiaceae | Angiospermae | Y |
| *Euphorbia milii* | Euphorbiaceae | Angiospermae | Y |
| *Euphorbia milii var. tananarivae* | Euphorbiaceae | Angiospermae | Y |
| *Euphorbia pekinensis* | Euphorbiaceae | Angiospermae | Y |
| *Euphorbia peplus* | Euphorbiaceae | Angiospermae | Y |
| *Euphorbia prolifera* | Euphorbiaceae | Angiospermae | N |
| *Euphorbia pulcherrima* | Euphorbiaceae | Angiospermae | Y |
| *Euphorbia royleana* | Euphorbiaceae | Angiospermae | Y |
| *Euphorbia sieboldiana* | Euphorbiaceae | Angiospermae | N |
| *Euphorbia sikkimensis* | Euphorbiaceae | Angiospermae | N |
| *Euphorbia soongarica* | Euphorbiaceae | Angiospermae | N |
| *Euphorbia thymifolia* | Euphorbiaceae | Angiospermae | Y |
| *Excoecaria venenata* | Euphorbiaceae | Angiospermae | N |
| *Flueggea suffruticosa* | Euphorbiaceae | Angiospermae | Y |

| | | | |
|---|---|---|---|
| *Flueggea virosa* | Euphorbiaceae | Angiospermae | Y |
| *Glochidion eriocarpum* | Euphorbiaceae | Angiospermae | Y |
| *Glochidion hirsutum* | Euphorbiaceae | Angiospermae | N |
| *Glochidion puberum* | Euphorbiaceae | Angiospermae | Y |
| *Glochidion ramiflorum* | Euphorbiaceae | Angiospermae | N |
| *Glochidion sphaerogynum* | Euphorbiaceae | Angiospermae | N |
| *Glochidion zeylanicum* | Euphorbiaceae | Angiospermae | Y |
| *Jatropha curcas* | Euphorbiaceae | Angiospermae | Y |
| *Leptopus pachyphyllus* | Euphorbiaceae | Angiospermae | N |
| *Phyllanthus bodinieri* | Euphorbiaceae | Angiospermae | N |
| *Phyllanthus emblica* | Euphorbiaceae | Angiospermae | Y |
| *Phyllanthus glaucus* | Euphorbiaceae | Angiospermae | N |
| *Phyllanthus niruri* | Euphorbiaceae | Angiospermae | Y |
| *Phyllanthus reticulatus* | Euphorbiaceae | Angiospermae | Y |
| *Phyllanthus tsarongensis* | Euphorbiaceae | Angiospermae | N |
| *Phyllanthus urinaria* | Euphorbiaceae | Angiospermae | Y |
| *Phyllanthus ussuriensis* | Euphorbiaceae | Angiospermae | N |
| *Phyllanthus virgatus* | Euphorbiaceae | Angiospermae | Y |
| *Ricinus communis* | Euphorbiaceae | Angiospermae | Y |
| *Sapium discolor* | Euphorbiaceae | Angiospermae | N |
| *Sapium sebiferum* | Euphorbiaceae | Angiospermae | Y |
| *Sauropus spatulifolius* | Euphorbiaceae | Angiospermae | N |
| *Trigonella foenum-graecum L.* | Fabaceae | Angiospermae | N |
| *Quercus dentata* | Fagaceae | Angiospermae | Y |
| *Quercus mongolica* | Fagaceae | Angiospermae | Y |
| *Quercus mongolica var. grosseserra* | Fagaceae | Angiospermae | Y |
| *Hydnocarpus anthelminthica* | Flacourtiaceae | Angiospermae | N |
| *Xylosma racemosum* | Flacourtiaceae | Angiospermae | N |
| *Flagellaria indica* | Flagellariaceae | Angiospermae | Y |
| *Gentiana apiata* | Gentianaceae | Angiospermae | N |
| *Gentiana officinalis* | Gentianaceae | Angiospermae | N |
| *Gentiana phyllopoda* | Gentianaceae | Angiospermae | N |
| *Gentiana rhodantha* | Gentianaceae | Angiospermae | N |
| *Gentiana triflora* | Gentianaceae | Angiospermae | N |
| *Halenia corniculata* | Gentianaceae | Angiospermae | Y |
| *Halenia elliptica* | Gentianaceae | Angiospermae | N |
| *Swertia diluta var. tosaensis* | Gentianaceae | Angiospermae | N |
| *Swertia mileensis* | Gentianaceae | Angiospermae | N |
| *Swertia mussotii var. flavescens* | Gentianaceae | Angiospermae | N |
| *Swertia pseudochinensis* | Gentianaceae | Angiospermae | N |
| *Veratrilla baillonii* | Gentianaceae | Angiospermae | N |
| *Erodium stephanianum* | Geraniaceae | Angiospermae | Y |
| *Geranium carolinianum* | Geraniaceae | Angiospermae | Y |
| *Geranium delavayi* | Geraniaceae | Angiospermae | N |
| *Geranium nepalense* | Geraniaceae | Angiospermae | Y |
| *Geranium pylzowianum* | Geraniaceae | Angiospermae | N |
| *Geranium sinense* | Geraniaceae | Angiospermae | N |
| *Geranium strictipes* | Geraniaceae | Angiospermae | N |
| *Geranium wilfordii* | Geraniaceae | Angiospermae | Y |
| *Aeschynanthus acuminatus* | Gesneriaceae | Angiospermae | N |

| | | | |
|---|---|---|---|
| *Aeschynanthus austroyunnanensis va* | Gesneriaceae | Angiospermae | N |
| *Boea clarkeana* | Gesneriaceae | Angiospermae | N |
| *Boea hygrometrica* | Gesneriaceae | Angiospermae | Y |
| *Briggsia mihieri* | Gesneriaceae | Angiospermae | N |
| *Chirita eburnea* | Gesneriaceae | Angiospermae | N |
| *Chirita fimbrisepala* | Gesneriaceae | Angiospermae | N |
| *Chirita gueilinensis* | Gesneriaceae | Angiospermae | N |
| *Chirita hamosa* | Gesneriaceae | Angiospermae | N |
| *Chirita linearifolia* | Gesneriaceae | Angiospermae | N |
| *Chirita longgangensis* | Gesneriaceae | Angiospermae | N |
| *Chirita longgangensis var. hongyao* | Gesneriaceae | Angiospermae | N |
| *Chirita medica* | Gesneriaceae | Angiospermae | N |
| *Chirita ophiopogoides* | Gesneriaceae | Angiospermae | N |
| *Chirita parvifolia* | Gesneriaceae | Angiospermae | N |
| *Chirita pinnatifida* | Gesneriaceae | Angiospermae | N |
| *Chirita spinulosa* | Gesneriaceae | Angiospermae | N |
| *Chiritopsis bipinnatifida* | Gesneriaceae | Angiospermae | N |
| *Chiritopsis cordifolia* | Gesneriaceae | Angiospermae | N |
| *Conandron ramondioides* | Gesneriaceae | Angiospermae | Y |
| *Corallodiscus cordatulus* | Gesneriaceae | Angiospermae | N |
| *Corallodiscus flabellatus* | Gesneriaceae | Angiospermae | Y |
| *Corallodiscus kingianus* | Gesneriaceae | Angiospermae | N |
| *Didissandra macrosiphon* | Gesneriaceae | Angiospermae | N |
| *Didissandra sesquifolia* | Gesneriaceae | Angiospermae | N |
| *Hemiboea cavaleriei* | Gesneriaceae | Angiospermae | N |
| *Hemiboea follicularis* | Gesneriaceae | Angiospermae | N |
| *Hemiboea henryi* | Gesneriaceae | Angiospermae | N |
| *Hemiboea omeiensis* | Gesneriaceae | Angiospermae | N |
| *Hemiboea subacaulis* | Gesneriaceae | Angiospermae | N |
| *Hemiboea subcapitata* | Gesneriaceae | Angiospermae | N |
| *Hemiboea subcapitata var. denticula* | Gesneriaceae | Angiospermae | N |
| *Lysionotus aeschynanthoides* | Gesneriaceae | Angiospermae | N |
| *Lysionotus pauciflorus* | Gesneriaceae | Angiospermae | Y |
| *Lysionotus pauciflorus var. latifolius* | Gesneriaceae | Angiospermae | Y |
| *Oreocharis benthamii* | Gesneriaceae | Angiospermae | N |
| *Oreocharis benthamii var. reticulata* | Gesneriaceae | Angiospermae | N |
| *Ornithoboea wildeana* | Gesneriaceae | Angiospermae | N |
| *Paraboea dictyoneura* | Gesneriaceae | Angiospermae | Y |
| *Paraboea rufescens* | Gesneriaceae | Angiospermae | N |
| *Petrocodon dealbatus* | Gesneriaceae | Angiospermae | N |
| *Rhynchoglossum obliquum var. holo* | Gesneriaceae | Angiospermae | N |
| *Apluda mutica* | Gramineae | Angiospermae | Y |
| *Bambusa pervariabilis* | Gramineae | Angiospermae | Y |
| *Bambusa pervariabilis × textilis* | Gramineae | Angiospermae | Y |
| *Bambusa textilis* | Gramineae | Angiospermae | Y |
| *Bambusa textilis var. persistens* | Gramineae | Angiospermae | Y |
| *Coix chinensis* | Gramineae | Angiospermae | N |
| *Cymbopogon citratus* | Gramineae | Angiospermae | Y |
| *Cymbopogon flexuosus* | Gramineae | Angiospermae | Y |
| *Cymbopogon jwarancusa* | Gramineae | Angiospermae | Y |

| | | | |
|---|---|---|---|
| *Cymbopogon nardus* | Gramineae | Angiospermae | Y |
| *Dendrocalamopsis oldhami* | Gramineae | Angiospermae | N |
| *Diandranthus aristatus* | Gramineae | Angiospermae | N |
| *Eleusine indica* | Gramineae | Angiospermae | Y |
| *Eragrostis ferruginea* | Gramineae | Angiospermae | Y |
| *Eragrostis pilosa* | Gramineae | Angiospermae | Y |
| *Eragrostis tenella* | Gramineae | Angiospermae | Y |
| *Hierochloe odorata var. pubescens* | Gramineae | Angiospermae | Y |
| *Imperata koenigii* | Gramineae | Angiospermae | N |
| *Lophatherum gracile* | Gramineae | Angiospermae | Y |
| *Lophatherum sinense* | Gramineae | Angiospermae | N |
| *Miscanthus floridulus* | Gramineae | Angiospermae | Y |
| *Oligostachyum sulcatum* | Gramineae | Angiospermae | N |
| *Phragmites australis* | Gramineae | Angiospermae | Y |
| *Phyllostachys nigra var. henonis* | Gramineae | Angiospermae | Y |
| *Pogonatherum crinitum* | Gramineae | Angiospermae | Y |
| *Saccharum officinarum* | Gramineae | Angiospermae | Y |
| *Saccharum sinense* | Gramineae | Angiospermae | N |
| *Schizostachyum dumetorum* | Gramineae | Angiospermae | N |
| *Setaria geniculata* | Gramineae | Angiospermae | Y |
| *Setaria italica* | Gramineae | Angiospermae | Y |
| *Setaria palmifolia* | Gramineae | Angiospermae | N |
| *Setaria viridis* | Gramineae | Angiospermae | Y |
| *Sinobambusa seminuda* | Gramineae | Angiospermae | N |
| *Calophyllum inophyllum* | Guttiferae | Angiospermae | Y |
| *Calophyllum membranaceum* | Guttiferae | Angiospermae | Y |
| *Cratoxylum cochinchinense* | Guttiferae | Angiospermae | Y |
| *Cratoxylum formosum subsp. prunifl* | Guttiferae | Angiospermae | Y |
| *Garcinia multiflora* | Guttiferae | Angiospermae | Y |
| *Garcinia xanthochymus* | Guttiferae | Angiospermae | Y |
| *Hypericum ascyron* | Guttiferae | Angiospermae | N |
| *Hypericum attenuatum* | Guttiferae | Angiospermae | Y |
| *Hypericum japonicum* | Guttiferae | Angiospermae | Y |
| *Hypericum longistylum* | Guttiferae | Angiospermae | N |
| *Hypericum monogynum* | Guttiferae | Angiospermae | Y |
| *Hypericum patulum* | Guttiferae | Angiospermae | N |
| *Hypericum seniavinii* | Guttiferae | Angiospermae | N |
| *Haloragis micrantha* | Haloragidacea | Angiospermae | N |
| *Myriophyllum spicatum* | Haloragidacea | Angiospermae | Y |
| *Altingia chinensis* | Hamamelidace | Angiospermae | N |
| *Altingia gracilipes* | Hamamelidace | Angiospermae | N |
| *Altingia gracilipes var. serrulata* | Hamamelidace | Angiospermae | N |
| *Liquidambar formosana* | Hamamelidace | Angiospermae | Y |
| *Loropetalum chinense* | Hamamelidace | Angiospermae | Y |
| *Semiliquidambar cathayensis* | Hamamelidace | Angiospermae | N |
| *Illigera cordata* | Hernandiaceae | Angiospermae | N |
| *Illigera cordata var. mollissima* | Hernandiaceae | Angiospermae | N |
| *Illigera grandiflora* | Hernandiaceae | Angiospermae | N |
| *Illigera parviflora* | Hernandiaceae | Angiospermae | N |
| *Aesculus chinensis* | Hippocastanac | Angiospermae | Y |

| | | | |
|---|---|---|---|
| *Aesculus wilsonii* | Hippocastanac | Angiospermae | N |
| *Salacia prinoides* | Hippocrateace | Angiospermae | Y |
| *Iodes balansae* | Icacinaceae | Angiospermae | N |
| *Iodes cirrhosa* | Icacinaceae | Angiospermae | N |
| *Mappianthus iodoides* | Icacinaceae | Angiospermae | N |
| *Pittosporopsis kerrii* | Icacinaceae | Angiospermae | Y |
| *Belamcanda chinensis* | Iridaceae | Angiospermae | Y |
| *Crocosmia crocosmiflora* | Iridaceae | Angiospermae | N |
| *Crocus alatavicus* | Iridaceae | Angiospermae | N |
| *Crocus sativus* | Iridaceae | Angiospermae | Y |
| *Eleutherine plicata* | Iridaceae | Angiospermae | N |
| *Iris anguifuga* | Iridaceae | Angiospermae | N |
| *Iris confusa* | Iridaceae | Angiospermae | N |
| *Iris decora* | Iridaceae | Angiospermae | N |
| *Iris dichotoma* | Iridaceae | Angiospermae | N |
| *Iris japonica* | Iridaceae | Angiospermae | N |
| *Iris lactea var. chinensis* | Iridaceae | Angiospermae | Y |
| *Iris leptophylla* | Iridaceae | Angiospermae | N |
| *Iris pallida* | Iridaceae | Angiospermae | N |
| *Iris tectorum* | Iridaceae | Angiospermae | Y |
| *Engelhardtia roxbughiana* | Juglandaceae | Angiospermae | N |
| *Engelhardtia roxburghiana* | Juglandaceae | Angiospermae | Y |
| *Platycarya strobilacea* | Juglandaceae | Angiospermae | Y |
| *Juncus effusus* | Juncaceae | Angiospermae | Y |
| *Juncus miyiensis* | Juncaceae | Angiospermae | N |
| *Agastache rugosa* | Labiatae | Angiospermae | Y |
| *Ajuga bracteosa* | Labiatae | Angiospermae | N |
| *Ajuga campylantha* | Labiatae | Angiospermae | N |
| *Ajuga ciliata* | Labiatae | Angiospermae | N |
| *Ajuga decumbens* | Labiatae | Angiospermae | Y |
| *Ajuga forrestii* | Labiatae | Angiospermae | N |
| *Ajuga macrosperma* | Labiatae | Angiospermae | N |
| *Ajuga multiflora* | Labiatae | Angiospermae | N |
| *Ajuga nipponensis* | Labiatae | Angiospermae | N |
| *Ajuga pantantha* | Labiatae | Angiospermae | N |
| *Amethystea caerulea* | Labiatae | Angiospermae | Y |
| *Anisochilus carnosus* | Labiatae | Angiospermae | N |
| *Clerodendranthus spicatus* | Labiatae | Angiospermae | N |
| *Clinopodium megalanthum* | Labiatae | Angiospermae | N |
| *Clinopodium polycephalum* | Labiatae | Angiospermae | N |
| *Colquhounia coccinea var. mollis* | Labiatae | Angiospermae | N |
| *Colquhounia elegans* | Labiatae | Angiospermae | N |
| *Colquhounia elegans var. tenuiflora* | Labiatae | Angiospermae | N |
| *Comanthosphace ningpoensis* | Labiatae | Angiospermae | Y |
| *Dracocephalum heterophyllum* | Labiatae | Angiospermae | Y |
| Dracocephalum integrifolium | Labiatae | Angiospermae | Y |
| Dracocephalum tanguticum | Labiatae | Angiospermae | N |
| Elsholtzia blanda | Labiatae | Angiospermae | Y |
| *Elsholtzia bodinieri* | Labiatae | Angiospermae | N |
| *Elsholtzia ciliata* | Labiatae | Angiospermae | Y |

| | | | |
|---|---|---|---|
| *Elsholtzia communis* | Labiatae | Angiospermae | Y |
| *Elsholtzia cypriani* | Labiatae | Angiospermae | Y |
| *Elsholtzia densa* | Labiatae | Angiospermae | Y |
| *Elsholtzia feddei f. remotibracteata* | Labiatae | Angiospermae | N |
| *Elsholtzia flava* | Labiatae | Angiospermae | N |
| *Elsholtzia fruticosa var. fruticosa f. l* | Labiatae | Angiospermae | N |
| *Elsholtzia heterophylla* | Labiatae | Angiospermae | N |
| *Elsholtzia penduliflora* | Labiatae | Angiospermae | N |
| *Elsholtzia rugulosa* | Labiatae | Angiospermae | Y |
| *Elsholtzia souliei* | Labiatae | Angiospermae | N |
| *Elsholtzia splendens* | Labiatae | Angiospermae | N |
| *Elsholtzia strobilifera* | Labiatae | Angiospermae | Y |
| *Epimeredi indica* | Labiatae | Angiospermae | N |
| *Eriophyton wallichii* | Labiatae | Angiospermae | N |
| *Glechoma biondiana* | Labiatae | Angiospermae | N |
| *Glechoma biondiana var. glabrescen* | Labiatae | Angiospermae | N |
| *Glechoma hederacea* | Labiatae | Angiospermae | Y |
| *Glechoma longituba* | Labiatae | Angiospermae | Y |
| *Glechoma sinograndis* | Labiatae | Angiospermae | N |
| *Gomphostemma leptodon* | Labiatae | Angiospermae | N |
| *Gomphostemma microdon* | Labiatae | Angiospermae | Y |
| *Heterolamium debile var. cardiophyl* | Labiatae | Angiospermae | N |
| *Hyptis suaveolens* | Labiatae | Angiospermae | Y |
| *Lagopsis supina* | Labiatae | Angiospermae | N |
| *Lamiophlomis rotata* | Labiatae | Angiospermae | Y |
| *Lamium album* | Labiatae | Angiospermae | Y |
| *Lamium barbatum* | Labiatae | Angiospermae | Y |
| *Leonurus artemisia var. albiflorus* | Labiatae | Angiospermae | Y |
| *Leonurus artemisia var. artemisia* | Labiatae | Angiospermae | Y |
| *Leonurus pseudomacranthus* | Labiatae | Angiospermae | N |
| *Leonurus pseudomacranthus f. leuca* | Labiatae | Angiospermae | N |
| *Leucas ciliata* | Labiatae | Angiospermae | N |
| *Leucas martinicensis* | Labiatae | Angiospermae | Y |
| *Leucas mollissima* | Labiatae | Angiospermae | N |
| *Leucas mollissima var. chinensis* | Labiatae | Angiospermae | N |
| *Leucas zeylanica* | Labiatae | Angiospermae | N |
| *Lycopus lucidus var. hirtus* | Labiatae | Angiospermae | N |
| *Marrubium vulgare* | Labiatae | Angiospermae | Y |
| *Meehania fargesii* | Labiatae | Angiospermae | N |
| *Meehania fargesii var. pedunculata* | Labiatae | Angiospermae | N |
| *Meehania fargesii var. radicans* | Labiatae | Angiospermae | N |
| *Melissa axillaris* | Labiatae | Angiospermae | N |
| *Melissa officinalis* | Labiatae | Angiospermae | Y |
| *Mentha haplocalyx* | Labiatae | Angiospermae | Y |
| *Mentha spicata* | Labiatae | Angiospermae | Y |
| *Mesona chinensis* | Labiatae | Angiospermae | N |
| *Micromeria biflora* | Labiatae | Angiospermae | N |
| *Microtoena omeiensis* | Labiatae | Angiospermae | N |
| *Microtoena patchoulii* | Labiatae | Angiospermae | N |
| *Microtoena pauciflora* | Labiatae | Angiospermae | N |

| | | | |
|---|---|---|---|
| *Mosla cavaleriei* | Labiatae | Angiospermae | N |
| *Mosla chinensis* | Labiatae | Angiospermae | N |
| *Mosla dianthera* | Labiatae | Angiospermae | Y |
| *Mosla scabra* | Labiatae | Angiospermae | Y |
| *Nepeta angustifolia* | Labiatae | Angiospermae | N |
| *Nepeta cataria* | Labiatae | Angiospermae | Y |
| *Nepeta laevigata* | Labiatae | Angiospermae | N |
| *Nosema cochinchinensis* | Labiatae | Angiospermae | N |
| *Ocimum americanum* | Labiatae | Angiospermae | Y |
| *Ocimum basilicum* | Labiatae | Angiospermae | Y |
| *Ocimum basilicum var. pilosum* | Labiatae | Angiospermae | Y |
| *Ocimum gratissimum var. suave* | Labiatae | Angiospermae | N |
| *Ocimum sanctum* | Labiatae | Angiospermae | Y |
| *Origanum vulgare* | Labiatae | Angiospermae | Y |
| *Orthosiphon wulfenioides* | Labiatae | Angiospermae | N |
| *Orthosiphon wulfenioides var. folios* | Labiatae | Angiospermae | N |
| *Panzeria alaschanica* | Labiatae | Angiospermae | Y |
| *Paraphlomis javanica var. coronata* | Labiatae | Angiospermae | Y |
| *Perilla frutescens* | Labiatae | Angiospermae | Y |
| *Phlomis maximowiczii* | Labiatae | Angiospermae | N |
| *Phlomis mongolica* | Labiatae | Angiospermae | N |
| *Phlomis umbrosa* | Labiatae | Angiospermae | Y |
| *Phlomis umbrosa var. australis* | Labiatae | Angiospermae | Y |
| *Phlomis younghusbandii* | Labiatae | Angiospermae | N |
| *Pogostemon auricularius* | Labiatae | Angiospermae | N |
| *Pogostemon cablin* | Labiatae | Angiospermae | Y |
| *Pogostemon esquirolii* | Labiatae | Angiospermae | N |
| *Pogostemon glaber* | Labiatae | Angiospermae | N |
| *Pogostemon nigrescens* | Labiatae | Angiospermae | N |
| *Prunella asiatica* | Labiatae | Angiospermae | N |
| *Prunella vulgaris* | Labiatae | Angiospermae | Y |
| *Rabdosia adenantha* | Labiatae | Angiospermae | N |
| *Rabdosia amethystoides* | Labiatae | Angiospermae | Y |
| *Rabdosia coetsa* | Labiatae | Angiospermae | N |
| *Rabdosia coetsoides* | Labiatae | Angiospermae | N |
| *Rabdosia eriocalyx* | Labiatae | Angiospermae | N |
| *Rabdosia hispida* | Labiatae | Angiospermae | N |
| *Rabdosia japonica* | Labiatae | Angiospermae | N |
| *Rabdosia lophanthoides* | Labiatae | Angiospermae | N |
| *Rabdosia lophanthoides var. gerardi* | Labiatae | Angiospermae | N |
| *Rabdosia megathyrsa* | Labiatae | Angiospermae | N |
| *Rabdosia nervosa* | Labiatae | Angiospermae | N |
| *Rabdosia oresbia* | Labiatae | Angiospermae | N |
| *Rabdosia rosthornii* | Labiatae | Angiospermae | N |
| *Rabdosia rubescens* | Labiatae | Angiospermae | N |
| *Rabdosia sculponeata* | Labiatae | Angiospermae | Y |
| *Rabdosia serra* | Labiatae | Angiospermae | N |
| *Rabdosia ternifolia* | Labiatae | Angiospermae | N |
| *Rabdosia yuennanensis* | Labiatae | Angiospermae | N |
| *Salvia aerea* | Labiatae | Angiospermae | N |

| | | | |
|---|---|---|---|
| *Salvia bowleyana* | Labiatae | Angiospermae | N |
| *Salvia cavaleriei var. simplicifolia* | Labiatae | Angiospermae | N |
| *Salvia chinensis* | Labiatae | Angiospermae | Y |
| *Salvia coccinea* | Labiatae | Angiospermae | N |
| *Salvia digitaloides* | Labiatae | Angiospermae | N |
| *Salvia evansiana* | Labiatae | Angiospermae | N |
| *Salvia kiangsiensis* | Labiatae | Angiospermae | N |
| *Salvia kiaometiensis* | Labiatae | Angiospermae | N |
| *Salvia miltiorrhiza* | Labiatae | Angiospermae | Y |
| *Salvia officinalis* | Labiatae | Angiospermae | Y |
| *Salvia plebeia* | Labiatae | Angiospermae | N |
| *Salvia plectranthoides* | Labiatae | Angiospermae | N |
| *Salvia przewalskii* | Labiatae | Angiospermae | Y |
| *Salvia scapiformis* | Labiatae | Angiospermae | N |
| *Salvia scapiformis var. hirsuta* | Labiatae | Angiospermae | N |
| *Salvia substolonifera* | Labiatae | Angiospermae | N |
| *Salvia trijuga* | Labiatae | Angiospermae | N |
| *Salvia yunnanensis* | Labiatae | Angiospermae | N |
| *Schizonepeta tenuifolia* | Labiatae | Angiospermae | Y |
| *Schnabelia oligophylla* | Labiatae | Angiospermae | N |
| *Scutellaria amoena* | Labiatae | Angiospermae | N |
| *Scutellaria baicalensis* | Labiatae | Angiospermae | Y |
| *Scutellaria barbata* | Labiatae | Angiospermae | Y |
| *Scutellaria caudifolia var. obliquifoli* | Labiatae | Angiospermae | N |
| *Scutellaria discolor* | Labiatae | Angiospermae | Y |
| *Scutellaria discolor var. hirta* | Labiatae | Angiospermae | Y |
| *Scutellaria franchetiana* | Labiatae | Angiospermae | N |
| *Scutellaria likiangensis* | Labiatae | Angiospermae | N |
| *Scutellaria obtusifolia* | Labiatae | Angiospermae | N |
| *Scutellaria omeiensis* | Labiatae | Angiospermae | N |
| *Scutellaria orthocalyx* | Labiatae | Angiospermae | N |
| *Scutellaria quadrilobulata* | Labiatae | Angiospermae | N |
| *Scutellaria scordifolia* | Labiatae | Angiospermae | Y |
| *Scutellaria sessilifolia* | Labiatae | Angiospermae | N |
| *Scutellaria tayloriana* | Labiatae | Angiospermae | N |
| *Scutellaria yunnanensis* | Labiatae | Angiospermae | N |
| *Scutellaria yunnanensis var. salicifol* | Labiatae | Angiospermae | N |
| *Siphocranion macranthum* | Labiatae | Angiospermae | N |
| *Smilacina nokomonticola* | Labiatae | Angiospermae | N |
| *Stachys geobombycis* | Labiatae | Angiospermae | N |
| *Stachys japonica* | Labiatae | Angiospermae | N |
| *Stachys kouyangensis* | Labiatae | Angiospermae | N |
| *Stachys oblongifolia* | Labiatae | Angiospermae | N |
| *Teucrium anlungense* | Labiatae | Angiospermae | N |
| *Teucrium bidentatum* | Labiatae | Angiospermae | N |
| *Teucrium japonicum* | Labiatae | Angiospermae | Y |
| *Teucrium pilosum* | Labiatae | Angiospermae | N |
| *Teucrium quadrifarium* | Labiatae | Angiospermae | Y |
| *Teucrium scordioides* | Labiatae | Angiospermae | N |
| *Teucrium scordium* | Labiatae | Angiospermae | Y |

| | | | |
|---|---|---|---|
| *Teucrium viscidum* | Labiatae | Angiospermae | Y |
| *Akebia quinata* | Lardizabalacea | Angiospermae | Y |
| *Akebia trifoliata* | Lardizabalacea | Angiospermae | Y |
| *Akebia trifoliata subsp. australis* | Lardizabalacea | Angiospermae | Y |
| *Decaisnea insignis* | Lardizabalacea | Angiospermae | N |
| *Holboellia coriacea* | Lardizabalacea | Angiospermae | N |
| *Holboellia fargesii* | Lardizabalacea | Angiospermae | N |
| *Holboellia latifolia var. latifolia* | Lardizabalacea | Angiospermae | N |
| *Sargentodoxa cuneata* | Lardizabalacea | Angiospermae | N |
| *Stauntonia chinensis* | Lardizabalacea | Angiospermae | N |
| *Stauntonia yaoshanensis* | Lardizabalacea | Angiospermae | N |
| *Actinodaphne cupularis* | Lauraceae | Angiospermae | N |
| *Actinodaphne obovata* | Lauraceae | Angiospermae | N |
| *Actinodaphne pilosa* | Lauraceae | Angiospermae | N |
| *Cassytha filiformis* | Lauraceae | Angiospermae | N |
| *Cinnamomum appelianum* | Lauraceae | Angiospermae | N |
| *Cinnamomum austrosinense* | Lauraceae | Angiospermae | Y |
| *Cinnamomum burmannii* | Lauraceae | Angiospermae | Y |
| *Cinnamomum camphora* | Lauraceae | Angiospermae | Y |
| *Cinnamomum camphora var. linaloo* | Lauraceae | Angiospermae | Y |
| *Cinnamomum cassia* | Lauraceae | Angiospermae | Y |
| *Cinnamomum glanduliferum* | Lauraceae | Angiospermae | Y |
| *Cinnamomum jensenianum* | Lauraceae | Angiospermae | N |
| *Cinnamomum pauciflorum* | Lauraceae | Angiospermae | N |
| *Cinnamomum porrectum* | Lauraceae | Angiospermae | Y |
| *Cinnamomum subavenium* | Lauraceae | Angiospermae | Y |
| *Cinnamomum tamala* | Lauraceae | Angiospermae | Y |
| *Cinnamomum wilsonii* | Lauraceae | Angiospermae | N |
| *Cinnamomum zeylanicum* | Lauraceae | Angiospermae | N |
| *Lindera aggregata* | Lauraceae | Angiospermae | Y |
| *Lindera aggregata var. playfairii* | Lauraceae | Angiospermae | Y |
| *Lindera chunii* | Lauraceae | Angiospermae | Y |
| *Lindera communis* | Lauraceae | Angiospermae | Y |
| *Lindera glauca* | Lauraceae | Angiospermae | N |
| *Lindera obtusiloba* | Lauraceae | Angiospermae | Y |
| *Lindera pulcherrima var. attenuata* | Lauraceae | Angiospermae | N |
| *Lindera reflexa* | Lauraceae | Angiospermae | Y |
| *Litsea auriculata* | Lauraceae | Angiospermae | N |
| *Litsea coreana var. sinensis* | Lauraceae | Angiospermae | Y |
| *Litsea cubeba var. cubeba* | Lauraceae | Angiospermae | Y |
| *Litsea cubeba var. cubeba f. obtusifo* | Lauraceae | Angiospermae | Y |
| *Litsea euosma* | Lauraceae | Angiospermae | N |
| *Litsea glutinosa* | Lauraceae | Angiospermae | Y |
| *Litsea mollis* | Lauraceae | Angiospermae | N |
| *Litsea monopetala* | Lauraceae | Angiospermae | Y |
| *Litsea rotundifolia var. oblongifolia* | Lauraceae | Angiospermae | N |
| *Litsea verticillata* | Lauraceae | Angiospermae | Y |
| *Machilus thunbergii* | Lauraceae | Angiospermae | Y |
| *Neocinnamomum delavayi* | Lauraceae | Angiospermae | Y |
| *Neolitsea aurata* | Lauraceae | Angiospermae | Y |

| | | | |
|---|---|---|---|
| *Neolitsea aurata var. chekiangensis* | Lauraceae | Angiospermae | Y |
| *Neolitsea cambodiana* | Lauraceae | Angiospermae | Y |
| *Neolitsea levinei* | Lauraceae | Angiospermae | N |
| *Persea americana* | Lauraceae | Angiospermae | Y |
| *Sassafras tzumu* | Lauraceae | Angiospermae | Y |
| *Barringtonia racemosa* | Lecythidaceae | Angiospermae | Y |
| *Abrus cantoniensis* | Leguminosae | Angiospermae | N |
| *Abrus precatorius* | Leguminosae | Angiospermae | Y |
| *Abrus pulchellus* | Leguminosae | Angiospermae | N |
| *Acacia catechu* | Leguminosae | Angiospermae | N |
| *Acacia farnesiana* | Leguminosae | Angiospermae | Y |
| *Acacia senegal* | Leguminosae | Angiospermae | Y |
| *Acacia sinuata* | Leguminosae | Angiospermae | N |
| *Aeschynomene indica* | Leguminosae | Angiospermae | Y |
| *Afzelia xylocarpa* | Leguminosae | Angiospermae | Y |
| *Albizia julibrissin* | Leguminosae | Angiospermae | Y |
| *Albizia julibrissin f. rosea* | Leguminosae | Angiospermae | Y |
| *Alysicarpus vaginalis* | Leguminosae | Angiospermae | Y |
| *Astragalus adsurgens* | Leguminosae | Angiospermae | Y |
| *Astragalus bhotanensis* | Leguminosae | Angiospermae | Y |
| *Astragalus complanatus* | Leguminosae | Angiospermae | N |
| *Astragalus ernestii* | Leguminosae | Angiospermae | N |
| *Astragalus hoantchy* | Leguminosae | Angiospermae | N |
| *Astragalus membranaceus var. mong* | Leguminosae | Angiospermae | Y |
| *Astragalus scaberrimus* | Leguminosae | Angiospermae | Y |
| *Astragalus variabilis* | Leguminosae | Angiospermae | N |
| *Bauhinia aurea* | Leguminosae | Angiospermae | Y |
| *Bauhinia purpurea* | Leguminosae | Angiospermae | Y |
| *Bauhinia tomentosa* | Leguminosae | Angiospermae | Y |
| *Bauhinia variegata* | Leguminosae | Angiospermae | Y |
| *Butea monosperma* | Leguminosae | Angiospermae | Y |
| *Caesalpinia decapetala* | Leguminosae | Angiospermae | Y |
| *Caesalpinia digyna* | Leguminosae | Angiospermae | N |
| *Caesalpinia minax* | Leguminosae | Angiospermae | N |
| *Caesalpinia sappan* | Leguminosae | Angiospermae | N |
| *Cajanus scarabaeoides* | Leguminosae | Angiospermae | Y |
| *Campylotropis bonatiana* | Leguminosae | Angiospermae | N |
| *Campylotropis delavayi* | Leguminosae | Angiospermae | Y |
| *Campylotropis hirtella* | Leguminosae | Angiospermae | N |
| *Campylotropis pinetorum* | Leguminosae | Angiospermae | Y |
| *Campylotropis pinetorum subsp. velu* | Leguminosae | Angiospermae | Y |
| *Campylotropis polyantha* | Leguminosae | Angiospermae | N |
| *Campylotropis trigonoclada* | Leguminosae | Angiospermae | N |
| *Cassia agnes* | Leguminosae | Angiospermae | N |
| *Cassia fistula* | Leguminosae | Angiospermae | Y |
| *Cassia mimosoides* | Leguminosae | Angiospermae | Y |
| *Cassia occidentalis* | Leguminosae | Angiospermae | Y |
| *Cassia sophera* | Leguminosae | Angiospermae | Y |
| *Cercis chinensis* | Leguminosae | Angiospermae | Y |
| *Christia vespertilionis* | Leguminosae | Angiospermae | Y |

| | | | |
|---|---|---|---|
| *Clitoria hanceana* | Leguminosae | Angiospermae | N |
| *Codariocalyx motorius* | Leguminosae | Angiospermae | Y |
| *Coronilla varia* | Leguminosae | Angiospermae | Y |
| *Crotalaria alata* | Leguminosae | Angiospermae | N |
| *Crotalaria albida* | Leguminosae | Angiospermae | N |
| *Crotalaria assamica* | Leguminosae | Angiospermae | Y |
| *Crotalaria ferruginea* | Leguminosae | Angiospermae | N |
| *Crotalaria juncea* | Leguminosae | Angiospermae | Y |
| *Crotalaria linifolia* | Leguminosae | Angiospermae | Y |
| *Crotalaria mairei* | Leguminosae | Angiospermae | N |
| *Crotalaria medicaginea* | Leguminosae | Angiospermae | Y |
| *Crotalaria micans* | Leguminosae | Angiospermae | N |
| *Crotalaria retusa* | Leguminosae | Angiospermae | Y |
| *Crotalaria sessiliflora* | Leguminosae | Angiospermae | N |
| *Crotalaria spectabilis* | Leguminosae | Angiospermae | Y |
| *Crotalaria verrucosa* | Leguminosae | Angiospermae | Y |
| *Crotalaria zanzibarica* | Leguminosae | Angiospermae | N |
| *Dalbergia hancei* | Leguminosae | Angiospermae | N |
| *Dalbergia hupeana* | Leguminosae | Angiospermae | Y |
| *Dalbergia odorifera* | Leguminosae | Angiospermae | Y |
| *Dalbergia pinnata* | Leguminosae | Angiospermae | Y |
| *Dendrolobium triangulare* | Leguminosae | Angiospermae | Y |
| *Desmodium amethystinum* | Leguminosae | Angiospermae | N |
| *Desmodium caudatum* | Leguminosae | Angiospermae | N |
| *Desmodium heterocarpon* | Leguminosae | Angiospermae | Y |
| *Desmodium microphyllum* | Leguminosae | Angiospermae | Y |
| *Desmodium multiflorum* | Leguminosae | Angiospermae | Y |
| *Desmodium styracifolium* | Leguminosae | Angiospermae | N |
| *Desmodium triflorum* | Leguminosae | Angiospermae | Y |
| *Dichrostachys cinerea* | Leguminosae | Angiospermae | Y |
| *Dumasia forrestii* | Leguminosae | Angiospermae | N |
| *Eriosema chinense* | Leguminosae | Angiospermae | N |
| *Eriosema himalaicum* | Leguminosae | Angiospermae | N |
| *Erythrina caffra* | Leguminosae | Angiospermae | N |
| *Erythrina corallodendron* | Leguminosae | Angiospermae | N |
| *Erythrina senegalensis* | Leguminosae | Angiospermae | N |
| *Erythrina speciosa* | Leguminosae | Angiospermae | N |
| *Erythrina variegata* | Leguminosae | Angiospermae | Y |
| *Euchresta horsfieldii* | Leguminosae | Angiospermae | N |
| *Euchresta tubulosa* | Leguminosae | Angiospermae | N |
| *Flemingia fluminalis* | Leguminosae | Angiospermae | N |
| *Flemingia macrophylla* | Leguminosae | Angiospermae | Y |
| *Flemingia philippinensis* | Leguminosae | Angiospermae | N |
| *Flemingia strobilifera* | Leguminosae | Angiospermae | Y |
| *Fordia cauliflora* | Leguminosae | Angiospermae | N |
| *Galega officinalis* | Leguminosae | Angiospermae | N |
| *Galega officinalis var. hartlandii* | Leguminosae | Angiospermae | N |
| *Galega officinalis var. patus* | Leguminosae | Angiospermae | N |
| *Galega officinalis var. persica* | Leguminosae | Angiospermae | N |
| *Gleditsia fera* | Leguminosae | Angiospermae | Y |

| | | | |
|---|---|---|---|
| *Gleditsia japonica* | Leguminosae | Angiospermae | Y |
| *Gleditsia sinensis* | Leguminosae | Angiospermae | Y |
| *Glycine max* | Leguminosae | Angiospermae | Y |
| *Glycine soja* | Leguminosae | Angiospermae | Y |
| *Glycine soja var. albiflora f. angusti* | Leguminosae | Angiospermae | Y |
| *Glycyrrhiza glabra* | Leguminosae | Angiospermae | Y |
| *Glycyrrhiza inflata* | Leguminosae | Angiospermae | Y |
| *Glycyrrhiza uralensis* | Leguminosae | Angiospermae | Y |
| *Gueldenstaedtia verna subsp. multifl* | Leguminosae | Angiospermae | Y |
| *Gueldenstaedtia verna subsp. multifl* | Leguminosae | Angiospermae | Y |
| *Hedysarum polybotrys* | Leguminosae | Angiospermae | N |
| *Indigofera amblyantha* | Leguminosae | Angiospermae | Y |
| *Indigofera bungeana* | Leguminosae | Angiospermae | N |
| *Indigofera carlesii* | Leguminosae | Angiospermae | N |
| *Indigofera decora var. cooperii* | Leguminosae | Angiospermae | N |
| *Indigofera longispica* | Leguminosae | Angiospermae | N |
| *Indigofera pseudotinctoria* | Leguminosae | Angiospermae | Y |
| *Indigofera squalida* | Leguminosae | Angiospermae | N |
| *Indigofera suffruticosa* | Leguminosae | Angiospermae | Y |
| *Indigofera tinctoria* | Leguminosae | Angiospermae | Y |
| *Kummerowia stipulacea* | Leguminosae | Angiospermae | N |
| *Kummerowia striata* | Leguminosae | Angiospermae | Y |
| *Lablab purpureus* | Leguminosae | Angiospermae | Y |
| *Lespedeza pilosa* | Leguminosae | Angiospermae | Y |
| *Lespedeza tomentosa* | Leguminosae | Angiospermae | N |
| *Lysidice brevicalyx* | Leguminosae | Angiospermae | N |
| *Lysidice rhodostegia* | Leguminosae | Angiospermae | N |
| *Maackia amurensis* | Leguminosae | Angiospermae | Y |
| *Maackia amurensis var. buergeri* | Leguminosae | Angiospermae | Y |
| *Millettia bonatiana* | Leguminosae | Angiospermae | N |
| *Millettia championii* | Leguminosae | Angiospermae | N |
| *Millettia eurybotrya* | Leguminosae | Angiospermae | N |
| *Millettia ichthyochtona* | Leguminosae | Angiospermae | N |
| *Millettia nitida var. minor* | Leguminosae | Angiospermae | N |
| *Millettia oosperma* | Leguminosae | Angiospermae | N |
| *Millettia pachycarpa* | Leguminosae | Angiospermae | N |
| *Millettia speciosa* | Leguminosae | Angiospermae | N |
| *Millettia tsui* | Leguminosae | Angiospermae | N |
| *Mimosa pudica* | Leguminosae | Angiospermae | Y |
| *Mucuna birdwoodiana* | Leguminosae | Angiospermae | N |
| *Mucuna sempervirens* | Leguminosae | Angiospermae | Y |
| *Ormosia henryi* | Leguminosae | Angiospermae | N |
| *Ormosia microphylla* | Leguminosae | Angiospermae | N |
| *Oxytropis aciphylla var. aciphylla* | Leguminosae | Angiospermae | Y |
| *Oxytropis chiliophylla* | Leguminosae | Angiospermae | N |
| *Oxytropis falcata* | Leguminosae | Angiospermae | N |
| *Oxytropis microphylla* | Leguminosae | Angiospermae | Y |
| *Oxytropis racemosa* | Leguminosae | Angiospermae | Y |
| *Pachyrhizus erosus* | Leguminosae | Angiospermae | Y |
| *Parkinsonia aculeata* | Leguminosae | Angiospermae | Y |

| | | | |
|---|---|---|---|
| *Phyllodium elegans* | Leguminosae | Angiospermae | N |
| *Phyllodium pulchellum* | Leguminosae | Angiospermae | N |
| *Physostigma venenosum* | Leguminosae | Angiospermae | N |
| *Pisum sativum* | Leguminosae | Angiospermae | Y |
| *Pithecellobium lucidum* | Leguminosae | Angiospermae | N |
| *Podocarpium duclouxii* | Leguminosae | Angiospermae | N |
| *Podocarpium oldhamii* | Leguminosae | Angiospermae | N |
| *Podocarpium podocarpum var. falla* | Leguminosae | Angiospermae | N |
| *Podocarpium podocarpum var. oxyp* | Leguminosae | Angiospermae | N |
| *Podocarpium podocarpum var. szech* | Leguminosae | Angiospermae | N |
| *Psoralea corylifolia* | Leguminosae | Angiospermae | Y |
| *Pterocarpus indicus* | Leguminosae | Angiospermae | Y |
| *Pueraria lobata* | Leguminosae | Angiospermae | Y |
| *Rhynchosia dielsii* | Leguminosae | Angiospermae | N |
| *Rhynchosia volubilis* | Leguminosae | Angiospermae | Y |
| *Saraca dives* | Leguminosae | Angiospermae | Y |
| *Sesbania grandiflora* | Leguminosae | Angiospermae | Y |
| *Sesbania sesban* | Leguminosae | Angiospermae | Y |
| *Shuteria involucrata* | Leguminosae | Angiospermae | N |
| *Shuteria involucrata var. glabrata* | Leguminosae | Angiospermae | N |
| *Smithia sensitiva* | Leguminosae | Angiospermae | Y |
| *Sophora alopecuroides* | Leguminosae | Angiospermae | Y |
| *Sophora flavescens* | Leguminosae | Angiospermae | Y |
| *Sophora flavescens var. favescens* | Leguminosae | Angiospermae | Y |
| *Sophora flavescens var. kronei* | Leguminosae | Angiospermae | Y |
| *Sophora japonica* | Leguminosae | Angiospermae | Y |
| *Sophora tonkinensis* | Leguminosae | Angiospermae | Y |
| *Spatholobus suberectus* | Leguminosae | Angiospermae | N |
| *Sphaerophysa salsula* | Leguminosae | Angiospermae | Y |
| *Tadehagi triquetrum* | Leguminosae | Angiospermae | Y |
| *Tamarindus indica* | Leguminosae | Angiospermae | Y |
| *Thermopsis chinensis* | Leguminosae | Angiospermae | Y |
| *Thermopsis lanceolata* | Leguminosae | Angiospermae | Y |
| *Trifolium repens* | Leguminosae | Angiospermae | Y |
| *Trigonella foenum-graecum* | Leguminosae | Angiospermae | Y |
| *Uraria crinita* | Leguminosae | Angiospermae | N |
| *Uraria lagopodioides* | Leguminosae | Angiospermae | Y |
| *Uraria picta* | Leguminosae | Angiospermae | N |
| *Vicia amoena* | Leguminosae | Angiospermae | Y |
| *Vicia amurensis* | Leguminosae | Angiospermae | N |
| *Vicia faba* | Leguminosae | Angiospermae | Y |
| *Vicia hirsuta* | Leguminosae | Angiospermae | Y |
| *Vicia kioshanica* | Leguminosae | Angiospermae | N |
| *Vicia kulingiana* | Leguminosae | Angiospermae | N |
| *Vicia pseudorobus* | Leguminosae | Angiospermae | N |
| *Vicia sativa* | Leguminosae | Angiospermae | Y |
| *Vicia sepium* | Leguminosae | Angiospermae | Y |
| *Vicia tetrasperma* | Leguminosae | Angiospermae | Y |
| *Vicia unijuga* | Leguminosae | Angiospermae | N |
| *Vigna radiata* | Leguminosae | Angiospermae | Y |

| | | | |
|---|---|---|---|
| *Vigna umbellata* | Leguminosae | Angiospermae | Y |
| *Vigna vexillata* | Leguminosae | Angiospermae | Y |
| *Zornia gibbosa* | Leguminosae | Angiospermae | Y |
| *Lemna minor* | Lemnaceae | Angiospermae | Y |
| *Spirodela polyrrhiza* | Lemnaceae | Angiospermae | Y |
| *Aletris spicata* | Liliaceae | Angiospermae | Y |
| *Allium fistulosum* | Liliaceae | Angiospermae | Y |
| *Allium macrostemon* | Liliaceae | Angiospermae | Y |
| *Aloe arborescens var. natalensis* | Liliaceae | Angiospermae | Y |
| *Aloe vera var. chinensis* | Liliaceae | Angiospermae | Y |
| *Anemarrhena asphodeloides* | Liliaceae | Angiospermae | Y |
| *Asparagus cochinchinensis* | Liliaceae | Angiospermae | Y |
| *Asparagus filicinus* | Liliaceae | Angiospermae | N |
| *Asparagus lycopodineus* | Liliaceae | Angiospermae | N |
| *Asparagus schoberioides* | Liliaceae | Angiospermae | N |
| *Aspidistra attenuata* | Liliaceae | Angiospermae | N |
| *Aspidistra daibuensis* | Liliaceae | Angiospermae | N |
| *Aspidistra lurida* | Liliaceae | Angiospermae | N |
| *Aspidistra mushaensis* | Liliaceae | Angiospermae | N |
| *Cardiocrinum cathayanum* | Liliaceae | Angiospermae | N |
| *Cardiocrinum giganteum* | Liliaceae | Angiospermae | N |
| *Chlorophytum laxum* | Liliaceae | Angiospermae | N |
| *Convallaria majalis* | Liliaceae | Angiospermae | Y |
| *Cordyline fruticosa* | Liliaceae | Angiospermae | Y |
| *Dianella ensifolia* | Liliaceae | Angiospermae | Y |
| *Disporum cantoniense* | Liliaceae | Angiospermae | N |
| *Disporum sessile* | Liliaceae | Angiospermae | N |
| *Dracaena cochinchinensis* | Liliaceae | Angiospermae | Y |
| *Fritillaria cirrhosa* | Liliaceae | Angiospermae | Y |
| *Fritillaria delavayi* | Liliaceae | Angiospermae | N |
| *Fritillaria karelinii* | Liliaceae | Angiospermae | Y |
| *Fritillaria pallidiflora* | Liliaceae | Angiospermae | N |
| *Fritillaria przewalskii* | Liliaceae | Angiospermae | N |
| *Fritillaria taipaiensis* | Liliaceae | Angiospermae | Y |
| *Fritillaria thunbergii* | Liliaceae | Angiospermae | Y |
| *Fritillaria thunbergii var. chekiange* | Liliaceae | Angiospermae | Y |
| *Fritillaria ussuriensis* | Liliaceae | Angiospermae | Y |
| *Hemerocallis citrina* | Liliaceae | Angiospermae | Y |
| *Hosta plantaginea* | Liliaceae | Angiospermae | Y |
| *Hosta ventricosa* | Liliaceae | Angiospermae | Y |
| *Iphigenia indica* | Liliaceae | Angiospermae | Y |
| *Lilium brownii* | Liliaceae | Angiospermae | Y |
| *Lilium brownii var. viridulum* | Liliaceae | Angiospermae | Y |
| *Lilium concolor* | Liliaceae | Angiospermae | N |
| *Lilium dauricum* | Liliaceae | Angiospermae | N |
| *Lilium lancifolium* | Liliaceae | Angiospermae | Y |
| *Lilium pumilum* | Liliaceae | Angiospermae | Y |
| *Lilium rosthornii* | Liliaceae | Angiospermae | N |
| *Lilium speciosum var. gloriosoides* | Liliaceae | Angiospermae | Y |
| *Lilium sulphureum* | Liliaceae | Angiospermae | N |

| | | | |
|---|---|---|---|
| *Lloydia tibetica* | Liliaceae | Angiospermae | N |
| *Notholirion macrophyllum* | Liliaceae | Angiospermae | N |
| *Ophiopogon chingii* | Liliaceae | Angiospermae | N |
| *Ophiopogon japonicus* | Liliaceae | Angiospermae | Y |
| *Ophiopogon zingiberaceus* | Liliaceae | Angiospermae | N |
| *Polygonatum odoratum* | Liliaceae | Angiospermae | Y |
| *Polygonatum sibiricum* | Liliaceae | Angiospermae | Y |
| *Reineckia carnea* | Liliaceae | Angiospermae | Y |
| *Rohdea japonica* | Liliaceae | Angiospermae | Y |
| *Smilacina formosana* | Liliaceae | Angiospermae | N |
| *Smilax glabra* | Liliaceae | Angiospermae | Y |
| *Smilax mairei* | Liliaceae | Angiospermae | N |
| *Smilax nipponica* | Liliaceae | Angiospermae | N |
| *Smilax riparia* | Liliaceae | Angiospermae | N |
| *Smilax scobinicaulis* | Liliaceae | Angiospermae | N |
| *Tulipa edulis* | Liliaceae | Angiospermae | N |
| *Veratrum mengtzeanum* | Liliaceae | Angiospermae | N |
| *Veratrum stenophyllum* | Liliaceae | Angiospermae | N |
| *Veratrum taliense* | Liliaceae | Angiospermae | N |
| *Linum usitatissimum* | Linaceae | Angiospermae | Y |
| *Reinwardtia indica* | Linaceae | Angiospermae | Y |
| *Tirpitzia ovoidea* | Linaceae | Angiospermae | Y |
| *Tirpitzia sinensis* | Linaceae | Angiospermae | N |
| *Buddleja asiatica* | Loganiaceae | Angiospermae | Y |
| *Buddleja caryopteridifolia* | Loganiaceae | Angiospermae | N |
| *Buddleja davidii* | Loganiaceae | Angiospermae | Y |
| *Buddleja fallowiana* | Loganiaceae | Angiospermae | N |
| *Buddleja lindleyana* | Loganiaceae | Angiospermae | Y |
| *Buddleja madagascariensis* | Loganiaceae | Angiospermae | N |
| *Buddleja officinalis* | Loganiaceae | Angiospermae | N |
| *Gardneria angustifolia* | Loganiaceae | Angiospermae | N |
| *Gardneria multiflora* | Loganiaceae | Angiospermae | Y |
| *Gelsemium elegans* | Loganiaceae | Angiospermae | N |
| *Mitrasacme pygmaea* | Loganiaceae | Angiospermae | Y |
| *Strychnos angustiflora* | Loganiaceae | Angiospermae | N |
| *Strychnos cathayensis* | Loganiaceae | Angiospermae | N |
| *Strychnos ignatii* | Loganiaceae | Angiospermae | N |
| *Strychnos nitida* | Loganiaceae | Angiospermae | N |
| *Strychnos nux-vomica* | Loganiaceae | Angiospermae | Y |
| *Strychnos ovata* | Loganiaceae | Angiospermae | N |
| *Strychnos umbellata* | Loganiaceae | Angiospermae | N |
| *Strychnos wallichiana* | Loganiaceae | Angiospermae | N |
| *Helixanthera parasitica* | Loranthaceae | Angiospermae | N |
| *Loranthus tanakae* | Loranthaceae | Angiospermae | N |
| *Macrosolen cochinchinensis* | Loranthaceae | Angiospermae | N |
| *Scurrula parasitica* | Loranthaceae | Angiospermae | N |
| *Taxillus caloreas* | Loranthaceae | Angiospermae | N |
| *Taxillus chinensis* | Loranthaceae | Angiospermae | N |
| *Taxillus delavayi* | Loranthaceae | Angiospermae | N |
| *Taxillus levinei* | Loranthaceae | Angiospermae | N |

| | | | |
|---|---|---|---|
| *Taxillus nigrans* | Loranthaceae | Angiospermae | N |
| *Taxillus sutchuenensis var. sutchuen* | Loranthaceae | Angiospermae | Y |
| *Viscum coloratum* | Loranthaceae | Angiospermae | N |
| *Viscum liquidambaricolum* | Loranthaceae | Angiospermae | N |
| *Viscum nudum* | Loranthaceae | Angiospermae | N |
| *Viscum ovalifolium* | Loranthaceae | Angiospermae | N |
| *Lagerstroemia indica* | Lythraceae | Angiospermae | Y |
| *Lagerstroemia indica f. alba* | Lythraceae | Angiospermae | Y |
| *Lagerstroemia speciosa* | Lythraceae | Angiospermae | Y |
| *Lagerstroemia subcostata* | Lythraceae | Angiospermae | Y |
| *Lawsonia inermis* | Lythraceae | Angiospermae | Y |
| *Lythrum salicaria* | Lythraceae | Angiospermae | Y |
| *Woodfordia fruticosa* | Lythraceae | Angiospermae | Y |
| *Illicium difengpi* | Magnoliaceae | Angiospermae | Y |
| *Illicium henryi* | Magnoliaceae | Angiospermae | N |
| *Illicium jiadifengpi var. jiadifengpi* | Magnoliaceae | Angiospermae | N |
| *Illicium lanceolatum* | Magnoliaceae | Angiospermae | N |
| *Illicium tsangii* | Magnoliaceae | Angiospermae | N |
| *Illicium verum* | Magnoliaceae | Angiospermae | Y |
| *Kadsura coccinea* | Magnoliaceae | Angiospermae | N |
| *Kadsura heteroclita* | Magnoliaceae | Angiospermae | N |
| *Kadsura interior* | Magnoliaceae | Angiospermae | N |
| *Kadsura japonica* | Magnoliaceae | Angiospermae | Y |
| *Kadsura longipedunculata* | Magnoliaceae | Angiospermae | N |
| *Liriodendron chinense* | Magnoliaceae | Angiospermae | Y |
| *Magnolia biondii* | Magnoliaceae | Angiospermae | N |
| *Magnolia coco* | Magnoliaceae | Angiospermae | N |
| *Magnolia denudata* | Magnoliaceae | Angiospermae | Y |
| *Magnolia grandiflora* | Magnoliaceae | Angiospermae | Y |
| *Magnolia hypoleuca* | Magnoliaceae | Angiospermae | N |
| *Magnolia liliflora* | Magnoliaceae | Angiospermae | Y |
| *Magnolia officinalis* | Magnoliaceae | Angiospermae | Y |
| *Magnolia officinalis subsp. biloba* | Magnoliaceae | Angiospermae | Y |
| *Magnolia pilocarpa* | Magnoliaceae | Angiospermae | N |
| *Magnolia rostrata* | Magnoliaceae | Angiospermae | N |
| *Magnolia sieboldii* | Magnoliaceae | Angiospermae | Y |
| *Magnolia sinensis* | Magnoliaceae | Angiospermae | N |
| *Magnolia wilsonii* | Magnoliaceae | Angiospermae | N |
| *Mahonia decipiens* | Magnoliaceae | Angiospermae | N |
| *Mahonia fortunei* | Magnoliaceae | Angiospermae | Y |
| *Mahonia gracilipes* | Magnoliaceae | Angiospermae | N |
| *Manglietia fordiana* | Magnoliaceae | Angiospermae | N |
| *Manglietia patungensis* | Magnoliaceae | Angiospermae | N |
| *Michelia alba* | Magnoliaceae | Angiospermae | N |
| *Michelia figo* | Magnoliaceae | Angiospermae | Y |
| *Michelia maudiae* | Magnoliaceae | Angiospermae | Y |
| *Schisandra chinensis* | Magnoliaceae | Angiospermae | Y |
| *Schisandra henryi* | Magnoliaceae | Angiospermae | N |
| *Schisandra incarnata* | Magnoliaceae | Angiospermae | N |
| *Schisandra neglecta* | Magnoliaceae | Angiospermae | N |

| | | | |
|---|---|---|---|
| *Schisandra propinqua* | Magnoliaceae | Angiospermae | N |
| *Schisandra sphenanthera* | Magnoliaceae | Angiospermae | Y |
| *Abelmoschus manihot* | Malvaceae | Angiospermae | N |
| *Abelmoschus moschatus* | Malvaceae | Angiospermae | Y |
| *Abelmoschus muliensis* | Malvaceae | Angiospermae | N |
| *Abelmoschus sagittifolius* | Malvaceae | Angiospermae | N |
| *Abutilon indicum* | Malvaceae | Angiospermae | Y |
| *Abutilon theophrasti* | Malvaceae | Angiospermae | Y |
| *Althaea officinalis* | Malvaceae | Angiospermae | Y |
| *Althaea rosea* | Malvaceae | Angiospermae | Y |
| *Hibiscus mutabilis* | Malvaceae | Angiospermae | Y |
| *Hibiscus mutabilis f. mutablis* | Malvaceae | Angiospermae | Y |
| *Hibiscus radiatus* | Malvaceae | Angiospermae | N |
| *Hibiscus syriacus* | Malvaceae | Angiospermae | Y |
| *Hibiscus trionum* | Malvaceae | Angiospermae | N |
| *Malva sinensis* | Malvaceae | Angiospermae | Y |
| *Malva verticillata* | Malvaceae | Angiospermae | Y |
| *Malvastrum coromandelianum* | Malvaceae | Angiospermae | Y |
| *Sida acuta* | Malvaceae | Angiospermae | Y |
| *Sida szechuensis* | Malvaceae | Angiospermae | Y |
| *Urena lobata* | Malvaceae | Angiospermae | Y |
| *Maranta arundinacea* | Marantaceae | Angiospermae | Y |
| *Phrynium capitatum* | Marantaceae | Angiospermae | Y |
| *Allomorphia balansae* | Melastomatace | Angiospermae | N |
| *Blastus apricus* | Melastomatace | Angiospermae | N |
| *Blastus cavaleriei* | Melastomatace | Angiospermae | N |
| *Blastus cochinchinensis* | Melastomatace | Angiospermae | N |
| *Blastus dunnianus* | Melastomatace | Angiospermae | N |
| *Blastus pauciflorus* | Melastomatace | Angiospermae | N |
| *Bredia amoena* | Melastomatace | Angiospermae | N |
| *Bredia esquirolii var. cordata* | Melastomatace | Angiospermae | N |
| *Bredia quadrangularis* | Melastomatace | Angiospermae | N |
| *Bredia sinensis* | Melastomatace | Angiospermae | N |
| *Melastoma affine* | Melastomatace | Angiospermae | Y |
| *Melastoma candidum* | Melastomatace | Angiospermae | Y |
| *Melastoma dodecandrum* | Melastomatace | Angiospermae | N |
| *Melastoma normale* | Melastomatace | Angiospermae | N |
| *Melastoma sanguineum* | Melastomatace | Angiospermae | N |
| *Osbeckia chinensis* | Melastomatace | Angiospermae | N |
| *Osbeckia crinita* | Melastomatace | Angiospermae | N |
| *Osbeckia paludosa* | Melastomatace | Angiospermae | N |
| *Oxyspora paniculata* | Melastomatace | Angiospermae | N |
| *Phyllagathis cavaleriei* | Melastomatace | Angiospermae | N |
| *Phyllagathis cavaleriei var. tankahke* | Melastomatace | Angiospermae | N |
| *Phyllagathis elattandra* | Melastomatace | Angiospermae | N |
| *Phyllagathis fordii* | Melastomatace | Angiospermae | N |
| *Sarcopyramis nepalensis* | Melastomatace | Angiospermae | N |
| *Sonerila cantonensis* | Melastomatace | Angiospermae | N |
| *Sonerila rivularis* | Melastomatace | Angiospermae | N |
| *Styrophyton caudatum* | Melastomatace | Angiospermae | N |

| | | | |
|---|---|---|---|
| *Cipadessa cinerascens* | Meliaceae | Angiospermae | Y |
| *Khaya senegalensis* | Meliaceae | Angiospermae | N |
| *Melia azedarach* | Meliaceae | Angiospermae | Y |
| *Melia toosendan* | Meliaceae | Angiospermae | Y |
| *Munronia sinica* | Meliaceae | Angiospermae | N |
| *Toona sinensis* | Meliaceae | Angiospermae | Y |
| *Cyclea barbata* | Menispermace | Angiospermae | N |
| *Diploclisia glaucescens* | Menispermace | Angiospermae | N |
| *Fibraurea recisa* | Menispermace | Angiospermae | Y |
| *Hypserpa nitida* | Menispermace | Angiospermae | N |
| *Sinomenium acutum* | Menispermace | Angiospermae | Y |
| *Stephania cepharantha* | Menispermace | Angiospermae | N |
| *Stephania dielsiana* | Menispermace | Angiospermae | N |
| *Stephania epigaea* | Menispermace | Angiospermae | N |
| *Stephania glabra* | Menispermace | Angiospermae | N |
| *Stephania hainanensis* | Menispermace | Angiospermae | N |
| *Stephania japonica* | Menispermace | Angiospermae | Y |
| *Stephania succifera* | Menispermace | Angiospermae | N |
| *Stephania sutchuenensis* | Menispermace | Angiospermae | N |
| *Stephania tetrandra* | Menispermace | Angiospermae | Y |
| *Tinospora crispa* | Menispermace | Angiospermae | N |
| *Tinospora sagittata* | Menispermace | Angiospermae | N |
| *Tinospora sagittata var. craveniana* | Menispermace | Angiospermae | N |
| *Tinospora sagittata var. yunnanensis* | Menispermace | Angiospermae | N |
| *Tinospora sinensis* | Menispermace | Angiospermae | Y |
| *Artocarpus nitidus* | Moraceae | Angiospermae | Y |
| *Artocarpus nitidus subsp. lingnanens* | Moraceae | Angiospermae | Y |
| *Cannabis sativa* | Moraceae | Angiospermae | Y |
| *Cannabis sativa subsp. indica* | Moraceae | Angiospermae | Y |
| *Cudrania cochinchinensis* | Moraceae | Angiospermae | Y |
| *Cudrania tricuspidata* | Moraceae | Angiospermae | Y |
| *Ficus carica* | Moraceae | Angiospermae | Y |
| *Ficus hirta* | Moraceae | Angiospermae | Y |
| *Ficus pumila* | Moraceae | Angiospermae | Y |
| *Ficus variolosa* | Moraceae | Angiospermae | Y |
| *Humulus scandens* | Moraceae | Angiospermae | Y |
| *Morus alba* | Moraceae | Angiospermae | Y |
| *Morus mongolica* | Moraceae | Angiospermae | N |
| *Musa basjoo* | Musaceae | Angiospermae | Y |
| *Musa wilsonii* | Musaceae | Angiospermae | N |
| *Musella lasiocarpa* | Musaceae | Angiospermae | N |
| *Bontia daphnoides* | Myoporaceae | Angiospermae | N |
| *Myrica rubra* | Myricaceae | Angiospermae | Y |
| *Myristica fragrans* | Myristicaceae | Angiospermae | Y |
| *Ardisia affinis* | Myrsinaceae | Angiospermae | N |
| *Ardisia alyxiaefolia* | Myrsinaceae | Angiospermae | N |
| *Ardisia brevicaulis* | Myrsinaceae | Angiospermae | N |
| *Ardisia brunnescens* | Myrsinaceae | Angiospermae | N |
| *Ardisia chinensis* | Myrsinaceae | Angiospermae | Y |
| *Ardisia crenata* | Myrsinaceae | Angiospermae | Y |

| | | | |
|---|---|---|---|
| *Ardisia crispa* | Myrsinaceae | Angiospermae | Y |
| *Ardisia crispa var. amplifolia* | Myrsinaceae | Angiospermae | Y |
| *Ardisia crispa var. dielsii* | Myrsinaceae | Angiospermae | Y |
| *Ardisia densilepidotula* | Myrsinaceae | Angiospermae | N |
| *Ardisia elegans* | Myrsinaceae | Angiospermae | N |
| *Ardisia ensifolia* | Myrsinaceae | Angiospermae | N |
| *Ardisia faberi* | Myrsinaceae | Angiospermae | N |
| *Ardisia filiformis* | Myrsinaceae | Angiospermae | N |
| *Ardisia gigantifolia* | Myrsinaceae | Angiospermae | N |
| *Ardisia humilis* | Myrsinaceae | Angiospermae | N |
| *Ardisia japonica* | Myrsinaceae | Angiospermae | Y |
| *Ardisia maculosa* | Myrsinaceae | Angiospermae | N |
| *Ardisia mamillata* | Myrsinaceae | Angiospermae | N |
| *Ardisia nervosa* | Myrsinaceae | Angiospermae | N |
| *Ardisia primulaefolia* | Myrsinaceae | Angiospermae | N |
| *Ardisia punctata* | Myrsinaceae | Angiospermae | N |
| *Ardisia pusilla* | Myrsinaceae | Angiospermae | Y |
| *Ardisia quinquegona* | Myrsinaceae | Angiospermae | Y |
| *Ardisia villosa* | Myrsinaceae | Angiospermae | N |
| *Embelia laeta* | Myrsinaceae | Angiospermae | N |
| *Embelia longifolia* | Myrsinaceae | Angiospermae | N |
| *Embelia parviflora* | Myrsinaceae | Angiospermae | N |
| *Embelia ribes* | Myrsinaceae | Angiospermae | N |
| *Embelia rudis* | Myrsinaceae | Angiospermae | N |
| *Embelia subcoriacea* | Myrsinaceae | Angiospermae | N |
| *Maesa japonica* | Myrsinaceae | Angiospermae | Y |
| *Maesa perlarius* | Myrsinaceae | Angiospermae | N |
| *Myrsine africana* | Myrsinaceae | Angiospermae | Y |
| *Rapanea neriifolia* | Myrsinaceae | Angiospermae | N |
| *Baeckea frutescens* | Myrtaceae | Angiospermae | N |
| *Cleistocalyx operculatus* | Myrtaceae | Angiospermae | N |
| *Eucalyptus robusta* | Myrtaceae | Angiospermae | Y |
| *Rhodomyrtus tomentosa* | Myrtaceae | Angiospermae | Y |
| *Nepenthes mirabilis* | Nepenthaceae | Angiospermae | N |
| *Boerhavia diffusa* | Nyctaginaceae | Angiospermae | Y |
| *Bougainvillea glabra* | Nyctaginaceae | Angiospermae | N |
| *Mirabilis jalapa* | Nyctaginaceae | Angiospermae | Y |
| *Oxybaphus himalaicus var. chinensis* | Nyctaginaceae | Angiospermae | N |
| *Euryale ferox* | Nymphaeaceae | Angiospermae | Y |
| *Nelumbo nucifera* | Nymphaeaceae | Angiospermae | Y |
| *Camptotheca acuminata* | Nyssaceae | Angiospermae | Y |
| *Erythropalum scandens* | Olacaceae | Angiospermae | N |
| *Schoepfia fragrans* | Olacaceae | Angiospermae | N |
| *Forsythia suspensa* | Oleaceae | Angiospermae | Y |
| *Fraxinus americana* | Oleaceae | Angiospermae | Y |
| *Fraxinus bungeana* | Oleaceae | Angiospermae | N |
| *Fraxinus chinensis* | Oleaceae | Angiospermae | Y |
| *Fraxinus malacophylla* | Oleaceae | Angiospermae | Y |
| *Fraxinus paxiana* | Oleaceae | Angiospermae | N |
| *Fraxinus pennsylvanica* | Oleaceae | Angiospermae | Y |

| | | | |
|---|---|---|---|
| *Fraxinus pennsylvanica var. subinteg* | Oleaceae | Angiospermae | Y |
| *Fraxinus retusifoliolata* | Oleaceae | Angiospermae | N |
| *Fraxinus rhynchophylla* | Oleaceae | Angiospermae | Y |
| *Fraxinus szaboana* | Oleaceae | Angiospermae | N |
| *Jasminum albicalyx* | Oleaceae | Angiospermae | N |
| *Jasminum coarctatum* | Oleaceae | Angiospermae | N |
| *Jasminum duclouxii* | Oleaceae | Angiospermae | N |
| *Jasminum elongatum* | Oleaceae | Angiospermae | N |
| *Jasminum laurifolium* | Oleaceae | Angiospermae | Y |
| *Jasminum pentaneurum* | Oleaceae | Angiospermae | Y |
| *Jasminum sambac* | Oleaceae | Angiospermae | Y |
| *Jasminum seguinii* | Oleaceae | Angiospermae | N |
| *Ligustrum lucidum* | Oleaceae | Angiospermae | Y |
| *Ligustrum quihoui* | Oleaceae | Angiospermae | N |
| *Ligustrum sinense* | Oleaceae | Angiospermae | Y |
| *Syringa pinnatifolia* | Oleaceae | Angiospermae | N |
| *Syringa reticulata var. amurensis* | Oleaceae | Angiospermae | Y |
| *Epilobium angustifolium* | Onagraceae | Angiospermae | Y |
| *Epilobium hirsutum* | Onagraceae | Angiospermae | Y |
| *Epilobium palustre* | Onagraceae | Angiospermae | Y |
| *Ludwigia adscendens* | Onagraceae | Angiospermae | Y |
| *Ludwigia epilobioides* | Onagraceae | Angiospermae | N |
| *Ludwigia hyssopifolia* | Onagraceae | Angiospermae | Y |
| *Oenothera glazioviana* | Onagraceae | Angiospermae | Y |
| *Oenothera rosea* | Onagraceae | Angiospermae | Y |
| *Oenothera stricta* | Onagraceae | Angiospermae | N |
| *Anoectochilus roxburghii* | Orchidaceae | Angiospermae | Y |
| *Bulbophyllum omerandrum* | Orchidaceae | Angiospermae | N |
| *Diplomeris pulchella* | Orchidaceae | Angiospermae | N |
| *Gastrochilus platycalcaratus* | Orchidaceae | Angiospermae | N |
| *Goodyera procera* | Orchidaceae | Angiospermae | N |
| *Goodyera repens* | Orchidaceae | Angiospermae | Y |
| *Goodyera schlechtendaliana* | Orchidaceae | Angiospermae | N |
| *Gymnadenia conopsea* | Orchidaceae | Angiospermae | Y |
| *Gymnadenia crassinervis* | Orchidaceae | Angiospermae | N |
| *Gymnadenia emeiensis* | Orchidaceae | Angiospermae | N |
| *Gymnadenia orchidis* | Orchidaceae | Angiospermae | N |
| *Habenaria dentata* | Orchidaceae | Angiospermae | Y |
| *Habenaria viridiflora* | Orchidaceae | Angiospermae | N |
| *Herminium monorchis* | Orchidaceae | Angiospermae | Y |
| *Ludisia discolor* | Orchidaceae | Angiospermae | Y |
| *Nervilia aragoana* | Orchidaceae | Angiospermae | N |
| *Nervilia fordii* | Orchidaceae | Angiospermae | N |
| *Nervilia plicata* | Orchidaceae | Angiospermae | N |
| *Orchis latifolia* | Orchidaceae | Angiospermae | N |
| *Peristylus densus* | Orchidaceae | Angiospermae | N |
| *Peristylus tentaculatus* | Orchidaceae | Angiospermae | N |
| *Spiranthes sinensis* | Orchidaceae | Angiospermae | Y |
| *Aeginetia indica* | Orobanchacea | Angiospermae | N |
| *Boschniakia himalaica* | Orobanchacea | Angiospermae | N |

| | | | |
|---|---|---|---|
| *Boschniakia rossica* | Orobanchaceae | Angiospermae | N |
| *Cistanche deserticola* | Orobanchaceae | Angiospermae | N |
| *Cistanche tubulosa* | Orobanchaceae | Angiospermae | N |
| *Orobanche coerulescens* | Orobanchaceae | Angiospermae | N |
| *Averrhoa carambola* | Oxalidaceae | Angiospermae | Y |
| *Biophytum fruticosum* | Oxalidaceae | Angiospermae | N |
| *Biophytum sensitivum* | Oxalidaceae | Angiospermae | Y |
| *Oxalis acetosella* | Oxalidaceae | Angiospermae | Y |
| *Oxalis acetosella subsp. griffithii* | Oxalidaceae | Angiospermae | Y |
| *Oxalis corniculata* | Oxalidaceae | Angiospermae | Y |
| *Oxalis corymbosa* | Oxalidaceae | Angiospermae | Y |
| *Areca catechu* | Palmae | Angiospermae | Y |
| *Calamus macrorrhynchus* | Palmae | Angiospermae | N |
| *Cocos nucifera* | Palmae | Angiospermae | Y |
| *Corypha umbraculifera* | Palmae | Angiospermae | N |
| *Livistona chinensis* | Palmae | Angiospermae | Y |
| *Rhapis excelsa* | Palmae | Angiospermae | Y |
| *Trachycarpus fortunei* | Palmae | Angiospermae | Y |
| *Pandanus furcatus* | Pandanaceae | Angiospermae | Y |
| *Pandanus tectorius* | Pandanaceae | Angiospermae | Y |
| *Pandanus gressitii* | Pandanaceae | Angiospermae | N |
| *Chelidonium majus* | Papaveraceae | Angiospermae | Y |
| *Corydalis adunca* | Papaveraceae | Angiospermae | N |
| *Corydalis appendiculata* | Papaveraceae | Angiospermae | N |
| *Corydalis balansae* | Papaveraceae | Angiospermae | N |
| *Corydalis boweri* | Papaveraceae | Angiospermae | N |
| *Corydalis bulbillifera* | Papaveraceae | Angiospermae | N |
| *Corydalis cheilanthifolia* | Papaveraceae | Angiospermae | N |
| *Corydalis dasyptera* | Papaveraceae | Angiospermae | N |
| *Corydalis daviddi* | Papaveraceae | Angiospermae | N |
| *Corydalis decumbens* | Papaveraceae | Angiospermae | N |
| *Corydalis densispica* | Papaveraceae | Angiospermae | N |
| *Corydalis edulis* | Papaveraceae | Angiospermae | N |
| *Corydalis fangshanensis* | Papaveraceae | Angiospermae | N |
| *Corydalis hendersonii* | Papaveraceae | Angiospermae | N |
| *Corydalis humicola* | Papaveraceae | Angiospermae | N |
| *Corydalis humosa* | Papaveraceae | Angiospermae | N |
| *Corydalis imbricata* | Papaveraceae | Angiospermae | N |
| *Corydalis incisa* | Papaveraceae | Angiospermae | N |
| *Corydalis kingii var. megalantha* | Papaveraceae | Angiospermae | N |
| *Corydalis latiloba* | Papaveraceae | Angiospermae | N |
| *Corydalis linarioides* | Papaveraceae | Angiospermae | N |
| *Corydalis linstowiana* | Papaveraceae | Angiospermae | N |
| *Corydalis meifolia* | Papaveraceae | Angiospermae | N |
| *Corydalis ochotensis* | Papaveraceae | Angiospermae | N |
| *Corydalis ophiocarpa* | Papaveraceae | Angiospermae | N |
| *Corydalis pallida* | Papaveraceae | Angiospermae | N |
| *Corydalis pseudomucronata* | Papaveraceae | Angiospermae | N |
| *Corydalis racemosa* | Papaveraceae | Angiospermae | N |
| *Corydalis repens* | Papaveraceae | Angiospermae | N |

| | | | |
|---|---|---|---|
| *Corydalis rheinbebeniana* | Papaveraceae | Angiospermae | N |
| *Corydalis rheinbebeniana var. rheini* | Papaveraceae | Angiospermae | N |
| *Corydalis saxicola* | Papaveraceae | Angiospermae | N |
| *Corydalis schanginii* | Papaveraceae | Angiospermae | Y |
| *Corydalis sheareri* | Papaveraceae | Angiospermae | N |
| *Corydalis temulifolia* | Papaveraceae | Angiospermae | N |
| *Corydalis trachycarpa* | Papaveraceae | Angiospermae | N |
| *Corydalis tsayulensis* | Papaveraceae | Angiospermae | N |
| *Corydalis yanhusuo* | Papaveraceae | Angiospermae | Y |
| *Dactylicapnos scandens* | Papaveraceae | Angiospermae | N |
| *Dactylicapnos torulosa* | Papaveraceae | Angiospermae | N |
| *Dicentra macrantha* | Papaveraceae | Angiospermae | N |
| *Dicentra spectabilis* | Papaveraceae | Angiospermae | Y |
| *Dicranostigma leptopodum* | Papaveraceae | Angiospermae | Y |
| *Eomecon chionantha* | Papaveraceae | Angiospermae | N |
| *Hylomecon japonica* | Papaveraceae | Angiospermae | Y |
| *Hylomecon japonica var. subincisa* | Papaveraceae | Angiospermae | Y |
| *Hypecoum erectum* | Papaveraceae | Angiospermae | Y |
| *Hypecoum leptocarpum* | Papaveraceae | Angiospermae | N |
| *Macleaya cordata* | Papaveraceae | Angiospermae | Y |
| *Macleaya microcarpa* | Papaveraceae | Angiospermae | N |
| *Meconopsis napaulensis* | Papaveraceae | Angiospermae | N |
| *Meconopsis punicea* | Papaveraceae | Angiospermae | N |
| *Meconopsis quintuplinervia* | Papaveraceae | Angiospermae | N |
| *Meconopsis racemosa* | Papaveraceae | Angiospermae | N |
| *Papaver rhoeas* | Papaveraceae | Angiospermae | Y |
| *Papaver somniferum* | Papaveraceae | Angiospermae | Y |
| *Stylophorum lasiocarpum* | Papaveraceae | Angiospermae | N |
| *Passiflora coerulea* | Passifloraceae | Angiospermae | Y |
| *Passiflora cupiformis* | Passifloraceae | Angiospermae | N |
| *Passiflora edulis* | Passifloraceae | Angiospermae | Y |
| *Passiflora foetida* | Passifloraceae | Angiospermae | Y |
| *Passiflora henryi* | Passifloraceae | Angiospermae | N |
| *Passiflora jugorum* | Passifloraceae | Angiospermae | N |
| *Passiflora wilsonii* | Passifloraceae | Angiospermae | N |
| *Sesamum indicum* | Pedaliaceae | Angiospermae | Y |
| *Philydrum lanuginosum* | Philydraceae | Angiospermae | Y |
| *Phryma leptostachya subsp. asiatica* | Phrymaceae | Angiospermae | Y |
| *Phytolacca acinosa* | Phytolaccaceae | Angiospermae | Y |
| *Phytolacca americana* | Phytolaccaceae | Angiospermae | Y |
| *Peperomia tetraphylla* | Piperaceae | Angiospermae | Y |
| *Piper arboricola* | Piperaceae | Angiospermae | N |
| *Piper betle* | Piperaceae | Angiospermae | Y |
| *Piper boehmeriaefolium var. tonkine* | Piperaceae | Angiospermae | N |
| *Piper flaviflorum* | Piperaceae | Angiospermae | Y |
| *Piper hancei* | Piperaceae | Angiospermae | Y |
| *Piper longum* | Piperaceae | Angiospermae | Y |
| *Piper mullesua* | Piperaceae | Angiospermae | N |
| *Piper nigrum* | Piperaceae | Angiospermae | Y |
| *Piper retrofractum* | Piperaceae | Angiospermae | N |

| | | | |
|---|---|---|---|
| *Piper sarmentosum* | Piperaceae | Angiospermae | Y |
| *Piper wallichii* | Piperaceae | Angiospermae | N |
| *Piper yunnanense* | Piperaceae | Angiospermae | N |
| *Pittosporum crispulum* | Pittosporaceae | Angiospermae | N |
| *Pittosporum glabratum* | Pittosporaceae | Angiospermae | N |
| *Pittosporum kerrii* | Pittosporaceae | Angiospermae | N |
| *Pittosporum perryanum* | Pittosporaceae | Angiospermae | N |
| *Pittosporum podocarpum* | Pittosporaceae | Angiospermae | N |
| *Pittosporum trigonocarpum* | Pittosporaceae | Angiospermae | N |
| *Veronica anagallis-aquatica Linnaeu* | Plantaginaceae | Angiospermae | N |
| *Ceratostigma minus* | Plumbaginacea | Angiospermae | N |
| *Ceratostigma minus f. mimus* | Plumbaginacea | Angiospermae | N |
| *Ceratostigma willmottianum* | Plumbaginacea | Angiospermae | N |
| *Limonium aureum* | Plumbaginacea | Angiospermae | N |
| *Limonium aureum var. potaninii* | Plumbaginacea | Angiospermae | N |
| *Limonium gmelinii* | Plumbaginacea | Angiospermae | Y |
| *Limonium wrightii* | Plumbaginacea | Angiospermae | N |
| *Plumbagella micrantha* | Plumbaginacea | Angiospermae | N |
| *Plumbago auriculata* | Plumbaginacea | Angiospermae | Y |
| *Plumbago zeylanica* | Plumbaginacea | Angiospermae | Y |
| *Polygala arillata* | Polygalaceae | Angiospermae | N |
| *Polygala arvensis* | Polygalaceae | Angiospermae | N |
| *Polygala barbellata* | Polygalaceae | Angiospermae | N |
| *Polygala caudata* | Polygalaceae | Angiospermae | N |
| *Polygala crotalarioides* | Polygalaceae | Angiospermae | N |
| *Polygala fallax* | Polygalaceae | Angiospermae | N |
| *Polygala glomerata* | Polygalaceae | Angiospermae | N |
| *Polygala glomerata var. villosa* | Polygalaceae | Angiospermae | N |
| *Polygala hybrida* | Polygalaceae | Angiospermae | Y |
| *Polygala isocarpa* | Polygalaceae | Angiospermae | N |
| *Polygala japonica* | Polygalaceae | Angiospermae | N |
| *Polygala koi* | Polygalaceae | Angiospermae | N |
| *Polygala persicariifolia* | Polygalaceae | Angiospermae | N |
| *Polygala sibirica* | Polygalaceae | Angiospermae | Y |
| *Polygala sibirica var. megalopha* | Polygalaceae | Angiospermae | Y |
| *Polygala subopposita* | Polygalaceae | Angiospermae | N |
| *Polygala tatarinowii* | Polygalaceae | Angiospermae | Y |
| *Polygala tenuifolia* | Polygalaceae | Angiospermae | Y |
| *Polygala tricornis* | Polygalaceae | Angiospermae | N |
| *Polygala wattersii* | Polygalaceae | Angiospermae | N |
| *Salomonia cantoniensis* | Polygalaceae | Angiospermae | N |
| *Salomonia cantoniensis var. edentulo* | Polygalaceae | Angiospermae | N |
| *Securidaca inappendiculata* | Polygalaceae | Angiospermae | N |
| *Fagopyrum dibotrys* | Polygonaceae | Angiospermae | Y |
| *Fagopyrum esculentum* | Polygonaceae | Angiospermae | Y |
| *Fagopyrum tataricum* | Polygonaceae | Angiospermae | Y |
| *Fallopia denticulata* | Polygonaceae | Angiospermae | N |
| *Fallopia multiflora* | Polygonaceae | Angiospermae | Y |
| *Fallopia multiflora var. ciliinerve* | Polygonaceae | Angiospermae | Y |
| *Polygonum amplexicaule* | Polygonaceae | Angiospermae | Y |

| | | | |
|---|---|---|---|
| *Polygonum amplexicaule var. sinens* | Polygonaceae | Angiospermae | Y |
| *Polygonum aviculare* | Polygonaceae | Angiospermae | Y |
| *Polygonum bistorta* | Polygonaceae | Angiospermae | Y |
| *Polygonum capitatum* | Polygonaceae | Angiospermae | Y |
| *Polygonum chinense* | Polygonaceae | Angiospermae | Y |
| *Polygonum hydropiper* | Polygonaceae | Angiospermae | Y |
| *Polygonum japonicum* | Polygonaceae | Angiospermae | N |
| *Polygonum orientale* | Polygonaceae | Angiospermae | N |
| *Polygonum paleaceum* | Polygonaceae | Angiospermae | N |
| *Polygonum runcinatum var. sinense* | Polygonaceae | Angiospermae | N |
| *Polygonum sieboldii* | Polygonaceae | Angiospermae | N |
| *Polygonum suffultum* | Polygonaceae | Angiospermae | N |
| *Polygonum tinctorium* | Polygonaceae | Angiospermae | N |
| *Polygonum viviparum* | Polygonaceae | Angiospermae | Y |
| *Pongamia pinnata* | Polygonaceae | Angiospermae | Y |
| *Pteroxygonum giraldii* | Polygonaceae | Angiospermae | N |
| *Reynoutria japonica* | Polygonaceae | Angiospermae | Y |
| *Rheum officinale* | Polygonaceae | Angiospermae | N |
| *Rheum palmatum* | Polygonaceae | Angiospermae | Y |
| *Rheum tanguticum* | Polygonaceae | Angiospermae | N |
| *Rumex acetosa* | Polygonaceae | Angiospermae | Y |
| *Rumex japonicus* | Polygonaceae | Angiospermae | Y |
| *Rumex nepalensis* | Polygonaceae | Angiospermae | Y |
| *Eichhornia crassipes* | Pontederiaceae | Angiospermae | Y |
| *Portulaca grandiflora* | Portulacaceae | Angiospermae | Y |
| *Portulaca oleracea* | Portulacaceae | Angiospermae | Y |
| *Portulaca pilosa* | Portulacaceae | Angiospermae | Y |
| *Portulaca quadrifida* | Portulacaceae | Angiospermae | Y |
| *Talinum paniculatum* | Portulacaceae | Angiospermae | Y |
| *Potamogeton pectinatus* | Potamogetona | Angiospermae | Y |
| *Androsace sarmentosa* | Primulaceae | Angiospermae | N |
| *Androsace strigillosa* | Primulaceae | Angiospermae | N |
| *Androsace tapete* | Primulaceae | Angiospermae | N |
| *Androsace umbellata* | Primulaceae | Angiospermae | N |
| *Lysimachia barystachys* | Primulaceae | Angiospermae | Y |
| *Lysimachia candida* | Primulaceae | Angiospermae | N |
| *Lysimachia capillipes* | Primulaceae | Angiospermae | N |
| *Lysimachia christinae* | Primulaceae | Angiospermae | Y |
| *Lysimachia circaeoides* | Primulaceae | Angiospermae | N |
| *Lysimachia clethroides* | Primulaceae | Angiospermae | Y |
| *Lysimachia congestiflora* | Primulaceae | Angiospermae | Y |
| *Lysimachia decurrens* | Primulaceae | Angiospermae | N |
| *Lysimachia foenum-graecum* | Primulaceae | Angiospermae | N |
| *Lysimachia fortunei* | Primulaceae | Angiospermae | Y |
| *Lysimachia insignis* | Primulaceae | Angiospermae | N |
| *Lysimachia klattiana* | Primulaceae | Angiospermae | Y |
| *Lysimachia lobelioides* | Primulaceae | Angiospermae | Y |
| *Lysimachia longipes* | Primulaceae | Angiospermae | N |
| *Lysimachia paridiformis* | Primulaceae | Angiospermae | N |
| *Primula atrodentata* | Primulaceae | Angiospermae | Y |

| | | | |
|---|---|---|---|
| *Primula faberi* | Primulaceae | Angiospermae | N |
| *Primula forbesii* | Primulaceae | Angiospermae | N |
| *Primula forrestii* | Primulaceae | Angiospermae | N |
| *Primula sikkimensis* | Primulaceae | Angiospermae | N |
| *Primula tangutica* | Primulaceae | Angiospermae | N |
| *Heliciopsis terminalis* | Proteaceae | Angiospermae | N |
| *Punica granatum* | Punicaceae | Angiospermae | Y |
| *Pyrola calliantha var. calliantha* | Pyrolaceae | Angiospermae | Y |
| *Pyrola decorata* | Pyrolaceae | Angiospermae | N |
| *Aconitum anthoroideum* | Ranunculaceae | Angiospermae | N |
| *Aconitum austroyunnanense* | Ranunculaceae | Angiospermae | N |
| *Aconitum barbatum var. hispidum* | Ranunculaceae | Angiospermae | N |
| *Aconitum barbatum var. puberulum* | Ranunculaceae | Angiospermae | N |
| *Aconitum brachypodum* | Ranunculaceae | Angiospermae | N |
| *Aconitum brachypodum var. laxiflor* | Ranunculaceae | Angiospermae | N |
| *Aconitum carmichaeli var. hwangsho* | Ranunculaceae | Angiospermae | N |
| *Aconitum chasmanthum* | Ranunculaceae | Angiospermae | Y |
| *Aconitum chilienshanicum* | Ranunculaceae | Angiospermae | N |
| *Aconitum coreanum* | Ranunculaceae | Angiospermae | Y |
| *Aconitum episcopale* | Ranunculaceae | Angiospermae | N |
| *Aconitum flavum* | Ranunculaceae | Angiospermae | N |
| *Aconitum gymnandrum* | Ranunculaceae | Angiospermae | Y |
| *Aconitum hemsleyanum* | Ranunculaceae | Angiospermae | N |
| *Aconitum hemsleyanum var. circinat* | Ranunculaceae | Angiospermae | N |
| *Aconitum karakolicum* | Ranunculaceae | Angiospermae | N |
| *Aconitum kongboense* | Ranunculaceae | Angiospermae | N |
| *Aconitum kusnezoffii* | Ranunculaceae | Angiospermae | N |
| *Aconitum longilobum* | Ranunculaceae | Angiospermae | N |
| *Aconitum nagarum* | Ranunculaceae | Angiospermae | N |
| *Aconitum nagarum var. heterotrichu* | Ranunculaceae | Angiospermae | N |
| *Aconitum nagarum var. heterotrichu* | Ranunculaceae | Angiospermae | N |
| *Aconitum nagarum var. lasiandrum* | Ranunculaceae | Angiospermae | N |
| Aconitum naviculare | Ranunculaceae | Angiospermae | N |
| Aconitum pendulum | Ranunculaceae | Angiospermae | N |
| Aconitum polyschistum | Ranunculaceae | Angiospermae | N |
| *Aconitum pomeense* | Ranunculaceae | Angiospermae | N |
| *Aconitum racemulosum* | Ranunculaceae | Angiospermae | N |
| *Aconitum richardsonianum var. pseu* | Ranunculaceae | Angiospermae | N |
| *Aconitum rotundifolium* | Ranunculaceae | Angiospermae | Y |
| *Aconitum scaposum* | Ranunculaceae | Angiospermae | N |
| *Aconitum scaposum var. vaginatum* | Ranunculaceae | Angiospermae | N |
| *Aconitum sessiliflorum* | Ranunculaceae | Angiospermae | N |
| *Aconitum sinomontanum* | Ranunculaceae | Angiospermae | N |
| *Aconitum sinomontanum var. angust* | Ranunculaceae | Angiospermae | N |
| *Aconitum soongaricum* | Ranunculaceae | Angiospermae | Y |
| *Aconitum spicatum* | Ranunculaceae | Angiospermae | N |
| *Aconitum sungpanense* | Ranunculaceae | Angiospermae | N |
| *Aconitum taipeicum* | Ranunculaceae | Angiospermae | N |
| *Aconitum tanguticum* | Ranunculaceae | Angiospermae | N |
| *Aconitum transsectum* | Ranunculaceae | Angiospermae | N |

| | | | |
|---|---|---|---|
| *Aconitum vilmorinianum* | Ranunculaceae | Angiospermae | N |
| *Aconitum vilmorinianum var. altifidu* | Ranunculaceae | Angiospermae | N |
| *Actaea asiatica* | Ranunculaceae | Angiospermae | N |
| *Adonis amurensis* | Ranunculaceae | Angiospermae | N |
| *Adonis chrysocyatha* | Ranunculaceae | Angiospermae | N |
| *Adonis coerulea* | Ranunculaceae | Angiospermae | N |
| *Adonis sutchuenensis* | Ranunculaceae | Angiospermae | N |
| *Anemone altaica* | Ranunculaceae | Angiospermae | Y |
| *Anemone amurensis* | Ranunculaceae | Angiospermae | N |
| *Anemone davidii* | Ranunculaceae | Angiospermae | N |
| *Anemone demissa* | Ranunculaceae | Angiospermae | N |
| *Anemone flaccida* | Ranunculaceae | Angiospermae | N |
| *Anemone hupehensis* | Ranunculaceae | Angiospermae | Y |
| *Anemone hupehensis var. hupehensis* | Ranunculaceae | Angiospermae | Y |
| *Anemone hupehensis var. japonica* | Ranunculaceae | Angiospermae | Y |
| *Anemone imbricata* | Ranunculaceae | Angiospermae | N |
| *Anemone obtusiloba subsp. ovalifolia* | Ranunculaceae | Angiospermae | N |
| *Anemone raddeana* | Ranunculaceae | Angiospermae | N |
| *Anemone reflexa* | Ranunculaceae | Angiospermae | N |
| *Anemone rivularis* | Ranunculaceae | Angiospermae | Y |
| *Anemone rivularis var. flore-minore* | Ranunculaceae | Angiospermae | Y |
| *Anemone tomentosa* | Ranunculaceae | Angiospermae | Y |
| *Anemone vitifolia* | Ranunculaceae | Angiospermae | Y |
| *Aquilegia incurvata* | Ranunculaceae | Angiospermae | Y |
| *Aquilegia oxysepala* | Ranunculaceae | Angiospermae | N |
| *Aquilegia parviflora* | Ranunculaceae | Angiospermae | N |
| *Aquilegia viridiflora* | Ranunculaceae | Angiospermae | N |
| *Asteropyrum cavaleriei* | Ranunculaceae | Angiospermae | N |
| *Beesia calthifolia* | Ranunculaceae | Angiospermae | N |
| *Calathodes oxycarpa* | Ranunculaceae | Angiospermae | N |
| *Callianthemum taipaicum* | Ranunculaceae | Angiospermae | N |
| *Caltha palustris* | Ranunculaceae | Angiospermae | Y |
| *Caltha palustris var. sibirica* | Ranunculaceae | Angiospermae | Y |
| *Caltha scaposa* | Ranunculaceae | Angiospermae | N |
| *Cimicifuga acerina* | Ranunculaceae | Angiospermae | N |
| *Cimicifuga dahurica* | Ranunculaceae | Angiospermae | N |
| *Cimicifuga foetida* | Ranunculaceae | Angiospermae | N |
| *Cimicifuga foetida var. foliolosa* | Ranunculaceae | Angiospermae | N |
| *Cimicifuga heracleifolia* | Ranunculaceae | Angiospermae | N |
| *Cimicifuga nanchuenensis* | Ranunculaceae | Angiospermae | N |
| *Cimicifuga simplex* | Ranunculaceae | Angiospermae | N |
| *Clematis aethusifolia* | Ranunculaceae | Angiospermae | N |
| *Clematis alsomitrifolia* | Ranunculaceae | Angiospermae | N |
| *Clematis apiifolia* | Ranunculaceae | Angiospermae | Y |
| *Clematis apiifolia var. obtusidentata* | Ranunculaceae | Angiospermae | Y |
| *Clematis argentilucida* | Ranunculaceae | Angiospermae | N |
| *Clematis armandii* | Ranunculaceae | Angiospermae | N |
| *Clematis brevicaudata* | Ranunculaceae | Angiospermae | N |
| *Clematis chinensis* | Ranunculaceae | Angiospermae | N |
| *Clematis chrysocoma* | Ranunculaceae | Angiospermae | N |

| | | | |
|---|---|---|---|
| *Clematis courtoisii* | Ranunculaceae | Angiospermae | N |
| *Clematis crassifolia* | Ranunculaceae | Angiospermae | N |
| *Clematis fasciculiflora* | Ranunculaceae | Angiospermae | N |
| *Clematis filamentosa* | Ranunculaceae | Angiospermae | N |
| *Clematis finetiana* | Ranunculaceae | Angiospermae | N |
| *Clematis florida* | Ranunculaceae | Angiospermae | Y |
| *Clematis glauca* | Ranunculaceae | Angiospermae | N |
| *Clematis gouriana* | Ranunculaceae | Angiospermae | N |
| *Clematis henryi* | Ranunculaceae | Angiospermae | N |
| *Clematis heracleifolia* | Ranunculaceae | Angiospermae | N |
| *Clematis hexapetala* | Ranunculaceae | Angiospermae | N |
| *Clematis huchouensis* | Ranunculaceae | Angiospermae | N |
| *Clematis kerriana* | Ranunculaceae | Angiospermae | N |
| *Clematis koreana* | Ranunculaceae | Angiospermae | N |
| *Clematis lancifolia* | Ranunculaceae | Angiospermae | N |
| *Clematis leschenaultiana* | Ranunculaceae | Angiospermae | N |
| *Clematis loureiroana* | Ranunculaceae | Angiospermae | N |
| *Clematis meyeniana* | Ranunculaceae | Angiospermae | N |
| *Clematis meyeniana var. granulata* | Ranunculaceae | Angiospermae | N |
| *Clematis montana* | Ranunculaceae | Angiospermae | N |
| *Clematis peterae* | Ranunculaceae | Angiospermae | N |
| *Clematis potaninii* | Ranunculaceae | Angiospermae | N |
| *Clematis quinquefoliolata* | Ranunculaceae | Angiospermae | N |
| *Clematis rehderiana* | Ranunculaceae | Angiospermae | N |
| *Clematis tangutica* | Ranunculaceae | Angiospermae | N |
| *Clematis terniflora* | Ranunculaceae | Angiospermae | N |
| *Clematis terniflora var. mandshurica* | Ranunculaceae | Angiospermae | Y |
| *Clematis trichocarpa* | Ranunculaceae | Angiospermae | N |
| *Clematis uncinata* | Ranunculaceae | Angiospermae | N |
| *Clematis zygophylla* | Ranunculaceae | Angiospermae | N |
| *Coptis chinensis* | Ranunculaceae | Angiospermae | Y |
| *Coptis chinensis var. brevisepala* | Ranunculaceae | Angiospermae | Y |
| *Coptis deltoidea* | Ranunculaceae | Angiospermae | Y |
| *Coptis omeiensis* | Ranunculaceae | Angiospermae | N |
| *Coptis teeta* | Ranunculaceae | Angiospermae | N |
| *Delphinium albocoeruleum* | Ranunculaceae | Angiospermae | N |
| *Delphinium anthriscifolium* | Ranunculaceae | Angiospermae | N |
| *Delphinium anthriscifolium var. maj* | Ranunculaceae | Angiospermae | N |
| *Delphinium bonvalotii* | Ranunculaceae | Angiospermae | N |
| *Delphinium bonvalotii var. eriostylu* | Ranunculaceae | Angiospermae | N |
| *Delphinium brunonianum* | Ranunculaceae | Angiospermae | N |
| *Delphinium candelabrum* | Ranunculaceae | Angiospermae | N |
| *Delphinium candelabrum var. monan* | Ranunculaceae | Angiospermae | N |
| *Delphinium chefoense* | Ranunculaceae | Angiospermae | N |
| *Delphinium delavayi* | Ranunculaceae | Angiospermae | N |
| *Delphinium delavayi var. pogonanth* | Ranunculaceae | Angiospermae | N |
| *Delphinium densiflorum* | Ranunculaceae | Angiospermae | N |
| *Delphinium grandiflorum* | Ranunculaceae | Angiospermae | N |
| *Delphinium honanense var. piliferum* | Ranunculaceae | Angiospermae | N |
| *Delphinium kamaonense var. glabres* | Ranunculaceae | Angiospermae | N |

| | | | |
|---|---|---|---|
| *Delphinium omeiense* | Ranunculaceae | Angiospermae | N |
| *Delphinium pylzowii* | Ranunculaceae | Angiospermae | N |
| *Delphinium pylzowii var. trigynum* | Ranunculaceae | Angiospermae | N |
| *Delphinium taliense var. pubipes* | Ranunculaceae | Angiospermae | N |
| *Delphinium yunnanense* | Ranunculaceae | Angiospermae | N |
| *Dichocarpum auriculatum* | Ranunculaceae | Angiospermae | N |
| *Dichocarpum basilare* | Ranunculaceae | Angiospermae | N |
| *Dichocarpum dalzielii* | Ranunculaceae | Angiospermae | N |
| *Dichocarpum trifoliolatum* | Ranunculaceae | Angiospermae | N |
| *Helleborus thibetanus* | Ranunculaceae | Angiospermae | N |
| *Hepatica nobilis var. asiatica* | Ranunculaceae | Angiospermae | Y |
| *Isopyrum manshuricum* | Ranunculaceae | Angiospermae | N |
| *Leptopyrum fumarioides* | Ranunculaceae | Angiospermae | N |
| *Nigella glandulifera* | Ranunculaceae | Angiospermae | N |
| *Paeonia delavayi* | Ranunculaceae | Angiospermae | N |
| *Paeonia delavayi var. angustiloba* | Ranunculaceae | Angiospermae | N |
| *Paeonia delavayi var. lutea* | Ranunculaceae | Angiospermae | N |
| *Paeonia emodi* | Ranunculaceae | Angiospermae | Y |
| *Paeonia lactiflora* | Ranunculaceae | Angiospermae | Y |
| *Paeonia mairei* | Ranunculaceae | Angiospermae | N |
| *Paeonia obovata* | Ranunculaceae | Angiospermae | Y |
| *Paeonia obovata var. willmottiae* | Ranunculaceae | Angiospermae | Y |
| *Paeonia sinjiangensis* | Ranunculaceae | Angiospermae | N |
| *Paeonia suffruticosa* | Ranunculaceae | Angiospermae | Y |
| *Paeonia suffruticosa var. papaverace* | Ranunculaceae | Angiospermae | Y |
| *Paeonia veitchii* | Ranunculaceae | Angiospermae | N |
| *Paraquilegia microphylla* | Ranunculaceae | Angiospermae | N |
| *Pulsatilla ambigua* | Ranunculaceae | Angiospermae | N |
| *Pulsatilla campanella* | Ranunculaceae | Angiospermae | Y |
| *Pulsatilla cernua* | Ranunculaceae | Angiospermae | Y |
| *Pulsatilla chinensis* | Ranunculaceae | Angiospermae | Y |
| *Pulsatilla dahurica* | Ranunculaceae | Angiospermae | N |
| *Pulsatilla turczaninovii* | Ranunculaceae | Angiospermae | N |
| *Ranunculus arvensis* | Ranunculaceae | Angiospermae | Y |
| *Ranunculus cantoniensis* | Ranunculaceae | Angiospermae | Y |
| *Ranunculus chinensis* | Ranunculaceae | Angiospermae | N |
| *Ranunculus glareosus* | Ranunculaceae | Angiospermae | N |
| *Ranunculus japonicus* | Ranunculaceae | Angiospermae | Y |
| *Ranunculus lingua* | Ranunculaceae | Angiospermae | Y |
| *Ranunculus sceleratus* | Ranunculaceae | Angiospermae | Y |
| *Ranunculus sieboldii* | Ranunculaceae | Angiospermae | Y |
| *Ranunculus tanguticus* | Ranunculaceae | Angiospermae | N |
| *Ranunculus ternatus* | Ranunculaceae | Angiospermae | N |
| *Semiaquilegia adoxoides* | Ranunculaceae | Angiospermae | Y |
| *Souliea vaginata* | Ranunculaceae | Angiospermae | N |
| *Thalictrum acutifolium* | Ranunculaceae | Angiospermae | N |
| *Thalictrum alpinum var. elatum* | Ranunculaceae | Angiospermae | Y |
| *Thalictrum aquilegifolium var. sibiri* | Ranunculaceae | Angiospermae | Y |
| *Thalictrum atriplex* | Ranunculaceae | Angiospermae | N |
| *Thalictrum delavayi* | Ranunculaceae | Angiospermae | Y |

| | | | |
|---|---|---|---|
| *Thalictrum faberi* | Ranunculaceae | Angiospermae | N |
| *Thalictrum foetidum* | Ranunculaceae | Angiospermae | Y |
| *Thalictrum foliolosum* | Ranunculaceae | Angiospermae | N |
| *Thalictrum glandulosissimum* | Ranunculaceae | Angiospermae | N |
| *Thalictrum glandulosissimum var. ch* | Ranunculaceae | Angiospermae | N |
| *Thalictrum ichangense* | Ranunculaceae | Angiospermae | N |
| *Thalictrum javanicum* | Ranunculaceae | Angiospermae | N |
| *Thalictrum microgynum* | Ranunculaceae | Angiospermae | N |
| *Thalictrum minus* | Ranunculaceae | Angiospermae | Y |
| *Thalictrum minus var. hypoleucum* | Ranunculaceae | Angiospermae | Y |
| *Thalictrum omeiense* | Ranunculaceae | Angiospermae | N |
| *Thalictrum petaloideum* | Ranunculaceae | Angiospermae | Y |
| *Thalictrum przewalskii* | Ranunculaceae | Angiospermae | Y |
| *Thalictrum ramosum* | Ranunculaceae | Angiospermae | N |
| *Thalictrum reniforme* | Ranunculaceae | Angiospermae | N |
| *Thalictrum reticulatum* | Ranunculaceae | Angiospermae | N |
| *Thalictrum simplex var. brevipes* | Ranunculaceae | Angiospermae | Y |
| *Thalictrum smithii* | Ranunculaceae | Angiospermae | N |
| *Thalictrum squamiferum* | Ranunculaceae | Angiospermae | N |
| *Thalictrum trichopus* | Ranunculaceae | Angiospermae | N |
| *Thalictrum virgatum* | Ranunculaceae | Angiospermae | N |
| *Trollius asiaticus* | Ranunculaceae | Angiospermae | Y |
| *Trollius buddae* | Ranunculaceae | Angiospermae | N |
| *Trollius chinensis* | Ranunculaceae | Angiospermae | Y |
| *Trollius farreri* | Ranunculaceae | Angiospermae | N |
| *Trollius ledebouri* | Ranunculaceae | Angiospermae | N |
| *Trollius ranunculoides* | Ranunculaceae | Angiospermae | N |
| *Urophysa henryi* | Ranunculaceae | Angiospermae | N |
| *Berchemia floribunda* | Rhamnaceae | Angiospermae | N |
| *Berchemia kulingensis* | Rhamnaceae | Angiospermae | N |
| *Berchemia lineata* | Rhamnaceae | Angiospermae | N |
| *Berchemia polyphylla* | Rhamnaceae | Angiospermae | Y |
| *Berchemia polyphylla var. leioclada* | Rhamnaceae | Angiospermae | Y |
| *Hovenia acerba* | Rhamnaceae | Angiospermae | Y |
| *Paliurus ramosissimus* | Rhamnaceae | Angiospermae | N |
| *Rhamnella franguloides* | Rhamnaceae | Angiospermae | Y |
| *Rhamnus cathartica* | Rhamnaceae | Angiospermae | Y |
| *Rhamnus crenata* | Rhamnaceae | Angiospermae | N |
| *Rhamnus davurica* | Rhamnaceae | Angiospermae | Y |
| *Rhamnus esquirolii var. glabrata* | Rhamnaceae | Angiospermae | N |
| *Rhamnus frangula* | Rhamnaceae | Angiospermae | Y |
| *Rhamnus globosa* | Rhamnaceae | Angiospermae | Y |
| *Rhamnus leptophylla* | Rhamnaceae | Angiospermae | N |
| *Rhamnus ussuriensis* | Rhamnaceae | Angiospermae | Y |
| *Sageretia henryi* | Rhamnaceae | Angiospermae | N |
| *Sageretia thea* | Rhamnaceae | Angiospermae | Y |
| *Ventilago leiocarpa* | Rhamnaceae | Angiospermae | N |
| *Ziziphus jujuba* | Rhamnaceae | Angiospermae | Y |
| *Ziziphus jujuba var. spinosa* | Rhamnaceae | Angiospermae | Y |
| *Ziziphus mauritiana* | Rhamnaceae | Angiospermae | Y |

| | | | |
|---|---|---|---|
| *Ceriops tagal* | Rhizophoracea | Angiospermae | Y |
| *Rhizophora mucronata* | Rhizophoracea | Angiospermae | Y |
| *Agrimonia pilosa var. nepalensis* | Rosaceae | Angiospermae | Y |
| *Amelanchier sinica* | Rosaceae | Angiospermae | N |
| *Amygdalus communis* | Rosaceae | Angiospermae | Y |
| *Amygdalus mongolica* | Rosaceae | Angiospermae | Y |
| *Amygdalus nana* | Rosaceae | Angiospermae | N |
| *Amygdalus pedunculata* | Rosaceae | Angiospermae | Y |
| *Amygdalus persica* | Rosaceae | Angiospermae | Y |
| *Armeniaca mandshurica* | Rosaceae | Angiospermae | N |
| *Armeniaca mume* | Rosaceae | Angiospermae | Y |
| *Armeniaca sibirica* | Rosaceae | Angiospermae | Y |
| *Armeniaca vulgaris* | Rosaceae | Angiospermae | Y |
| *Armeniaca vulgaris var. ansu* | Rosaceae | Angiospermae | Y |
| *Cerasus dictyoneura* | Rosaceae | Angiospermae | N |
| *Cerasus humilis* | Rosaceae | Angiospermae | Y |
| *Cerasus japonica* | Rosaceae | Angiospermae | N |
| *Cerasus tomentosa* | Rosaceae | Angiospermae | N |
| *Chaenomeles cathayensis* | Rosaceae | Angiospermae | N |
| *Chaenomeles sinensis* | Rosaceae | Angiospermae | Y |
| *Chaenomeles speciosa* | Rosaceae | Angiospermae | Y |
| *Chamaerhodos erecta* | Rosaceae | Angiospermae | Y |
| *Coluria longifolia* | Rosaceae | Angiospermae | N |
| *Cotoneaster multiflorus* | Rosaceae | Angiospermae | Y |
| *Crataegus cuneata* | Rosaceae | Angiospermae | Y |
| *Crataegus pinnatifida* | Rosaceae | Angiospermae | Y |
| *Crataegus scabrifolia* | Rosaceae | Angiospermae | N |
| *Crataegus tang-chungchangii* | Rosaceae | Angiospermae | N |
| *Cydonia oblonga* | Rosaceae | Angiospermae | Y |
| *Duchesnea chrysantha* | Rosaceae | Angiospermae | Y |
| *Duchesnea indica* | Rosaceae | Angiospermae | Y |
| *Eriobotrya deflexa* | Rosaceae | Angiospermae | N |
| *Eriobotrya japonica* | Rosaceae | Angiospermae | Y |
| *Geum aleppicum* | Rosaceae | Angiospermae | Y |
| *Kerria japonica* | Rosaceae | Angiospermae | Y |
| *Kerria japonica f. pleniflora* | Rosaceae | Angiospermae | Y |
| *Photinia crassifolia* | Rosaceae | Angiospermae | N |
| *Photinia glabra* | Rosaceae | Angiospermae | Y |
| *Photinia kwangsiensis* | Rosaceae | Angiospermae | N |
| *Photinia parvifolia* | Rosaceae | Angiospermae | Y |
| *Photinia serrulata var. serrulata* | Rosaceae | Angiospermae | Y |
| *Photinia villosa* | Rosaceae | Angiospermae | Y |
| *Potentilla anserina* | Rosaceae | Angiospermae | Y |
| *Potentilla bifurca* | Rosaceae | Angiospermae | Y |
| *Potentilla chinensis* | Rosaceae | Angiospermae | Y |
| *Potentilla conferta* | Rosaceae | Angiospermae | N |
| *Potentilla discolor* | Rosaceae | Angiospermae | Y |
| *Potentilla freyniana* | Rosaceae | Angiospermae | N |
| *Potentilla fruticosa* | Rosaceae | Angiospermae | Y |
| *Potentilla fulgens* | Rosaceae | Angiospermae | N |

| | | | |
|---|---|---|---|
| *Potentilla glabra* | Rosaceae | Angiospermae | N |
| *Potentilla granulosa* | Rosaceae | Angiospermae | N |
| *Potentilla griffithii var. velutina* | Rosaceae | Angiospermae | Y |
| *Potentilla kleiniana* | Rosaceae | Angiospermae | Y |
| *Potentilla leuconota* | Rosaceae | Angiospermae | N |
| *Potentilla longifolia* | Rosaceae | Angiospermae | N |
| *Potentilla multifida* | Rosaceae | Angiospermae | N |
| *Potentilla reptans var. sericophylla* | Rosaceae | Angiospermae | Y |
| *Potentilla tanacetifolia* | Rosaceae | Angiospermae | Y |
| *Potentilla yokusaiana* | Rosaceae | Angiospermae | N |
| *Prinsepia uniflora* | Rosaceae | Angiospermae | N |
| *Prinsepia utilis* | Rosaceae | Angiospermae | Y |
| *Pyrus betulifolia* | Rosaceae | Angiospermae | Y |
| *Pyrus ussuriensis* | Rosaceae | Angiospermae | Y |
| *Rhodotypos scandens* | Rosaceae | Angiospermae | Y |
| *Rosa banksiae var. normalis* | Rosaceae | Angiospermae | Y |
| *Rosa bella* | Rosaceae | Angiospermae | N |
| *Rosa bracteata* | Rosaceae | Angiospermae | N |
| *Rosa chinensis* | Rosaceae | Angiospermae | Y |
| *Rosa davurica* | Rosaceae | Angiospermae | Y |
| *Rosa davurica var. glabra* | Rosaceae | Angiospermae | Y |
| *Rosa laevigata* | Rosaceae | Angiospermae | Y |
| *Rosa macrophylla* | Rosaceae | Angiospermae | N |
| *Rosa maximowicziana* | Rosaceae | Angiospermae | Y |
| *Rosa multiflora var. cathayensis* | Rosaceae | Angiospermae | Y |
| *Rosa omeiensis* | Rosaceae | Angiospermae | N |
| *Rosa roxburghii* | Rosaceae | Angiospermae | Y |
| *Rosa rugosa* | Rosaceae | Angiospermae | Y |
| *Rubus alceaefolius* | Rosaceae | Angiospermae | N |
| *Rubus amphidasys* | Rosaceae | Angiospermae | N |
| *Rubus buergeri* | Rosaceae | Angiospermae | N |
| *Rubus chingii* | Rosaceae | Angiospermae | N |
| *Rubus cochinchinensis* | Rosaceae | Angiospermae | N |
| *Rubus corchorifolius* | Rosaceae | Angiospermae | Y |
| *Rubus coreanus var. coreanus* | Rosaceae | Angiospermae | Y |
| *Rubus crataegifolius* | Rosaceae | Angiospermae | Y |
| *Rubus delavayi* | Rosaceae | Angiospermae | N |
| *Rubus doyonensis* | Rosaceae | Angiospermae | N |
| *Rubus ellipticus var. obcordatus* | Rosaceae | Angiospermae | Y |
| *Rubus eucalyptus* | Rosaceae | Angiospermae | N |
| *Rubus feddei* | Rosaceae | Angiospermae | N |
| *Rubus hastifolius* | Rosaceae | Angiospermae | N |
| *Rubus hirsutus* | Rosaceae | Angiospermae | N |
| *Rubus ichangensis* | Rosaceae | Angiospermae | N |
| *Rubus idaeus* | Rosaceae | Angiospermae | Y |
| *Rubus innominatus* | Rosaceae | Angiospermae | N |
| *Rubus irenaeus var. irenaeus* | Rosaceae | Angiospermae | N |
| *Rubus lambertianus* | Rosaceae | Angiospermae | Y |
| *Rubus leucanthus* | Rosaceae | Angiospermae | N |
| *Rubus multibracteatus* | Rosaceae | Angiospermae | N |

| | | | |
|---|---|---|---|
| *Rubus parvifolius* | Rosaceae | Angiospermae | Y |
| *Rubus pectinellus* | Rosaceae | Angiospermae | N |
| *Rubus peltatus* | Rosaceae | Angiospermae | Y |
| *Rubus phoenicolasius* | Rosaceae | Angiospermae | Y |
| *Rubus pinfaensis* | Rosaceae | Angiospermae | N |
| *Rubus pirifolius* | Rosaceae | Angiospermae | N |
| *Rubus pungens* | Rosaceae | Angiospermae | Y |
| *Rubus reflexus* | Rosaceae | Angiospermae | N |
| *Rubus rosaefolius* | Rosaceae | Angiospermae | N |
| *Rubus setchuenensis* | Rosaceae | Angiospermae | N |
| *Rubus sumatranus* | Rosaceae | Angiospermae | N |
| *Rubus tephrodes* | Rosaceae | Angiospermae | N |
| *Rubus trianthus* | Rosaceae | Angiospermae | Y |
| *Rubus xanthocarpus* | Rosaceae | Angiospermae | N |
| *Sanguisorba filiformis* | Rosaceae | Angiospermae | N |
| *Sanguisorba officinalis* | Rosaceae | Angiospermae | Y |
| *Sanguisorba officinalis var. longifoli* | Rosaceae | Angiospermae | Y |
| *Sanguisorba sitchensis* | Rosaceae | Angiospermae | N |
| *Sibbaldia procumbens var. aphanope* | Rosaceae | Angiospermae | Y |
| *Sorbus pohuashanensis* | Rosaceae | Angiospermae | Y |
| *Spenceria ramalana* | Rosaceae | Angiospermae | N |
| *Spiraea blumei* | Rosaceae | Angiospermae | Y |
| *Spiraea cantoniensis* | Rosaceae | Angiospermae | N |
| *Pyrus lindleyi* | Rosaceae | Angiospermae | N |
| *Rubus otophorus* | Rosaceae | Angiospermae | N |
| *Rubus rolfei* | Rosaceae | Angiospermae | N |
| *Adina pilulifera* | Rubiaceae | Angiospermae | N |
| *Adina rubella* | Rubiaceae | Angiospermae | N |
| *Canthium horridum* | Rubiaceae | Angiospermae | N |
| *Catunaregam spinosa* | Rubiaceae | Angiospermae | Y |
| *Cephalanthus tetrandrus* | *Rubiaceae* | *Angiospermae* | N |
| *Cinchona ledgeriana* | *Rubiaceae* | *Angiospermae* | Y |
| *Cinchona officinalis* | *Rubiaceae* | *Angiospermae* | N |
| *Cinchona succirubra* | *Rubiaceae* | *Angiospermae* | N |
| *Coptosapelta diffusa* | Rubiaceae | Angiospermae | N |
| *Damnacanthus giganteus* | Rubiaceae | Angiospermae | N |
| *Damnacanthus indicus* | Rubiaceae | Angiospermae | N |
| *Diplospora dubia* | Rubiaceae | Angiospermae | N |
| *Galium aparine* | Rubiaceae | Angiospermae | Y |
| *Galium bungei* | Rubiaceae | Angiospermae | Y |
| *Galium verum* | Rubiaceae | Angiospermae | Y |
| *Gardenia jasminoides* | Rubiaceae | Angiospermae | Y |
| *Gardenia stenophylla* | Rubiaceae | Angiospermae | N |
| *Hedyotis acutangula* | Rubiaceae | Angiospermae | N |
| *Hedyotis ampliflora* | Rubiaceae | Angiospermae | N |
| *Hedyotis auriclaria var. auricularia* | Rubiaceae | Angiospermae | N |
| *Hedyotis auricularia* | Rubiaceae | Angiospermae | Y |
| *Hedyotis corymbosa var. corymbosa* | Rubiaceae | Angiospermae | N |
| *Hedyotis diffusa* | Rubiaceae | Angiospermae | N |
| *Hedyotis hedyotidea* | Rubiaceae | Angiospermae | N |

| | | | |
|---|---|---|---|
| *Hedyotis pinifolia* | Rubiaceae | Angiospermae | N |
| *Hedyotis verticillata* | Rubiaceae | Angiospermae | N |
| *Hymenodictyon orixense* | Rubiaceae | Angiospermae | Y |
| *Knoxia corymbosa* | Rubiaceae | Angiospermae | N |
| *Knoxia valerianoides* | Rubiaceae | Angiospermae | N |
| *Morinda citrifolia* | Rubiaceae | Angiospermae | Y |
| *Morinda citrina var. chlorina* | Rubiaceae | Angiospermae | N |
| *Morinda officinalis* | Rubiaceae | Angiospermae | N |
| *Morinda parvifolia* | Rubiaceae | Angiospermae | N |
| *Morinda pubiofficinalis* | Rubiaceae | Angiospermae | N |
| *Morinda scabrifolia* | Rubiaceae | Angiospermae | N |
| *Morinda shuanghuaensis* | Rubiaceae | Angiospermae | N |
| *Mussaenda erosa* | Rubiaceae | Angiospermae | N |
| *Mussaenda pubescens* | Rubiaceae | Angiospermae | Y |
| *Nauclea officinalis* | Rubiaceae | Angiospermae | N |
| *Neohymenopogon oligocarpus* | Rubiaceae | Angiospermae | N |
| *Neohymenopogon parasiticus* | Rubiaceae | Angiospermae | N |
| *Ophiorrhiza calcarata* | Rubiaceae | Angiospermae | N |
| *Pavetta hongkongensis* | Rubiaceae | Angiospermae | Y |
| *Psychotria calocarpa* | Rubiaceae | Angiospermae | Y |
| *Psychotria prainii* | Rubiaceae | Angiospermae | Y |
| *Psychotria rubra* | Rubiaceae | Angiospermae | N |
| *Psychotria serpens* | Rubiaceae | Angiospermae | Y |
| *Rubia manjith* | Rubiaceae | Angiospermae | N |
| *Rubia oncotricha* | Rubiaceae | Angiospermae | N |
| *Schizomussaenda dehiscens* | Rubiaceae | Angiospermae | N |
| *Tarenna attenuata* | Rubiaceae | Angiospermae | N |
| *Tarenna depauperata* | Rubiaceae | Angiospermae | N |
| *Tarenna mollissima* | Rubiaceae | Angiospermae | Y |
| *Uncaria macrophylla* | Rubiaceae | Angiospermae | N |
| *Uncaria rhynchophylla* | Rubiaceae | Angiospermae | N |
| *Uncaria yunnanensis* | Rubiaceae | Angiospermae | N |
| *Wendlandia uvariifolia* | Rubiaceae | Angiospermae | Y |
| *Luculia intermedia* | Rubiaceae | Angiospermae | N |
| *Luculia intermedia var. pinciana* | Rubiaceae | Angiospermae | N |
| *Aegle marmelos* | Rutaceae | Angiospermae | Y |
| *Boenninghausenia albiflora* | Rutaceae | Angiospermae | Y |
| *Boenninghausenia sessilicarpa* | Rutaceae | Angiospermae | Y |
| *Citrus aurantium* | Rutaceae | Angiospermae | Y |
| *Citrus medica var. sarcodactylis* | Rutaceae | Angiospermae | Y |
| *Clausena anisum-olens* | Rutaceae | Angiospermae | N |
| *Clausena emarginata* | Rutaceae | Angiospermae | N |
| *Clausena excavata* | Rutaceae | Angiospermae | N |
| *Evodia lepta* | Rutaceae | Angiospermae | Y |
| *Evodia rutaecarpa* | Rutaceae | Angiospermae | N |
| *Evodia trichotoma* | Rutaceae | Angiospermae | N |
| *Fortunella hindsii* | Rutaceae | Angiospermae | Y |
| *Glycosmis parviflora* | Rutaceae | Angiospermae | N |
| *Micromelum falcatum* | Rutaceae | Angiospermae | N |
| *Micromelum integerrimum* | Rutaceae | Angiospermae | Y |

| | | | |
|---|---|---|---|
| *Murraya kwangsiensis var. kwangsie* | Rutaceae | Angiospermae | N |
| *Orixa japonica* | Rutaceae | Angiospermae | Y |
| *Phellodendron amurense* | Rutaceae | Angiospermae | Y |
| *Psilopeganum sinense* | Rutaceae | Angiospermae | N |
| *Ptelea trifoliata* | Rutaceae | Angiospermae | Y |
| *Ruta graveolens* | Rutaceae | Angiospermae | Y |
| *Skimmia reevesiana* | Rutaceae | Angiospermae | N |
| *Zanthoxylum acanthopodium var. tin* | Rutaceae | Angiospermae | N |
| *Zanthoxylum ailanthoides var. ailant* | Rutaceae | Angiospermae | Y |
| *Zanthoxylum armatum var. armatum* | Rutaceae | Angiospermae | Y |
| *Zanthoxylum austrosinense var. aust* | Rutaceae | Angiospermae | N |
| *Zanthoxylum dissitum var. dissitum* | Rutaceae | Angiospermae | N |
| *Zanthoxylum echinocarpum var. echi* | Rutaceae | Angiospermae | Y |
| *Zanthoxylum laetum* | Rutaceae | Angiospermae | N |
| *Zanthoxylum myriacanthum* | Rutaceae | Angiospermae | N |
| *Zanthoxylum ovalifolium var. ovalifo* | Rutaceae | Angiospermae | N |
| *Meliosma cuneifolia* | Sabiaceae | Angiospermae | N |
| *Meliosma fordii* | Sabiaceae | Angiospermae | N |
| *Sabia japonica* | Sabiaceae | Angiospermae | Y |
| *Sabia schumanniana* | Sabiaceae | Angiospermae | N |
| *Populus × canadensis* | Salicaceae | Angiospermae | Y |
| *Populus davidiana* | Salicaceae | Angiospermae | Y |
| *Populus nigra* | Salicaceae | Angiospermae | Y |
| *Populus tomentosa* | Salicaceae | Angiospermae | Y |
| *Salix matsudana* | Salicaceae | Angiospermae | Y |
| *Salix wallichiana* | Salicaceae | Angiospermae | N |
| *Buckleya lanceolate* | Santalaceae | Angiospermae | N |
| *Dendrotrophe frutescens* | Santalaceae | Angiospermae | N |
| *Osyris wightiana* | Santalaceae | Angiospermae | N |
| *Pyrularia edulis* | Santalaceae | Angiospermae | N |
| *Santalum album* | Santalaceae | Angiospermae | Y |
| *Thesium chinense var. chinense* | Santalaceae | Angiospermae | Y |
| *Allophylus viridis* | Sapindaceae | Angiospermae | N |
| *Cardiospermum halicacabum* | Sapindaceae | Angiospermae | Y |
| *Dimocarpus longan* | Sapindaceae | Angiospermae | Y |
| *Dimocarpus longan var. obtusus* | Sapindaceae | Angiospermae | Y |
| *Erioglossum rubiginosum* | Sapindaceae | Angiospermae | N |
| *Koelreuteria bipinnata* | Sapindaceae | Angiospermae | Y |
| *Koelreuteria paniculata* | Sapindaceae | Angiospermae | Y |
| *Nephelium chryseum* | Sapindaceae | Angiospermae | Y |
| *Sapindus delavayi* | Sapindaceae | Angiospermae | N |
| *Sapindus mukorossi* | Sapindaceae | Angiospermae | Y |
| *Sapindus rarak* | Sapindaceae | Angiospermae | N |
| *Melicoccus bijugatus* | Sapindaceae | Angiospermae | N |
| *Chrysophyllum lanceolatum var. stel* | Sapotaceae | Angiospermae | Y |
| *Manilkara hexandra* | Sapotaceae | Angiospermae | Y |
| *Manilkara zapota* | Sapotaceae | Angiospermae | Y |
| *Planchonella obovata* | Sapotaceae | Angiospermae | Y |
| *Pouteria annamensis* | Sapotaceae | Angiospermae | N |
| *Gymnotheca chinensis* | Saururaceae | Angiospermae | N |

| | | | |
|---|---|---|---|
| *Houttuynia cordata* | Saururaceae | Angiospermae | Y |
| *Saururus chinensis* | Saururaceae | Angiospermae | N |
| *Astilbe chinensis* | Saxifragaceae | Angiospermae | N |
| *Astilbe grandis* | Saxifragaceae | Angiospermae | N |
| *Astilbe rivularis* | Saxifragaceae | Angiospermae | N |
| *Astilbe rivularis var. myriantha* | Saxifragaceae | Angiospermae | N |
| *Bergenia purpurascens* | Saxifragaceae | Angiospermae | N |
| *Bergenia scopulosa* | Saxifragaceae | Angiospermae | N |
| *Chrysosplenium axillare* | Saxifragaceae | Angiospermae | N |
| *Chrysosplenium macrophyllum* | Saxifragaceae | Angiospermae | N |
| *Chrysosplenium nudicaule* | Saxifragaceae | Angiospermae | N |
| *Dichroa febrifuga* | Saxifragaceae | Angiospermae | N |
| *Hydrangea anomala* | Saxifragaceae | Angiospermae | N |
| *Hydrangea macrophylla* | Saxifragaceae | Angiospermae | Y |
| *Hydrangea petiolaris* | Saxifragaceae | Angiospermae | N |
| *Penthorum chinense* | Saxifragaceae | Angiospermae | N |
| *Rodgersia sambucifolia* | Saxifragaceae | Angiospermae | N |
| *Saxifraga aculeata* | Saxifragaceae | Angiospermae | N |
| *Saxifraga atrata* | Saxifragaceae | Angiospermae | N |
| *Saxifraga brunonis* | Saxifragaceae | Angiospermae | N |
| *Saxifraga divaricata* | Saxifragaceae | Angiospermae | N |
| *Saxifraga gemmuligera* | Saxifragaceae | Angiospermae | N |
| *Saxifraga melanocentra* | Saxifragaceae | Angiospermae | N |
| *Saxifraga montana* | Saxifragaceae | Angiospermae | N |
| *Saxifraga nangxianensis* | Saxifragaceae | Angiospermae | N |
| *Saxifraga saginoides* | Saxifragaceae | Angiospermae | N |
| *Saxifraga sanguinea* | Saxifragaceae | Angiospermae | N |
| *Saxifraga signatella* | Saxifragaceae | Angiospermae | N |
| *Saxifraga stolonifera* | Saxifragaceae | Angiospermae | N |
| *Saxifraga tangutica* | Saxifragaceae | Angiospermae | N |
| *Saxifraga umbellulata* | Saxifragaceae | Angiospermae | N |
| *Saxifraga umbellulata var. pectinata* | Saxifragaceae | Angiospermae | N |
| *Saxifraga unguiculata* | Saxifragaceae | Angiospermae | N |
| *Tiarella polyphylla* | Saxifragaceae | Angiospermae | Y |
| *Bacopa monnieri* | Scrophulariace | Angiospermae | Y |
| *Centranthera cochinchinensis var. n* | Scrophulariace | Angiospermae | Y |
| *Linaria vulgaris subsp. sinensis* | Scrophulariace | Angiospermae | Y |
| *Lindernia anagallis* | Scrophulariace | Angiospermae | N |
| *Lindernia antipoda* | Scrophulariace | Angiospermae | Y |
| *Lindernia crustacea* | Scrophulariace | Angiospermae | N |
| *Lindernia ruellioides* | Scrophulariace | Angiospermae | N |
| *Lindernia viscosa* | Scrophulariace | Angiospermae | N |
| *Mimulus tenellus var. platyphyllus* | Scrophulariace | Angiospermae | N |
| *Picrorhiza scrophulariiflora* | Scrophulariace | Angiospermae | N |
| *Scrophularia amugensis* | Scrophulariace | Angiospermae | N |
| *Scrophularia buergeriana var. tsingl* | Scrophulariace | Angiospermae | N |
| *Scrophularia macrocarpa* | Scrophulariace | Angiospermae | N |
| *Scrophularia mandshurica* | Scrophulariace | Angiospermae | N |
| *Scrophularia ningpoensis* | Scrophulariace | Angiospermae | Y |
| *Striga asiatica* | Scrophulariace | Angiospermae | Y |

| | | | |
|---|---|---|---|
| *Veronica ciliata subsp. cephaloides* | Scrophulariace | Angiospermae | N |
| *Veronica linariifolia subsp. dilatata* | Scrophulariace | Angiospermae | Y |
| *Veronica peregrina* | Scrophulariace | Angiospermae | Y |
| *Veronicastrum axillare* | Scrophulariace | Angiospermae | Y |
| *Veronicastrum caulopterum* | Scrophulariace | Angiospermae | N |
| *Veronicastrum villosulum* | Scrophulariace | Angiospermae | N |
| *Veronicastrum villosulum var. glabru* | Scrophulariace | Angiospermae | N |
| *Ailanthus altissima* | Simaroubaceae | Angiospermae | Y |
| *Brucea javanica* | Simaroubaceae | Angiospermae | Y |
| *Harrisonia perforata* | Simaroubaceae | Angiospermae | N |
| *Anisodus acutangulus* | Solanaceae | Angiospermae | N |
| *Lycium barbarum* | Solanaceae | Angiospermae | Y |
| *Lycium chinense* | Solanaceae | Angiospermae | Y |
| *Lycium flexicaule* | Solanaceae | Angiospermae | N |
| *Solanum aviculare* | Solanaceae | Angiospermae | N |
| *Solanum cathayanum* | Solanaceae | Angiospermae | N |
| *Solanum dulcamara* | Solanaceae | Angiospermae | Y |
| *Solanum ferox* | Solanaceae | Angiospermae | N |
| *Solanum indicum* | Solanaceae | Angiospermae | Y |
| *Solanum lyratum* | Solanaceae | Angiospermae | Y |
| *Solanum melongena* | Solanaceae | Angiospermae | Y |
| *Solanum melongena var. esculentum* | Solanaceae | Angiospermae | Y |
| *Solanum melongena var. serpentinum* | Solanaceae | Angiospermae | Y |
| *Solanum nigrum* | *Solanaceae* | *Angiospermae* | Y |
| *Solanum photeinocarpum* | *Solanaceae* | *Angiospermae* | Y |
| *Solanum spirale* | *Solanaceae* | *Angiospermae* | N |
| *Solanum surattense* | Solanaceae | Angiospermae | Y |
| *Solanum torvum* | Solanaceae | Angiospermae | Y |
| *Solanum verbascifolium* | Solanaceae | Angiospermae | Y |
| *Sparganium simplex* | Sparganiaceae | Angiospermae | N |
| *Sparganium stoloniferum* | Sparganiaceae | Angiospermae | Y |
| *Stachyurus himalaicus* | Stachyuraceae | Angiospermae | N |
| *Stachyurus obovatus* | Stachyuraceae | Angiospermae | N |
| *Euscaphis japonica* | Staphyleaceae | Angiospermae | Y |
| *Croomia japonica* | Stemonaceae | Angiospermae | Y |
| *Stemona japonica* | Stemonaceae | Angiospermae | Y |
| *Stemona parviflora* | Stemonaceae | Angiospermae | N |
| *Stemona sessilifolia* | Stemonaceae | Angiospermae | N |
| *Stemona tuberosa* | Stemonaceae | Angiospermae | N |
| *Ambroma augusta* | Sterculiaceae | Angiospermae | N |
| *Firmiana platanifolia* | Sterculiaceae | Angiospermae | Y |
| *Helicteres angustifolia* | Sterculiaceae | Angiospermae | N |
| *Helicteres elongata* | Sterculiaceae | Angiospermae | N |
| *Helicteres isora* | Sterculiaceae | Angiospermae | Y |
| *Pterospermum heterophyllum* | Sterculiaceae | Angiospermae | Y |
| *Sterculia villosa* | Sterculiaceae | Angiospermae | N |
| *Symplocos chinensis* | Symplocaceae | Angiospermae | N |
| *Symplocos congesta* | Symplocaceae | Angiospermae | Y |
| *Symplocos glauca* | Symplocaceae | Angiospermae | Y |
| *Symplocos lancifolia* | Symplocaceae | Angiospermae | Y |

| | | | |
|---|---|---|---|
| *Symplocos laurina* | Symplocaceae | Angiospermae | Y |
| *Symplocos paniculata* | Symplocaceae | Angiospermae | Y |
| *Symplocos poilanei* | Symplocaceae | Angiospermae | N |
| *Symplocos racemosa* | Symplocaceae | Angiospermae | Y |
| *Symplocos sumuntia* | Symplocaceae | Angiospermae | Y |
| *Symplocos terminalis* | Symplocaceae | Angiospermae | N |
| *Schizocapsa plantaginea* | Taccaceae | Angiospermae | N |
| *Tacca chantrieri* | Taccaceae | Angiospermae | Y |
| *Tamarix chinensis* | Tamaricaceae | Angiospermae | Y |
| *Eurya japonica* | Theaceae | Angiospermae | Y |
| *Eurya obtusifolia* | Theaceae | Angiospermae | N |
| *Aquilaria sinensis* | Thymelaeaceae | Angiospermae | Y |
| *Aquilaria yunnanensis* | Thymelaeaceae | Angiospermae | N |
| *Daphne feddei* | Thymelaeaceae | Angiospermae | N |
| *Daphne genkwa* | Thymelaeaceae | Angiospermae | N |
| *Daphne giraldii* | Thymelaeaceae | Angiospermae | N |
| *Daphne jinyunensis var. ptilostyla* | Thymelaeaceae | Angiospermae | N |
| *Edgeworthia chrysantha* | Thymelaeaceae | Angiospermae | N |
| *Stellera chamaejasme* | Thymelaeaceae | Angiospermae | Y |
| *Wikstroemia chamaedaphne* | Thymelaeaceae | Angiospermae | N |
| *Wikstroemia indica* | Thymelaeaceae | Angiospermae | N |
| *Wikstroemia micrantha* | Thymelaeaceae | Angiospermae | N |
| *Wikstroemia nutans* | Thymelaeaceae | Angiospermae | Y |
| *Corchorus aestuans* | Tiliaceae | Angiospermae | Y |
| *Microcos paniculata* | Tiliaceae | Angiospermae | Y |
| *Triumfetta rhomboidea* | Tiliaceae | Angiospermae | N |
| *Typha angustifolia* | Typhaceae | Angiospermae | Y |
| *Typha latifolia* | Typhaceae | Angiospermae | Y |
| *Typha laxmannii* | Typhaceae | Angiospermae | N |
| *Typha orientalis* | Typhaceae | Angiospermae | Y |
| *Typha przewalskii* | Typhaceae | Angiospermae | N |
| *Gironniera subaequalis* | Ulmaceae | Angiospermae | Y |
| *Ulmus gaussenii* | Ulmaceae | Angiospermae | Y |
| *Ulmus macrocarpa* | Ulmaceae | Angiospermae | Y |
| *Ulmus parvifolia* | Ulmaceae | Angiospermae | Y |
| *Ulmus pumila* | Ulmaceae | Angiospermae | Y |
| *Ulmus tonkinensis* | Ulmaceae | Angiospermae | N |
| *Zelkova serrata* | Ulmaceae | Angiospermae | Y |
| *Ammi visnaga* | Umbelliferae | Angiospermae | N |
| *Anethum graveolens* | Umbelliferae | Angiospermae | Y |
| *Angelica acutiloba* | Umbelliferae | Angiospermae | N |
| *Angelica anomala* | Umbelliferae | Angiospermae | N |
| *Angelica biserrata* | Umbelliferae | Angiospermae | N |
| *Angelica dahurica* | Umbelliferae | Angiospermae | Y |
| *Angelica laxifoliata* | Umbelliferae | Angiospermae | N |
| *Angelica megaphylla* | Umbelliferae | Angiospermae | N |
| *Angelica morii* | Umbelliferae | Angiospermae | N |
| *Angelica nitida* | Umbelliferae | Angiospermae | N |
| *Angelica sinensis* | Umbelliferae | Angiospermae | Y |
| *Anthriscus sylvestris* | Umbelliferae | Angiospermae | Y |

| | | | |
|---|---|---|---|
| *Bupleurum bicaule* | Umbelliferae | Angiospermae | N |
| *Bupleurum candollei* | Umbelliferae | Angiospermae | N |
| *Bupleurum chaishoui* | Umbelliferae | Angiospermae | N |
| *Bupleurum chinense* | Umbelliferae | Angiospermae | Y |
| *Bupleurum microcephalum* | Umbelliferae | Angiospermae | N |
| *Bupleurum scorzonerifolium* | Umbelliferae | Angiospermae | Y |
| *Bupleurum wenchuanense* | Umbelliferae | Angiospermae | N |
| *Bupleurum yinchowense* | Umbelliferae | Angiospermae | N |
| *Centella asiatica* | Umbelliferae | Angiospermae | Y |
| *Changium smyrnioides* | Umbelliferae | Angiospermae | N |
| *Chuanminshen violaceum* | Umbelliferae | Angiospermae | N |
| *Cnidium monnieri* | Umbelliferae | Angiospermae | N |
| *Conium maculatum* | Umbelliferae | Angiospermae | Y |
| *Coriandrum sativum* | Umbelliferae | Angiospermae | Y |
| *Cryptotaenia japonica* | Umbelliferae | Angiospermae | Y |
| *Cuminum cyminum* | Umbelliferae | Angiospermae | N |
| *Daucus carota* | Umbelliferae | Angiospermae | Y |
| *Eryngium foetidum* | Umbelliferae | Angiospermae | N |
| *Ferula bungeana* | Umbelliferae | Angiospermae | N |
| *Ferula ferulaeoides* | Umbelliferae | Angiospermae | Y |
| *Ferula fukanensis* | Umbelliferae | Angiospermae | Y |
| *Ferula lehmannii* | Umbelliferae | Angiospermae | Y |
| *Ferula olivacea* | Umbelliferae | Angiospermae | N |
| *Ferula sinkiangensis* | Umbelliferae | Angiospermae | Y |
| *Foeniculum vulgare* | Umbelliferae | Angiospermae | Y |
| *Glehnia littoralis* | Umbelliferae | Angiospermae | Y |
| *Heracleum barmanicum* | Umbelliferae | Angiospermae | N |
| *Heracleum bivittatum* | Umbelliferae | Angiospermae | N |
| *Heracleum dissectifolium* | Umbelliferae | Angiospermae | N |
| *Heracleum hemsleyanum* | Umbelliferae | Angiospermae | N |
| *Heracleum scabridum* | Umbelliferae | Angiospermae | N |
| *Heracleum stenopterum* | Umbelliferae | Angiospermae | N |
| *Heracleum tiliifolium* | Umbelliferae | Angiospermae | N |
| *Hydrocotyle chinensis* | Umbelliferae | Angiospermae | N |
| *Hydrocotyle nepalensis* | Umbelliferae | Angiospermae | N |
| *Hydrocotyle sibthorpioides* | Umbelliferae | Angiospermae | Y |
| *Hydrocotyle sibthorpioides var. batr* | Umbelliferae | Angiospermae | Y |
| *Levisticum officinale* | Umbelliferae | Angiospermae | Y |
| *Libanotis buchtormensis* | Umbelliferae | Angiospermae | N |
| *Ligusticum chuanxiong* | Umbelliferae | Angiospermae | Y |
| *Ligusticum jeholense* | Umbelliferae | Angiospermae | N |
| *Ligusticum sinense* | Umbelliferae | Angiospermae | N |
| *Ligusticum tenuissimum* | Umbelliferae | Angiospermae | N |
| *Nothosmyrnium japonicum* | Umbelliferae | Angiospermae | N |
| *Nothosmyrnium japonicum var. sutc* | Umbelliferae | Angiospermae | N |
| *Notopterygium forbesii var. oviform* | Umbelliferae | Angiospermae | N |
| *Oenanthe javanica* | Umbelliferae | Angiospermae | Y |
| *Osmorhiza aristata var. laxa* | Umbelliferae | Angiospermae | N |
| *Ostericum citriodorum* | Umbelliferae | Angiospermae | N |
| *Ostericum grosseserratum* | Umbelliferae | Angiospermae | N |

| | | | |
|---|---|---|---|
| *Ostericum sieboldii* | Umbelliferae | Angiospermae | N |
| *Peucedanum medicum* | Umbelliferae | Angiospermae | N |
| *Peucedanum praeruptorum* | Umbelliferae | Angiospermae | Y |
| *Peucedanum wawrae* | Umbelliferae | Angiospermae | N |
| *Pimpinella thellungiana* | Umbelliferae | Angiospermae | N |
| *Pleurospermum giraldii* | Umbelliferae | Angiospermae | N |
| *Pternopetalum botrychioides* | Umbelliferae | Angiospermae | N |
| *Pternopetalum leptophyllum* | Umbelliferae | Angiospermae | N |
| *Pternopetalum vulgare* | Umbelliferae | Angiospermae | N |
| *Pternopetalum vulgare var. acumina* | Umbelliferae | Angiospermae | N |
| *Pternopetalum vulgare var. strigosu* | Umbelliferae | Angiospermae | N |
| *Sanicula astrantiifolia* | Umbelliferae | Angiospermae | N |
| *Sanicula coerulescens* | Umbelliferae | Angiospermae | N |
| *Sanicula elata* | Umbelliferae | Angiospermae | N |
| *Sanicula lamelligera* | Umbelliferae | Angiospermae | N |
| *Sanicula orthacantha* | Umbelliferae | Angiospermae | N |
| *Sanicula orthacantha var. brevispina* | Umbelliferae | Angiospermae | N |
| *Saposhnikovia divaricata* | Umbelliferae | Angiospermae | Y |
| *Seseli squarrulosum* | Umbelliferae | Angiospermae | N |
| *Seselopsis tianschanicum* | Umbelliferae | Angiospermae | N |
| *Sinodielsia yunnanensis* | Umbelliferae | Angiospermae | N |
| *Sium suave* | Umbelliferae | Angiospermae | N |
| *Torilis japonica* | Umbelliferae | Angiospermae | Y |
| *Vicatia thibetica* | Umbelliferae | Angiospermae | N |
| *Boehmeria clidemioides var. diffusa* | Urticaceae | Angiospermae | N |
| *Boehmeria densiglomerata* | Urticaceae | Angiospermae | N |
| *Boehmeria gracilis* | Urticaceae | Angiospermae | N |
| *Boehmeria longispica* | Urticaceae | Angiospermae | N |
| *Boehmeria macrophylla* | Urticaceae | Angiospermae | N |
| *Boehmeria macrophylla var. scabrel* | Urticaceae | Angiospermae | N |
| *Boehmeria nivea* | Urticaceae | Angiospermae | Y |
| *Boehmeria nivea var. nipononivea* | Urticaceae | Angiospermae | Y |
| *Boehmeria nivea var. tenacissima* | Urticaceae | Angiospermae | Y |
| *Boehmeria penduliflora* | Urticaceae | Angiospermae | N |
| *Boehmeria siamensis* | Urticaceae | Angiospermae | N |
| *Boehmeria tricuspis* | Urticaceae | Angiospermae | N |
| *Chamabainia cuspidata* | Urticaceae | Angiospermae | N |
| *Elatostema brachyodontum* | Urticaceae | Angiospermae | N |
| *Elatostema cyrtandrifolium* | Urticaceae | Angiospermae | N |
| *Elatostema ichangense* | Urticaceae | Angiospermae | N |
| *Elatostema involucratum* | Urticaceae | Angiospermae | N |
| *Elatostema retrohirtum* | Urticaceae | Angiospermae | N |
| *Elatostema schizocephalum* | Urticaceae | Angiospermae | N |
| *Elatostema stewardii* | Urticaceae | Angiospermae | N |
| *Girardinia suborbiculata subsp. trilc* | Urticaceae | Angiospermae | N |
| *Gonostegia hirta* | Urticaceae | Angiospermae | Y |
| *Laportea cuspidata* | Urticaceae | Angiospermae | N |
| *Laportea violacea* | Urticaceae | Angiospermae | N |
| *Nanocnide lobata* | Urticaceae | Angiospermae | N |
| *Oreocnide frutescens* | Urticaceae | Angiospermae | Y |

| | | | |
|---|---|---|---|
| *Oreocnide obovata var. paradoxa* | Urticaceae | Angiospermae | N |
| *Parietaria micrantha* | Urticaceae | Angiospermae | N |
| *Pellionia radicans* | Urticaceae | Angiospermae | N |
| *Pilea cavaleriei* | Urticaceae | Angiospermae | N |
| *Pilea japonica* | Urticaceae | Angiospermae | N |
| *Pilea longicaulis* | Urticaceae | Angiospermae | N |
| *Pilea notata* | Urticaceae | Angiospermae | Y |
| *Pilea peploides var. major* | Urticaceae | Angiospermae | N |
| *Pilea plataniflora* | Urticaceae | Angiospermae | N |
| *Pilea pumila* | Urticaceae | Angiospermae | Y |
| *Pilea swinglei* | Urticaceae | Angiospermae | N |
| *Pilea verrucosa* | Urticaceae | Angiospermae | N |
| *Urtica angustifolia* | Urticaceae | Angiospermae | N |
| *Urtica cannabina* | Urticaceae | Angiospermae | N |
| *Urtica fissa* | Urticaceae | Angiospermae | Y |
| *Urtica laetevirens subsp. laetevirens* | Urticaceae | Angiospermae | N |
| *Urtica triangularis* | Urticaceae | Angiospermae | N |
| *Urtica triangularis subsp. pinnatifid* | Urticaceae | Angiospermae | N |
| *Patrinia heterophylla* | Valerianaceae | Angiospermae | Y |
| *Patrinia monandra* | Valerianaceae | Angiospermae | N |
| *Patrinia punctiflora* | Valerianaceae | Angiospermae | N |
| *Patrinia punctiflora var. robusta* | Valerianaceae | Angiospermae | N |
| *Patrinia scabiosaefolia* | Valerianaceae | Angiospermae | Y |
| *Patrinia villosa* | Valerianaceae | Angiospermae | Y |
| *Valeriana flagellifera* | Valerianaceae | Angiospermae | N |
| *Valeriana jatamansi* | Valerianaceae | Angiospermae | Y |
| *Valeriana officinalis* | Valerianaceae | Angiospermae | Y |
| *Avicennia marina* | Verbenaceae | Angiospermae | Y |
| *Callicarpa arborea* | Verbenaceae | Angiospermae | N |
| *Callicarpa bodinieri* | Verbenaceae | Angiospermae | N |
| *Callicarpa cathayana* | Verbenaceae | Angiospermae | Y |
| *Callicarpa dichotoma* | Verbenaceae | Angiospermae | N |
| *Callicarpa formosana* | Verbenaceae | Angiospermae | N |
| *Callicarpa giraldii* | Verbenaceae | Angiospermae | N |
| *Callicarpa kochiana* | Verbenaceae | Angiospermae | N |
| *Callicarpa longissima* | Verbenaceae | Angiospermae | N |
| *Callicarpa macrophylla* | Verbenaceae | Angiospermae | N |
| *Callicarpa nudiflora* | Verbenaceae | Angiospermae | N |
| *Callicarpa rubella* | Verbenaceae | Angiospermae | N |
| *Callicarpa rubella var. rubella f. cre* | Verbenaceae | Angiospermae | N |
| *Callicarpa yunnanensis* | Verbenaceae | Angiospermae | N |
| *Caryopteris incana* | Verbenaceae | Angiospermae | N |
| *Caryopteris mongholica* | Verbenaceae | Angiospermae | Y |
| *Caryopteris nepetaefolia* | Verbenaceae | Angiospermae | N |
| *Caryopteris paniculata* | Verbenaceae | Angiospermae | N |
| *Caryopteris terniflora* | Verbenaceae | Angiospermae | N |
| *Clerodendrum bungei* | Verbenaceae | Angiospermae | N |
| *Clerodendrum canescens* | Verbenaceae | Angiospermae | N |
| *Clerodendrum cyrtophyllum* | Verbenaceae | Angiospermae | Y |
| *Clerodendrum fortunatum* | Verbenaceae | Angiospermae | N |

| | | | |
|---|---|---|---|
| *Clerodendrum indicum* | Verbenaceae | Angiospermae | N |
| *Clerodendrum inerme* | Verbenaceae | Angiospermae | Y |
| *Clerodendrum japonicum* | Verbenaceae | Angiospermae | Y |
| *Clerodendrum kwangtungense* | Verbenaceae | Angiospermae | N |
| *Clerodendrum lindleyi* | Verbenaceae | Angiospermae | N |
| *Clerodendrum mandarinorum* | Verbenaceae | Angiospermae | N |
| *Clerodendrum philippinum* | Verbenaceae | Angiospermae | N |
| *Clerodendrum philippinum var. simp* | Verbenaceae | Angiospermae | N |
| *Clerodendrum serratum* | Verbenaceae | Angiospermae | N |
| *Clerodendrum serratum var. amplexi* | Verbenaceae | Angiospermae | N |
| *Clerodendrum serratum var. herbace* | Verbenaceae | Angiospermae | N |
| *Clerodendrum serratum var. wallich* | Verbenaceae | Angiospermae | N |
| *Clinacanthus nutans* | Verbenaceae | Angiospermae | N |
| *Duranta repens* | Verbenaceae | Angiospermae | Y |
| *Lantana camara* | Verbenaceae | Angiospermae | Y |
| *Phyla nodiflora* | Verbenaceae | Angiospermae | Y |
| *Premna crassa* | Verbenaceae | Angiospermae | N |
| *Premna henryana* | Verbenaceae | Angiospermae | N |
| *Premna ligustroides* | Verbenaceae | Angiospermae | N |
| *Premna microphylla* | Verbenaceae | Angiospermae | N |
| *Premna puberula* | Verbenaceae | Angiospermae | N |
| *Premna sunyiensis* | Verbenaceae | Angiospermae | N |
| *Premna szemaoensis* | Verbenaceae | Angiospermae | N |
| *Premna urticifolia* | Verbenaceae | Angiospermae | N |
| *Pygmaeopremna herbacea* | Verbenaceae | Angiospermae | N |
| *Sphenodesme pentandra var. pentano* | Verbenaceae | Angiospermae | N |
| *Stachytarpheta jamaicensis* | Verbenaceae | Angiospermae | Y |
| *Tectona grandis* | Verbenaceae | Angiospermae | Y |
| *Verbena officinalis* | Verbenaceae | Angiospermae | Y |
| *Vitex canescens* | Verbenaceae | Angiospermae | N |
| *Vitex negundo* | Verbenaceae | Angiospermae | Y |
| *Vitex negundo var. cannabifolia* | Verbenaceae | Angiospermae | Y |
| *Vitex negundo var. heterophylla* | Verbenaceae | Angiospermae | Y |
| *Vitex peduncularis* | Verbenaceae | Angiospermae | N |
| *Vitex quinata var. puberula* | Verbenaceae | Angiospermae | Y |
| *Vitex trifolia* | Verbenaceae | Angiospermae | Y |
| *Vitex trifolia var. simplicifolia* | Verbenaceae | Angiospermae | Y |
| *Viola acuminata* | Violaceae | Angiospermae | Y |
| *Viola acuminata var. pilifera* | Violaceae | Angiospermae | Y |
| *Viola betonicifolia* | Violaceae | Angiospermae | N |
| *Viola betonicifolia subsp. jaunsarien* | Violaceae | Angiospermae | N |
| *Viola biflora* | Violaceae | Angiospermae | Y |
| *Viola collina* | Violaceae | Angiospermae | Y |
| *Viola delavayi* | Violaceae | Angiospermae | N |
| *Viola diffusa* | Violaceae | Angiospermae | Y |
| *Viola diffusa var. brevibarbata* | Violaceae | Angiospermae | Y |
| *Viola grypoceras* | Violaceae | Angiospermae | Y |
| *Viola inconspicua* | Violaceae | Angiospermae | N |
| *Viola mandshurica* | Violaceae | Angiospermae | Y |
| *Viola moupinensis* | Violaceae | Angiospermae | N |

| | | | |
|---|---|---|---|
| *Viola patrinii* | Violaceae | Angiospermae | Y |
| *Viola philippica* | Violaceae | Angiospermae | Y |
| *Viola prioantha* | Violaceae | Angiospermae | N |
| *Viola prionantha var. prionantha* | Violaceae | Angiospermae | N |
| *Viola rockiana* | Violaceae | Angiospermae | N |
| *Viola verecunda* | Violaceae | Angiospermae | N |
| *Ampelopsis heterophylla var. brevipe* | Vitaceae | Angiospermae | Y |
| *Ampelopsis japonica* | Vitaceae | Angiospermae | N |
| *Cayratia corniculata* | Vitaceae | Angiospermae | N |
| *Cayratia japonica* | Vitaceae | Angiospermae | Y |
| *Parthenocissus tricuspidata* | Vitaceae | Angiospermae | Y |
| *Tetrastigma hemsleyanum* | Vitaceae | Angiospermae | Y |
| *Tetrastigma obtectum* | Vitaceae | Angiospermae | N |
| *Tetrastigma planicaule* | Vitaceae | Angiospermae | Y |
| *Vitis balanseana* | Vitaceae | Angiospermae | N |
| *Vitis davidii* | Vitaceae | Angiospermae | N |
| *Vitis flexuosa* | Vitaceae | Angiospermae | Y |
| *Vitis romanetii Romanet du Caillaud* | Vitaceae | Angiospermae | N |
| *Alpinia blepharocalyx* | Zingiberaceae | Angiospermae | N |
| *Alpinia chinensis* | Zingiberaceae | Angiospermae | Y |
| *Alpinia conchigera* | Zingiberaceae | Angiospermae | Y |
| *Alpinia galanga* | Zingiberaceae | Angiospermae | Y |
| *Alpinia japonica* | Zingiberaceae | Angiospermae | Y |
| *Alpinia nigra* | Zingiberaceae | Angiospermae | Y |
| *Alpinia officinarum* | Zingiberaceae | Angiospermae | Y |
| *Alpinia oxyphylla* | Zingiberaceae | Angiospermae | N |
| *Alpinia psilogyna* | Zingiberaceae | Angiospermae | N |
| *Alpinia stachyoides* | Zingiberaceae | Angiospermae | N |
| *Alpinia zerumbet* | Zingiberaceae | Angiospermae | Y |
| *Amomum aurantiacum* | Zingiberaceae | Angiospermae | N |
| *Amomum compactum* | Zingiberaceae | Angiospermae | N |
| *Amomum kravanh* | Zingiberaceae | Angiospermae | N |
| *Amomum kwangsiense* | Zingiberaceae | Angiospermae | N |
| *Amomum maximum* | Zingiberaceae | Angiospermae | Y |
| *Amomum microcarpum* | Zingiberaceae | Angiospermae | N |
| *Amomum muricarpum* | Zingiberaceae | Angiospermae | N |
| *Amomum subulatum* | Zingiberaceae | Angiospermae | Y |
| *Amomum thyrsoideum* | Zingiberaceae | Angiospermae | N |
| *Amomum tsaoko* | Zingiberaceae | Angiospermae | N |
| *Amomum villosum* | Zingiberaceae | Angiospermae | Y |
| *Amomum villosum var. nanum* | Zingiberaceae | Angiospermae | Y |
| *Amomum villosum var. xanthioides* | Zingiberaceae | Angiospermae | Y |
| *Caulokaempferia coenobialis* | Zingiberaceae | Angiospermae | N |
| *Costus speciosus* | Zingiberaceae | Angiospermae | Y |
| *Costus tonkinensis* | Zingiberaceae | Angiospermae | Y |
| *Curcuma aromatica* | Zingiberaceae | Angiospermae | Y |
| *Curcuma caesia* | Zingiberaceae | Angiospermae | Y |
| *Curcuma elata* | Zingiberaceae | Angiospermae | Y |
| *Curcuma kwangsiensis* | Zingiberaceae | Angiospermae | N |
| *Curcuma longa* | Zingiberaceae | Angiospermae | Y |

| | | | |
|---|---|---|---|
| *Curcuma viridiflora* | Zingiberaceae | Angiospermae | N |
| *Curcuma zedoaria* | Zingiberaceae | Angiospermae | Y |
| *Hedychium coronarium* | Zingiberaceae | Angiospermae | Y |
| *Hedychium spicatum* | Zingiberaceae | Angiospermae | N |
| *Hedychium villosum* | Zingiberaceae | Angiospermae | N |
| *Kaempferia rotunda* | Zingiberaceae | Angiospermae | Y |
| *Stahlianthus involucratus* | Zingiberaceae | Angiospermae | N |
| *Zingiber corallinum* | Zingiberaceae | Angiospermae | N |
| *Zingiber mioga* | Zingiberaceae | Angiospermae | Y |
| *Zingiber officinale* | Zingiberaceae | Angiospermae | Y |
| *Zingiber zerumbet* | Zingiberaceae | Angiospermae | Y |
| *Nitraria sibirica* | Zygophyllacea | Angiospermae | Y |
| *Peganum harmala* | Zygophyllacea | Angiospermae | Y |
| *Peganum nigellastrum* | Zygophyllacea | Angiospermae | Y |
| *Tribulus terrester* | Zygophyllacea | Angiospermae | Y |
| *Agathis dammara* | Araucariaceae | Gymnospermae | Y |
| *Cephalotaxus fortunei* | Cephalotaxace | Gymnospermae | Y |
| *Cephalotaxus hainanensis* | Cephalotaxace | Gymnospermae | Y |
| *Cephalotaxus lanceolata* | Cephalotaxace | Gymnospermae | N |
| *Cephalotaxus mannii* | Cephalotaxace | Gymnospermae | N |
| *Cephalotaxus oliveri* | Cephalotaxace | Gymnospermae | N |
| *Cephalotaxus sinensis* | Cephalotaxace | Gymnospermae | Y |
| *Juniperus communis* | Cupressaceae | Gymnospermae | Y |
| *Juniperus rigida* | Cupressaceae | Gymnospermae | Y |
| *Platycladus orientalis* | Cupressaceae | Gymnospermae | Y |
| *Sabina chinensis* | Cupressaceae | Gymnospermae | Y |
| *Cycas revoluta* | Cycadaceae | Gymnospermae | Y |
| *Ephedra equisetina* | Ephedraceae | Gymnospermae | Y |
| *Ephedra intermedia* | Ephedraceae | Gymnospermae | Y |
| *Ephedra likiangensis* | Ephedraceae | Gymnospermae | N |
| *Ephedra minuta* | Ephedraceae | Gymnospermae | N |
| *Ephedra monosperma* | Ephedraceae | Gymnospermae | Y |
| *Ephedra sinica* | Ephedraceae | Gymnospermae | Y |
| *Ginkgo biloba* | Ginkgoaceae | Gymnospermae | Y |
| *Pinus koraiensis* | Pinaceae | Gymnospermae | Y |
| *Pinus massoniana* | Pinaceae | Gymnospermae | Y |
| *Pinus massoniana var. hainanensis* | Pinaceae | Gymnospermae | Y |
| *Pinus tabulaeformis* | Pinaceae | Gymnospermae | Y |
| *Pseudolarix amabilis* | Pinaceae | Gymnospermae | N |
| *Taxus cuspidata* | Taxaceae | Gymnospermae | Y |
| *Adiantum capillus-veneris f. dissectu* | Adiantaceae | Pteridophyta | Y |
| *Adiantum flabellulatum* | Adiantaceae | Pteridophyta | Y |
| *Adiantum pedatum* | Adiantaceae | Pteridophyta | Y |
| *Angiopteris officinalis* | Angiopteridac | Pteridophyta | N |
| *Angiopteris petiolulata* | Angiopteridac | Pteridophyta | N |
| *Ceterach officinarum* | Aspleniaceae | Pteridophyta | Y |
| *Botrychium officinale* | Botrychiaceae | Pteridophyta | N |
| *Botrychium ternatum* | Botrychiaceae | Pteridophyta | Y |
| *Botrychium virginianum* | Botrychiaceae | Pteridophyta | Y |
| *Davallia mariesii* | Davalliaceae | Pteridophyta | Y |

| | | | |
|---|---|---|---|
| *Humata tyermanni* | Davalliaceae | Pteridophyta | N |
| *Drynaria delavayi* | Drynariaceae | Pteridophyta | N |
| *Drynaria propinqua* | Drynariaceae | Pteridophyta | Y |
| *Drynaria roosii* | Drynariaceae | Pteridophyta | N |
| *Dryopteris crassirhizoma* | Dryopteridacea | Pteridophyta | N |
| *Dryopteris lacera* | Dryopteridacea | Pteridophyta | Y |
| *Vandenboschia naseana* | Hymenophylla | Pteridophyta | N |
| *Stenoloma chusanum* | Lindsaeaceae | Pteridophyta | N |
| *Lygodium japonicum* | Lygodiaceae | Pteridophyta | Y |
| *Marsilea quadrifolia* | Marsileaceae | Pteridophyta | Y |
| *Nephrolepis auriculata* | Nephrolepidac | Pteridophyta | Y |
| *Ophioglossum thermale* | Ophioglossace | Pteridophyta | Y |
| *Ophioglossum vulgatum* | Ophioglossace | Pteridophyta | Y |
| *Ceratopteris pteridoides* | Parkeriaceae | Pteridophyta | N |
| *Ceratopteris thalictroides* | Parkeriaceae | Pteridophyta | Y |
| *Lepidogrammitis diversa* | Polypodiaceae | Pteridophyta | N |
| *Lepidogrammitis drymoglossoides* | Polypodiaceae | Pteridophyta | N |
| *Microsorum fortunei* | Polypodiaceae | Pteridophyta | N |
| *Pyrrosia calvata* | Polypodiaceae | Pteridophyta | N |
| *Pyrrosia lingua* | Polypodiaceae | Pteridophyta | Y |
| *Pyrrosia petiolosa* | Polypodiaceae | Pteridophyta | Y |
| *Pteris ensiformis* | Pteridaceae | Pteridophyta | Y |
| *Pteris ensiformis var. merrilli* | Pteridaceae | Pteridophyta | Y |
| *Pteris multifida* | Pteridaceae | Pteridophyta | Y |
| *Pteridium aquilinum* | Pteridiaceae | Pteridophyta | Y |
| *Pteridium aquilinum var. latiusculun* | Pteridiaceae | Pteridophyta | Y |
| *Salvinia natans* | Salviniaceae | Pteridophyta | Y |
| *Schizaea digitata* | Schizaeaceae | Pteridophyta | N |
| *Onychium japonicum* | Sinopteridacea | Pteridophyta | Y |
| *Onychium japonicum var. lucidum* | Sinopteridacea | Pteridophyta | Y |
| *Pronephrium penangianum* | Thelypteridace | Pteridophyta | N |
| *Pseudocyclosorus falcilobus* | Thelypteridace | Pteridophyta | N |
