# Supplementary material for: A Database on Mycorrhizal Traits of Chinese Medicinal Plants
Source: Front Plant Sci. 2022 Mar 1;13:840343. doi: 10.3389/fpls.2022.840343 (PMC8921535; doi:10.3389/fpls.2022.840343)
Supplement: Supplementary file 2 [file Data_Sheet_2.PDF]

Species	Family	Phylum	Mycorrhizal types
Adhatoda vasica	Acanthaceae	Angiospermae	AM
Andrographis paniculata	Acanthaceae	Angiospermae	AM+NM
Asystasia chelonoides	Acanthaceae	Angiospermae	
Asystasiella neesiana	Acanthaceae	Angiospermae	
Baphicacanthus cusia	Acanthaceae	Angiospermae	
Barleria cristata	Acanthaceae	Angiospermae	AM
Barleria lupulina	Acanthaceae	Angiospermae	AM
Championella sarcorrhiza	Acanthaceae	Angiospermae	
Dicliptera chinensis	Acanthaceae	Angiospermae	
Gendarussa ventricosa	Acanthaceae	Angiospermae	
Gendarussa vulgaris	Acanthaceae	Angiospermae	
Hygrophila salicifolia	Acanthaceae	Angiospermae	AM
Hypoestes purpurea	Acanthaceae	Angiospermae	
Lepidagathis incurva	Acanthaceae	Angiospermae	
Peristrophe japonica	Acanthaceae	Angiospermae	
Rhinacanthus nasutus	Acanthaceae	Angiospermae	AM
Rostellularia procumbens	Acanthaceae	Angiospermae	
Rungia pectinata	Acanthaceae	Angiospermae	
Actinidia polygama	Actinidiaceae	Angiospermae	AM+NM
Saurauia tristyla var. tristyla	Actinidiaceae	Angiospermae	
Mollugo cerviana	Aizoaceae	Angiospermae	AM
Mollugo stricta	Aizoaceae	Angiospermae	NM
Tetragonia tetragonioides	Aizoaceae	Angiospermae	AM+NM
Alangium chinense	Alangiaceae	Angiospermae	AM+NM
Alangium faberi	Alangiaceae	Angiospermae	
Alangium faberi var. platyphyllum	Alangiaceae	Angiospermae	
Alangium platanifolium	Alangiaceae	Angiospermae	AM+ECM
Alangium salviifolium	Alangiaceae	Angiospermae	AM
Alisma canaliculatum	Alismataceae	Angiospermae	AM
Alisma orinentale	Alismataceae	Angiospermae	
Alisma plantago-aquatica	Alismataceae	Angiospermae	AM+NM
Sagittaria pygmaea	Alismataceae	Angiospermae	NM

Achyranthes aspera	Amaranthaceae	Angiospermae	AM+NM
Achyranthes bidentata	Amaranthaceae	Angiospermae	AM+NM
Achyranthes longifolia	Amaranthaceae	Angiospermae	
Achyranthes longifolia f. rubra	Amaranthaceae	Angiospermae	
Achyranthes ogatai	Amaranthaceae	Angiospermae	
Aerva sanguinolenta	Amaranthaceae	Angiospermae	AM
Alternanthera philoxeroides	Amaranthaceae	Angiospermae	AM+NM
Alternanthera sessilis	Amaranthaceae	Angiospermae	AM+NM
Amaranthus caudatus	Amaranthaceae	Angiospermae	AM+NM
Amaranthus lividus	Amaranthaceae	Angiospermae	AM
Amaranthus retroflexus	Amaranthaceae	Angiospermae	AM+NM
Amaranthus spinosus	Amaranthaceae	Angiospermae	AM+NM
Amaranthus tricolor	Amaranthaceae	Angiospermae	AM+NM
Amaranthus viridis	Amaranthaceae	Angiospermae	AM+NM
Celosia argentea	Amaranthaceae	Angiospermae	AM+NM
Celosia cristata	Amaranthaceae	Angiospermae	AM
Celosia swinhoei	Amaranthaceae	Angiospermae	
Cladostachys frutescens	Amaranthaceae	Angiospermae	
Cyathula capitata	Amaranthaceae	Angiospermae	
Cyathula officinalis	Amaranthaceae	Angiospermae	
Cyathula prostrata	Amaranthaceae	Angiospermae	AM
Iresine herbstii	Amaranthaceae	Angiospermae	
Agave americana	Amaryllidaceae	Angiospermae	AM+NM
Agave sisalana	Amaryllidaceae	Angiospermae	AM
Crinum asiaticum	Amaryllidaceae	Angiospermae	AM
Crinum asiaticum var. sinicum	Amaryllidaceae	Angiospermae	AM
Curculigo orchioides	Amaryllidaceae	Angiospermae	AM+NM
Narcissus tazetta var. chinensis	Amaryllidaceae	Angiospermae	AM
Anacardium occidentale	Anacardiaceae	Angiospermae	AM+NM
Choerospondias axillaris var. axillaris	Anacardiaceae	Angiospermae	AM
Dobinea delavayi	Anacardiaceae	Angiospermae	
Dracontomelon duperreanum	Anacardiaceae	Angiospermae	
Mangifera indica	Anacardiaceae	Angiospermae	AM+NM

Pistacia vera	Anacardiaceae	Angiospermae	AM
Pistacia weinmannifolia	Anacardiaceae	Angiospermae	AM
Rhus chinensis	Anacardiaceae	Angiospermae	AM+ECM
Toxicodendron succedaneum	Anacardiaceae	Angiospermae	AM
Toxicodendron vernicifluum	Anacardiaceae	Angiospermae	
Annona squamosa	Annonaceae	Angiospermae	AM
Artabotrys hexapetalus	Annonaceae	Angiospermae	AM+NM
Desmos chinensis	Annonaceae	Angiospermae	AM
Fissistigma bracteolatum	Annonaceae	Angiospermae	
Fissistigma glaucescens	Annonaceae	Angiospermae	NM
Fissistigma oldhamii	Annonaceae	Angiospermae	
Fissistigma polyanthum	Annonaceae	Angiospermae	
Fissistigma uonicum	Annonaceae	Angiospermae	
Goniothalamus donnaiensis	Annonaceae	Angiospermae	
Uvaria microcarpa	Annonaceae	Angiospermae	
Uvaria tonkinensis	Annonaceae	Angiospermae	
Angelica decusiva	Apiaceae	Angiospermae	
Aganosma kwangsiensis	Apocynaceae	Angiospermae	
Aganosma schlechteriana	Apocynaceae	Angiospermae	
Alstonia mairei	Apocynaceae	Angiospermae	
Alstonia scholaris	Apocynaceae	Angiospermae	AM
Alstonia yunnanensis	Apocynaceae	Angiospermae	
Alyxia levinei	Apocynaceae	Angiospermae	
Alyxia sinensis	Apocynaceae	Angiospermae	
Amsonia sinensis	Apocynaceae	Angiospermae	
Apocynum venetum	Apocynaceae	Angiospermae	AM+ECM
Apocynum venetum var. ellipticifolium	Apocynaceae	Angiospermae	AM+ECM
Beaumontia grandiflora	Apocynaceae	Angiospermae	
Catharanthus roseus	Apocynaceae	Angiospermae	AM+NM
Cerbera manghas	Apocynaceae	Angiospermae	AM
Chonemorpha eriostylis	Apocynaceae	Angiospermae	
Chonemorpha megacalyx	Apocynaceae	Angiospermae	
Chonemorpha valvata	Apocynaceae	Angiospermae	

Ecdysanthera rosea	Apocynaceae	Angiospermae	AM
Ecdysanthera utilis	Apocynaceae	Angiospermae	
Ervatamia hainanensis	Apocynaceae	Angiospermae	
Ervatamia kwangsiensis	Apocynaceae	Angiospermae	
Ervatamia officinalis	Apocynaceae	Angiospermae	
Holarrhena antidysenterica	Apocynaceae	Angiospermae	
Ichnocarpus frutescens	Apocynaceae	Angiospermae	AM+NM
Ichnocarpus oliganthus	Apocynaceae	Angiospermae	
Kopsia officinalis	Apocynaceae	Angiospermae	
Melodinus fusiformis	Apocynaceae	Angiospermae	AM
Melodinus henryi	Apocynaceae	Angiospermae	
Melodinus suaveolens	Apocynaceae	Angiospermae	
Nerium oleander	Apocynaceae	Angiospermae	AM+ECM+NM
Parabarium huaitingii	Apocynaceae	Angiospermae	
Parabarium micranthum	Apocynaceae	Angiospermae	
Pottsia laxiflora	Apocynaceae	Angiospermae	
Rauvolfia latifrons	Apocynaceae	Angiospermae	
Rauvolfia serpentina	Apocynaceae	Angiospermae	AM+NM
Rauvolfia tetraphylla	Apocynaceae	Angiospermae	AM
Rauvolfia verticillata	Apocynaceae	Angiospermae	AM
Rauvolfia verticillata var. hainanensis	Apocynaceae	Angiospermae	AM
Rauvolfia vomitoria	Apocynaceae	Angiospermae	AM
Rauvolfia yunnanensis	Apocynaceae	Angiospermae	AM
Sindechites henryi	Apocynaceae	Angiospermae	
Strophanthus divaricatus	Apocynaceae	Angiospermae	
Strophanthus gratus	Apocynaceae	Angiospermae	
Strophanthus hispidus	Apocynaceae	Angiospermae	
Strophanthus sarmentosus	Apocynaceae	Angiospermae	
Trachelospermum dunnii	Apocynaceae	Angiospermae	
Trachelospermum jasminoides	Apocynaceae	Angiospermae	AM
Trachelospermum jasminoides var. heterophyllum	Apocynaceae	Angiospermae	AM
Vallaris indecora	Apocynaceae	Angiospermae	
Wrightia laevis	Apocynaceae	Angiospermae	

Wrightia pubescens	Apocynaceae	Angiospermae	AM
Tylophora koi Merr.	Apocynaceae	Angiospermae	
Tylophora ovata (Lindl.) Hook. ex Steud.	Apocynaceae	Angiospermae	
Wrightia annamensis	Apocynaceae	Angiospermae	
Ilex aculeolata	Aquifoliaceae	Angiospermae	
Ilex asprella	Aquifoliaceae	Angiospermae	AM
Ilex cornuta	Aquifoliaceae	Angiospermae	AM
Ilex latifolia	Aquifoliaceae	Angiospermae	AM
Ilex macrocarpa	Aquifoliaceae	Angiospermae	
Ilex macrocarpa var. longipedunculata	Aquifoliaceae	Angiospermae	
Ilex microcca f. pilosa	Aquifoliaceae	Angiospermae	AM+NM
Ilex pernyi	Aquifoliaceae	Angiospermae	
Ilex rotunda	Aquifoliaceae	Angiospermae	
Acorus calamus var. verus	Araceae	Angiospermae	AM+NM
Acorus gramineus	Araceae	Angiospermae	NM
Acorus rumphianus	Araceae	Angiospermae	
Acorus tatarinowii	Araceae	Angiospermae	AM
Aglaonema modestum	Araceae	Angiospermae	AM
Aglaonema tenuipes	Araceae	Angiospermae	AM+NM
Alocasia cucullata	Araceae	Angiospermae	
Alocasia macrorrhiza	Araceae	Angiospermae	AM+NM
Amorphophallus mairei	Araceae	Angiospermae	
Amorphophallus rivieri	Araceae	Angiospermae	ECM
Amorphophallus sinensis	Araceae	Angiospermae	
Amydrium sinense	Araceae	Angiospermae	
Arisaema amurense var. serratum	Araceae	Angiospermae	
Arisaema angustatum	Araceae	Angiospermae	
Arisaema angustatum var. peninsulae	Araceae	Angiospermae	
Arisaema asperatum	Araceae	Angiospermae	
Arisaema austro-yunnanense	Araceae	Angiospermae	
Arisaema bathycoleum	Araceae	Angiospermae	
Arisaema calcareum	Araceae	Angiospermae	
Arisaema decipiens	Araceae	Angiospermae	

Arisaema du-bois-reymondiae	Araceae	Angiospermae	
Arisaema elephas	Araceae	Angiospermae	
Arisaema erubescens	Araceae	Angiospermae	AM
Arisaema fargesii	Araceae	Angiospermae	
Arisaema flavum	Araceae	Angiospermae	AM
Arisaema franchetianum	Araceae	Angiospermae	
Arisaema heterophyllum	Araceae	Angiospermae	
Arisaema inkiangense var. maculatum	Araceae	Angiospermae	
Arisaema lichiangense	Araceae	Angiospermae	
Arisaema lingyunense	Araceae	Angiospermae	
Arisaema lobatum	Araceae	Angiospermae	
Arisaema nepenthoides	Araceae	Angiospermae	
Arisaema prazeri	Araceae	Angiospermae	
Arisaema rhizomatum var. nudum	Araceae	Angiospermae	
Arisaema rhombiforme	Araceae	Angiospermae	
Arisaema sikokianum var. henryanum	Araceae	Angiospermae	
Arisaema yunnanense	Araceae	Angiospermae	
Caladium bicolor	Araceae	Angiospermae	AM
Colocasia antiquorum	Araceae	Angiospermae	NM
Colocasia esculenta	Araceae	Angiospermae	AM+NM
Colocasia gigantea	Araceae	Angiospermae	
Cryptocoryne yunnanensis	Araceae	Angiospermae	
Epipremnum pinnatum	Araceae	Angiospermae	
Gonatanthus pumilus	Araceae	Angiospermae	
Homalomena occulta	Araceae	Angiospermae	
Lasia spinosa	Araceae	Angiospermae	NM
Pinellia cordata	Araceae	Angiospermae	
Pinellia integrifolia	Araceae	Angiospermae	
Pinellia pedatisecta	Araceae	Angiospermae	
Pinellia ternata	Araceae	Angiospermae	AM
Pistia stratiotes	Araceae	Angiospermae	NM
Pothos chinensis var. lotienensis	Araceae	Angiospermae	
Pothos kerrii	Araceae	Angiospermae	

Pothos pilulifer	Araceae	Angiospermae	
Pothos repens	Araceae	Angiospermae	
Pothos scandens	Araceae	Angiospermae	
Remusatia vivipara	Araceae	Angiospermae	
Rhaphidophora decursiva	Araceae	Angiospermae	AM
Rhaphidophora hongkongensis	Araceae	Angiospermae	AM
Rhaphidophora hookeri	Araceae	Angiospermae	
Rhaphidophora peepla	Araceae	Angiospermae	
Schismatoglottis calyptrata	Araceae	Angiospermae	AM
Typhonium albidinervum	Araceae	Angiospermae	
Typhonium divaricatum	Araceae	Angiospermae	
Typhonium flagelliforme	Araceae	Angiospermae	
Typhonium giganteum	Araceae	Angiospermae	
Typhonium roxburgii	Araceae	Angiospermae	
Typhonium trifoliatum	Araceae	Angiospermae	
Typhonium trilobatum	Araceae	Angiospermae	
Steudnera colocasiifolia	Araceae	Angiospermae	
Acanthopanax evodiaefolius var. ferrugineus	Araliaceae	Angiospermae	
Acanthopanax gracilistylus	Araliaceae	Angiospermae	AM
Acanthopanax lasiogyne	Araliaceae	Angiospermae	
Acanthopanax senticosus	Araliaceae	Angiospermae	ORM
Acanthopanax sessiliflorus	Araliaceae	Angiospermae	AM
Acanthopanax trifoliatus	Araliaceae	Angiospermae	
Acanthopanax verticillatus	Araliaceae	Angiospermae	
Aralia armata	Araliaceae	Angiospermae	
Aralia chinensis	Araliaceae	Angiospermae	
Aralia cordata	Araliaceae	Angiospermae	AM
Aralia decaisneana	Araliaceae	Angiospermae	
Aralia fargesii	Araliaceae	Angiospermae	
Dendropanax dentiger	Araliaceae	Angiospermae	
Dendropanax proteus	Araliaceae	Angiospermae	
Hedera nepalensis var. sinensis	Araliaceae	Angiospermae	AM
Heteropanax brevipedicellatus	Araliaceae	Angiospermae	

Heteropanax chinensis	Araliaceae	Angiospermae	
Heteropanax fragrans	Araliaceae	Angiospermae	
Kalopanax septemlobus	Araliaceae	Angiospermae	AM+ECM+NM
Macropanax rosthornii	Araliaceae	Angiospermae	
Nothopanax davidii	Araliaceae	Angiospermae	
Nothopanax delavayi	Araliaceae	Angiospermae	
Panax ginseng	Araliaceae	Angiospermae	AM
Schefflera arboricola	Araliaceae	Angiospermae	
Schefflera delavayi	Araliaceae	Angiospermae	
Schefflera kwangsiensis	Araliaceae	Angiospermae	
Schefflera octophylla	Araliaceae	Angiospermae	
Schefflera venulosa	Araliaceae	Angiospermae	
Tetrapanax papyrifer	Araliaceae	Angiospermae	AM
Tupidanthus calyptratus	Araliaceae	Angiospermae	
Panax japonicus var. bipinnatifidus	Araliaceae	Angiospermae	
Panax pseudo-ginseng var. elegantior	Araliaceae	Angiospermae	
Panax pseudo-ginseng var. japonicus	Araliaceae	Angiospermae	
Panax pseudo-ginseng var. notoginseng	Araliaceae	Angiospermae	
Aristolochia cathcartii	Aristolochiaceae	Angiospermae	
Aristolochia championii	Aristolochiaceae	Angiospermae	
Aristolochia contorta	Aristolochiaceae	Angiospermae	
Aristolochia debilis	Aristolochiaceae	Angiospermae	AM
Aristolochia elegans	Aristolochiaceae	Angiospermae	
Aristolochia fangchi	Aristolochiaceae	Angiospermae	
Aristolochia fordiana	Aristolochiaceae	Angiospermae	
Aristolochia foveolata	Aristolochiaceae	Angiospermae	
Aristolochia hainanensis	Aristolochiaceae	Angiospermae	
Aristolochia howii	Aristolochiaceae	Angiospermae	
Aristolochia impresinervis	Aristolochiaceae	Angiospermae	
Aristolochia kwangsiensis	Aristolochiaceae	Angiospermae	
Aristolochia manshuriensis	Aristolochiaceae	Angiospermae	
Aristolochia mollissima	Aristolochiaceae	Angiospermae	AM
Aristolochia obliqua	Aristolochiaceae	Angiospermae	

Aristolochia tagala	Aristolochiaceae	Angiospermae	
Aristolochia thwaitesii	Aristolochiaceae	Angiospermae	
Aristolochia tuberosa	Aristolochiaceae	Angiospermae	
Aristolochia tubiflora	Aristolochiaceae	Angiospermae	
Aristolochia versicolor	Aristolochiaceae	Angiospermae	
Asarum caudigerellum	Aristolochiaceae	Angiospermae	
Asarum caudigerum	Aristolochiaceae	Angiospermae	
Asarum caulescens	Aristolochiaceae	Angiospermae	AM
Asarum debile	Aristolochiaceae	Angiospermae	
Asarum delavayi	Aristolochiaceae	Angiospermae	
Asarum forbesii	Aristolochiaceae	Angiospermae	
Asarum fukienense	Aristolochiaceae	Angiospermae	
Asarum geophilum	Aristolochiaceae	Angiospermae	
Asarum himalaicum	Aristolochiaceae	Angiospermae	
Asarum ichangense	Aristolochiaceae	Angiospermae	
Asarum inflatum	Aristolochiaceae	Angiospermae	
Asarum longerhizomatosum	Aristolochiaceae	Angiospermae	
Asarum magnificum	Aristolochiaceae	Angiospermae	
Asarum magnificum var. dinghuense	Aristolochiaceae	Angiospermae	
Asarum maximum	Aristolochiaceae	Angiospermae	
Asarum petelotii	Aristolochiaceae	Angiospermae	
Asarum porphyronotum var. atrovirens	Aristolochiaceae	Angiospermae	
Asarum pulchellum	Aristolochiaceae	Angiospermae	
Asarum renicordatum	Aristolochiaceae	Angiospermae	
Asarum sagittarioides	Aristolochiaceae	Angiospermae	
Asarum sieboldii	Aristolochiaceae	Angiospermae	AM
Asarum sieboldii f. seoulense	Aristolochiaceae	Angiospermae	AM
Asarum splendens	Aristolochiaceae	Angiospermae	
Asarum wulingense	Aristolochiaceae	Angiospermae	
Saruma henryi	Aristolochiaceae	Angiospermae	
Aristolochia arborea	Aristolochiaceae	Angiospermae	
Asclepias curassavica	Asclepiadaceae	Angiospermae	AM+NM
Brachystelma edule	Asclepiadaceae	Angiospermae	

Brachystelma kerrii	Asclepiadaceae	Angiospermae	
Calotropis gigantea	Asclepiadaceae	Angiospermae	AM
Calotropis procera	Asclepiadaceae	Angiospermae	AM+NM
Ceropegia mairei	Asclepiadaceae	Angiospermae	
Ceropegia trichantha	Asclepiadaceae	Angiospermae	
Cryptolepis buchananii	Asclepiadaceae	Angiospermae	AM
Cryptolepis sinensis	Asclepiadaceae	Angiospermae	
Cynanchum amplexicaule var. castaneum	Asclepiadaceae	Angiospermae	
Cynanchum atratum	Asclepiadaceae	Angiospermae	
Cynanchum auriculatum	Asclepiadaceae	Angiospermae	AM
Cynanchum bungei	Asclepiadaceae	Angiospermae	
Cynanchum chekiangense	Asclepiadaceae	Angiospermae	
Cynanchum corymbosum	Asclepiadaceae	Angiospermae	
Cynanchum dubium	Asclepiadaceae	Angiospermae	
Cynanchum forrestii	Asclepiadaceae	Angiospermae	
Cynanchum glaucescens	Asclepiadaceae	Angiospermae	
Cynanchum inamoenum	Asclepiadaceae	Angiospermae	
Cynanchum mooreanum	Asclepiadaceae	Angiospermae	
Cynanchum officinale	Asclepiadaceae	Angiospermae	
Cynanchum otophyllum	Asclepiadaceae	Angiospermae	
Cynanchum paniculatum	Asclepiadaceae	Angiospermae	
Cynanchum sibiricum	Asclepiadaceae	Angiospermae	AM
Cynanchum stauntonii	Asclepiadaceae	Angiospermae	
Cynanchum versicolor	Asclepiadaceae	Angiospermae	
Cynanchum wilfordii	Asclepiadaceae	Angiospermae	
Dischidanthus urceolatus	Asclepiadaceae	Angiospermae	
Dischidia australis	Asclepiadaceae	Angiospermae	
Dischidia chinensis	Asclepiadaceae	Angiospermae	
Dolichopetalum kwangsiense	Asclepiadaceae	Angiospermae	
Dregea sinensis	Asclepiadaceae	Angiospermae	
Dregea volubilis	Asclepiadaceae	Angiospermae	
Gomphocarpus fruticosus	Asclepiadaceae	Angiospermae	AM
Gongronema nepalense	Asclepiadaceae	Angiospermae	

Graphistemma pictum	Asclepiadaceae	Angiospermae	
Gymnema longiretinaculatum	Asclepiadaceae	Angiospermae	
Gymnema sylvestre	Asclepiadaceae	Angiospermae	AM+NM
Gymnema tingens	Asclepiadaceae	Angiospermae	
Heterostemma alatum	Asclepiadaceae	Angiospermae	
Heterostemma oblongifolium	Asclepiadaceae	Angiospermae	
Holostemma annulare	Asclepiadaceae	Angiospermae	
Hoya carnosa	Asclepiadaceae	Angiospermae	AM
Hoya carnosa var. marmorata	Asclepiadaceae	Angiospermae	AM
Hoya formosana	Asclepiadaceae	Angiospermae	
Hoya fungii	Asclepiadaceae	Angiospermae	
Hoya lancilimba	Asclepiadaceae	Angiospermae	
Hoya lancilimba f. tsoi	Asclepiadaceae	Angiospermae	
Hoya pottsii	Asclepiadaceae	Angiospermae	
Hoya pottsii var. angustifolia	Asclepiadaceae	Angiospermae	
Marsdenia griffithii	Asclepiadaceae	Angiospermae	
Marsdenia longipes	Asclepiadaceae	Angiospermae	
Marsdenia officinalis	Asclepiadaceae	Angiospermae	
Marsdenia sinensis	Asclepiadaceae	Angiospermae	
Marsdenia tenacissima	Asclepiadaceae	Angiospermae	
Marsdenia tomentosa	Asclepiadaceae	Angiospermae	AM
Metaplexis hemsleyana	Asclepiadaceae	Angiospermae	
Metaplexis japonica	Asclepiadaceae	Angiospermae	AM
Myriopteron extensum	Asclepiadaceae	Angiospermae	
Oxystelma esculentum	Asclepiadaceae	Angiospermae	
Pentasacme championii	Asclepiadaceae	Angiospermae	
Periploca calophylla	Asclepiadaceae	Angiospermae	
Periploca forrestii	Asclepiadaceae	Angiospermae	
Periploca sepium	Asclepiadaceae	Angiospermae	AM
Sarcostemma acidum	Asclepiadaceae	Angiospermae	
Secamone lanceolata	Asclepiadaceae	Angiospermae	
Secamone sinica	Asclepiadaceae	Angiospermae	
Stelmatocrypton khasianum	Asclepiadaceae	Angiospermae	

Stephanotis chunii	Asclepiadaceae	Angiospermae	
Streptocaulon griffithii	Asclepiadaceae	Angiospermae	
Telosma cordata	Asclepiadaceae	Angiospermae	
Toxocarpus wightianus	Asclepiadaceae	Angiospermae	
Tylophora arenicola	Asclepiadaceae	Angiospermae	AM
Tylophora atrofolliculata	Asclepiadaceae	Angiospermae	
Tylophora floribunda	Asclepiadaceae	Angiospermae	
Tylophora kerrii	Asclepiadaceae	Angiospermae	
Tylophora koi	Asclepiadaceae	Angiospermae	
Tylophora mollissima	Asclepiadaceae	Angiospermae	
Tylophora ovata	Asclepiadaceae	Angiospermae	
Tylophora renchangii	Asclepiadaceae	Angiospermae	
Tylophora secamonoides	Asclepiadaceae	Angiospermae	
Tylophora trichophylla	Asclepiadaceae	Angiospermae	
Tylophora yunnanensis	Asclepiadaceae	Angiospermae	
Smilacina nokomoticola	Asparagaceae	Angiospermae	
Dendranthema morii	Asteraceae	Angiospermae	
Inula aspera	Asteraceae	Angiospermae	
Inula simonii	Asteraceae	Angiospermae	
Inula verrucosa	Asteraceae	Angiospermae	
Balanophora fargesii	Balanophoraceae	Angiospermae	
Balanophora formosana	Balanophoraceae	Angiospermae	
Balanophora indica	Balanophoraceae	Angiospermae	
Balanophora involucrata	Balanophoraceae	Angiospermae	
Balanophora laxiflora	Balanophoraceae	Angiospermae	
Balanophora parvior	Balanophoraceae	Angiospermae	
Impatiens apalophylla	Balsaminaceae	Angiospermae	
Impatiens arguta	Balsaminaceae	Angiospermae	
Impatiens balsamina	Balsaminaceae	Angiospermae	AM+NM
Impatiens chinensis	Balsaminaceae	Angiospermae	AM
Impatiens chlorosepala	Balsaminaceae	Angiospermae	
Impatiens claviger	Balsaminaceae	Angiospermae	
Impatiens cyanantha	Balsaminaceae	Angiospermae	

Impatiens lasiophyton	Balsaminaceae	Angiospermae	
Impatiens leptocaulon	Balsaminaceae	Angiospermae	
Impatiens loulanensis	Balsaminaceae	Angiospermae	
Impatiens pritzelii	Balsaminaceae	Angiospermae	
Impatiens siculifer	Balsaminaceae	Angiospermae	
Impatiens uliginosa	Balsaminaceae	Angiospermae	
Anredera cordifolia	Basellaceae	Angiospermae	
Anredera scandens	Basellaceae	Angiospermae	
Basella alba	Basellaceae	Angiospermae	NM
Begonia argenteo-guttata	Begoniaceae	Angiospermae	
Begonia maculata	Begoniaceae	Angiospermae	AM
Berberis aggregata	Berberidaceae	Angiospermae	
Berberis amurensis	Berberidaceae	Angiospermae	AM+NM
Berberis brachypoda	Berberidaceae	Angiospermae	
Berberis circumserrata	Berberidaceae	Angiospermae	
Berberis dasystachya	Berberidaceae	Angiospermae	
Berberis dielsiana	Berberidaceae	Angiospermae	
Berberis ferdinandi-coburgii	Berberidaceae	Angiospermae	
Berberis henryana	Berberidaceae	Angiospermae	
Berberis hsuyunensis	Berberidaceae	Angiospermae	
Berberis julianae	Berberidaceae	Angiospermae	
Berberis lempergiana	Berberidaceae	Angiospermae	
Berberis poiretii	Berberidaceae	Angiospermae	ORM
Berberis polyantha	Berberidaceae	Angiospermae	
Berberis pruinosa var. pruinosa	Berberidaceae	Angiospermae	
Berberis sargentiana	Berberidaceae	Angiospermae	AM
Berberis sibirica	Berberidaceae	Angiospermae	AM
Berberis thunbergii	Berberidaceae	Angiospermae	AM
Berberis vernae	Berberidaceae	Angiospermae	
Berberis verruculosa	Berberidaceae	Angiospermae	
Berberis virgetorum	Berberidaceae	Angiospermae	
Berberis wilsonae	Berberidaceae	Angiospermae	
Berberis xinganensis	Berberidaceae	Angiospermae	

Caulophyllum robustum	Berberidaceae	Angiospermae	
Diphylleia sinensis	Berberidaceae	Angiospermae	
Dysosma aurantiocaulis	Berberidaceae	Angiospermae	
Dysosma difformis	Berberidaceae	Angiospermae	
Dysosma majorensis	Berberidaceae	Angiospermae	
Dysosma pleiantha	Berberidaceae	Angiospermae	
Dysosma veitchii	Berberidaceae	Angiospermae	
Dysosma versipellis	Berberidaceae	Angiospermae	ORM
Epimedium acuminatum	Berberidaceae	Angiospermae	
Epimedium boreali-guizhouense	Berberidaceae	Angiospermae	
Epimedium brevicornu	Berberidaceae	Angiospermae	
Epimedium koreanum	Berberidaceae	Angiospermae	
Epimedium pubescens	Berberidaceae	Angiospermae	
Epimedium sagittatum	Berberidaceae	Angiospermae	
Gymnospermium kiangnanense	Berberidaceae	Angiospermae	
Nandina domestica	Berberidaceae	Angiospermae	AM+ECM+NM
Plagiorhegma dubia	Berberidaceae	Angiospermae	
Sinopodophyllum hexandrum	Berberidaceae	Angiospermae	AM
Betula chinensis	Betulaceae	Angiospermae	
Campsis grandiflora	Bignoniaceae	Angiospermae	AM
Campsis radicans	Bignoniaceae	Angiospermae	
Catalpa bungei	Bignoniaceae	Angiospermae	AM
Catalpa fargesii	Bignoniaceae	Angiospermae	
Catalpa fargesii f. duclouxii	Bignoniaceae	Angiospermae	
Catalpa ovata	Bignoniaceae	Angiospermae	
Incarvillea arguta	Bignoniaceae	Angiospermae	AM
Incarvillea compacta	Bignoniaceae	Angiospermae	
Incarvillea delavayi	Bignoniaceae	Angiospermae	
Incarvillea lutea	Bignoniaceae	Angiospermae	
Incarvillea mairei	Bignoniaceae	Angiospermae	
Incarvillea mairei var. grandiflora	Bignoniaceae	Angiospermae	
Incarvillea younghusbandii	Bignoniaceae	Angiospermae	AM
Kigelia africana	Bignoniaceae	Angiospermae	NM

Millingtonia hortensis	Bignoniaceae	Angiospermae	AM
Oroxylum indicum	Bignoniaceae	Angiospermae	AM
Radermachera hainanensis	Bignoniaceae	Angiospermae	
Radermachera pentandra	Bignoniaceae	Angiospermae	
Radermachera sinica	Bignoniaceae	Angiospermae	AM
Radermachera yunnanensis	Bignoniaceae	Angiospermae	
Bixa orellana	Bixaceae	Angiospermae	AM
Woodwardia cochi-chinensis	Blechnaceae	Angiospermae	
Bombax malabaricum	Bombacaceae	Angiospermae	AM
Antiotrema dunnianum	Boraginaceae	Angiospermae	
Arnebia euchroma	Boraginaceae	Angiospermae	
Arnebia guttata	Boraginaceae	Angiospermae	
Cordia dichotoma	Boraginaceae	Angiospermae	
Cynoglossum amabile var. amabile	Boraginaceae	Angiospermae	
Cynoglossum divaricatum	Boraginaceae	Angiospermae	
Cynoglossum officinale	Boraginaceae	Angiospermae	AM
Cynoglossum zeylanicum	Boraginaceae	Angiospermae	AM
Heliotropium indicum	Boraginaceae	Angiospermae	AM
Lappula heteracantha	Boraginaceae	Angiospermae	
Lithospermum erythrorhizon	Boraginaceae	Angiospermae	
Lithospermum officinale	Boraginaceae	Angiospermae	AM+NM
Lithospermum zollingeri	Boraginaceae	Angiospermae	
Thyrocarpus sampsonii	Boraginaceae	Angiospermae	
Trigonotis macrophylla var. verrucosa	Boraginaceae	Angiospermae	
Trigonotis peduncularis	Boraginaceae	Angiospermae	AM
Canarium album	Burseraceae	Angiospermae	AM
Canarium pimela	Burseraceae	Angiospermae	AM
Commiphora myrrha	Burseraceae	Angiospermae	
Boswellia carteri	Burseraceae	Angiospermae	
Opuntia monacantha	Cactaceae	Angiospermae	AM
Opuntia stricta var. dillenii	Cactaceae	Angiospermae	AM
Chimonanthus nitens	Calycanthaceae	Angiospermae	AM
Chimonanthus praecox	Calycanthaceae	Angiospermae	AM

Campanula colorata	Campanulaceae	Angiospermae	
Campanumoea javanica	Campanulaceae	Angiospermae	
Campanumoea lancifolia	Campanulaceae	Angiospermae	
Lobelia chinensis	Campanulaceae	Angiospermae	
Lobelia davidii var. davidii	Campanulaceae	Angiospermae	
Lobelia melliana	Campanulaceae	Angiospermae	
Lobelia sequinii	Campanulaceae	Angiospermae	
Lobelia sessilifolia	Campanulaceae	Angiospermae	
Platycodon grandiflorus	Campanulaceae	Angiospermae	AM
Pratia nummularia	Campanulaceae	Angiospermae	
Wahlenbergia marginata	Campanulaceae	Angiospermae	
Canna indica	Cannaceae	Angiospermae	AM
Capparis acutifolia	Capparaceae	Angiospermae	
Capparis bodinieri	Capparaceae	Angiospermae	
Capparis cantoniensis	Capparaceae	Angiospermae	
Capparis chingiana	Capparaceae	Angiospermae	
Capparis masaikai	Capparaceae	Angiospermae	
Capparis versicolor	Capparaceae	Angiospermae	
Capparis yunnanensis	Capparaceae	Angiospermae	
Cleome gynandra	Capparaceae	Angiospermae	AM+NM
Cleome viscosa	Capparaceae	Angiospermae	AM+ECM+NM
Leycesteria formosa	Caprifoliaceae	Angiospermae	
Leycesteria formosa var. stenosepala	Caprifoliaceae	Angiospermae	
Lonicera acuminata	Caprifoliaceae	Angiospermae	
Lonicera buchananii	Caprifoliaceae	Angiospermae	
Lonicera confusa	Caprifoliaceae	Angiospermae	ORM
Lonicera crassifolia	Caprifoliaceae	Angiospermae	
Lonicera dasystyla	Caprifoliaceae	Angiospermae	
Lonicera hispida	Caprifoliaceae	Angiospermae	AM
Lonicera hypoglauca	Caprifoliaceae	Angiospermae	
Lonicera hypoglauca subsp. nudiflora	Caprifoliaceae	Angiospermae	
Lonicera inodora	Caprifoliaceae	Angiospermae	
Lonicera macranthoides	Caprifoliaceae	Angiospermae	

Lonicera pampaninii	Caprifoliaceae	Angiospermae	
Lonicera rhytidophylla	Caprifoliaceae	Angiospermae	
Lonicera similis	Caprifoliaceae	Angiospermae	
Lonicera similis var. omeiensis	Caprifoliaceae	Angiospermae	
Lonicera tragophylla	Caprifoliaceae	Angiospermae	
Sambucus adnata	Caprifoliaceae	Angiospermae	AM
Sambucus chinensis	Caprifoliaceae	Angiospermae	
Viburnum cylindricum	Caprifoliaceae	Angiospermae	
Viburnum odoratissimum var. odoratissimum	Caprifoliaceae	Angiospermae	
Viburnum odoratissinum	Caprifoliaceae	Angiospermae	
Viburnum subalpinum	Caprifoliaceae	Angiospermae	
Carica papaya	Caricaceae	Angiospermae	AM
Agrostemma githago	Caryophyllaceae	Angiospermae	AM+NM
Arenaria brevipetala	Caryophyllaceae	Angiospermae	
Arenaria juncea	Caryophyllaceae	Angiospermae	
Arenaria melanandra	Caryophyllaceae	Angiospermae	
Arenaria przewalskii	Caryophyllaceae	Angiospermae	
Arenaria serpyllifolia	Caryophyllaceae	Angiospermae	AM+NM
Brachystemma calycinum	Caryophyllaceae	Angiospermae	
Cucubalus baccifer	Caryophyllaceae	Angiospermae	
Dianthus chinensis	Caryophyllaceae	Angiospermae	AM+NM
Dianthus chinensis var. longisquama	Caryophyllaceae	Angiospermae	AM+NM
Dianthus chinensis var. versicolor	Caryophyllaceae	Angiospermae	AM+NM
Dianthus superbus	Caryophyllaceae	Angiospermae	NM
Drymaria diandra	Caryophyllaceae	Angiospermae	
Gypsophila davurica	Caryophyllaceae	Angiospermae	
Gypsophila oldhamiana	Caryophyllaceae	Angiospermae	
Gypsophila pacifica	Caryophyllaceae	Angiospermae	
Gypsophila paniculata	Caryophyllaceae	Angiospermae	NM
Lepyrodiclis holosteoides	Caryophyllaceae	Angiospermae	
Lychnis coronata	Caryophyllaceae	Angiospermae	
Lychnis senno	Caryophyllaceae	Angiospermae	
Myosoton aquaticum	Caryophyllaceae	Angiospermae	AM+NM

Psammosilene tunicoides	Caryophyllaceae	Angiospermae	
Pseudostellaria heterophylla	Caryophyllaceae	Angiospermae	NM
Sagina japonica	Caryophyllaceae	Angiospermae	NM
Saponaria officinalis	Caryophyllaceae	Angiospermae	NM
Silene aprica	Caryophyllaceae	Angiospermae	
Silene conoidea	Caryophyllaceae	Angiospermae	AM
Silene fortunei	Caryophyllaceae	Angiospermae	AM
Silene gracilicaulis	Caryophyllaceae	Angiospermae	
Silene gracilicaulis var. rubescens	Caryophyllaceae	Angiospermae	
Silene jenisseensis	Caryophyllaceae	Angiospermae	
Silene viscidula	Caryophyllaceae	Angiospermae	AM
Stellaria chinensis	Caryophyllaceae	Angiospermae	
Stellaria dichotoma var. lanceolata	Caryophyllaceae	Angiospermae	
Stellaria henryi	Caryophyllaceae	Angiospermae	
Stellaria media	Caryophyllaceae	Angiospermae	AM+NM
Stellaria neglecta	Caryophyllaceae	Angiospermae	
Stellaria uliginosa	Caryophyllaceae	Angiospermae	AM+NM
Stellaria vestita	Caryophyllaceae	Angiospermae	
Stellaria yunnanensis	Caryophyllaceae	Angiospermae	
Vaccaria segetalis	Caryophyllaceae	Angiospermae	
Casuarina equisetifolia	Casuarinaceae	Angiospermae	AM
Celastrus orbiculatus	Celastraceae	Angiospermae	AM
Celastrus paniculatus	Celastraceae	Angiospermae	
Celastrus rosthornianus	Celastraceae	Angiospermae	
Euonymus alatus	Celastraceae	Angiospermae	AM+ECM+NM
Euonymus laxiflorus	Celastraceae	Angiospermae	AM
Maytenus confertiflorus	Celastraceae	Angiospermae	
Maytenus guangxiensis	Celastraceae	Angiospermae	
Ceratophyllum demersum	Ceratophyllaceae	Angiospermae	AM+NM
Anabasis aphylla	Chenopodiaceae	Angiospermae	NM
Atriplex centralasiatica	Chenopodiaceae	Angiospermae	AM
Chenopodium album	Chenopodiaceae	Angiospermae	AM+NM
Chenopodium ambrosioides	Chenopodiaceae	Angiospermae	AM+NM

Chenopodium aristatum	Chenopodiaceae	Angiospermae	
Chenopodium hybridum	Chenopodiaceae	Angiospermae	NM
Kochia scoparia	Chenopodiaceae	Angiospermae	AM
Salsola collina	Chenopodiaceae	Angiospermae	NM
Chloranthus fortunei	ChloranthaceaeOrchidaceae	Angiospermae	
Chloranthus henryi	ChloranthaceaeOrchidaceae	Angiospermae	
Chloranthus henryi var. hupehensis	ChloranthaceaeOrchidaceae	Angiospermae	
Chloranthus holostegius	ChloranthaceaeOrchidaceae	Angiospermae	
Chloranthus japonicus	ChloranthaceaeOrchidaceae	Angiospermae	AM
Chloranthus multistachys	ChloranthaceaeOrchidaceae	Angiospermae	ORM
Chloranthus serratus	ChloranthaceaeOrchidaceae	Angiospermae	AM
Chloranthus sessilifolius	ChloranthaceaeOrchidaceae	Angiospermae	
Chloranthus spicatus	ChloranthaceaeOrchidaceae	Angiospermae	AM
Sarcandra glabra	ChloranthaceaeOrchidaceae	Angiospermae	AM+NM
Sarcandra hainanensis	ChloranthaceaeOrchidaceae	Angiospermae	
Calycopteris floribunda	Combretaceae	Angiospermae	
Quisqualis indica	Combretaceae	Angiospermae	
Terminalia bellirica	Combretaceae	Angiospermae	AM
Terminalia catappa	Combretaceae	Angiospermae	AM
Terminalia chebula	Combretaceae	Angiospermae	AM
Commelina bengalensis	Commelinaceae	Angiospermae	AM+NM
Commelina communis	Commelinaceae	Angiospermae	AM+NM
Commelina diffusa	Commelinaceae	Angiospermae	AM+NM
Commelina paludosa	Commelinaceae	Angiospermae	
Cyanotis arachnoidea	Commelinaceae	Angiospermae	
Cyanotis cristata	Commelinaceae	Angiospermae	AM+NM
Floscopa scandens	Commelinaceae	Angiospermae	
Murdannia nudiflora	Commelinaceae	Angiospermae	NM
Murdannia triquetra	Commelinaceae	Angiospermae	
Pollia japonica	Commelinaceae	Angiospermae	NM
Pollia miranda	Commelinaceae	Angiospermae	
Ageratum conyzoides	Compositae	Angiospermae	AM+NM
Ageratum houstonianum	Compositae	Angiospermae	AM

Ainsliaea elegans var. strigosa	Compositae	Angiospermae	
Ainsliaea fragrans	Compositae	Angiospermae	
Ainsliaea pertyoides var. albo-tomentosa	Compositae	Angiospermae	
Ajania variifolia	Compositae	Angiospermae	
Anaphalis bulleyana	Compositae	Angiospermae	
Anaphalis flavescens var. lanata	Compositae	Angiospermae	
Anaphalis lactea	Compositae	Angiospermae	AM
Anaphalis nepalensis	Compositae	Angiospermae	
Anaphalis porphyrolepis	Compositae	Angiospermae	
Antennaria dioica	Compositae	Angiospermae	AM
Artemisia absinthium	Compositae	Angiospermae	AM+NM
Artemisia anethifolia	Compositae	Angiospermae	AM
Artemisia anethoides	Compositae	Angiospermae	
Artemisia angustissima	Compositae	Angiospermae	
Artemisia annua	Compositae	Angiospermae	AM
Artemisia anomala	Compositae	Angiospermae	
Artemisia anomala var. tomentella	Compositae	Angiospermae	
Artemisia argyi	Compositae	Angiospermae	AM
Artemisia argyi var. gracilis	Compositae	Angiospermae	AM
Artemisia brachyloba	Compositae	Angiospermae	AM
Artemisia capillaris	Compositae	Angiospermae	AM+NM
Artemisia carvifolia	Compositae	Angiospermae	ORM
Artemisia conaensis	Compositae	Angiospermae	
Artemisia dracunculus	Compositae	Angiospermae	AM
Artemisia dracunculus var. turkestanica	Compositae	Angiospermae	AM
Artemisia dubia	Compositae	Angiospermae	AM
Artemisia dubia var. subdigitata	Compositae	Angiospermae	AM
Artemisia edgeworthii	Compositae	Angiospermae	
Artemisia edgeworthii var. diffusa	Compositae	Angiospermae	
Artemisia eriopoda	Compositae	Angiospermae	AM
Artemisia frigida	Compositae	Angiospermae	AM+NM
Artemisia gilvescens	Compositae	Angiospermae	
Artemisia giraldii	Compositae	Angiospermae	AM

Artemisia hedinii	Compositae	Angiospermae	
Artemisia igniaria	Compositae	Angiospermae	
Artemisia japonica	Compositae	Angiospermae	AM
Artemisia japonica var. hainanensis	Compositae	Angiospermae	AM
Artemisia keiskeana	Compositae	Angiospermae	
Artemisia lactiflora	Compositae	Angiospermae	
Artemisia lactiflora var. incisa	Compositae	Angiospermae	
Artemisia lavandulaefolia	Compositae	Angiospermae	AM
Artemisia leucophylla	Compositae	Angiospermae	AM
Artemisia littoricola	Compositae	Angiospermae	AM
Artemisia macrocephala	Compositae	Angiospermae	
Artemisia manshurica	Compositae	Angiospermae	
Artemisia mongolica	Compositae	Angiospermae	AM
Artemisia moorcroftiana	Compositae	Angiospermae	
Artemisia ordosica	Compositae	Angiospermae	AM+NM
Artemisia orientali-hengduangensis	Compositae	Angiospermae	
Artemisia parviflora	Compositae	Angiospermae	
Artemisia princeps	Compositae	Angiospermae	AM
Artemisia qinlingensis	Compositae	Angiospermae	
Artemisia roxburghiana	Compositae	Angiospermae	AM
Artemisia rubripes	Compositae	Angiospermae	AM
Artemisia rupestris	Compositae	Angiospermae	AM
Artemisia sacrorum	Compositae	Angiospermae	AM
Artemisia sacrorum var. incana	Compositae	Angiospermae	NM
Artemisia sacrorum var. messerschmidtiana	Compositae	Angiospermae	NM
Artemisia selengensis	Compositae	Angiospermae	
Artemisia sieversiana	Compositae	Angiospermae	AM
Artemisia sphaerocephala	Compositae	Angiospermae	AM+NM
Artemisia tournefortiana	Compositae	Angiospermae	
Artemisia verbenacea	Compositae	Angiospermae	
Artemisia verlotorum	Compositae	Angiospermae	AM
Artemisia vestita	Compositae	Angiospermae	AM
Artemisia vulgaris	Compositae	Angiospermae	AM+NM

Artemisia wellbyi	Compositae	Angiospermae	AM
Aster ageratoides	Compositae	Angiospermae	AM
Aster ageratoides var. lasiocladus	Compositae	Angiospermae	AM
Aster alatipes	Compositae	Angiospermae	
Aster auriculatus	Compositae	Angiospermae	
Aster auriculatus f. crenatus	Compositae	Angiospermae	
Aster batangensis var. staticefolius	Compositae	Angiospermae	
Aster flaccidus	Compositae	Angiospermae	
Aster likiangensis	Compositae	Angiospermae	
Aster likiangensis f. polianthus	Compositae	Angiospermae	
Aster oreophilus	Compositae	Angiospermae	
Aster oreophilus f. inaequisquamus	Compositae	Angiospermae	
Aster oreophilus f. umbrosus	Compositae	Angiospermae	
Aster prainii	Compositae	Angiospermae	
Aster souliei	Compositae	Angiospermae	
Aster turbinatus var. chekiangensis	Compositae	Angiospermae	
Atractylodes lancea	Compositae	Angiospermae	AM
Atractylodes macrocephala	Compositae	Angiospermae	AM
Bidens bipinnata	Compositae	Angiospermae	AM
Bidens biternata	Compositae	Angiospermae	AM+NM
Bidens frondosa	Compositae	Angiospermae	AM
Bidens parviflora	Compositae	Angiospermae	AM
Bidens pilosa	Compositae	Angiospermae	AM+NM
Bidens pilosa var. radiata	Compositae	Angiospermae	AM+NM
Bidens tripartita	Compositae	Angiospermae	AM+NM
Blumea balsamifera	Compositae	Angiospermae	AM
Carpesium abrotanoides	Compositae	Angiospermae	AM
Carpesium cernuum	Compositae	Angiospermae	
Carpesium divaricatum	Compositae	Angiospermae	AM
Carpesium lipskyi	Compositae	Angiospermae	AM
Carpesium macrocephalum	Compositae	Angiospermae	
Carpesium nepalense var. lanatum	Compositae	Angiospermae	
Carthamus tinctorius	Compositae	Angiospermae	AM

Cavea tanguensis	Compositae	Angiospermae	
Cavea tanguensis f. acaulis	Compositae	Angiospermae	
Centipeda minima	Compositae	Angiospermae	AM
Conyza blinii	Compositae	Angiospermae	AM
Conyza bonariensis	Compositae	Angiospermae	NM
Conyza canadensis	Compositae	Angiospermae	AM
Conyza japonica	Compositae	Angiospermae	AM
Crassocephalum crepidioides	Compositae	Angiospermae	AM
Crepis phoenix	Compositae	Angiospermae	
Dahlia pinnata	Compositae	Angiospermae	AM
Dendranthema indicum	Compositae	Angiospermae	AM
Dichrocephala auriculata	Compositae	Angiospermae	
Dichrocephala benthamii	Compositae	Angiospermae	
Doellingeria marchandii	Compositae	Angiospermae	
Doellingeria scaber	Compositae	Angiospermae	AM
Dolomiaea souliei	Compositae	Angiospermae	
Eclipta prostrata	Compositae	Angiospermae	AM
Elephantopus scaber	Compositae	Angiospermae	AM
Elephantopus tomentosus	Compositae	Angiospermae	
Emilia sonchifolia	Compositae	Angiospermae	AM+NM
Erigeron annuus	Compositae	Angiospermae	AM
Erigeron breviscapus	Compositae	Angiospermae	ORM
Eupatorium chinense	Compositae	Angiospermae	AM
Eupatorium fortunei	Compositae	Angiospermae	
Eupatorium fortunei var. angustilobum	Compositae	Angiospermae	
Eupatorium heterophyllum	Compositae	Angiospermae	
Eupatorium japonicum	Compositae	Angiospermae	
Eupatorium lindleyanum	Compositae	Angiospermae	
Eupatorium odoratum	Compositae	Angiospermae	AM
Farfugium japonicum	Compositae	Angiospermae	AM
Galinsoga parviflora	Compositae	Angiospermae	AM
Gerbera delavayi	Compositae	Angiospermae	
Gerbera piloselloides	Compositae	Angiospermae	

Glossogyne tenuifolia	Compositae	Angiospermae	AM
Gnaphalium affine	Compositae	Angiospermae	AM
Helianthus annuus	Compositae	Angiospermae	AM
Helianthus tuberosus	Compositae	Angiospermae	AM
Helichrysum arenarium	Compositae	Angiospermae	AM
Hippolytia delavayi	Compositae	Angiospermae	
Hippolytia kennedyi	Compositae	Angiospermae	
Inula britannica	Compositae	Angiospermae	AM+NM
Inula helenium	Compositae	Angiospermae	AM
Inula helianthus-aquatica	Compositae	Angiospermae	AM
Inula hookeri	Compositae	Angiospermae	
Inula hookeri f. major	Compositae	Angiospermae	
Inula japonica	Compositae	Angiospermae	AM
Inula leariifolia	Compositae	Angiospermae	
Inula nervosa	Compositae	Angiospermae	
Inula pterocaula	Compositae	Angiospermae	
Inula racemosa	Compositae	Angiospermae	
Inula salicina	Compositae	Angiospermae	AM
Ixeridium sonchifolium	Compositae	Angiospermae	
Ixeris polycephala	Compositae	Angiospermae	AM
Kalimeris indica	Compositae	Angiospermae	AM
Leontopodium andersonii	Compositae	Angiospermae	
Leontopodium artemisiifolium	Compositae	Angiospermae	
Leontopodium calocephalum var. uliginosum	Compositae	Angiospermae	
Leontopodium japonicum	Compositae	Angiospermae	AM
Leontopodium leontopodioides	Compositae	Angiospermae	AM
Ligularia hodgsonii	Compositae	Angiospermae	
Ligularia sibirica	Compositae	Angiospermae	AM
Matricaria recutita	Compositae	Angiospermae	AM+NM
Microglossa pyrifolia	Compositae	Angiospermae	
Myriactis nepalensis	Compositae	Angiospermae	
Pentanema indicum	Compositae	Angiospermae	AM+NM
Pentanema indicum var. hypoleucum	Compositae	Angiospermae	AM+NM

Petasites japonicus	Compositae	Angiospermae	AM
Petasites tricholobus	Compositae	Angiospermae	
Picris japonica	Compositae	Angiospermae	
Pulicaria chrysantha	Compositae	Angiospermae	
Pulicaria dysenterica	Compositae	Angiospermae	AM
Pulicaria insignis	Compositae	Angiospermae	
Pulicaria prostrata	Compositae	Angiospermae	
Pulicaria uliginosa	Compositae	Angiospermae	
Pyrethrum cinerariifolium	Compositae	Angiospermae	ORM
Pyrethrum tatsienense	Compositae	Angiospermae	
Rhynchospermum verticillatum	Compositae	Angiospermae	
Saussurea costus	Compositae	Angiospermae	AM
Saussurea gossypiphora	Compositae	Angiospermae	AM
Saussurea medusa	Compositae	Angiospermae	
Saussurea obvallata	Compositae	Angiospermae	
Scorzonera divaricata var. sublilacina	Compositae	Angiospermae	
Scorzonera muriculata	Compositae	Angiospermae	AM
Seriphidium brevifolium	Compositae	Angiospermae	
Seriphidium cinum	Compositae	Angiospermae	
Seriphidium transiliense	Compositae	Angiospermae	AM
Siegesbeckia orientalis	Compositae	Angiospermae	AM
Sinosenecio homogyniphyllus	Compositae	Angiospermae	
Solidago decurrens	Compositae	Angiospermae	
Solidago virgaurea	Compositae	Angiospermae	AM
Sonchus oleraceus	Compositae	Angiospermae	AM+NM
Spilanthes callimorpha	Compositae	Angiospermae	AM
Spilanthes paniculata	Compositae	Angiospermae	
Stemmacantha carthamoides	Compositae	Angiospermae	
Stemmacantha uniflora	Compositae	Angiospermae	
Syneilesis aconitifolia	Compositae	Angiospermae	
Taraxacum mongolicum	Compositae	Angiospermae	AM
Taraxacum platypecidum	Compositae	Angiospermae	AM
Tussilago farfara	Compositae	Angiospermae	AM+NM

Vernonia anthelmintica	Compositae	Angiospermae	AM
Vernonia chingiana	Compositae	Angiospermae	
Vernonia cinerea	Compositae	Angiospermae	
Vernonia cumingiana	Compositae	Angiospermae	
Vernonia esculenta	Compositae	Angiospermae	AM
Vernonia extensa	Compositae	Angiospermae	
Vernonia parishii	Compositae	Angiospermae	
Vernonia patula	Compositae	Angiospermae	
Vernonia saligna	Compositae	Angiospermae	
Vernonia solanifolia	Compositae	Angiospermae	
Vernonia squarrosa	Compositae	Angiospermae	
Waldheimia glabra	Compositae	Angiospermae	
Xanthium sibiricum	Compositae	Angiospermae	AM
Youngia stenoma	Compositae	Angiospermae	
Rourea microphylla	Connaraceae	Angiospermae	AM
Aniseia biflora	Convolvulaceae	Angiospermae	
Argyreia acuta	Convolvulaceae	Angiospermae	
Argyreia capitata	Convolvulaceae	Angiospermae	
Argyreia obtusifolia	Convolvulaceae	Angiospermae	
Argyreia pierreana	Convolvulaceae	Angiospermae	
Argyreia seguinii	Convolvulaceae	Angiospermae	
Argyreia wallichii	Convolvulaceae	Angiospermae	
Blinkworthia convolvuloides	Convolvulaceae	Angiospermae	
Calonyction muricatum	Convolvulaceae	Angiospermae	
Calystegia hederacea	Convolvulaceae	Angiospermae	AM
Calystegia sepium	Convolvulaceae	Angiospermae	AM
Convolvulus arvensis	Convolvulaceae	Angiospermae	AM+NM
Cuscuta australis	Convolvulaceae	Angiospermae	AM
Cuscuta campestris	Convolvulaceae	Angiospermae	AM
Cuscuta chinensis	Convolvulaceae	Angiospermae	
Cuscuta japonica	Convolvulaceae	Angiospermae	
Cuscuta reflexa var. reflexa	Convolvulaceae	Angiospermae	
Dichondra repens	Convolvulaceae	Angiospermae	AM

Erycibe obtusifolia	Convolvulaceae	Angiospermae	NM
Erycibe schmidtii	Convolvulaceae	Angiospermae	
Evolvulus alsinoides	Convolvulaceae	Angiospermae	AM+NM
Evolvulus alsinoides var. decumbens	Convolvulaceae	Angiospermae	AM
Ipomoea aquatica	Convolvulaceae	Angiospermae	AM+NM
Ipomoea cairica	Convolvulaceae	Angiospermae	NM
Ipomoea digitata	Convolvulaceae	Angiospermae	
Ipomoea pes-caprae	Convolvulaceae	Angiospermae	AM+NM
Merremia hungaiensis var. linifolia	Convolvulaceae	Angiospermae	
Merremia sibirica	Convolvulaceae	Angiospermae	
Merremia umbellata subsp. orientalis	Convolvulaceae	Angiospermae	AM
Operculina turpethum	Convolvulaceae	Angiospermae	AM+NM
Parasitipomoea formosana	Convolvulaceae	Angiospermae	
Pharbitis nil	Convolvulaceae	Angiospermae	AM
Porana mairei	Convolvulaceae	Angiospermae	
Porana racemosa	Convolvulaceae	Angiospermae	AM
Coriaria nepalensis	Coriariaceae	Angiospermae	AM
Cornus officinalis	Cornaceae	Angiospermae	AM
Dendrobenthamia capitata	Cornaceae	Angiospermae	
Dendrobenthamia melanotricha	Cornaceae	Angiospermae	
Helwingia japonica	Cornaceae	Angiospermae	AM+NM
Swida oblonga	Cornaceae	Angiospermae	
Swida paucinervis	Cornaceae	Angiospermae	
Toricellia angulata var. intermedia	Cornaceae	Angiospermae	
Bryophyllum pinnatum	Crassulaceae	Angiospermae	
Hylotelephium erythrostictum	Crassulaceae	Angiospermae	AM
Hylotelephium mingjinianum	Crassulaceae	Angiospermae	
Hylotelephium verticillatum	Crassulaceae	Angiospermae	
Kalanchoe laciniata	Crassulaceae	Angiospermae	
Orostachys fimbriatus	Crassulaceae	Angiospermae	AM
Rhodiola algida var. tangutica	Crassulaceae	Angiospermae	
Rhodiola dumulosa	Crassulaceae	Angiospermae	
Rhodiola henryi	Crassulaceae	Angiospermae	

Rhodiola kirilowii	Crassulaceae	Angiospermae	
Rhodiola yunnanensis	Crassulaceae	Angiospermae	
Sedum aizoon	Crassulaceae	Angiospermae	AM
Sedum elatinoides	Crassulaceae	Angiospermae	
Sedum emarginatum	Crassulaceae	Angiospermae	
Sedum lineare	Crassulaceae	Angiospermae	
Sedum major	Crassulaceae	Angiospermae	AM
Sedum odontophyllum	Crassulaceae	Angiospermae	
Sedum sarmentosum	Crassulaceae	Angiospermae	AM
Sinocrassula indica	Crassulaceae	Angiospermae	
Arabis flagellosa	Cruciferae	Angiospermae	NM
Armoracia rusticana	Cruciferae	Angiospermae	AM+NM
Brassica campestris	Cruciferae	Angiospermae	AM
Brassica juncea	Cruciferae	Angiospermae	AM+NM
Brassica juncea var. gracilis	Cruciferae	Angiospermae	AM+NM
Capsella bursa-pastoris	Cruciferae	Angiospermae	AM+NM
Cardamine flexuosa	Cruciferae	Angiospermae	AM+NM
Cardamine griffithii	Cruciferae	Angiospermae	ECM
Cardamine hirsuta	Cruciferae	Angiospermae	AM+NM
Cardamine impatiens	Cruciferae	Angiospermae	AM+NM
Cardamine leucantha	Cruciferae	Angiospermae	
Cardamine lyrata	Cruciferae	Angiospermae	
Cardamine macrophylla	Cruciferae	Angiospermae	ECM
Cardamine macrophylla var. polyphylla	Cruciferae	Angiospermae	ECM
Cardamine scaposa	Cruciferae	Angiospermae	
Cardamine tangutorum	Cruciferae	Angiospermae	
Cardamine urbaniana	Cruciferae	Angiospermae	
Cheiranthus roseus	Cruciferae	Angiospermae	
Cochlearia officinalis	Cruciferae	Angiospermae	AM+ECM+NM
Descurainia sophia	Cruciferae	Angiospermae	NM
Draba oreades	Cruciferae	Angiospermae	NM
Eruca sativa	Cruciferae	Angiospermae	NM
Erysimum cheiranthoides	Cruciferae	Angiospermae	NM

Isatis indigotica	Cruciferae	Angiospermae	
Isatis tinctoria	Cruciferae	Angiospermae	AM+NM
Lepidium apetalum	Cruciferae	Angiospermae	AM+NM
Lepidium latifolium	Cruciferae	Angiospermae	NM
Lepidium latifolium var. affine	Cruciferae	Angiospermae	NM
Lepidium sativum	Cruciferae	Angiospermae	AM+NM
Lepidium virginicum	Cruciferae	Angiospermae	AM+NM
Megacarpaea delavayi	Cruciferae	Angiospermae	
Nasturtium officinale	Cruciferae	Angiospermae	NM
Pegaeophyton scapiflorum	Cruciferae	Angiospermae	
Pugionium cornutum	Cruciferae	Angiospermae	
Raphanus sativus	Cruciferae	Angiospermae	AM+NM
Raphanus sativus var. longipinnatus	Cruciferae	Angiospermae	AM+NM
Rorippa dubia	Cruciferae	Angiospermae	
Rorippa indica	Cruciferae	Angiospermae	AM+NM
Sinapis alba	Cruciferae	Angiospermae	AM+NM
Solms-Laubachia dolichocarpa	Cruciferae	Angiospermae	
Solms-Laubachia eurycarpa	Cruciferae	Angiospermae	
Solms-Laubachia linearifolia	Cruciferae	Angiospermae	
Thlaspi arvense	Cruciferae	Angiospermae	AM+NM
Actinostemma tenerum	Cucurbitaceae	Angiospermae	AM
Benincasa hispida	Cucurbitaceae	Angiospermae	AM
Bolbostemma paniculatum	Cucurbitaceae	Angiospermae	
Citrullus lanatus	Cucurbitaceae	Angiospermae	AM
Cucumis melo	Cucurbitaceae	Angiospermae	AM+NM
Cucumis sativus	Cucurbitaceae	Angiospermae	AM
Cucurbita moschata	Cucurbitaceae	Angiospermae	AM
Gynostemma pentaphyllum	Cucurbitaceae	Angiospermae	AM
Hemsleya amabilis	Cucurbitaceae	Angiospermae	
Hemsleya graciliflora	Cucurbitaceae	Angiospermae	
Lagenaria siceraria	Cucurbitaceae	Angiospermae	AM
Lagenaria siceraria var. microcarpa	Cucurbitaceae	Angiospermae	AM
Luffa acutangula	Cucurbitaceae	Angiospermae	

Luffa cylindrica	Cucurbitaceae	Angiospermae	AM
Momordica charantia	Cucurbitaceae	Angiospermae	AM+NM
Momordica cochinchinensis	Cucurbitaceae	Angiospermae	
Siraitia grosvenorii	Cucurbitaceae	Angiospermae	AM
Solena amplexicaulis	Cucurbitaceae	Angiospermae	
Thladiantha hookeri	Cucurbitaceae	Angiospermae	
Thladiantha hookeri var. pentadactyla	Cucurbitaceae	Angiospermae	
Thladiantha sessilifolia	Cucurbitaceae	Angiospermae	
Trichosanthes anguina	Cucurbitaceae	Angiospermae	
Trichosanthes cucumerina	Cucurbitaceae	Angiospermae	AM
Trichosanthes jinggangshanica	Cucurbitaceae	Angiospermae	
Trichosanthes rosthornii	Cucurbitaceae	Angiospermae	
Trichosanthes rosthornii var. multicirrata	Cucurbitaceae	Angiospermae	
Zehneria indica	Cucurbitaceae	Angiospermae	
Cynomorium songaricum	Cynomoriaceae	Angiospermae	
Cyperus rotundus	Cyperaceae	Angiospermae	AM+NM
Heleocharis dulcis	Cyperaceae	Angiospermae	
Scirpus yagara	Cyperaceae	Angiospermae	
Daphniphyllum calycinum	Daphniphyllaceae	Angiospermae	
Dillenia pentagyna	Dilleniaceae	Angiospermae	
Dioscorea alata	Dioscoreaceae	Angiospermae	AM
Dioscorea althaeoides	Dioscoreaceae	Angiospermae	
Dioscorea bulbifera	Dioscoreaceae	Angiospermae	AM
Dioscorea chingii	Dioscoreaceae	Angiospermae	
Dioscorea cirrhosa	Dioscoreaceae	Angiospermae	
Dioscorea deltoidea	Dioscoreaceae	Angiospermae	AM
Dioscorea esquirolii	Dioscoreaceae	Angiospermae	
Dioscorea futschauensis	Dioscoreaceae	Angiospermae	
Dioscorea glabra	Dioscoreaceae	Angiospermae	
Dioscorea gracillima	Dioscoreaceae	Angiospermae	
Dioscorea hispida	Dioscoreaceae	Angiospermae	
Dioscorea japonica	Dioscoreaceae	Angiospermae	AM
Dioscorea melanophyma	Dioscoreaceae	Angiospermae	

Dioscorea nipponica	Dioscoreaceae	Angiospermae	ORM
Dioscorea opposita	Dioscoreaceae	Angiospermae	AM
Dioscorea panthaica	Dioscoreaceae	Angiospermae	
Dioscorea parviflora	Dioscoreaceae	Angiospermae	
Dioscorea septemloba	Dioscoreaceae	Angiospermae	
Dioscorea subcalva	Dioscoreaceae	Angiospermae	
Dioscorea tokoro	Dioscoreaceae	Angiospermae	AM
Dioscorea zingiberensis	Dioscoreaceae	Angiospermae	ORM
Ottelia alismoides	Dioscoreaceae	Angiospermae	AM+NM
Dipsacus asperoides	Dipsacaceae	Angiospermae	AM
Dipsacus atropurpureus	Dipsacaceae	Angiospermae	
Dipsacus japonicus	Dipsacaceae	Angiospermae	AM
Morina chinensis	Dipsacaceae	Angiospermae	
Pterocephalus hookeri	Dipsacaceae	Angiospermae	
Triplostegia grandiflora	Dipsacaceae	Angiospermae	
Dipterocarpus retusus	Dipterocarpaceae	Angiospermae	AM
Dipterocarpus turbinatus	Dipterocarpaceae	Angiospermae	AM+ECM
Drosera burmanni	Droseraceae	Angiospermae	
Drosera peltata	Droseraceae	Angiospermae	NM
Drosera peltata var. multisepala	Droseraceae	Angiospermae	NM
Drosera rotundifolia	Droseraceae	Angiospermae	AM+NM
Diospyros cathayensis	Ebenaceae	Angiospermae	AM
Diospyros glaucifolia	Ebenaceae	Angiospermae	
Diospyros kaki	Ebenaceae	Angiospermae	AM+NM
Diospyros lotus	Ebenaceae	Angiospermae	AM
Diospyros maritima	Ebenaceae	Angiospermae	AM
Diospyros morrisiana	Ebenaceae	Angiospermae	AM
Diospyros oleifera	Ebenaceae	Angiospermae	
Diospyros siderophylla	Ebenaceae	Angiospermae	
Elaeagnus angustifolia	Elaeagnaceae	Angiospermae	AM+NM
Elaeagnus bockii	Elaeagnaceae	Angiospermae	
Elaeagnus glabra	Elaeagnaceae	Angiospermae	AM+ECM
Elaeagnus gonyanthes	Elaeagnaceae	Angiospermae	

Elaeagnus henryi	Elaeagnaceae	Angiospermae	
Elaeagnus lanceolata	Elaeagnaceae	Angiospermae	
Elaeagnus mollis	Elaeagnaceae	Angiospermae	AM
Elaeagnus multiflora	Elaeagnaceae	Angiospermae	
Elaeagnus multiflora var. tenuipes	Elaeagnaceae	Angiospermae	
Elaeagnus oxycarpa	Elaeagnaceae	Angiospermae	
Elaeagnus pungens	Elaeagnaceae	Angiospermae	AM
Elaeagnus stellipila	Elaeagnaceae	Angiospermae	
Elaeagnus umbellata	Elaeagnaceae	Angiospermae	AM
Elaeagnus yunnanensis	Elaeagnaceae	Angiospermae	
Hippophae thibetana	Elaeagnaceae	Angiospermae	AM
Elaeocarpus prunifolioides	Elaeocarpaceae	Angiospermae	AM
Empetrum nigrum var. japonicum	Empetraceae	Angiospermae	AM+ERM+NM
Agapetes brandisiana	Ericaceae	Angiospermae	
Agapetes mannii	Ericaceae	Angiospermae	
Cassiope fastigiata	Ericaceae	Angiospermae	ECM
Craibiodendron stellatum	Ericaceae	Angiospermae	
Craibiodendron yunnanense	Ericaceae	Angiospermae	
Gaultheria forrestii	Ericaceae	Angiospermae	AM
Gaultheria leucocarpa	Ericaceae	Angiospermae	
Gaultheria leucocarpa var. crenulata	Ericaceae	Angiospermae	
Gaultheria leucocarpa var. hirsuta	Ericaceae	Angiospermae	
Rhododendron anthopogon	Ericaceae	Angiospermae	
Rhododendron anthopogonoides	Ericaceae	Angiospermae	
Rhododendron decorum	Ericaceae	Angiospermae	ERM
Rhododendron molle	Ericaceae	Angiospermae	ERM
Rhododendron naamkwanense var. naamkwanense	Ericaceae	Angiospermae	
Rhododendron ovatum	Ericaceae	Angiospermae	AM
Rhododendron simsii	Ericaceae	Angiospermae	AM
Sect. Ponticum	Ericaceae	Angiospermae	
Vaccinium dunalianum	Ericaceae	Angiospermae	
Vaccinium fragile	Ericaceae	Angiospermae	
Vaccinium vitis-idaea	Ericaceae	Angiospermae	AM+ERM+NM

Eriocaulon acutibracteatum	Eriocaulaceae	Angiospermae	
Eriocaulon buergerianum	Eriocaulaceae	Angiospermae	
Eriocaulon sexangulare	Eriocaulaceae	Angiospermae	
Erythroxylum novogranatense	Erythroxylaceae	Angiospermae	
Eucommia ulmoides	Eucommiaceae	Angiospermae	AM+ECM
Alchornea trewioides	Euphorbiaceae	Angiospermae	AM
Antidesma bunius	Euphorbiaceae	Angiospermae	AM
Antidesma ghaesembilla	Euphorbiaceae	Angiospermae	AM+ECM
Bischofia javanica	Euphorbiaceae	Angiospermae	AM
Breynia fruticosa	Euphorbiaceae	Angiospermae	AM
Breynia retusa	Euphorbiaceae	Angiospermae	
Breynia rostrata	Euphorbiaceae	Angiospermae	
Breynia vitis-idaea	Euphorbiaceae	Angiospermae	NM
Bridelia stipularis	Euphorbiaceae	Angiospermae	
Bridelia tomentosa	Euphorbiaceae	Angiospermae	
Claoxylon indicum	Euphorbiaceae	Angiospermae	
Croton crassifolius	Euphorbiaceae	Angiospermae	
Croton tiglium	Euphorbiaceae	Angiospermae	AM
Euphorbia antiquorum	Euphorbiaceae	Angiospermae	AM
Euphorbia esula	Euphorbiaceae	Angiospermae	AM
Euphorbia fischeriana	Euphorbiaceae	Angiospermae	
Euphorbia griffithii	Euphorbiaceae	Angiospermae	
Euphorbia helioscopia	Euphorbiaceae	Angiospermae	AM+NM
Euphorbia heyneana	Euphorbiaceae	Angiospermae	
Euphorbia humifusa	Euphorbiaceae	Angiospermae	AM+NM
Euphorbia hypericifolia	Euphorbiaceae	Angiospermae	AM
Euphorbia jolkinii	Euphorbiaceae	Angiospermae	ORM
Euphorbia kansuensis	Euphorbiaceae	Angiospermae	
Euphorbia kansui	Euphorbiaceae	Angiospermae	
Euphorbia lathylris	Euphorbiaceae	Angiospermae	AM
Euphorbia milii	Euphorbiaceae	Angiospermae	AM
Euphorbia milii var. tananarivae	Euphorbiaceae	Angiospermae	AM
Euphorbia pekinensis	Euphorbiaceae	Angiospermae	AM

Euphorbia peplus	Euphorbiaceae	Angiospermae	AM+NM
Euphorbia prolifera	Euphorbiaceae	Angiospermae	
Euphorbia pulcherrima	Euphorbiaceae	Angiospermae	NM
Euphorbia royleana	Euphorbiaceae	Angiospermae	AM
Euphorbia sieboldiana	Euphorbiaceae	Angiospermae	
Euphorbia sikkimensis	Euphorbiaceae	Angiospermae	
Euphorbia soongarica	Euphorbiaceae	Angiospermae	
Euphorbia thymifolia	Euphorbiaceae	Angiospermae	AM+NM
Excoecaria venenata	Euphorbiaceae	Angiospermae	
Flueggea suffruticosa	Euphorbiaceae	Angiospermae	AM
Flueggea virosa	Euphorbiaceae	Angiospermae	AM
Glochidion eriocarpum	Euphorbiaceae	Angiospermae	AM
Glochidion hirsutum	Euphorbiaceae	Angiospermae	
Glochidion puberum	Euphorbiaceae	Angiospermae	AM
Glochidion ramiflorum	Euphorbiaceae	Angiospermae	
Glochidion sphaerogynum	Euphorbiaceae	Angiospermae	
Glochidion zeylanicum	Euphorbiaceae	Angiospermae	AM
Jatropha curcas	Euphorbiaceae	Angiospermae	AM
Leptopus pachyphyllus	Euphorbiaceae	Angiospermae	
Phyllanthus bodinieri	Euphorbiaceae	Angiospermae	
Phyllanthus emblica	Euphorbiaceae	Angiospermae	AM+NM
Phyllanthus glaucus	Euphorbiaceae	Angiospermae	
Phyllanthus niruri	Euphorbiaceae	Angiospermae	AM+NM
Phyllanthus reticulatus	Euphorbiaceae	Angiospermae	AM+NM
Phyllanthus tsarongensis	Euphorbiaceae	Angiospermae	
Phyllanthus urinaria	Euphorbiaceae	Angiospermae	AM
Phyllanthus ussuriensis	Euphorbiaceae	Angiospermae	
Phyllanthus virgatus	Euphorbiaceae	Angiospermae	AM
Ricinus communis	Euphorbiaceae	Angiospermae	AM+NM
Sapium discolor	Euphorbiaceae	Angiospermae	
Sapium sebiferum	Euphorbiaceae	Angiospermae	AM
Sauropus spatulifolius	Euphorbiaceae	Angiospermae	
Trigonella foenum-graecum L.	Fabaceae	Angiospermae	

Quercus dentata	Fagaceae	Angiospermae	ECM+NM
Quercus mongolica	Fagaceae	Angiospermae	ECM+NM
Quercus mongolica var. grosseserrata	Fagaceae	Angiospermae	ECM+NM
Hydnocarpus anthelminthica	Flacourtiaceae	Angiospermae	
Xylosma racemosum	Flacourtiaceae	Angiospermae	
Flagellaria indica	Flagellariaceae	Angiospermae	AM
Gentiana apiata	Gentianaceae	Angiospermae	
Gentiana officinalis	Gentianaceae	Angiospermae	
Gentiana phyllopoda	Gentianaceae	Angiospermae	
Gentiana rhodantha	Gentianaceae	Angiospermae	
Gentiana triflora	Gentianaceae	Angiospermae	
Halenia corniculata	Gentianaceae	Angiospermae	NM
Halenia elliptica	Gentianaceae	Angiospermae	
Swertia diluta var. tosaensis	Gentianaceae	Angiospermae	
Swertia mileensis	Gentianaceae	Angiospermae	
Swertia mussotii var. flavescens	Gentianaceae	Angiospermae	
Swertia pseudochinensis	Gentianaceae	Angiospermae	
Veratrilla baillonii	Gentianaceae	Angiospermae	
Erodium stephanianum	Geraniaceae	Angiospermae	AM
Geranium carolinianum	Geraniaceae	Angiospermae	AM
Geranium delavayi	Geraniaceae	Angiospermae	
Geranium nepalense	Geraniaceae	Angiospermae	AM
Geranium pylzowianum	Geraniaceae	Angiospermae	
Geranium sinense	Geraniaceae	Angiospermae	
Geranium strictipes	Geraniaceae	Angiospermae	
Geranium wilfordii	Geraniaceae	Angiospermae	AM
Aeschynanthus acuminatus	Gesneriaceae	Angiospermae	
Aeschynanthus austroyunnanensis var. guangxiensis	Gesneriaceae	Angiospermae	
Boea clarkeana	Gesneriaceae	Angiospermae	
Boea hygrometrica	Gesneriaceae	Angiospermae	AM
Briggsia mihieri	Gesneriaceae	Angiospermae	
Chirita eburnea	Gesneriaceae	Angiospermae	
Chirita fimbrisepala	Gesneriaceae	Angiospermae	

Chirita gueilinensis	Gesneriaceae	Angiospermae	
Chirita hamosa	Gesneriaceae	Angiospermae	
Chirita linearifolia	Gesneriaceae	Angiospermae	
Chirita longgangensis	Gesneriaceae	Angiospermae	
Chirita longgangensis var. hongyao	Gesneriaceae	Angiospermae	
Chirita medica	Gesneriaceae	Angiospermae	
Chirita ophiopogoides	Gesneriaceae	Angiospermae	
Chirita parvifolia	Gesneriaceae	Angiospermae	
Chirita pinnatifida	Gesneriaceae	Angiospermae	
Chirita spinulosa	Gesneriaceae	Angiospermae	
Chiritopsis bipinnatifida	Gesneriaceae	Angiospermae	
Chiritopsis cordifolia	Gesneriaceae	Angiospermae	
Conandron ramondioides	Gesneriaceae	Angiospermae	AM
Corallodiscus cordatulus	Gesneriaceae	Angiospermae	
Corallodiscus flabellatus	Gesneriaceae	Angiospermae	AM
Corallodiscus kingianus	Gesneriaceae	Angiospermae	
Didissandra macrosiphon	Gesneriaceae	Angiospermae	
Didissandra sesquifolia	Gesneriaceae	Angiospermae	
Hemiboea cavaleriei	Gesneriaceae	Angiospermae	
Hemiboea follicularis	Gesneriaceae	Angiospermae	
Hemiboea henryi	Gesneriaceae	Angiospermae	
Hemiboea omeiensis	Gesneriaceae	Angiospermae	
Hemiboea subacaulis	Gesneriaceae	Angiospermae	
Hemiboea subcapitata	Gesneriaceae	Angiospermae	
Hemiboea subcapitata var. denticulata	Gesneriaceae	Angiospermae	
Lysionotus aeschynanthoides	Gesneriaceae	Angiospermae	
Lysionotus pauciflorus	Gesneriaceae	Angiospermae	AM
Lysionotus pauciflorus var. latifolius	Gesneriaceae	Angiospermae	AM
Oreocharis benthamii	Gesneriaceae	Angiospermae	
Oreocharis benthamii var. reticulata	Gesneriaceae	Angiospermae	
Ornithoboea wildeana	Gesneriaceae	Angiospermae	
Paraboea dictyoneura	Gesneriaceae	Angiospermae	AM
Paraboea rufescens	Gesneriaceae	Angiospermae	

Petrocodon dealbatus	Gesneriaceae	Angiospermae	
Rhynchoglossum obliquum var. hologlossum	Gesneriaceae	Angiospermae	
Apluda mutica	Gramineae	Angiospermae	AM+NM
Bambusa pervariabilis	Gramineae	Angiospermae	AM
Bambusa pervariabilis × textilis	Gramineae	Angiospermae	AM
Bambusa textilis	Gramineae	Angiospermae	AM
Bambusa textilis var. persistens	Gramineae	Angiospermae	AM
Coix chinensis	Gramineae	Angiospermae	
Cymbopogon citratus	Gramineae	Angiospermae	AM+NM
Cymbopogon flexuosus	Gramineae	Angiospermae	NM
Cymbopogon jwarancusa	Gramineae	Angiospermae	AM
Cymbopogon nardus	Gramineae	Angiospermae	AM
Dendrocalamopsis oldhami	Gramineae	Angiospermae	
Diandranthus aristatus	Gramineae	Angiospermae	
Eleusine indica	Gramineae	Angiospermae	AM+NM
Eragrostis ferruginea	Gramineae	Angiospermae	AM
Eragrostis pilosa	Gramineae	Angiospermae	AM+NM
Eragrostis tenella	Gramineae	Angiospermae	AM
Hierochloe odorata var. pubescens	Gramineae	Angiospermae	AM+NM
Imperata koenigii	Gramineae	Angiospermae	
Lophatherum gracile	Gramineae	Angiospermae	AM+NM
Lophatherum sinense	Gramineae	Angiospermae	
Miscanthus floridulus	Gramineae	Angiospermae	AM
Oligostachyum sulcatum	Gramineae	Angiospermae	
Phragmites australis	Gramineae	Angiospermae	AM+NM
Phyllostachys nigra var. henonis	Gramineae	Angiospermae	AM
Pogonatherum crinitum	Gramineae	Angiospermae	AM+NM
Saccharum officinarum	Gramineae	Angiospermae	AM
Saccharum sinense	Gramineae	Angiospermae	
Schizostachyum dumetorum	Gramineae	Angiospermae	
Setaria geniculata	Gramineae	Angiospermae	AM
Setaria italica	Gramineae	Angiospermae	AM
Setaria palmifolia	Gramineae	Angiospermae	

Setaria viridis	Gramineae	Angiospermae	AM+NM
Sinobambusa seminuda	Gramineae	Angiospermae	
Calophyllum inophyllum	Guttiferae	Angiospermae	AM
Calophyllum membranaceum	Guttiferae	Angiospermae	NM
Cratoxylum cochinchinense	Guttiferae	Angiospermae	AM
Cratoxylum formosum subsp. pruniflorum	Guttiferae	Angiospermae	AM
Garcinia multiflora	Guttiferae	Angiospermae	AM
Garcinia xanthochymus	Guttiferae	Angiospermae	AM+NM
Hypericum ascyron	Guttiferae	Angiospermae	
Hypericum attenuatum	Guttiferae	Angiospermae	AM
Hypericum japonicum	Guttiferae	Angiospermae	AM
Hypericum longistylum	Guttiferae	Angiospermae	
Hypericum monogynum	Guttiferae	Angiospermae	AM+NM
Hypericum patulum	Guttiferae	Angiospermae	
Hypericum seniavinii	Guttiferae	Angiospermae	
Haloragis micrantha	Haloragidaceae	Angiospermae	
Myriophyllum spicatum	Haloragidaceae	Angiospermae	NM
Altingia chinensis	Hamamelidaceae	Angiospermae	
Altingia gracilipes	Hamamelidaceae	Angiospermae	
Altingia gracilipes var. serrulata	Hamamelidaceae	Angiospermae	
Liquidambar formosana	Hamamelidaceae	Angiospermae	AM+ECM+NM
Loropetalum chinense	Hamamelidaceae	Angiospermae	ECM+NM
Semiliquidambar cathayensis	Hamamelidaceae	Angiospermae	
Illigera cordata	Hernandiaceae	Angiospermae	
Illigera cordata var. mollissima	Hernandiaceae	Angiospermae	
Illigera grandiflora	Hernandiaceae	Angiospermae	
Illigera parviflora	Hernandiaceae	Angiospermae	
Aesculus chinensis	Hippocastanaceae	Angiospermae	AM
Aesculus wilsonii	Hippocastanaceae	Angiospermae	
Salacia prinoides	Hippocrateaceae	Angiospermae	AM
Iodes balansae	Icacinaceae	Angiospermae	
Iodes cirrhosa	Icacinaceae	Angiospermae	
Mappianthus iodoides	Icacinaceae	Angiospermae	

Pittosporopsis kerrii	Icacinaceae	Angiospermae	AM
Belamcanda chinensis	Iridaceae	Angiospermae	AM
Crocosmia crocosmiflora	Iridaceae	Angiospermae	
Crocus alatavicus	Iridaceae	Angiospermae	
Crocus sativus	Iridaceae	Angiospermae	AM
Eleutherine plicata	Iridaceae	Angiospermae	
Iris anguifuga	Iridaceae	Angiospermae	
Iris confusa	Iridaceae	Angiospermae	
Iris decora	Iridaceae	Angiospermae	
Iris dichotoma	Iridaceae	Angiospermae	
Iris japonica	Iridaceae	Angiospermae	
Iris lactea var. chinensis	Iridaceae	Angiospermae	AM
Iris leptophylla	Iridaceae	Angiospermae	
Iris pallida	Iridaceae	Angiospermae	
Iris tectorum	Iridaceae	Angiospermae	AM
Engelhardtia roxbughiana	Juglandaceae	Angiospermae	
Engelhardtia roxburghiana	Juglandaceae	Angiospermae	ECM+NM
Platycarya strobilacea	Juglandaceae	Angiospermae	AM+ECM+NM
Juncus effusus	Juncaceae	Angiospermae	AM+NM
Juncus miyiensis	Juncaceae	Angiospermae	
Agastache rugosa	Labiatae	Angiospermae	ORM
Ajuga bracteosa	Labiatae	Angiospermae	
Ajuga campylantha	Labiatae	Angiospermae	
Ajuga ciliata	Labiatae	Angiospermae	
Ajuga decumbens	Labiatae	Angiospermae	AM
Ajuga forrestii	Labiatae	Angiospermae	
Ajuga macrosperma	Labiatae	Angiospermae	
Ajuga multiflora	Labiatae	Angiospermae	
Ajuga nipponensis	Labiatae	Angiospermae	
Ajuga pantantha	Labiatae	Angiospermae	
Amethystea caerulea	Labiatae	Angiospermae	AM
Anisochilus carnosus	Labiatae	Angiospermae	
Clerodendranthus spicatus	Labiatae	Angiospermae	

Clinopodium megalanthum	Labiatae	Angiospermae	
Clinopodium polycephalum	Labiatae	Angiospermae	
Colquhounia coccinea var. mollis	Labiatae	Angiospermae	
Colquhounia elegans	Labiatae	Angiospermae	
Colquhounia elegans var. tenuiflora	Labiatae	Angiospermae	
Comanthosphace ningpoensis	Labiatae	Angiospermae	NM
Dracocephalum heterophyllum	Labiatae	Angiospermae	AM
Dracocephalum integrifolium	Labiatae	Angiospermae	AM
Dracocephalum tanguticum	Labiatae	Angiospermae	
Elsholtzia blanda	Labiatae	Angiospermae	AM
Elsholtzia bodinieri	Labiatae	Angiospermae	
Elsholtzia ciliata	Labiatae	Angiospermae	AM
Elsholtzia communis	Labiatae	Angiospermae	AM
Elsholtzia cypriani	Labiatae	Angiospermae	AM
Elsholtzia densa	Labiatae	Angiospermae	AM
Elsholtzia feddei f. remotibracteata	Labiatae	Angiospermae	
Elsholtzia flava	Labiatae	Angiospermae	
Elsholtzia fruticosa var. fruticosa f. leptostachya	Labiatae	Angiospermae	
Elsholtzia heterophylla	Labiatae	Angiospermae	
Elsholtzia penduliflora	Labiatae	Angiospermae	
Elsholtzia rugulosa	Labiatae	Angiospermae	AM
Elsholtzia souliei	Labiatae	Angiospermae	
Elsholtzia splendens	Labiatae	Angiospermae	
Elsholtzia strobilifera	Labiatae	Angiospermae	AM
Epimeredi indica	Labiatae	Angiospermae	
Eriophyton wallichii	Labiatae	Angiospermae	
Glechoma biondiana	Labiatae	Angiospermae	
Glechoma biondiana var. glabrescens	Labiatae	Angiospermae	
Glechoma hederacea	Labiatae	Angiospermae	AM+NM
Glechoma longituba	Labiatae	Angiospermae	ECM
Glechoma sinograndis	Labiatae	Angiospermae	
Gomphostemma leptodon	Labiatae	Angiospermae	
Gomphostemma microdon	Labiatae	Angiospermae	AM

Heterolamium debile var. cardiophyllum	Labiatae	Angiospermae	
Hyptis suaveolens	Labiatae	Angiospermae	AM
Lagopsis supina	Labiatae	Angiospermae	
Lamiophlomis rotata	Labiatae	Angiospermae	AM
Lamium album	Labiatae	Angiospermae	AM+NM
Lamium barbatum	Labiatae	Angiospermae	AM
Leonurus artemisia var. albiflorus	Labiatae	Angiospermae	AM
Leonurus artemisia var. artemisia	Labiatae	Angiospermae	AM
Leonurus pseudomacranthus	Labiatae	Angiospermae	
Leonurus pseudomacranthus f. leucanthus	Labiatae	Angiospermae	
Leucas ciliata	Labiatae	Angiospermae	
Leucas martinicensis	Labiatae	Angiospermae	NM
Leucas mollissima	Labiatae	Angiospermae	
Leucas mollissima var. chinensis	Labiatae	Angiospermae	
Leucas zeylanica	Labiatae	Angiospermae	
Lycopus lucidus var. hirtus	Labiatae	Angiospermae	
Marrubium vulgare	Labiatae	Angiospermae	AM
Meehania fargesii	Labiatae	Angiospermae	
Meehania fargesii var. pedunculata	Labiatae	Angiospermae	
Meehania fargesii var. radicans	Labiatae	Angiospermae	
Melissa axillaris	Labiatae	Angiospermae	
Melissa officinalis	Labiatae	Angiospermae	AM
Mentha haplocalyx	Labiatae	Angiospermae	AM
Mentha spicata	Labiatae	Angiospermae	AM+NM
Mesona chinensis	Labiatae	Angiospermae	
Micromeria biflora	Labiatae	Angiospermae	
Microtoena omeiensis	Labiatae	Angiospermae	
Microtoena patchoulii	Labiatae	Angiospermae	
Microtoena pauciflora	Labiatae	Angiospermae	
Mosla cavaleriei	Labiatae	Angiospermae	
Mosla chinensis	Labiatae	Angiospermae	
Mosla dianthera	Labiatae	Angiospermae	AM
Mosla scabra	Labiatae	Angiospermae	AM

Nepeta angustifolia	Labiatae	Angiospermae	
Nepeta cataria	Labiatae	Angiospermae	AM
Nepeta laevigata	Labiatae	Angiospermae	
Nosema cochinchinensis	Labiatae	Angiospermae	
Ocimum americanum	Labiatae	Angiospermae	AM+NM
Ocimum basilicum	Labiatae	Angiospermae	AM+NM
Ocimum basilicum var. pilosum	Labiatae	Angiospermae	AM+NM
Ocimum gratissimum var. suave	Labiatae	Angiospermae	
Ocimum sanctum	Labiatae	Angiospermae	AM
Origanum vulgare	Labiatae	Angiospermae	AM
Orthosiphon wulfenioides	Labiatae	Angiospermae	
Orthosiphon wulfenioides var. foliosus	Labiatae	Angiospermae	
Panzeria alaschanica	Labiatae	Angiospermae	AM
Paraphlomis javanica var. coronata	Labiatae	Angiospermae	AM
Perilla frutescens	Labiatae	Angiospermae	AM
Phlomis maximowiczii	Labiatae	Angiospermae	
Phlomis mongolica	Labiatae	Angiospermae	
Phlomis umbrosa	Labiatae	Angiospermae	AM
Phlomis umbrosa var. australis	Labiatae	Angiospermae	AM
Phlomis younghusbandii	Labiatae	Angiospermae	
Pogostemon auricularius	Labiatae	Angiospermae	
Pogostemon cablin	Labiatae	Angiospermae	ORM
Pogostemon esquirolii	Labiatae	Angiospermae	
Pogostemon glaber	Labiatae	Angiospermae	
Pogostemon nigrescens	Labiatae	Angiospermae	
Prunella asiatica	Labiatae	Angiospermae	
Prunella vulgaris	Labiatae	Angiospermae	AM+NM
Rabdosia adenantha	Labiatae	Angiospermae	
Rabdosia amethystoides	Labiatae	Angiospermae	AM
Rabdosia coetsa	Labiatae	Angiospermae	
Rabdosia coetsoides	Labiatae	Angiospermae	
Rabdosia eriocalyx	Labiatae	Angiospermae	
Rabdosia hispida	Labiatae	Angiospermae	

Rabdosia japonica	Labiatae	Angiospermae	
Rabdosia lophanthoides	Labiatae	Angiospermae	
Rabdosia lophanthoides var. gerardiana	Labiatae	Angiospermae	
Rabdosia megathyrsa	Labiatae	Angiospermae	
Rabdosia nervosa	Labiatae	Angiospermae	
Rabdosia oresbia	Labiatae	Angiospermae	
Rabdosia rosthornii	Labiatae	Angiospermae	
Rabdosia rubescens	Labiatae	Angiospermae	
Rabdosia sculponeata	Labiatae	Angiospermae	AM
Rabdosia serra	Labiatae	Angiospermae	
Rabdosia ternifolia	Labiatae	Angiospermae	
Rabdosia yuennanensis	Labiatae	Angiospermae	
Salvia aerea	Labiatae	Angiospermae	
Salvia bowleyana	Labiatae	Angiospermae	
Salvia cavaleriei var. simplicifolia	Labiatae	Angiospermae	
Salvia chinensis	Labiatae	Angiospermae	AM
Salvia coccinea	Labiatae	Angiospermae	
Salvia digitaloides	Labiatae	Angiospermae	
Salvia evansiana	Labiatae	Angiospermae	
Salvia kiangsiensis	Labiatae	Angiospermae	
Salvia kiaometiensis	Labiatae	Angiospermae	
Salvia miltiorrhiza	Labiatae	Angiospermae	AM
Salvia officinalis	Labiatae	Angiospermae	AM
Salvia plebeia	Labiatae	Angiospermae	
Salvia plectranthoides	Labiatae	Angiospermae	
Salvia przewalskii	Labiatae	Angiospermae	AM
Salvia scapiformis	Labiatae	Angiospermae	
Salvia scapiformis var. hirsuta	Labiatae	Angiospermae	
Salvia substolonifera	Labiatae	Angiospermae	
Salvia trijuga	Labiatae	Angiospermae	
Salvia yunnanensis	Labiatae	Angiospermae	
Schizonepeta tenuifolia	Labiatae	Angiospermae	AM
Schnabelia oligophylla	Labiatae	Angiospermae	

Scutellaria amoena	Labiatae	Angiospermae	
Scutellaria baicalensis	Labiatae	Angiospermae	NM
Scutellaria barbata	Labiatae	Angiospermae	AM
Scutellaria caudifolia var. obliquifolia	Labiatae	Angiospermae	
Scutellaria discolor	Labiatae	Angiospermae	AM
Scutellaria discolor var. hirta	Labiatae	Angiospermae	AM
Scutellaria franchetiana	Labiatae	Angiospermae	
Scutellaria likiangensis	Labiatae	Angiospermae	
Scutellaria obtusifolia	Labiatae	Angiospermae	
Scutellaria omeiensis	Labiatae	Angiospermae	
Scutellaria orthocalyx	Labiatae	Angiospermae	
Scutellaria quadrilobulata	Labiatae	Angiospermae	
Scutellaria scordifolia	Labiatae	Angiospermae	AM
Scutellaria sessilifolia	Labiatae	Angiospermae	
Scutellaria tayloriana	Labiatae	Angiospermae	
Scutellaria yunnanensis	Labiatae	Angiospermae	
Scutellaria yunnanensis var. salicifolia	Labiatae	Angiospermae	
Siphocranion macranthum	Labiatae	Angiospermae	
Smilacina nokomonticola	Labiatae	Angiospermae	
Stachys geobombycis	Labiatae	Angiospermae	
Stachys japonica	Labiatae	Angiospermae	
Stachys kouyangensis	Labiatae	Angiospermae	
Stachys oblongifolia	Labiatae	Angiospermae	
Teucrium anlungense	Labiatae	Angiospermae	
Teucrium bidentatum	Labiatae	Angiospermae	
Teucrium japonicum	Labiatae	Angiospermae	AM
Teucrium pilosum	Labiatae	Angiospermae	
Teucrium quadrifarium	Labiatae	Angiospermae	AM
Teucrium scordioides	Labiatae	Angiospermae	
Teucrium scordium	Labiatae	Angiospermae	AM
Teucrium viscidum	Labiatae	Angiospermae	NM
Akebia quinata	Lardizabalaceae	Angiospermae	AM
Akebia trifoliata	Lardizabalaceae	Angiospermae	AM

Akebia trifoliata subsp. australis	Lardizabalaceae	Angiospermae	AM
Decaisnea insignis	Lardizabalaceae	Angiospermae	
Holboellia coriacea	Lardizabalaceae	Angiospermae	
Holboellia fargesii	Lardizabalaceae	Angiospermae	
Holboellia latifolia var. latifolia	Lardizabalaceae	Angiospermae	
Sargentodoxa cuneata	Lardizabalaceae	Angiospermae	
Stauntonia chinensis	Lardizabalaceae	Angiospermae	
Stauntonia yaoshanensis	Lardizabalaceae	Angiospermae	
Actinodaphne cupularis	Lauraceae	Angiospermae	
Actinodaphne obovata	Lauraceae	Angiospermae	
Actinodaphne pilosa	Lauraceae	Angiospermae	
Cassytha filiformis	Lauraceae	Angiospermae	
Cinnamomum appelianum	Lauraceae	Angiospermae	
Cinnamomum austrosinense	Lauraceae	Angiospermae	AM
Cinnamomum burmannii	Lauraceae	Angiospermae	AM
Cinnamomum camphora	Lauraceae	Angiospermae	AM+ECM
Cinnamomum camphora var. linaloolifera	Lauraceae	Angiospermae	AM+ECM
Cinnamomum cassia	Lauraceae	Angiospermae	AM
Cinnamomum glanduliferum	Lauraceae	Angiospermae	AM
Cinnamomum jensenianum	Lauraceae	Angiospermae	
Cinnamomum pauciflorum	Lauraceae	Angiospermae	
Cinnamomum porrectum	Lauraceae	Angiospermae	AM
Cinnamomum subavenium	Lauraceae	Angiospermae	AM
Cinnamomum tamala	Lauraceae	Angiospermae	AM
Cinnamomum wilsonii	Lauraceae	Angiospermae	
Cinnamomum zeylanicum	Lauraceae	Angiospermae	
Lindera aggregata	Lauraceae	Angiospermae	AM
Lindera aggregata var. playfairii	Lauraceae	Angiospermae	AM
Lindera chunii	Lauraceae	Angiospermae	NM
Lindera communis	Lauraceae	Angiospermae	AM
Lindera glauca	Lauraceae	Angiospermae	
Lindera obtusiloba	Lauraceae	Angiospermae	AM
Lindera pulcherrima var. attenuata	Lauraceae	Angiospermae	

Lindera reflexa	Lauraceae	Angiospermae	AM
Litsea auriculata	Lauraceae	Angiospermae	
Litsea coreana var. sinensis	Lauraceae	Angiospermae	AM+ECM
Litsea cubeba var. cubeba	Lauraceae	Angiospermae	AM
Litsea cubeba var. cubeba f. obtusifolia	Lauraceae	Angiospermae	AM
Litsea euosma	Lauraceae	Angiospermae	
Litsea glutinosa	Lauraceae	Angiospermae	AM
Litsea mollis	Lauraceae	Angiospermae	
Litsea monopetala	Lauraceae	Angiospermae	AM
Litsea rotundifolia var. oblongifolia	Lauraceae	Angiospermae	
Litsea verticillata	Lauraceae	Angiospermae	AM+NM
Machilus thunbergii	Lauraceae	Angiospermae	AM
Neocinnamomum delavayi	Lauraceae	Angiospermae	AM
Neolitsea aurata	Lauraceae	Angiospermae	AM+ECM
Neolitsea aurata var. chekiangensis	Lauraceae	Angiospermae	AM+ECM
Neolitsea cambodiana	Lauraceae	Angiospermae	AM
Neolitsea levinei	Lauraceae	Angiospermae	
Persea americana	Lauraceae	Angiospermae	AM+NM
Sassafras tzumu	Lauraceae	Angiospermae	AM
Barringtonia racemosa	Lecythidaceae	Angiospermae	AM
Abrus cantoniensis	Leguminosae	Angiospermae	
Abrus precatorius	Leguminosae	Angiospermae	AM
Abrus pulchellus	Leguminosae	Angiospermae	
Acacia catechu	Leguminosae	Angiospermae	
Acacia farnesiana	Leguminosae	Angiospermae	AM+NM
Acacia senegal	Leguminosae	Angiospermae	AM
Acacia sinuata	Leguminosae	Angiospermae	
Aeschynomene indica	Leguminosae	Angiospermae	AM
Afzelia xylocarpa	Leguminosae	Angiospermae	ECM
Albizia julibrissin	Leguminosae	Angiospermae	AM+ECM
Albizia julibrissin f. rosea	Leguminosae	Angiospermae	AM+ECM
Alysicarpus vaginalis	Leguminosae	Angiospermae	AM
Astragalus adsurgens	Leguminosae	Angiospermae	AM+NM

Astragalus bhotanensis	Leguminosae	Angiospermae	AM
Astragalus complanatus	Leguminosae	Angiospermae	
Astragalus ernestii	Leguminosae	Angiospermae	
Astragalus hoantchy	Leguminosae	Angiospermae	
Astragalus membranaceus var. mongholicus	Leguminosae	Angiospermae	AM
Astragalus scaberrimus	Leguminosae	Angiospermae	AM
Astragalus variabilis	Leguminosae	Angiospermae	
Bauhinia aurea	Leguminosae	Angiospermae	AM
Bauhinia purpurea	Leguminosae	Angiospermae	AM
Bauhinia tomentosa	Leguminosae	Angiospermae	NM
Bauhinia variegata	Leguminosae	Angiospermae	AM+NM
Butea monosperma	Leguminosae	Angiospermae	AM
Caesalpinia decapetala	Leguminosae	Angiospermae	AM+ECM
Caesalpinia digyna	Leguminosae	Angiospermae	
Caesalpinia minax	Leguminosae	Angiospermae	
Caesalpinia sappan	Leguminosae	Angiospermae	
Cajanus scarabaeoides	Leguminosae	Angiospermae	AM
Campylotropis bonatiana	Leguminosae	Angiospermae	
Campylotropis delavayi	Leguminosae	Angiospermae	AM
Campylotropis hirtella	Leguminosae	Angiospermae	
Campylotropis pinetorum	Leguminosae	Angiospermae	AM
Campylotropis pinetorum subsp. velutina	Leguminosae	Angiospermae	AM
Campylotropis polyantha	Leguminosae	Angiospermae	
Campylotropis trigonoclada	Leguminosae	Angiospermae	
Cassia agnes	Leguminosae	Angiospermae	
Cassia fistula	Leguminosae	Angiospermae	AM+ECM
Cassia mimosoides	Leguminosae	Angiospermae	AM+NM
Cassia occidentalis	Leguminosae	Angiospermae	AM
Cassia sophera	Leguminosae	Angiospermae	AM
Cercis chinensis	Leguminosae	Angiospermae	AM
Christia vespertilionis	Leguminosae	Angiospermae	AM
Clitoria hanceana	Leguminosae	Angiospermae	
Codariocalyx motorius	Leguminosae	Angiospermae	NM

Coronilla varia	Leguminosae	Angiospermae	AM+NM
Crotalaria alata	Leguminosae	Angiospermae	
Crotalaria albida	Leguminosae	Angiospermae	
Crotalaria assamica	Leguminosae	Angiospermae	AM
Crotalaria ferruginea	Leguminosae	Angiospermae	
Crotalaria juncea	Leguminosae	Angiospermae	AM
Crotalaria linifolia	Leguminosae	Angiospermae	AM
Crotalaria mairei	Leguminosae	Angiospermae	
Crotalaria medicaginea	Leguminosae	Angiospermae	AM
Crotalaria micans	Leguminosae	Angiospermae	
Crotalaria retusa	Leguminosae	Angiospermae	AM
Crotalaria sessiliflora	Leguminosae	Angiospermae	
Crotalaria spectabilis	Leguminosae	Angiospermae	AM
Crotalaria verrucosa	Leguminosae	Angiospermae	AM
Crotalaria zanzibarica	Leguminosae	Angiospermae	
Dalbergia hancei	Leguminosae	Angiospermae	
Dalbergia hupeana	Leguminosae	Angiospermae	AM+ECM
Dalbergia odorifera	Leguminosae	Angiospermae	AM
Dalbergia pinnata	Leguminosae	Angiospermae	AM
Dendrolobium triangulare	Leguminosae	Angiospermae	AM
Desmodium amethystinum	Leguminosae	Angiospermae	
Desmodium caudatum	Leguminosae	Angiospermae	
Desmodium heterocarpon	Leguminosae	Angiospermae	AM
Desmodium microphyllum	Leguminosae	Angiospermae	AM
Desmodium multiflorum	Leguminosae	Angiospermae	AM
Desmodium styracifolium	Leguminosae	Angiospermae	
Desmodium triflorum	Leguminosae	Angiospermae	AM
Dichrostachys cinerea	Leguminosae	Angiospermae	AM
Dumasia forrestii	Leguminosae	Angiospermae	
Eriosema chinense	Leguminosae	Angiospermae	
Eriosema himalaicum	Leguminosae	Angiospermae	
Erythrina caffra	Leguminosae	Angiospermae	
Erythrina corallodendron	Leguminosae	Angiospermae	

Erythrina senegalensis	Leguminosae	Angiospermae	
Erythrina speciosa	Leguminosae	Angiospermae	
Erythrina variegata	Leguminosae	Angiospermae	AM+NM
Euchresta horsfieldii	Leguminosae	Angiospermae	
Euchresta tubulosa	Leguminosae	Angiospermae	
Flemingia fluminalis	Leguminosae	Angiospermae	
Flemingia macrophylla	Leguminosae	Angiospermae	AM
Flemingia philippinensis	Leguminosae	Angiospermae	
Flemingia strobilifera	Leguminosae	Angiospermae	AM
Fordia cauliflora	Leguminosae	Angiospermae	
Galega officinalis	Leguminosae	Angiospermae	
Galega officinalis var. hartlandii	Leguminosae	Angiospermae	
Galega officinalis var. patus	Leguminosae	Angiospermae	
Galega officinalis var. persica	Leguminosae	Angiospermae	
Gleditsia fera	Leguminosae	Angiospermae	NM
Gleditsia japonica	Leguminosae	Angiospermae	AM
Gleditsia sinensis	Leguminosae	Angiospermae	AM
Glycine max	Leguminosae	Angiospermae	AM
Glycine soja	Leguminosae	Angiospermae	AM
Glycine soja var. albiflora f. angustifolia	Leguminosae	Angiospermae	AM
Glycyrrhiza glabra	Leguminosae	Angiospermae	AM
Glycyrrhiza inflata	Leguminosae	Angiospermae	AM
Glycyrrhiza uralensis	Leguminosae	Angiospermae	AM
Gueldenstaedtia verna subsp. multiflora	Leguminosae	Angiospermae	AM
Gueldenstaedtia verna subsp. multiflora f. multiflora	Leguminosae	Angiospermae	AM
Hedysarum polybotrys	Leguminosae	Angiospermae	
Indigofera amblyantha	Leguminosae	Angiospermae	AM
Indigofera bungeana	Leguminosae	Angiospermae	
Indigofera carlesii	Leguminosae	Angiospermae	
Indigofera decora var. cooperii	Leguminosae	Angiospermae	
Indigofera longispica	Leguminosae	Angiospermae	
Indigofera pseudotinctoria	Leguminosae	Angiospermae	AM
Indigofera squalida	Leguminosae	Angiospermae	

Indigofera suffruticosa	Leguminosae	Angiospermae	AM
Indigofera tinctoria	Leguminosae	Angiospermae	AM
Kummerowia stipulacea	Leguminosae	Angiospermae	
Kummerowia striata	Leguminosae	Angiospermae	AM
Lablab purpureus	Leguminosae	Angiospermae	AM
Lespedeza pilosa	Leguminosae	Angiospermae	AM
Lespedeza tomentosa	Leguminosae	Angiospermae	
Lysidice brevicalyx	Leguminosae	Angiospermae	
Lysidice rhodostegia	Leguminosae	Angiospermae	
Maackia amurensis	Leguminosae	Angiospermae	AM+NM
Maackia amurensis var. buergeri	Leguminosae	Angiospermae	AM+NM
Millettia bonatiana	Leguminosae	Angiospermae	
Millettia championii	Leguminosae	Angiospermae	
Millettia eurybotrya	Leguminosae	Angiospermae	
Millettia ichthyochtona	Leguminosae	Angiospermae	
Millettia nitida var. minor	Leguminosae	Angiospermae	
Millettia oosperma	Leguminosae	Angiospermae	
Millettia pachycarpa	Leguminosae	Angiospermae	
Millettia speciosa	Leguminosae	Angiospermae	
Millettia tsui	Leguminosae	Angiospermae	
Mimosa pudica	Leguminosae	Angiospermae	AM
Mucuna birdwoodiana	Leguminosae	Angiospermae	
Mucuna sempervirens	Leguminosae	Angiospermae	AM
Ormosia henryi	Leguminosae	Angiospermae	
Ormosia microphylla	Leguminosae	Angiospermae	
Oxytropis aciphylla var. aciphylla	Leguminosae	Angiospermae	AM
Oxytropis chiliophylla	Leguminosae	Angiospermae	
Oxytropis falcata	Leguminosae	Angiospermae	
Oxytropis microphylla	Leguminosae	Angiospermae	AM
Oxytropis racemosa	Leguminosae	Angiospermae	AM
Pachyrhizus erosus	Leguminosae	Angiospermae	AM
Parkinsonia aculeata	Leguminosae	Angiospermae	AM
Phyllodium elegans	Leguminosae	Angiospermae	

Phyllodium pulchellum	Leguminosae	Angiospermae	
Physostigma venenosum	Leguminosae	Angiospermae	
Pisum sativum	Leguminosae	Angiospermae	AM
Pithecellobium lucidum	Leguminosae	Angiospermae	
Podocarpium duclouxii	Leguminosae	Angiospermae	
Podocarpium oldhamii	Leguminosae	Angiospermae	
Podocarpium podocarpum var. fallax	Leguminosae	Angiospermae	
Podocarpium podocarpum var. oxyphyllum	Leguminosae	Angiospermae	
Podocarpium podocarpum var. szechuenense	Leguminosae	Angiospermae	
Psoralea corylifolia	Leguminosae	Angiospermae	AM
Pterocarpus indicus	Leguminosae	Angiospermae	AM
Pueraria lobata	Leguminosae	Angiospermae	AM
Rhynchosia dielsii	Leguminosae	Angiospermae	
Rhynchosia volubilis	Leguminosae	Angiospermae	AM
Saraca dives	Leguminosae	Angiospermae	AM
Sesbania grandiflora	Leguminosae	Angiospermae	AM+NM
Sesbania sesban	Leguminosae	Angiospermae	AM+NM
Shuteria involucrata	Leguminosae	Angiospermae	
Shuteria involucrata var. glabrata	Leguminosae	Angiospermae	
Smithia sensitiva	Leguminosae	Angiospermae	AM
Sophora alopecuroides	Leguminosae	Angiospermae	AM
Sophora flavescens	Leguminosae	Angiospermae	AM
Sophora flavescens var. favescens	Leguminosae	Angiospermae	AM
Sophora flavescens var. kronei	Leguminosae	Angiospermae	AM
Sophora japonica	Leguminosae	Angiospermae	AM
Sophora tonkinensis	Leguminosae	Angiospermae	ORM
Spatholobus suberectus	Leguminosae	Angiospermae	
Sphaerophysa salsula	Leguminosae	Angiospermae	AM
Tadehagi triquetrum	Leguminosae	Angiospermae	AM
Tamarindus indica	Leguminosae	Angiospermae	AM+NM
Thermopsis chinensis	Leguminosae	Angiospermae	AM
Thermopsis lanceolata	Leguminosae	Angiospermae	AM
Trifolium repens	Leguminosae	Angiospermae	AM

Trigonella foenum-graecum	Leguminosae	Angiospermae	AM
Uraria crinita	Leguminosae	Angiospermae	
Uraria lagopodioides	Leguminosae	Angiospermae	AM
Uraria picta	Leguminosae	Angiospermae	
Vicia amoena	Leguminosae	Angiospermae	AM
Vicia amurensis	Leguminosae	Angiospermae	
Vicia faba	Leguminosae	Angiospermae	AM
Vicia hirsuta	Leguminosae	Angiospermae	AM
Vicia kioshanica	Leguminosae	Angiospermae	
Vicia kulingiana	Leguminosae	Angiospermae	
Vicia pseudorobus	Leguminosae	Angiospermae	
Vicia sativa	Leguminosae	Angiospermae	AM
Vicia sepium	Leguminosae	Angiospermae	AM
Vicia tetrasperma	Leguminosae	Angiospermae	AM
Vicia unijuga	Leguminosae	Angiospermae	
Vigna radiata	Leguminosae	Angiospermae	AM
Vigna umbellata	Leguminosae	Angiospermae	AM
Vigna vexillata	Leguminosae	Angiospermae	AM
Zornia gibbosa	Leguminosae	Angiospermae	AM+NM
Lemna minor	Lemnaceae	Angiospermae	NM
Spirodela polyrrhiza	Lemnaceae	Angiospermae	AM+NM
Aletris spicata	Liliaceae	Angiospermae	AM
Allium fistulosum	Liliaceae	Angiospermae	AM
Allium macrostemon	Liliaceae	Angiospermae	AM
Aloe arborescens var. natalensis	Liliaceae	Angiospermae	AM
Aloe vera var. chinensis	Liliaceae	Angiospermae	AM+NM
Anemarrhena asphodeloides	Liliaceae	Angiospermae	ORM
Asparagus cochinchinensis	Liliaceae	Angiospermae	AM
Asparagus filicinus	Liliaceae	Angiospermae	
Asparagus lycopodineus	Liliaceae	Angiospermae	
Asparagus schoberioides	Liliaceae	Angiospermae	
Aspidistra attenuata	Liliaceae	Angiospermae	
Aspidistra daibuensis	Liliaceae	Angiospermae	

Aspidistra lurida	Liliaceae	Angiospermae	
Aspidistra mushaensis	Liliaceae	Angiospermae	
Cardiocrinum cathayanum	Liliaceae	Angiospermae	
Cardiocrinum giganteum	Liliaceae	Angiospermae	
Chlorophytum laxum	Liliaceae	Angiospermae	
Convallaria majalis	Liliaceae	Angiospermae	AM+NM
Cordyline fruticosa	Liliaceae	Angiospermae	NM
Dianella ensifolia	Liliaceae	Angiospermae	AM
Disporum cantoniense	Liliaceae	Angiospermae	
Disporum sessile	Liliaceae	Angiospermae	
Dracaena cochinchinensis	Liliaceae	Angiospermae	ORM
Fritillaria cirrhosa	Liliaceae	Angiospermae	AM
Fritillaria delavayi	Liliaceae	Angiospermae	
Fritillaria karelinii	Liliaceae	Angiospermae	AM
Fritillaria pallidiflora	Liliaceae	Angiospermae	
Fritillaria przewalskii	Liliaceae	Angiospermae	
Fritillaria taipaiensis	Liliaceae	Angiospermae	AM
Fritillaria thunbergii	Liliaceae	Angiospermae	ORM
Fritillaria thunbergii var. chekiangensis	Liliaceae	Angiospermae	AM+ORM
Fritillaria ussuriensis	Liliaceae	Angiospermae	ORM
Hemerocallis citrina	Liliaceae	Angiospermae	AM
Hosta plantaginea	Liliaceae	Angiospermae	AM
Hosta ventricosa	Liliaceae	Angiospermae	AM
Iphigenia indica	Liliaceae	Angiospermae	AM
Lilium brownii	Liliaceae	Angiospermae	AM
Lilium brownii var. viridulum	Liliaceae	Angiospermae	AM
Lilium concolor	Liliaceae	Angiospermae	
Lilium dauricum	Liliaceae	Angiospermae	
Lilium lancifolium	Liliaceae	Angiospermae	AM
Lilium pumilum	Liliaceae	Angiospermae	AM
Lilium rosthornii	Liliaceae	Angiospermae	
Lilium speciosum var. gloriosoides	Liliaceae	Angiospermae	AM
Lilium sulphureum	Liliaceae	Angiospermae	

Lloydia tibetica	Liliaceae	Angiospermae	
Notholirion macrophyllum	Liliaceae	Angiospermae	
Ophiopogon chingii	Liliaceae	Angiospermae	
Ophiopogon japonicus	Liliaceae	Angiospermae	AM+NM
Ophiopogon zingiberaceus	Liliaceae	Angiospermae	
Polygonatum odoratum	Liliaceae	Angiospermae	AM+NM
Polygonatum sibiricum	Liliaceae	Angiospermae	NM
Reineckia carnea	Liliaceae	Angiospermae	AM
Rohdea japonica	Liliaceae	Angiospermae	AM
Smilacina formosana	Liliaceae	Angiospermae	
Smilax glabra	Liliaceae	Angiospermae	AM
Smilax mairei	Liliaceae	Angiospermae	
Smilax nipponica	Liliaceae	Angiospermae	
Smilax riparia	Liliaceae	Angiospermae	
Smilax scobinicaulis	Liliaceae	Angiospermae	
Tulipa edulis	Liliaceae	Angiospermae	
Veratrum mengtzeanum	Liliaceae	Angiospermae	
Veratrum stenophyllum	Liliaceae	Angiospermae	
Veratrum taliense	Liliaceae	Angiospermae	
Linum usitatissimum	Linaceae	Angiospermae	AM+NM
Reinwardtia indica	Linaceae	Angiospermae	AM
Tirpitzia ovoidea	Linaceae	Angiospermae	AM
Tirpitzia sinensis	Linaceae	Angiospermae	
Buddleja asiatica	Loganiaceae	Angiospermae	AM
Buddleja caryopteridifolia	Loganiaceae	Angiospermae	
Buddleja davidii	Loganiaceae	Angiospermae	AM+NM
Buddleja fallowiana	Loganiaceae	Angiospermae	
Buddleja lindleyana	Loganiaceae	Angiospermae	AM+ECM
Buddleja madagascariensis	Loganiaceae	Angiospermae	
Buddleja officinalis	Loganiaceae	Angiospermae	
Gardneria angustifolia	Loganiaceae	Angiospermae	
Gardneria multiflora	Loganiaceae	Angiospermae	AM
Gelsemium elegans	Loganiaceae	Angiospermae	

Mitrasacme pygmaea	Loganiaceae	Angiospermae	AM
Strychnos angustiflora	Loganiaceae	Angiospermae	
Strychnos cathayensis	Loganiaceae	Angiospermae	
Strychnos ignatii	Loganiaceae	Angiospermae	
Strychnos nitida	Loganiaceae	Angiospermae	
Strychnos nux-vomica	Loganiaceae	Angiospermae	AM
Strychnos ovata	Loganiaceae	Angiospermae	
Strychnos umbellata	Loganiaceae	Angiospermae	
Strychnos wallichiana	Loganiaceae	Angiospermae	
Helixanthera parasitica	Loranthaceae	Angiospermae	
Loranthus tanakae	Loranthaceae	Angiospermae	
Macrosolen cochinchinensis	Loranthaceae	Angiospermae	
Scurrula parasitica	Loranthaceae	Angiospermae	
Taxillus caloreas	Loranthaceae	Angiospermae	
Taxillus chinensis	Loranthaceae	Angiospermae	
Taxillus delavayi	Loranthaceae	Angiospermae	
Taxillus levinei	Loranthaceae	Angiospermae	
Taxillus nigrans	Loranthaceae	Angiospermae	
Taxillus sutchuenensis var. sutchuenensis	Loranthaceae	Angiospermae	ORM
Viscum coloratum	Loranthaceae	Angiospermae	
Viscum liquidambaricolum	Loranthaceae	Angiospermae	
Viscum nudum	Loranthaceae	Angiospermae	
Viscum ovalifolium	Loranthaceae	Angiospermae	
Lagerstroemia indica	Lythraceae	Angiospermae	AM+NM
Lagerstroemia indica f. alba	Lythraceae	Angiospermae	AM+NM
Lagerstroemia speciosa	Lythraceae	Angiospermae	AM+NM
Lagerstroemia subcostata	Lythraceae	Angiospermae	AM
Lawsonia inermis	Lythraceae	Angiospermae	AM+NM
Lythrum salicaria	Lythraceae	Angiospermae	AM+NM
Woodfordia fruticosa	Lythraceae	Angiospermae	AM+NM
Illicium difengpi	Magnoliaceae	Angiospermae	ORM
Illicium henryi	Magnoliaceae	Angiospermae	
Illicium jiadifengpi var. jiadifengpi	Magnoliaceae	Angiospermae	

Illicium lanceolatum	Magnoliaceae	Angiospermae	
Illicium tsangii	Magnoliaceae	Angiospermae	
Illicium verum	Magnoliaceae	Angiospermae	AM
Kadsura coccinea	Magnoliaceae	Angiospermae	
Kadsura heteroclita	Magnoliaceae	Angiospermae	
Kadsura interior	Magnoliaceae	Angiospermae	
Kadsura japonica	Magnoliaceae	Angiospermae	AM
Kadsura longipedunculata	Magnoliaceae	Angiospermae	
Liriodendron chinense	Magnoliaceae	Angiospermae	AM
Magnolia biondii	Magnoliaceae	Angiospermae	
Magnolia coco	Magnoliaceae	Angiospermae	
Magnolia denudata	Magnoliaceae	Angiospermae	AM
Magnolia grandiflora	Magnoliaceae	Angiospermae	AM
Magnolia hypoleuca	Magnoliaceae	Angiospermae	
Magnolia liliflora	Magnoliaceae	Angiospermae	AM
Magnolia officinalis	Magnoliaceae	Angiospermae	AM
Magnolia officinalis subsp. biloba	Magnoliaceae	Angiospermae	AM
Magnolia pilocarpa	Magnoliaceae	Angiospermae	
Magnolia rostrata	Magnoliaceae	Angiospermae	
Magnolia sieboldii	Magnoliaceae	Angiospermae	AM
Magnolia sinensis	Magnoliaceae	Angiospermae	
Magnolia wilsonii	Magnoliaceae	Angiospermae	
Mahonia decipiens	Magnoliaceae	Angiospermae	
Mahonia fortunei	Magnoliaceae	Angiospermae	AM
Mahonia gracilipes	Magnoliaceae	Angiospermae	
Manglietia fordiana	Magnoliaceae	Angiospermae	
Manglietia patungensis	Magnoliaceae	Angiospermae	
Michelia alba	Magnoliaceae	Angiospermae	
Michelia figo	Magnoliaceae	Angiospermae	AM
Michelia maudiae	Magnoliaceae	Angiospermae	AM
Schisandra chinensis	Magnoliaceae	Angiospermae	AM+NM
Schisandra henryi	Magnoliaceae	Angiospermae	
Schisandra incarnata	Magnoliaceae	Angiospermae	

Schisandra neglecta	Magnoliaceae	Angiospermae	
Schisandra propinqua	Magnoliaceae	Angiospermae	
Schisandra sphenanthera	Magnoliaceae	Angiospermae	AM
Abelmoschus manihot	Malvaceae	Angiospermae	
Abelmoschus moschatus	Malvaceae	Angiospermae	AM
Abelmoschus muliensis	Malvaceae	Angiospermae	
Abelmoschus sagittifolius	Malvaceae	Angiospermae	
Abutilon indicum	Malvaceae	Angiospermae	AM+NM
Abutilon theophrasti	Malvaceae	Angiospermae	AM
Althaea officinalis	Malvaceae	Angiospermae	AM
Althaea rosea	Malvaceae	Angiospermae	AM
Hibiscus mutabilis	Malvaceae	Angiospermae	AM+NM
Hibiscus mutabilis f. mutablis	Malvaceae	Angiospermae	AM+NM
Hibiscus radiatus	Malvaceae	Angiospermae	
Hibiscus syriacus	Malvaceae	Angiospermae	AM
Hibiscus trionum	Malvaceae	Angiospermae	
Malva sinensis	Malvaceae	Angiospermae	AM
Malva verticillata	Malvaceae	Angiospermae	AM
Malvastrum coromandelianum	Malvaceae	Angiospermae	AM
Sida acuta	Malvaceae	Angiospermae	AM+NM
Sida szechuensis	Malvaceae	Angiospermae	AM
Urena lobata	Malvaceae	Angiospermae	AM+NM
Maranta arundinacea	Marantaceae	Angiospermae	AM
Phrynium capitatum	Marantaceae	Angiospermae	AM
Allomorphia balansae	Melastomataceae	Angiospermae	
Blastus apricus	Melastomataceae	Angiospermae	
Blastus cavaleriei	Melastomataceae	Angiospermae	
Blastus cochinchinensis	Melastomataceae	Angiospermae	
Blastus dunnianus	Melastomataceae	Angiospermae	
Blastus pauciflorus	Melastomataceae	Angiospermae	
Bredia amoena	Melastomataceae	Angiospermae	
Bredia esquirolii var. cordata	Melastomataceae	Angiospermae	
Bredia quadrangularis	Melastomataceae	Angiospermae	

Bredia sinensis	Melastomataceae	Angiospermae	
Melastoma affine	Melastomataceae	Angiospermae	NM
Melastoma candidum	Melastomataceae	Angiospermae	AM
Melastoma dodecandrum	Melastomataceae	Angiospermae	
Melastoma normale	Melastomataceae	Angiospermae	
Melastoma sanguineum	Melastomataceae	Angiospermae	
Osbeckia chinensis	Melastomataceae	Angiospermae	
Osbeckia crinita	Melastomataceae	Angiospermae	
Osbeckia paludosa	Melastomataceae	Angiospermae	
Oxyspora paniculata	Melastomataceae	Angiospermae	
Phyllagathis cavaleriei	Melastomataceae	Angiospermae	
Phyllagathis cavaleriei var. tankahkeei	Melastomataceae	Angiospermae	
Phyllagathis elattandra	Melastomataceae	Angiospermae	
Phyllagathis fordii	Melastomataceae	Angiospermae	
Sarcopyramis nepalensis	Melastomataceae	Angiospermae	
Sonerila cantonensis	Melastomataceae	Angiospermae	
Sonerila rivularis	Melastomataceae	Angiospermae	
Styrophyton caudatum	Melastomataceae	Angiospermae	
Cipadessa cinerascens	Meliaceae	Angiospermae	AM
Khaya senegalensis	Meliaceae	Angiospermae	
Melia azedarach	Meliaceae	Angiospermae	AM+NM
Melia toosendan	Meliaceae	Angiospermae	NM
Munronia sinica	Meliaceae	Angiospermae	
Toona sinensis	Meliaceae	Angiospermae	AM
Cyclea barbata	Menispermaceae	Angiospermae	
Diploclisia glaucescens	Menispermaceae	Angiospermae	
Fibraurea recisa	Menispermaceae	Angiospermae	AM
Hypserpa nitida	Menispermaceae	Angiospermae	
Sinomenium acutum	Menispermaceae	Angiospermae	AM
Stephania cepharantha	Menispermaceae	Angiospermae	
Stephania dielsiana	Menispermaceae	Angiospermae	
Stephania epigaea	Menispermaceae	Angiospermae	
Stephania glabra	Menispermaceae	Angiospermae	

Stephania hainanensis	Menispermaceae	Angiospermae	
Stephania japonica	Menispermaceae	Angiospermae	AM
Stephania succifera	Menispermaceae	Angiospermae	
Stephania sutchuenensis	Menispermaceae	Angiospermae	
Stephania tetrandra	Menispermaceae	Angiospermae	AM
Tinospora crispa	Menispermaceae	Angiospermae	
Tinospora sagittata	Menispermaceae	Angiospermae	
Tinospora sagittata var. craveniana	Menispermaceae	Angiospermae	
Tinospora sagittata var. yunnanensis	Menispermaceae	Angiospermae	
Tinospora sinensis	Menispermaceae	Angiospermae	AM
Artocarpus nitidus	Moraceae	Angiospermae	AM
Artocarpus nitidus subsp. lingnanensis	Moraceae	Angiospermae	AM
Cannabis sativa	Moraceae	Angiospermae	AM
Cannabis sativa subsp. indica	Moraceae	Angiospermae	AM
Cudrania cochinchinensis	Moraceae	Angiospermae	AM
Cudrania tricuspidata	Moraceae	Angiospermae	AM
Ficus carica	Moraceae	Angiospermae	AM+NM
Ficus hirta	Moraceae	Angiospermae	AM
Ficus pumila	Moraceae	Angiospermae	AM
Ficus variolosa	Moraceae	Angiospermae	AM
Humulus scandens	Moraceae	Angiospermae	AM
Morus alba	Moraceae	Angiospermae	AM+ECM+NM
Morus mongolica	Moraceae	Angiospermae	
Musa basjoo	Musaceae	Angiospermae	AM
Musa wilsonii	Musaceae	Angiospermae	
Musella lasiocarpa	Musaceae	Angiospermae	
Bontia daphnoides	Myoporaceae	Angiospermae	
Myrica rubra	Myricaceae	Angiospermae	AM
Myristica fragrans	Myristicaceae	Angiospermae	AM
Ardisia affinis	Myrsinaceae	Angiospermae	
Ardisia alyxiaefolia	Myrsinaceae	Angiospermae	
Ardisia brevicaulis	Myrsinaceae	Angiospermae	
Ardisia brunnescens	Myrsinaceae	Angiospermae	

Ardisia chinensis	Myrsinaceae	Angiospermae	AM
Ardisia crenata	Myrsinaceae	Angiospermae	AM
Ardisia crispa	Myrsinaceae	Angiospermae	AM
Ardisia crispa var. amplifolia	Myrsinaceae	Angiospermae	AM
Ardisia crispa var. dielsii	Myrsinaceae	Angiospermae	AM
Ardisia densilepidotula	Myrsinaceae	Angiospermae	
Ardisia elegans	Myrsinaceae	Angiospermae	
Ardisia ensifolia	Myrsinaceae	Angiospermae	
Ardisia faberi	Myrsinaceae	Angiospermae	
Ardisia filiformis	Myrsinaceae	Angiospermae	
Ardisia gigantifolia	Myrsinaceae	Angiospermae	
Ardisia humilis	Myrsinaceae	Angiospermae	
Ardisia japonica	Myrsinaceae	Angiospermae	AM
Ardisia maculosa	Myrsinaceae	Angiospermae	
Ardisia mamillata	Myrsinaceae	Angiospermae	
Ardisia nervosa	Myrsinaceae	Angiospermae	
Ardisia primulaefolia	Myrsinaceae	Angiospermae	
Ardisia punctata	Myrsinaceae	Angiospermae	
Ardisia pusilla	Myrsinaceae	Angiospermae	AM
Ardisia quinquegona	Myrsinaceae	Angiospermae	AM+NM
Ardisia villosa	Myrsinaceae	Angiospermae	
Embelia laeta	Myrsinaceae	Angiospermae	
Embelia longifolia	Myrsinaceae	Angiospermae	
Embelia parviflora	Myrsinaceae	Angiospermae	
Embelia ribes	Myrsinaceae	Angiospermae	
Embelia rudis	Myrsinaceae	Angiospermae	
Embelia subcoriacea	Myrsinaceae	Angiospermae	
Maesa japonica	Myrsinaceae	Angiospermae	AM
Maesa perlarius	Myrsinaceae	Angiospermae	
Myrsine africana	Myrsinaceae	Angiospermae	AM
Rapanea neriifolia	Myrsinaceae	Angiospermae	
Baeckea frutescens	Myrtaceae	Angiospermae	
Cleistocalyx operculatus	Myrtaceae	Angiospermae	

Eucalyptus robusta	Myrtaceae	Angiospermae	AM+ECM+NM
Rhodomyrtus tomentosa	Myrtaceae	Angiospermae	AM
Nepenthes mirabilis	Nepenthaceae	Angiospermae	
Boerhavia diffusa	Nyctaginaceae	Angiospermae	AM+NM
Bougainvillea glabra	Nyctaginaceae	Angiospermae	
Mirabilis jalapa	Nyctaginaceae	Angiospermae	AM+NM
Oxybaphus himalaicus var. chinensis	Nyctaginaceae	Angiospermae	
Euryale ferox	Nymphaeaceae	Angiospermae	NM
Nelumbo nucifera	Nymphaeaceae	Angiospermae	AM+NM
Camptotheca acuminata	Nyssaceae	Angiospermae	AM
Erythropalum scandens	Olacaceae	Angiospermae	
Schoepfia fragrans	Olacaceae	Angiospermae	
Forsythia suspensa	Oleaceae	Angiospermae	AM
Fraxinus americana	Oleaceae	Angiospermae	AM+ECM
Fraxinus bungeana	Oleaceae	Angiospermae	
Fraxinus chinensis	Oleaceae	Angiospermae	AM+NM
Fraxinus malacophylla	Oleaceae	Angiospermae	AM
Fraxinus paxiana	Oleaceae	Angiospermae	
Fraxinus pennsylvanica	Oleaceae	Angiospermae	AM+NM
Fraxinus pennsylvanica var. subintegerrima	Oleaceae	Angiospermae	AM+NM
Fraxinus retusifoliolata	Oleaceae	Angiospermae	
Fraxinus rhynchophylla	Oleaceae	Angiospermae	AM+NM
Fraxinus szaboana	Oleaceae	Angiospermae	
Jasminum albicalyx	Oleaceae	Angiospermae	
Jasminum coarctatum	Oleaceae	Angiospermae	
Jasminum duclouxii	Oleaceae	Angiospermae	
Jasminum elongatum	Oleaceae	Angiospermae	
Jasminum laurifolium	Oleaceae	Angiospermae	AM
Jasminum pentaneurum	Oleaceae	Angiospermae	NM
Jasminum sambac	Oleaceae	Angiospermae	AM+NM
Jasminum seguinii	Oleaceae	Angiospermae	
Ligustrum lucidum	Oleaceae	Angiospermae	AM
Ligustrum quihoui	Oleaceae	Angiospermae	

Ligustrum sinense	Oleaceae	Angiospermae	AM+ECM
Syringa pinnatifolia	Oleaceae	Angiospermae	
Syringa reticulata var. amurensis	Oleaceae	Angiospermae	AM
Epilobium angustifolium	Onagraceae	Angiospermae	AM+NM
Epilobium hirsutum	Onagraceae	Angiospermae	AM+NM
Epilobium palustre	Onagraceae	Angiospermae	AM+NM
Ludwigia adscendens	Onagraceae	Angiospermae	AM
Ludwigia epilobioides	Onagraceae	Angiospermae	
Ludwigia hyssopifolia	Onagraceae	Angiospermae	AM+NM
Oenothera glazioviana	Onagraceae	Angiospermae	AM
Oenothera rosea	Onagraceae	Angiospermae	AM
Oenothera stricta	Onagraceae	Angiospermae	
Anoectochilus roxburghii	Orchidaceae	Angiospermae	ORM
Bulbophyllum omerandrum	Orchidaceae	Angiospermae	
Diplomeris pulchella	Orchidaceae	Angiospermae	
Gastrochilus platycalcaratus	Orchidaceae	Angiospermae	
Goodyera procera	Orchidaceae	Angiospermae	
Goodyera repens	Orchidaceae	Angiospermae	ORM
Goodyera schlechtendaliana	Orchidaceae	Angiospermae	
Gymnadenia conopsea	Orchidaceae	Angiospermae	ORM
Gymnadenia crassinervis	Orchidaceae	Angiospermae	
Gymnadenia emeiensis	Orchidaceae	Angiospermae	
Gymnadenia orchidis	Orchidaceae	Angiospermae	
Habenaria dentata	Orchidaceae	Angiospermae	ORM
Habenaria viridiflora	Orchidaceae	Angiospermae	
Herminium monorchis	Orchidaceae	Angiospermae	ORM
Ludisia discolor	Orchidaceae	Angiospermae	ORM
Nervilia aragoana	Orchidaceae	Angiospermae	
Nervilia fordii	Orchidaceae	Angiospermae	
Nervilia plicata	Orchidaceae	Angiospermae	
Orchis latifolia	Orchidaceae	Angiospermae	
Peristylus densus	Orchidaceae	Angiospermae	
Peristylus tentaculatus	Orchidaceae	Angiospermae	

Spiranthes sinensis	Orchidaceae	Angiospermae	AM+ORM
Aeginetia indica	Orobanchaceae	Angiospermae	
Boschniakia himalaica	Orobanchaceae	Angiospermae	
Boschniakia rossica	Orobanchaceae	Angiospermae	
Cistanche deserticola	Orobanchaceae	Angiospermae	
Cistanche tubulosa	Orobanchaceae	Angiospermae	
Orobanche coerulescens	Orobanchaceae	Angiospermae	
Averrhoa carambola	Oxalidaceae	Angiospermae	AM+NM
Biophytum fruticosum	Oxalidaceae	Angiospermae	
Biophytum sensitivum	Oxalidaceae	Angiospermae	AM+NM
Oxalis acetosella	Oxalidaceae	Angiospermae	AM+NM
Oxalis acetosella subsp. griffithii	Oxalidaceae	Angiospermae	AM+NM
Oxalis corniculata	Oxalidaceae	Angiospermae	AM
Oxalis corymbosa	Oxalidaceae	Angiospermae	AM
Areca catechu	Palmae	Angiospermae	AM+NM
Calamus macrorrhynchus	Palmae	Angiospermae	
Cocos nucifera	Palmae	Angiospermae	AM
Corypha umbraculifera	Palmae	Angiospermae	
Livistona chinensis	Palmae	Angiospermae	AM
Rhapis excelsa	Palmae	Angiospermae	AM
Trachycarpus fortunei	Palmae	Angiospermae	AM
Pandanus furcatus	Pandanaceae	Angiospermae	NM
Pandanus tectorius	Pandanaceae	Angiospermae	AM+NM
Pandanus gressitii	Pandanaceae	Angiospermae	
Chelidonium majus	Papaveraceae	Angiospermae	AM+NM
Corydalis adunca	Papaveraceae	Angiospermae	
Corydalis appendiculata	Papaveraceae	Angiospermae	
Corydalis balansae	Papaveraceae	Angiospermae	
Corydalis boweri	Papaveraceae	Angiospermae	
Corydalis bulbillifera	Papaveraceae	Angiospermae	
Corydalis cheilanthifolia	Papaveraceae	Angiospermae	
Corydalis dasyptera	Papaveraceae	Angiospermae	
Corydalis daviddi	Papaveraceae	Angiospermae	

Corydalis decumbens	Papaveraceae	Angiospermae	
Corydalis densispica	Papaveraceae	Angiospermae	
Corydalis edulis	Papaveraceae	Angiospermae	
Corydalis fangshanensis	Papaveraceae	Angiospermae	
Corydalis hendersonii	Papaveraceae	Angiospermae	
Corydalis humicola	Papaveraceae	Angiospermae	
Corydalis humosa	Papaveraceae	Angiospermae	
Corydalis imbricata	Papaveraceae	Angiospermae	
Corydalis incisa	Papaveraceae	Angiospermae	
Corydalis kingii var. megalantha	Papaveraceae	Angiospermae	
Corydalis latiloba	Papaveraceae	Angiospermae	
Corydalis linarioides	Papaveraceae	Angiospermae	
Corydalis linstowiana	Papaveraceae	Angiospermae	
Corydalis meifolia	Papaveraceae	Angiospermae	
Corydalis ochotensis	Papaveraceae	Angiospermae	
Corydalis ophiocarpa	Papaveraceae	Angiospermae	
Corydalis pallida	Papaveraceae	Angiospermae	
Corydalis pseudomucronata	Papaveraceae	Angiospermae	
Corydalis racemosa	Papaveraceae	Angiospermae	
Corydalis repens	Papaveraceae	Angiospermae	
Corydalis rheinbebeniana	Papaveraceae	Angiospermae	
Corydalis rheinbebeniana var. rheinbabeniana	Papaveraceae	Angiospermae	
Corydalis saxicola	Papaveraceae	Angiospermae	
Corydalis schanginii	Papaveraceae	Angiospermae	NM
Corydalis sheareri	Papaveraceae	Angiospermae	
Corydalis temulifolia	Papaveraceae	Angiospermae	
Corydalis trachycarpa	Papaveraceae	Angiospermae	
Corydalis tsayulensis	Papaveraceae	Angiospermae	
Corydalis yanhusuo	Papaveraceae	Angiospermae	ORM
Dactylicapnos scandens	Papaveraceae	Angiospermae	
Dactylicapnos torulosa	Papaveraceae	Angiospermae	
Dicentra macrantha	Papaveraceae	Angiospermae	
Dicentra spectabilis	Papaveraceae	Angiospermae	AM

Dicranostigma leptopodum	Papaveraceae	Angiospermae	AM
Eomecon chionantha	Papaveraceae	Angiospermae	
Hylomecon japonica	Papaveraceae	Angiospermae	AM
Hylomecon japonica var. subincisa	Papaveraceae	Angiospermae	AM
Hypecoum erectum	Papaveraceae	Angiospermae	AM
Hypecoum leptocarpum	Papaveraceae	Angiospermae	
Macleaya cordata	Papaveraceae	Angiospermae	AM
Macleaya microcarpa	Papaveraceae	Angiospermae	
Meconopsis napaulensis	Papaveraceae	Angiospermae	
Meconopsis punicea	Papaveraceae	Angiospermae	
Meconopsis quintuplinervia	Papaveraceae	Angiospermae	
Meconopsis racemosa	Papaveraceae	Angiospermae	
Papaver rhoeas	Papaveraceae	Angiospermae	AM+NM
Papaver somniferum	Papaveraceae	Angiospermae	AM+NM
Stylophorum lasiocarpum	Papaveraceae	Angiospermae	
Passiflora coerulea	Passifloraceae	Angiospermae	AM
Passiflora cupiformis	Passifloraceae	Angiospermae	
Passiflora edulis	Passifloraceae	Angiospermae	AM
Passiflora foetida	Passifloraceae	Angiospermae	AM
Passiflora henryi	Passifloraceae	Angiospermae	
Passiflora jugorum	Passifloraceae	Angiospermae	
Passiflora wilsonii	Passifloraceae	Angiospermae	
Sesamum indicum	Pedaliaceae	Angiospermae	AM
Philydrum lanuginosum	Philydraceae	Angiospermae	AM+NM
Phryma leptostachya subsp. asiatica	Phrymaceae	Angiospermae	AM
Phytolacca acinosa	Phytolaccaceae	Angiospermae	AM
Phytolacca americana	Phytolaccaceae	Angiospermae	NM
Peperomia tetraphylla	Piperaceae	Angiospermae	AM
Piper arboricola	Piperaceae	Angiospermae	
Piper betle	Piperaceae	Angiospermae	AM
Piper boehmeriaefolium var. tonkinense	Piperaceae	Angiospermae	
Piper flaviflorum	Piperaceae	Angiospermae	AM
Piper hancei	Piperaceae	Angiospermae	NM

Piper longum	Piperaceae	Angiospermae	AM
Piper mullesua	Piperaceae	Angiospermae	
Piper nigrum	Piperaceae	Angiospermae	AM+NM
Piper retrofractum	Piperaceae	Angiospermae	
Piper sarmentosum	Piperaceae	Angiospermae	AM
Piper wallichii	Piperaceae	Angiospermae	
Piper yunnanense	Piperaceae	Angiospermae	
Pittosporum crispulum	Pittosporaceae	Angiospermae	
Pittosporum glabratum	Pittosporaceae	Angiospermae	
Pittosporum kerrii	Pittosporaceae	Angiospermae	
Pittosporum perryanum	Pittosporaceae	Angiospermae	
Pittosporum podocarpum	Pittosporaceae	Angiospermae	
Pittosporum trigonocarpum	Pittosporaceae	Angiospermae	
Veronica anagallis-aquatica Linnaeus	Plantaginaceae	Angiospermae	
Ceratostigma minus	Plumbaginaceae	Angiospermae	
Ceratostigma minus f. mimus	Plumbaginaceae	Angiospermae	
Ceratostigma willmottianum	Plumbaginaceae	Angiospermae	
Limonium aureum	Plumbaginaceae	Angiospermae	
Limonium aureum var. potaninii	Plumbaginaceae	Angiospermae	
Limonium gmelinii	Plumbaginaceae	Angiospermae	AM
Limonium wrightii	Plumbaginaceae	Angiospermae	
Plumbagella micrantha	Plumbaginaceae	Angiospermae	
Plumbago auriculata	Plumbaginaceae	Angiospermae	AM
Plumbago zeylanica	Plumbaginaceae	Angiospermae	AM
Polygala arillata	Polygalaceae	Angiospermae	
Polygala arvensis	Polygalaceae	Angiospermae	
Polygala barbellata	Polygalaceae	Angiospermae	
Polygala caudata	Polygalaceae	Angiospermae	
Polygala crotalarioides	Polygalaceae	Angiospermae	
Polygala fallax	Polygalaceae	Angiospermae	
Polygala glomerata	Polygalaceae	Angiospermae	
Polygala glomerata var. villosa	Polygalaceae	Angiospermae	
Polygala hybrida	Polygalaceae	Angiospermae	AM

Polygala isocarpa	Polygalaceae	Angiospermae	
Polygala japonica	Polygalaceae	Angiospermae	
Polygala koi	Polygalaceae	Angiospermae	
Polygala persicariifolia	Polygalaceae	Angiospermae	
Polygala sibirica	Polygalaceae	Angiospermae	AM
Polygala sibirica var. megalopha	Polygalaceae	Angiospermae	AM
Polygala subopposita	Polygalaceae	Angiospermae	
Polygala tatarinowii	Polygalaceae	Angiospermae	AM
Polygala tenuifolia	Polygalaceae	Angiospermae	ORM
Polygala tricornis	Polygalaceae	Angiospermae	
Polygala wattersii	Polygalaceae	Angiospermae	
Salomonia cantoniensis	Polygalaceae	Angiospermae	
Salomonia cantoniensis var. edentula	Polygalaceae	Angiospermae	
Securidaca inappendiculata	Polygalaceae	Angiospermae	
Fagopyrum dibotrys	Polygonaceae	Angiospermae	AM
Fagopyrum esculentum	Polygonaceae	Angiospermae	AM+NM
Fagopyrum tataricum	Polygonaceae	Angiospermae	AM
Fallopia denticulata	Polygonaceae	Angiospermae	
Fallopia multiflora	Polygonaceae	Angiospermae	ECM
Fallopia multiflora var. ciliinerve	Polygonaceae	Angiospermae	ECM
Polygonum amplexicaule	Polygonaceae	Angiospermae	AM
Polygonum amplexicaule var. sinense	Polygonaceae	Angiospermae	AM
Polygonum aviculare	Polygonaceae	Angiospermae	AM+NM
Polygonum bistorta	Polygonaceae	Angiospermae	AM+ECM+NM
Polygonum capitatum	Polygonaceae	Angiospermae	AM+ECM+NM
Polygonum chinense	Polygonaceae	Angiospermae	AM
Polygonum hydropiper	Polygonaceae	Angiospermae	NM
Polygonum japonicum	Polygonaceae	Angiospermae	
Polygonum orientale	Polygonaceae	Angiospermae	
Polygonum paleaceum	Polygonaceae	Angiospermae	
Polygonum runcinatum var. sinense	Polygonaceae	Angiospermae	
Polygonum sieboldii	Polygonaceae	Angiospermae	
Polygonum suffultum	Polygonaceae	Angiospermae	

Polygonum tinctorium	Polygonaceae	Angiospermae	
Polygonum viviparum	Polygonaceae	Angiospermae	AM+ECM
Pongamia pinnata	Polygonaceae	Angiospermae	AM+NM
Pteroxygonum giraldii	Polygonaceae	Angiospermae	
Reynoutria japonica	Polygonaceae	Angiospermae	AM+NM
Rheum officinale	Polygonaceae	Angiospermae	
Rheum palmatum	Polygonaceae	Angiospermae	AM
Rheum tanguticum	Polygonaceae	Angiospermae	
Rumex acetosa	Polygonaceae	Angiospermae	AM+NM
Rumex japonicus	Polygonaceae	Angiospermae	AM+NM
Rumex nepalensis	Polygonaceae	Angiospermae	AM
Eichhornia crassipes	Pontederiaceae	Angiospermae	AM+NM
Portulaca grandiflora	Portulacaceae	Angiospermae	NM
Portulaca oleracea	Portulacaceae	Angiospermae	AM+NM
Portulaca pilosa	Portulacaceae	Angiospermae	AM+NM
Portulaca quadrifida	Portulacaceae	Angiospermae	NM
Talinum paniculatum	Portulacaceae	Angiospermae	AM
Potamogeton pectinatus	Potamogetonaceae	Angiospermae	NM
Androsace sarmentosa	Primulaceae	Angiospermae	
Androsace strigillosa	Primulaceae	Angiospermae	
Androsace tapete	Primulaceae	Angiospermae	
Androsace umbellata	Primulaceae	Angiospermae	
Lysimachia barystachys	Primulaceae	Angiospermae	AM
Lysimachia candida	Primulaceae	Angiospermae	
Lysimachia capillipes	Primulaceae	Angiospermae	
Lysimachia christinae	Primulaceae	Angiospermae	AM
Lysimachia circaeoides	Primulaceae	Angiospermae	
Lysimachia clethroides	Primulaceae	Angiospermae	AM
Lysimachia congestiflora	Primulaceae	Angiospermae	AM
Lysimachia decurrens	Primulaceae	Angiospermae	
Lysimachia foenum-graecum	Primulaceae	Angiospermae	
Lysimachia fortunei	Primulaceae	Angiospermae	AM
Lysimachia insignis	Primulaceae	Angiospermae	

Lysimachia klattiana	Primulaceae	Angiospermae	AM
Lysimachia lobelioides	Primulaceae	Angiospermae	AM
Lysimachia longipes	Primulaceae	Angiospermae	
Lysimachia paridiformis	Primulaceae	Angiospermae	
Primula atrodentata	Primulaceae	Angiospermae	AM
Primula faberi	Primulaceae	Angiospermae	
Primula forbesii	Primulaceae	Angiospermae	
Primula forrestii	Primulaceae	Angiospermae	
Primula sikkimensis	Primulaceae	Angiospermae	
Primula tangutica	Primulaceae	Angiospermae	
Heliciopsis terminalis	Proteaceae	Angiospermae	
Punica granatum	Punicaceae	Angiospermae	AM
Pyrola calliantha var. calliantha	Pyrolaceae	Angiospermae	ORM
Pyrola decorata	Pyrolaceae	Angiospermae	
Aconitum anthoroideum	Ranunculaceae	Angiospermae	
Aconitum austroyunnanense	Ranunculaceae	Angiospermae	
Aconitum barbatum var. hispidum	Ranunculaceae	Angiospermae	
Aconitum barbatum var. puberulum	Ranunculaceae	Angiospermae	
Aconitum brachypodum	Ranunculaceae	Angiospermae	
Aconitum brachypodum var. laxiflorum	Ranunculaceae	Angiospermae	
Aconitum carmichaeli var. hwangshanicum	Ranunculaceae	Angiospermae	
Aconitum chasmanthum	Ranunculaceae	Angiospermae	AM
Aconitum chilienshanicum	Ranunculaceae	Angiospermae	
Aconitum coreanum	Ranunculaceae	Angiospermae	ORM
Aconitum episcopale	Ranunculaceae	Angiospermae	
Aconitum flavum	Ranunculaceae	Angiospermae	
Aconitum gymnandrum	Ranunculaceae	Angiospermae	AM
Aconitum hemsleyanum	Ranunculaceae	Angiospermae	
Aconitum hemsleyanum var. circinatum	Ranunculaceae	Angiospermae	
Aconitum karakolicum	Ranunculaceae	Angiospermae	
Aconitum kongboense	Ranunculaceae	Angiospermae	
Aconitum kusnezoffii	Ranunculaceae	Angiospermae	
Aconitum longilobum	Ranunculaceae	Angiospermae	

Aconitum nagarum	Ranunculaceae	Angiospermae	
Aconitum nagarum var. heterotrichum	Ranunculaceae	Angiospermae	
Aconitum nagarum var. heterotrichum f. dielsianum	Ranunculaceae	Angiospermae	
Aconitum nagarum var. lasiandrum	Ranunculaceae	Angiospermae	
Aconitum naviculare	Ranunculaceae	Angiospermae	
Aconitum pendulum	Ranunculaceae	Angiospermae	
Aconitum polyschistum	Ranunculaceae	Angiospermae	
Aconitum pomeense	Ranunculaceae	Angiospermae	
Aconitum racemulosum	Ranunculaceae	Angiospermae	
Aconitum richardsonianum var. pseudosessiliflorum	Ranunculaceae	Angiospermae	
Aconitum rotundifolium	Ranunculaceae	Angiospermae	AM
Aconitum scaposum	Ranunculaceae	Angiospermae	
Aconitum scaposum var. vaginatum	Ranunculaceae	Angiospermae	
Aconitum sessiliflorum	Ranunculaceae	Angiospermae	
Aconitum sinomontanum	Ranunculaceae	Angiospermae	
Aconitum sinomontanum var. angustius	Ranunculaceae	Angiospermae	
Aconitum soongaricum	Ranunculaceae	Angiospermae	NM
Aconitum spicatum	Ranunculaceae	Angiospermae	
Aconitum sungpanense	Ranunculaceae	Angiospermae	
Aconitum taipeicum	Ranunculaceae	Angiospermae	
Aconitum tanguticum	Ranunculaceae	Angiospermae	
Aconitum transsectum	Ranunculaceae	Angiospermae	
Aconitum vilmorinianum	Ranunculaceae	Angiospermae	
Aconitum vilmorinianum var. altifidum	Ranunculaceae	Angiospermae	
Actaea asiatica	Ranunculaceae	Angiospermae	
Adonis amurensis	Ranunculaceae	Angiospermae	
Adonis chrysocyatha	Ranunculaceae	Angiospermae	
Adonis coerulea	Ranunculaceae	Angiospermae	
Adonis sutchuenensis	Ranunculaceae	Angiospermae	
Anemone altaica	Ranunculaceae	Angiospermae	AM
Anemone amurensis	Ranunculaceae	Angiospermae	
Anemone davidii	Ranunculaceae	Angiospermae	
Anemone demissa	Ranunculaceae	Angiospermae	

Anemone flaccida	Ranunculaceae	Angiospermae	
Anemone hupehensis	Ranunculaceae	Angiospermae	AM
Anemone hupehensis var. hupehensis f. alba	Ranunculaceae	Angiospermae	AM
Anemone hupehensis var. japonica	Ranunculaceae	Angiospermae	AM
Anemone imbricata	Ranunculaceae	Angiospermae	
Anemone obtusiloba subsp. ovalifolia	Ranunculaceae	Angiospermae	
Anemone raddeana	Ranunculaceae	Angiospermae	
Anemone reflexa	Ranunculaceae	Angiospermae	
Anemone rivularis	Ranunculaceae	Angiospermae	NM
Anemone rivularis var. flore-minore	Ranunculaceae	Angiospermae	NM
Anemone tomentosa	Ranunculaceae	Angiospermae	ORM
Anemone vitifolia	Ranunculaceae	Angiospermae	NM
Aquilegia incurvata	Ranunculaceae	Angiospermae	AM
Aquilegia oxysepala	Ranunculaceae	Angiospermae	
Aquilegia parviflora	Ranunculaceae	Angiospermae	
Aquilegia viridiflora	Ranunculaceae	Angiospermae	
Asteropyrum cavaleriei	Ranunculaceae	Angiospermae	
Beesia calthifolia	Ranunculaceae	Angiospermae	
Calathodes oxycarpa	Ranunculaceae	Angiospermae	
Callianthemum taipaicum	Ranunculaceae	Angiospermae	
Caltha palustris	Ranunculaceae	Angiospermae	AM+NM
Caltha palustris var. sibirica	Ranunculaceae	Angiospermae	AM+NM
Caltha scaposa	Ranunculaceae	Angiospermae	
Cimicifuga acerina	Ranunculaceae	Angiospermae	
Cimicifuga dahurica	Ranunculaceae	Angiospermae	
Cimicifuga foetida	Ranunculaceae	Angiospermae	
Cimicifuga foetida var. foliolosa	Ranunculaceae	Angiospermae	
Cimicifuga heracleifolia	Ranunculaceae	Angiospermae	
Cimicifuga nanchuenensis	Ranunculaceae	Angiospermae	
Cimicifuga simplex	Ranunculaceae	Angiospermae	
Clematis aethusifolia	Ranunculaceae	Angiospermae	
Clematis alsomitrifolia	Ranunculaceae	Angiospermae	
Clematis apiifolia	Ranunculaceae	Angiospermae	AM

Clematis apiifolia var. obtusidentata	Ranunculaceae	Angiospermae	AM
Clematis argentilucida	Ranunculaceae	Angiospermae	
Clematis armandii	Ranunculaceae	Angiospermae	
Clematis brevicaudata	Ranunculaceae	Angiospermae	
Clematis chinensis	Ranunculaceae	Angiospermae	
Clematis chrysocoma	Ranunculaceae	Angiospermae	
Clematis courtoisii	Ranunculaceae	Angiospermae	
Clematis crassifolia	Ranunculaceae	Angiospermae	
Clematis fasciculiflora	Ranunculaceae	Angiospermae	
Clematis filamentosa	Ranunculaceae	Angiospermae	
Clematis finetiana	Ranunculaceae	Angiospermae	
Clematis florida	Ranunculaceae	Angiospermae	AM
Clematis glauca	Ranunculaceae	Angiospermae	
Clematis gouriana	Ranunculaceae	Angiospermae	
Clematis henryi	Ranunculaceae	Angiospermae	
Clematis heracleifolia	Ranunculaceae	Angiospermae	
Clematis hexapetala	Ranunculaceae	Angiospermae	
Clematis huchouensis	Ranunculaceae	Angiospermae	
Clematis kerriana	Ranunculaceae	Angiospermae	
Clematis koreana	Ranunculaceae	Angiospermae	
Clematis lancifolia	Ranunculaceae	Angiospermae	
Clematis leschenaultiana	Ranunculaceae	Angiospermae	
Clematis loureiroana	Ranunculaceae	Angiospermae	
Clematis meyeniana	Ranunculaceae	Angiospermae	
Clematis meyeniana var. granulata	Ranunculaceae	Angiospermae	
Clematis montana	Ranunculaceae	Angiospermae	
Clematis peterae	Ranunculaceae	Angiospermae	
Clematis potaninii	Ranunculaceae	Angiospermae	
Clematis quinquefoliolata	Ranunculaceae	Angiospermae	
Clematis rehderiana	Ranunculaceae	Angiospermae	
Clematis tangutica	Ranunculaceae	Angiospermae	
Clematis terniflora	Ranunculaceae	Angiospermae	
Clematis terniflora var. mandshurica	Ranunculaceae	Angiospermae	AM

Clematis trichocarpa	Ranunculaceae	Angiospermae	
Clematis uncinata	Ranunculaceae	Angiospermae	
Clematis zygophylla	Ranunculaceae	Angiospermae	
Coptis chinensis	Ranunculaceae	Angiospermae	AM+ECM
Coptis chinensis var. brevisepala	Ranunculaceae	Angiospermae	AM+ECM
Coptis deltoidea	Ranunculaceae	Angiospermae	AM
Coptis omeiensis	Ranunculaceae	Angiospermae	
Coptis teeta	Ranunculaceae	Angiospermae	
Delphinium albocoeruleum	Ranunculaceae	Angiospermae	
Delphinium anthriscifolium	Ranunculaceae	Angiospermae	
Delphinium anthriscifolium var. majus	Ranunculaceae	Angiospermae	
Delphinium bonvalotii	Ranunculaceae	Angiospermae	
Delphinium bonvalotii var. eriostylum	Ranunculaceae	Angiospermae	
Delphinium brunonianum	Ranunculaceae	Angiospermae	
Delphinium candelabrum	Ranunculaceae	Angiospermae	
Delphinium candelabrum var. monanthum	Ranunculaceae	Angiospermae	
Delphinium chefoense	Ranunculaceae	Angiospermae	
Delphinium delavayi	Ranunculaceae	Angiospermae	
Delphinium delavayi var. pogonanthum	Ranunculaceae	Angiospermae	
Delphinium densiflorum	Ranunculaceae	Angiospermae	
Delphinium grandiflorum	Ranunculaceae	Angiospermae	
Delphinium honanense var. piliferum	Ranunculaceae	Angiospermae	
Delphinium kamaonense var. glabrescens	Ranunculaceae	Angiospermae	
Delphinium omeiense	Ranunculaceae	Angiospermae	
Delphinium pylzowii	Ranunculaceae	Angiospermae	
Delphinium pylzowii var. trigynum	Ranunculaceae	Angiospermae	
Delphinium taliense var. pubipes	Ranunculaceae	Angiospermae	
Delphinium yunnanense	Ranunculaceae	Angiospermae	
Dichocarpum auriculatum	Ranunculaceae	Angiospermae	
Dichocarpum basilare	Ranunculaceae	Angiospermae	
Dichocarpum dalzielii	Ranunculaceae	Angiospermae	
Dichocarpum trifoliolatum	Ranunculaceae	Angiospermae	
Helleborus thibetanus	Ranunculaceae	Angiospermae	

Hepatica nobilis var. asiatica	Ranunculaceae	Angiospermae	AM
Isopyrum manshuricum	Ranunculaceae	Angiospermae	
Leptopyrum fumarioides	Ranunculaceae	Angiospermae	
Nigella glandulifera	Ranunculaceae	Angiospermae	
Paeonia delavayi	Ranunculaceae	Angiospermae	
Paeonia delavayi var. angustiloba	Ranunculaceae	Angiospermae	
Paeonia delavayi var. lutea	Ranunculaceae	Angiospermae	
Paeonia emodi	Ranunculaceae	Angiospermae	AM
Paeonia lactiflora	Ranunculaceae	Angiospermae	AM
Paeonia mairei	Ranunculaceae	Angiospermae	
Paeonia obovata	Ranunculaceae	Angiospermae	AM+NM
Paeonia obovata var. willmottiae	Ranunculaceae	Angiospermae	AM+NM
Paeonia sinjiangensis	Ranunculaceae	Angiospermae	
Paeonia suffruticosa	Ranunculaceae	Angiospermae	AM
Paeonia suffruticosa var. papaveracea	Ranunculaceae	Angiospermae	AM
Paeonia veitchii	Ranunculaceae	Angiospermae	
Paraquilegia microphylla	Ranunculaceae	Angiospermae	
Pulsatilla ambigua	Ranunculaceae	Angiospermae	
Pulsatilla campanella	Ranunculaceae	Angiospermae	AM
Pulsatilla cernua	Ranunculaceae	Angiospermae	AM
Pulsatilla chinensis	Ranunculaceae	Angiospermae	AM
Pulsatilla dahurica	Ranunculaceae	Angiospermae	
Pulsatilla turczaninovii	Ranunculaceae	Angiospermae	
Ranunculus arvensis	Ranunculaceae	Angiospermae	AM
Ranunculus cantoniensis	Ranunculaceae	Angiospermae	AM
Ranunculus chinensis	Ranunculaceae	Angiospermae	
Ranunculus glareosus	Ranunculaceae	Angiospermae	
Ranunculus japonicus	Ranunculaceae	Angiospermae	AM
Ranunculus lingua	Ranunculaceae	Angiospermae	AM+NM
Ranunculus sceleratus	Ranunculaceae	Angiospermae	AM+NM
Ranunculus sieboldii	Ranunculaceae	Angiospermae	AM
Ranunculus tanguticus	Ranunculaceae	Angiospermae	
Ranunculus ternatus	Ranunculaceae	Angiospermae	

Semiaquilegia adoxoides	Ranunculaceae	Angiospermae	AM
Souliea vaginata	Ranunculaceae	Angiospermae	
Thalictrum acutifolium	Ranunculaceae	Angiospermae	
Thalictrum alpinum var. elatum	Ranunculaceae	Angiospermae	AM
Thalictrum aquilegifolium var. sibiricum	Ranunculaceae	Angiospermae	AM
Thalictrum atriplex	Ranunculaceae	Angiospermae	
Thalictrum delavayi	Ranunculaceae	Angiospermae	AM
Thalictrum faberi	Ranunculaceae	Angiospermae	
Thalictrum foetidum	Ranunculaceae	Angiospermae	AM
Thalictrum foliolosum	Ranunculaceae	Angiospermae	
Thalictrum glandulosissimum	Ranunculaceae	Angiospermae	
Thalictrum glandulosissimum var. chaotumgense	Ranunculaceae	Angiospermae	
Thalictrum ichangense	Ranunculaceae	Angiospermae	
Thalictrum javanicum	Ranunculaceae	Angiospermae	
Thalictrum microgynum	Ranunculaceae	Angiospermae	
Thalictrum minus	Ranunculaceae	Angiospermae	AM
Thalictrum minus var. hypoleucum	Ranunculaceae	Angiospermae	AM
Thalictrum omeiense	Ranunculaceae	Angiospermae	
Thalictrum petaloideum	Ranunculaceae	Angiospermae	NM
Thalictrum przewalskii	Ranunculaceae	Angiospermae	ECM
Thalictrum ramosum	Ranunculaceae	Angiospermae	
Thalictrum reniforme	Ranunculaceae	Angiospermae	
Thalictrum reticulatum	Ranunculaceae	Angiospermae	
Thalictrum simplex var. brevipes	Ranunculaceae	Angiospermae	AM
Thalictrum smithii	Ranunculaceae	Angiospermae	
Thalictrum squamiferum	Ranunculaceae	Angiospermae	
Thalictrum trichopus	Ranunculaceae	Angiospermae	
Thalictrum virgatum	Ranunculaceae	Angiospermae	
Trollius asiaticus	Ranunculaceae	Angiospermae	AM
Trollius buddae	Ranunculaceae	Angiospermae	
Trollius chinensis	Ranunculaceae	Angiospermae	AM+NM
Trollius farreri	Ranunculaceae	Angiospermae	
Trollius ledebouri	Ranunculaceae	Angiospermae	

Trollius ranunculoides	Ranunculaceae	Angiospermae	
Urophysa henryi	Ranunculaceae	Angiospermae	
Berchemia floribunda	Rhamnaceae	Angiospermae	
Berchemia kulingensis	Rhamnaceae	Angiospermae	
Berchemia lineata	Rhamnaceae	Angiospermae	
Berchemia polyphylla	Rhamnaceae	Angiospermae	AM
Berchemia polyphylla var. leioclada	Rhamnaceae	Angiospermae	AM
Hovenia acerba	Rhamnaceae	Angiospermae	ECM+NM
Paliurus ramosissimus	Rhamnaceae	Angiospermae	
Rhamnella franguloides	Rhamnaceae	Angiospermae	AM+NM
Rhamnus cathartica	Rhamnaceae	Angiospermae	AM+NM
Rhamnus crenata	Rhamnaceae	Angiospermae	
Rhamnus davurica	Rhamnaceae	Angiospermae	AM+NM
Rhamnus esquirolii var. glabrata	Rhamnaceae	Angiospermae	
Rhamnus frangula	Rhamnaceae	Angiospermae	ECM
Rhamnus globosa	Rhamnaceae	Angiospermae	AM+ECM
Rhamnus leptophylla	Rhamnaceae	Angiospermae	
Rhamnus ussuriensis	Rhamnaceae	Angiospermae	NM
Sageretia henryi	Rhamnaceae	Angiospermae	
Sageretia thea	Rhamnaceae	Angiospermae	AM
Ventilago leiocarpa	Rhamnaceae	Angiospermae	
Ziziphus jujuba	Rhamnaceae	Angiospermae	AM+NM
Ziziphus jujuba var. spinosa	Rhamnaceae	Angiospermae	AM+NM
Ziziphus mauritiana	Rhamnaceae	Angiospermae	AM
Ceriops tagal	Rhizophoraceae	Angiospermae	AM+NM
Rhizophora mucronata	Rhizophoraceae	Angiospermae	AM+NM
Agrimonia pilosa var. nepalensis	Rosaceae	Angiospermae	AM
Amelanchier sinica	Rosaceae	Angiospermae	
Amygdalus communis	Rosaceae	Angiospermae	AM
Amygdalus mongolica	Rosaceae	Angiospermae	AM
Amygdalus nana	Rosaceae	Angiospermae	
Amygdalus pedunculata	Rosaceae	Angiospermae	AM
Amygdalus persica	Rosaceae	Angiospermae	AM

Armeniaca mandshurica	Rosaceae	Angiospermae	
Armeniaca mume	Rosaceae	Angiospermae	AM
Armeniaca sibirica	Rosaceae	Angiospermae	AM
Armeniaca vulgaris	Rosaceae	Angiospermae	AM
Armeniaca vulgaris var. ansu	Rosaceae	Angiospermae	AM
Cerasus dictyoneura	Rosaceae	Angiospermae	
Cerasus humilis	Rosaceae	Angiospermae	AM
Cerasus japonica	Rosaceae	Angiospermae	
Cerasus tomentosa	Rosaceae	Angiospermae	
Chaenomeles cathayensis	Rosaceae	Angiospermae	
Chaenomeles sinensis	Rosaceae	Angiospermae	AM
Chaenomeles speciosa	Rosaceae	Angiospermae	AM
Chamaerhodos erecta	Rosaceae	Angiospermae	AM
Coluria longifolia	Rosaceae	Angiospermae	
Cotoneaster multiflorus	Rosaceae	Angiospermae	AM
Crataegus cuneata	Rosaceae	Angiospermae	AM
Crataegus pinnatifida	Rosaceae	Angiospermae	AM+NM
Crataegus scabrifolia	Rosaceae	Angiospermae	
Crataegus tang-chungchangii	Rosaceae	Angiospermae	
Cydonia oblonga	Rosaceae	Angiospermae	AM
Duchesnea chrysantha	Rosaceae	Angiospermae	AM
Duchesnea indica	Rosaceae	Angiospermae	AM
Eriobotrya deflexa	Rosaceae	Angiospermae	
Eriobotrya japonica	Rosaceae	Angiospermae	AM
Geum aleppicum	Rosaceae	Angiospermae	AM
Kerria japonica	Rosaceae	Angiospermae	AM+ECM+NM
Kerria japonica f. pleniflora	Rosaceae	Angiospermae	AM+ECM+NM
Photinia crassifolia	Rosaceae	Angiospermae	
Photinia glabra	Rosaceae	Angiospermae	AM+ECM+NM
Photinia kwangsiensis	Rosaceae	Angiospermae	
Photinia parvifolia	Rosaceae	Angiospermae	AM
Photinia serrulata var. serrulata	Rosaceae	Angiospermae	AM
Photinia villosa	Rosaceae	Angiospermae	AM

Potentilla anserina	Rosaceae	Angiospermae	AM
Potentilla bifurca	Rosaceae	Angiospermae	AM
Potentilla chinensis	Rosaceae	Angiospermae	NM
Potentilla conferta	Rosaceae	Angiospermae	
Potentilla discolor	Rosaceae	Angiospermae	AM
Potentilla freyniana	Rosaceae	Angiospermae	
Potentilla fruticosa	Rosaceae	Angiospermae	AM
Potentilla fulgens	Rosaceae	Angiospermae	
Potentilla glabra	Rosaceae	Angiospermae	
Potentilla granulosa	Rosaceae	Angiospermae	
Potentilla griffithii var. velutina	Rosaceae	Angiospermae	ECM
Potentilla kleiniana	Rosaceae	Angiospermae	AM
Potentilla leuconota	Rosaceae	Angiospermae	
Potentilla longifolia	Rosaceae	Angiospermae	
Potentilla multifida	Rosaceae	Angiospermae	
Potentilla reptans var. sericophylla	Rosaceae	Angiospermae	AM+ECM
Potentilla tanacetifolia	Rosaceae	Angiospermae	AM
Potentilla yokusaiana	Rosaceae	Angiospermae	
Prinsepia uniflora	Rosaceae	Angiospermae	
Prinsepia utilis	Rosaceae	Angiospermae	AM
Pyrus betulifolia	Rosaceae	Angiospermae	AM
Pyrus ussuriensis	Rosaceae	Angiospermae	AM+ECM+NM
Rhodotypos scandens	Rosaceae	Angiospermae	AM
Rosa banksiae var. normalis	Rosaceae	Angiospermae	AM
Rosa bella	Rosaceae	Angiospermae	
Rosa bracteata	Rosaceae	Angiospermae	
Rosa chinensis	Rosaceae	Angiospermae	AM
Rosa davurica	Rosaceae	Angiospermae	AM+NM
Rosa davurica var. glabra	Rosaceae	Angiospermae	AM+NM
Rosa laevigata	Rosaceae	Angiospermae	AM
Rosa macrophylla	Rosaceae	Angiospermae	
Rosa maximowicziana	Rosaceae	Angiospermae	AM
Rosa multiflora var. cathayensis	Rosaceae	Angiospermae	AM+NM

Rosa omeiensis	Rosaceae	Angiospermae	
Rosa roxburghii	Rosaceae	Angiospermae	AM
Rosa rugosa	Rosaceae	Angiospermae	AM+NM
Rubus alceaefolius	Rosaceae	Angiospermae	
Rubus amphidasys	Rosaceae	Angiospermae	
Rubus buergeri	Rosaceae	Angiospermae	
Rubus chingii	Rosaceae	Angiospermae	
Rubus cochinchinensis	Rosaceae	Angiospermae	
Rubus corchorifolius	Rosaceae	Angiospermae	AM
Rubus coreanus var. coreanus	Rosaceae	Angiospermae	AM
Rubus crataegifolius	Rosaceae	Angiospermae	NM
Rubus delavayi	Rosaceae	Angiospermae	
Rubus doyonensis	Rosaceae	Angiospermae	
Rubus ellipticus var. obcordatus	Rosaceae	Angiospermae	AM
Rubus eucalyptus	Rosaceae	Angiospermae	
Rubus feddei	Rosaceae	Angiospermae	
Rubus hastifolius	Rosaceae	Angiospermae	
Rubus hirsutus	Rosaceae	Angiospermae	
Rubus ichangensis	Rosaceae	Angiospermae	
Rubus idaeus	Rosaceae	Angiospermae	AM+NM
Rubus innominatus	Rosaceae	Angiospermae	
Rubus irenaeus var. irenaeus	Rosaceae	Angiospermae	
Rubus lambertianus	Rosaceae	Angiospermae	AM
Rubus leucanthus	Rosaceae	Angiospermae	
Rubus multibracteatus	Rosaceae	Angiospermae	
Rubus parvifolius	Rosaceae	Angiospermae	AM
Rubus pectinellus	Rosaceae	Angiospermae	
Rubus peltatus	Rosaceae	Angiospermae	AM+NM
Rubus phoenicolasius	Rosaceae	Angiospermae	AM
Rubus pinfaensis	Rosaceae	Angiospermae	
Rubus pirifolius	Rosaceae	Angiospermae	
Rubus pungens	Rosaceae	Angiospermae	AM+ECM
Rubus reflexus	Rosaceae	Angiospermae	

Rubus rosaefolius	Rosaceae	Angiospermae	
Rubus setchuenensis	Rosaceae	Angiospermae	
Rubus sumatranus	Rosaceae	Angiospermae	
Rubus tephrodes	Rosaceae	Angiospermae	
Rubus trianthus	Rosaceae	Angiospermae	AM
Rubus xanthocarpus	Rosaceae	Angiospermae	
Sanguisorba filiformis	Rosaceae	Angiospermae	
Sanguisorba officinalis	Rosaceae	Angiospermae	AM+NM
Sanguisorba officinalis var. longifolia	Rosaceae	Angiospermae	AM+NM
Sanguisorba sitchensis	Rosaceae	Angiospermae	
Sibbaldia procumbens var. aphanopetala	Rosaceae	Angiospermae	AM+NM
Sorbus pohuashanensis	Rosaceae	Angiospermae	AM
Spenceria ramalana	Rosaceae	Angiospermae	
Spiraea blumei	Rosaceae	Angiospermae	AM
Spiraea cantoniensis	Rosaceae	Angiospermae	
Pyrus lindleyi	Rosaceae	Angiospermae	
Rubus otophorus	Rosaceae	Angiospermae	
Rubus rolfei	Rosaceae	Angiospermae	
Adina pilulifera	Rubiaceae	Angiospermae	
Adina rubella	Rubiaceae	Angiospermae	
Canthium horridum	Rubiaceae	Angiospermae	
Catunaregam spinosa	Rubiaceae	Angiospermae	AM
Cephalanthus tetrandrus	*Rubiaceae*	*Angiospermae*	
Cinchona ledgeriana	*Rubiaceae*	*Angiospermae*	AM
Cinchona officinalis	*Rubiaceae*	*Angiospermae*	
Cinchona succirubra	*Rubiaceae*	*Angiospermae*	
Coptosapelta diffusa	Rubiaceae	Angiospermae	
Damnacanthus giganteus	Rubiaceae	Angiospermae	
Damnacanthus indicus	Rubiaceae	Angiospermae	
Diplospora dubia	Rubiaceae	Angiospermae	
Galium aparine	Rubiaceae	Angiospermae	AM+NM
Galium bungei	Rubiaceae	Angiospermae	AM
Galium verum	Rubiaceae	Angiospermae	AM+NM

Gardenia jasminoides	Rubiaceae	Angiospermae	AM+ECM+NM
Gardenia stenophylla	Rubiaceae	Angiospermae	
Hedyotis acutangula	Rubiaceae	Angiospermae	
Hedyotis ampliflora	Rubiaceae	Angiospermae	
Hedyotis auriclaria var. auricularia	Rubiaceae	Angiospermae	
Hedyotis auricularia	Rubiaceae	Angiospermae	AM
Hedyotis corymbosa var. corymbosa	Rubiaceae	Angiospermae	
Hedyotis diffusa	Rubiaceae	Angiospermae	
Hedyotis hedyotidea	Rubiaceae	Angiospermae	
Hedyotis pinifolia	Rubiaceae	Angiospermae	
Hedyotis verticillata	Rubiaceae	Angiospermae	
Hymenodictyon orixense	Rubiaceae	Angiospermae	AM
Knoxia corymbosa	Rubiaceae	Angiospermae	
Knoxia valerianoides	Rubiaceae	Angiospermae	
Morinda citrifolia	Rubiaceae	Angiospermae	AM
Morinda citrina var. chlorina	Rubiaceae	Angiospermae	
Morinda officinalis	Rubiaceae	Angiospermae	
Morinda parvifolia	Rubiaceae	Angiospermae	
Morinda pubiofficinalis	Rubiaceae	Angiospermae	
Morinda scabrifolia	Rubiaceae	Angiospermae	
Morinda shuanghuaensis	Rubiaceae	Angiospermae	
Mussaenda erosa	Rubiaceae	Angiospermae	
Mussaenda pubescens	Rubiaceae	Angiospermae	AM
Nauclea officinalis	Rubiaceae	Angiospermae	
Neohymenopogon oligocarpus	Rubiaceae	Angiospermae	
Neohymenopogon parasiticus	Rubiaceae	Angiospermae	
Ophiorrhiza calcarata	Rubiaceae	Angiospermae	
Pavetta hongkongensis	Rubiaceae	Angiospermae	AM+NM
Psychotria calocarpa	Rubiaceae	Angiospermae	AM
Psychotria prainii	Rubiaceae	Angiospermae	NM
Psychotria rubra	Rubiaceae	Angiospermae	
Psychotria serpens	Rubiaceae	Angiospermae	AM+NM
Rubia manjith	Rubiaceae	Angiospermae	

Rubia oncotricha	Rubiaceae	Angiospermae	
Schizomussaenda dehiscens	Rubiaceae	Angiospermae	
Tarenna attenuata	Rubiaceae	Angiospermae	
Tarenna depauperata	Rubiaceae	Angiospermae	
Tarenna mollissima	Rubiaceae	Angiospermae	NM
Uncaria macrophylla	Rubiaceae	Angiospermae	
Uncaria rhynchophylla	Rubiaceae	Angiospermae	
Uncaria yunnanensis	Rubiaceae	Angiospermae	
Wendlandia uvariifolia	Rubiaceae	Angiospermae	AM
Luculia intermedia	Rubiaceae	Angiospermae	
Luculia intermedia var. pinciana	Rubiaceae	Angiospermae	
Aegle marmelos	Rutaceae	Angiospermae	AM+NM
Boenninghausenia albiflora	Rutaceae	Angiospermae	AM
Boenninghausenia sessilicarpa	Rutaceae	Angiospermae	AM
Citrus aurantium	Rutaceae	Angiospermae	AM
Citrus medica var. sarcodactylis	Rutaceae	Angiospermae	AM
Clausena anisum-olens	Rutaceae	Angiospermae	
Clausena emarginata	Rutaceae	Angiospermae	
Clausena excavata	Rutaceae	Angiospermae	
Evodia lepta	Rutaceae	Angiospermae	AM
Evodia rutaecarpa	Rutaceae	Angiospermae	
Evodia trichotoma	Rutaceae	Angiospermae	
Fortunella hindsii	Rutaceae	Angiospermae	AM
Glycosmis parviflora	Rutaceae	Angiospermae	
Micromelum falcatum	Rutaceae	Angiospermae	
Micromelum integerrimum	Rutaceae	Angiospermae	AM
Murraya kwangsiensis var. kwangsiensis	Rutaceae	Angiospermae	
Orixa japonica	Rutaceae	Angiospermae	AM+NM
Phellodendron amurense	Rutaceae	Angiospermae	AM+NM
Psilopeganum sinense	Rutaceae	Angiospermae	
Ptelea trifoliata	Rutaceae	Angiospermae	AM
Ruta graveolens	Rutaceae	Angiospermae	AM
Skimmia reevesiana	Rutaceae	Angiospermae	

Zanthoxylum acanthopodium var. timbor	Rutaceae	Angiospermae	
Zanthoxylum ailanthoides var. ailanthoides	Rutaceae	Angiospermae	AM
Zanthoxylum armatum var. armatum	Rutaceae	Angiospermae	AM
Zanthoxylum austrosinense var. austrosinense	Rutaceae	Angiospermae	
Zanthoxylum dissitum var. dissitum	Rutaceae	Angiospermae	
Zanthoxylum echinocarpum var. echinocarpum	Rutaceae	Angiospermae	AM
Zanthoxylum laetum	Rutaceae	Angiospermae	
Zanthoxylum myriacanthum	Rutaceae	Angiospermae	
Zanthoxylum ovalifolium var. ovalifolium	Rutaceae	Angiospermae	
Meliosma cuneifolia	Sabiaceae	Angiospermae	
Meliosma fordii	Sabiaceae	Angiospermae	
Sabia japonica	Sabiaceae	Angiospermae	AM
Sabia schumanniana	Sabiaceae	Angiospermae	
Populus × canadensis	Salicaceae	Angiospermae	AM+ECM+NM
Populus davidiana	Salicaceae	Angiospermae	AM+ECM+NM
Populus nigra	Salicaceae	Angiospermae	AM+ECM+NM
Populus tomentosa	Salicaceae	Angiospermae	AM+ECM
Salix matsudana	Salicaceae	Angiospermae	AM+ECM
Salix wallichiana	Salicaceae	Angiospermae	
Buckleya lanceolate	Santalaceae	Angiospermae	
Dendrotrophe frutescens	Santalaceae	Angiospermae	
Osyris wightiana	Santalaceae	Angiospermae	
Pyrularia edulis	Santalaceae	Angiospermae	
Santalum album	Santalaceae	Angiospermae	AM+NM
Thesium chinense var. chinense	Santalaceae	Angiospermae	AM
Allophylus viridis	Sapindaceae	Angiospermae	
Cardiospermum halicacabum	Sapindaceae	Angiospermae	AM+NM
Dimocarpus longan	Sapindaceae	Angiospermae	AM+NM
Dimocarpus longan var. obtusus	Sapindaceae	Angiospermae	AM+NM
Erioglossum rubiginosum	Sapindaceae	Angiospermae	
Koelreuteria bipinnata	Sapindaceae	Angiospermae	NM
Koelreuteria paniculata	Sapindaceae	Angiospermae	AM
Nephelium chryseum	Sapindaceae	Angiospermae	AM

Sapindus delavayi	Sapindaceae	Angiospermae	
Sapindus mukorossi	Sapindaceae	Angiospermae	AM
Sapindus rarak	Sapindaceae	Angiospermae	
Melicoccus bijugatus	Sapindaceae	Angiospermae	
Chrysophyllum lanceolatum var. stellatocarpon	Sapotaceae	Angiospermae	AM
Manilkara hexandra	Sapotaceae	Angiospermae	AM
Manilkara zapota	Sapotaceae	Angiospermae	AM
Planchonella obovata	Sapotaceae	Angiospermae	AM
Pouteria annamensis	Sapotaceae	Angiospermae	
Gymnotheca chinensis	Saururaceae	Angiospermae	
Houttuynia cordata	Saururaceae	Angiospermae	AM
Saururus chinensis	Saururaceae	Angiospermae	
Astilbe chinensis	Saxifragaceae	Angiospermae	
Astilbe grandis	Saxifragaceae	Angiospermae	
Astilbe rivularis	Saxifragaceae	Angiospermae	
Astilbe rivularis var. myriantha	Saxifragaceae	Angiospermae	
Bergenia purpurascens	Saxifragaceae	Angiospermae	
Bergenia scopulosa	Saxifragaceae	Angiospermae	
Chrysosplenium axillare	Saxifragaceae	Angiospermae	
Chrysosplenium macrophyllum	Saxifragaceae	Angiospermae	
Chrysosplenium nudicaule	Saxifragaceae	Angiospermae	
Dichroa febrifuga	Saxifragaceae	Angiospermae	
Hydrangea anomala	Saxifragaceae	Angiospermae	
Hydrangea macrophylla	Saxifragaceae	Angiospermae	AM
Hydrangea petiolaris	Saxifragaceae	Angiospermae	
Penthorum chinense	Saxifragaceae	Angiospermae	
Rodgersia sambucifolia	Saxifragaceae	Angiospermae	
Saxifraga aculeata	Saxifragaceae	Angiospermae	
Saxifraga atrata	Saxifragaceae	Angiospermae	
Saxifraga brunonis	Saxifragaceae	Angiospermae	
Saxifraga divaricata	Saxifragaceae	Angiospermae	
Saxifraga gemmuligera	Saxifragaceae	Angiospermae	
Saxifraga melanocentra	Saxifragaceae	Angiospermae	

Saxifraga montana	Saxifragaceae	Angiospermae	
Saxifraga nangxianensis	Saxifragaceae	Angiospermae	
Saxifraga saginoides	Saxifragaceae	Angiospermae	
Saxifraga sanguinea	Saxifragaceae	Angiospermae	
Saxifraga signatella	Saxifragaceae	Angiospermae	
Saxifraga stolonifera	Saxifragaceae	Angiospermae	
Saxifraga tangutica	Saxifragaceae	Angiospermae	
Saxifraga umbellulata	Saxifragaceae	Angiospermae	
Saxifraga umbellulata var. pectinata	Saxifragaceae	Angiospermae	
Saxifraga unguiculata	Saxifragaceae	Angiospermae	
Tiarella polyphylla	Saxifragaceae	Angiospermae	AM
Bacopa monnieri	Scrophulariaceae	Angiospermae	AM+ECM+NM
Centranthera cochinchinensis var. nepalensis	Scrophulariaceae	Angiospermae	AM
Linaria vulgaris subsp. sinensis	Scrophulariaceae	Angiospermae	AM+NM
Lindernia anagallis	Scrophulariaceae	Angiospermae	
Lindernia antipoda	Scrophulariaceae	Angiospermae	NM
Lindernia crustacea	Scrophulariaceae	Angiospermae	
Lindernia ruellioides	Scrophulariaceae	Angiospermae	
Lindernia viscosa	Scrophulariaceae	Angiospermae	
Mimulus tenellus var. platyphyllus	Scrophulariaceae	Angiospermae	
Picrorhiza scrophulariiflora	Scrophulariaceae	Angiospermae	
Scrophularia amugensis	Scrophulariaceae	Angiospermae	
Scrophularia buergeriana var. tsinglingensis	Scrophulariaceae	Angiospermae	
Scrophularia macrocarpa	Scrophulariaceae	Angiospermae	
Scrophularia mandshurica	Scrophulariaceae	Angiospermae	
Scrophularia ningpoensis	Scrophulariaceae	Angiospermae	AM
Striga asiatica	Scrophulariaceae	Angiospermae	AM+NM
Veronica ciliata subsp. cephaloides	Scrophulariaceae	Angiospermae	
Veronica linariifolia subsp. dilatata	Scrophulariaceae	Angiospermae	AM
Veronica peregrina	Scrophulariaceae	Angiospermae	AM
Veronicastrum axillare	Scrophulariaceae	Angiospermae	AM
Veronicastrum caulopterum	Scrophulariaceae	Angiospermae	
Veronicastrum villosulum	Scrophulariaceae	Angiospermae	

Veronicastrum villosulum var. glabrum	Scrophulariaceae	Angiospermae	
Ailanthus altissima	Simaroubaceae	Angiospermae	AM+ECM+NM
Brucea javanica	Simaroubaceae	Angiospermae	AM
Harrisonia perforata	Simaroubaceae	Angiospermae	
Anisodus acutangulus	Solanaceae	Angiospermae	
Lycium barbarum	Solanaceae	Angiospermae	AM
Lycium chinense	Solanaceae	Angiospermae	AM
Lycium flexicaule	Solanaceae	Angiospermae	
Solanum aviculare	Solanaceae	Angiospermae	
Solanum cathayanum	Solanaceae	Angiospermae	
Solanum dulcamara	Solanaceae	Angiospermae	AM+NM
Solanum ferox	Solanaceae	Angiospermae	
Solanum indicum	Solanaceae	Angiospermae	AM
Solanum lyratum	Solanaceae	Angiospermae	AM
Solanum melongena	Solanaceae	Angiospermae	AM+NM
Solanum melongena var. esculentum	Solanaceae	Angiospermae	AM+NM
Solanum melongena var. serpentinum	Solanaceae	Angiospermae	AM+NM
Solanum nigrum	*Solanaceae*	*Angiospermae*	AM+NM
Solanum photeinocarpum	*Solanaceae*	*Angiospermae*	AM
Solanum spirale	*Solanaceae*	*Angiospermae*	
Solanum surattense	Solanaceae	Angiospermae	AM
Solanum torvum	Solanaceae	Angiospermae	NM
Solanum verbascifolium	Solanaceae	Angiospermae	AM
Sparganium simplex	Sparganiaceae	Angiospermae	
Sparganium stoloniferum	Sparganiaceae	Angiospermae	AM
Stachyurus himalaicus	Stachyuraceae	Angiospermae	
Stachyurus obovatus	Stachyuraceae	Angiospermae	
Euscaphis japonica	Staphyleaceae	Angiospermae	AM
Croomia japonica	Stemonaceae	Angiospermae	AM
Stemona japonica	Stemonaceae	Angiospermae	AM
Stemona parviflora	Stemonaceae	Angiospermae	
Stemona sessilifolia	Stemonaceae	Angiospermae	
Stemona tuberosa	Stemonaceae	Angiospermae	

Ambroma augusta	Sterculiaceae	Angiospermae	
Firmiana platanifolia	Sterculiaceae	Angiospermae	AM
Helicteres angustifolia	Sterculiaceae	Angiospermae	
Helicteres elongata	Sterculiaceae	Angiospermae	
Helicteres isora	Sterculiaceae	Angiospermae	AM
Pterospermum heterophyllum	Sterculiaceae	Angiospermae	AM
Sterculia villosa	Sterculiaceae	Angiospermae	
Symplocos chinensis	Symplocaceae	Angiospermae	
Symplocos congesta	Symplocaceae	Angiospermae	AM
Symplocos glauca	Symplocaceae	Angiospermae	AM
Symplocos lancifolia	Symplocaceae	Angiospermae	AM
Symplocos laurina	Symplocaceae	Angiospermae	AM+ECM
Symplocos paniculata	Symplocaceae	Angiospermae	AM
Symplocos poilanei	Symplocaceae	Angiospermae	
Symplocos racemosa	Symplocaceae	Angiospermae	AM
Symplocos sumuntia	Symplocaceae	Angiospermae	ECM
Symplocos terminalis	Symplocaceae	Angiospermae	
Schizocapsa plantaginea	Taccaceae	Angiospermae	
Tacca chantrieri	Taccaceae	Angiospermae	NM
Tamarix chinensis	Tamaricaceae	Angiospermae	AM
Eurya japonica	Theaceae	Angiospermae	AM
Eurya obtusifolia	Theaceae	Angiospermae	
Aquilaria sinensis	Thymelaeaceae	Angiospermae	AM
Aquilaria yunnanensis	Thymelaeaceae	Angiospermae	
Daphne feddei	Thymelaeaceae	Angiospermae	
Daphne genkwa	Thymelaeaceae	Angiospermae	
Daphne giraldii	Thymelaeaceae	Angiospermae	
Daphne jinyunensis var. ptilostyla	Thymelaeaceae	Angiospermae	
Edgeworthia chrysantha	Thymelaeaceae	Angiospermae	
Stellera chamaejasme	Thymelaeaceae	Angiospermae	AM
Wikstroemia chamaedaphne	Thymelaeaceae	Angiospermae	
Wikstroemia indica	Thymelaeaceae	Angiospermae	
Wikstroemia micrantha	Thymelaeaceae	Angiospermae	

Wikstroemia nutans	Thymelaeaceae	Angiospermae	AM
Corchorus aestuans	Tiliaceae	Angiospermae	AM
Microcos paniculata	Tiliaceae	Angiospermae	AM
Triumfetta rhomboidea	Tiliaceae	Angiospermae	
Typha angustifolia	Typhaceae	Angiospermae	AM+NM
Typha latifolia	Typhaceae	Angiospermae	AM+NM
Typha laxmannii	Typhaceae	Angiospermae	
Typha orientalis	Typhaceae	Angiospermae	AM+NM
Typha przewalskii	Typhaceae	Angiospermae	
Gironniera subaequalis	Ulmaceae	Angiospermae	NM
Ulmus gaussenii	Ulmaceae	Angiospermae	NM
Ulmus macrocarpa	Ulmaceae	Angiospermae	ECM
Ulmus parvifolia	Ulmaceae	Angiospermae	AM+ECM+NM
Ulmus pumila	Ulmaceae	Angiospermae	AM+ECM+NM
Ulmus tonkinensis	Ulmaceae	Angiospermae	
Zelkova serrata	Ulmaceae	Angiospermae	AM
Ammi visnaga	Umbelliferae	Angiospermae	
Anethum graveolens	Umbelliferae	Angiospermae	AM
Angelica acutiloba	Umbelliferae	Angiospermae	
Angelica anomala	Umbelliferae	Angiospermae	
Angelica biserrata	Umbelliferae	Angiospermae	
Angelica dahurica	Umbelliferae	Angiospermae	AM
Angelica laxifoliata	Umbelliferae	Angiospermae	
Angelica megaphylla	Umbelliferae	Angiospermae	
Angelica morii	Umbelliferae	Angiospermae	
Angelica nitida	Umbelliferae	Angiospermae	
Angelica sinensis	Umbelliferae	Angiospermae	AM
Anthriscus sylvestris	Umbelliferae	Angiospermae	AM+NM
Bupleurum bicaule	Umbelliferae	Angiospermae	
Bupleurum candollei	Umbelliferae	Angiospermae	
Bupleurum chaishoui	Umbelliferae	Angiospermae	
Bupleurum chinense	Umbelliferae	Angiospermae	AM
Bupleurum microcephalum	Umbelliferae	Angiospermae	

Bupleurum scorzonerifolium	Umbelliferae	Angiospermae	AM
Bupleurum wenchuanense	Umbelliferae	Angiospermae	
Bupleurum yinchowense	Umbelliferae	Angiospermae	
Centella asiatica	Umbelliferae	Angiospermae	AM+NM
Changium smyrnioides	Umbelliferae	Angiospermae	
Chuanminshen violaceum	Umbelliferae	Angiospermae	
Cnidium monnieri	Umbelliferae	Angiospermae	
Conium maculatum	Umbelliferae	Angiospermae	AM+NM
Coriandrum sativum	Umbelliferae	Angiospermae	AM
Cryptotaenia japonica	Umbelliferae	Angiospermae	NM
Cuminum cyminum	Umbelliferae	Angiospermae	
Daucus carota	Umbelliferae	Angiospermae	AM+NM
Eryngium foetidum	Umbelliferae	Angiospermae	
Ferula bungeana	Umbelliferae	Angiospermae	
Ferula ferulaeoides	Umbelliferae	Angiospermae	AM
Ferula fukanensis	Umbelliferae	Angiospermae	ORM
Ferula lehmannii	Umbelliferae	Angiospermae	AM
Ferula olivacea	Umbelliferae	Angiospermae	
Ferula sinkiangensis	Umbelliferae	Angiospermae	ORM
Foeniculum vulgare	Umbelliferae	Angiospermae	AM
Glehnia littoralis	Umbelliferae	Angiospermae	AM
Heracleum barmanicum	Umbelliferae	Angiospermae	
Heracleum bivittatum	Umbelliferae	Angiospermae	
Heracleum dissectifolium	Umbelliferae	Angiospermae	
Heracleum hemsleyanum	Umbelliferae	Angiospermae	
Heracleum scabridum	Umbelliferae	Angiospermae	
Heracleum stenopterum	Umbelliferae	Angiospermae	
Heracleum tiliifolium	Umbelliferae	Angiospermae	
Hydrocotyle chinensis	Umbelliferae	Angiospermae	
Hydrocotyle nepalensis	Umbelliferae	Angiospermae	
Hydrocotyle sibthorpioides	Umbelliferae	Angiospermae	NM
Hydrocotyle sibthorpioides var. batrachium	Umbelliferae	Angiospermae	NM
Levisticum officinale	Umbelliferae	Angiospermae	AM

Libanotis buchtormensis	Umbelliferae	Angiospermae	
Ligusticum chuanxiong	Umbelliferae	Angiospermae	AM
Ligusticum jeholense	Umbelliferae	Angiospermae	
Ligusticum sinense	Umbelliferae	Angiospermae	
Ligusticum tenuissimum	Umbelliferae	Angiospermae	
Nothosmyrnium japonicum	Umbelliferae	Angiospermae	
Nothosmyrnium japonicum var. sutchuensis	Umbelliferae	Angiospermae	
Notopterygium forbesii var. oviforme	Umbelliferae	Angiospermae	
Oenanthe javanica	Umbelliferae	Angiospermae	AM+NM
Osmorhiza aristata var. laxa	Umbelliferae	Angiospermae	
Ostericum citriodorum	Umbelliferae	Angiospermae	
Ostericum grosseserratum	Umbelliferae	Angiospermae	
Ostericum sieboldii	Umbelliferae	Angiospermae	
Peucedanum medicum	Umbelliferae	Angiospermae	
Peucedanum praeruptorum	Umbelliferae	Angiospermae	AM
Peucedanum wawrae	Umbelliferae	Angiospermae	
Pimpinella thellungiana	Umbelliferae	Angiospermae	
Pleurospermum giraldii	Umbelliferae	Angiospermae	
Pternopetalum botrychioides	Umbelliferae	Angiospermae	
Pternopetalum leptophyllum	Umbelliferae	Angiospermae	
Pternopetalum vulgare	Umbelliferae	Angiospermae	
Pternopetalum vulgare var. acuminatum	Umbelliferae	Angiospermae	
Pternopetalum vulgare var. strigosum	Umbelliferae	Angiospermae	
Sanicula astrantiifolia	Umbelliferae	Angiospermae	
Sanicula coerulescens	Umbelliferae	Angiospermae	
Sanicula elata	Umbelliferae	Angiospermae	
Sanicula lamelligera	Umbelliferae	Angiospermae	
Sanicula orthacantha	Umbelliferae	Angiospermae	
Sanicula orthacantha var. brevispina	Umbelliferae	Angiospermae	
Saposhnikovia divaricata	Umbelliferae	Angiospermae	AM
Seseli squarrulosum	Umbelliferae	Angiospermae	
Seselopsis tianschanicum	Umbelliferae	Angiospermae	
Sinodielsia yunnanensis	Umbelliferae	Angiospermae	

Sium suave	Umbelliferae	Angiospermae	
Torilis japonica	Umbelliferae	Angiospermae	AM
Vicatia thibetica	Umbelliferae	Angiospermae	
Boehmeria clidemioides var. diffusa	Urticaceae	Angiospermae	
Boehmeria densiglomerata	Urticaceae	Angiospermae	
Boehmeria gracilis	Urticaceae	Angiospermae	
Boehmeria longispica	Urticaceae	Angiospermae	
Boehmeria macrophylla	Urticaceae	Angiospermae	
Boehmeria macrophylla var. scabrella	Urticaceae	Angiospermae	
Boehmeria nivea	Urticaceae	Angiospermae	AM
Boehmeria nivea var. nipononivea	Urticaceae	Angiospermae	AM
Boehmeria nivea var. tenacissima	Urticaceae	Angiospermae	AM
Boehmeria penduliflora	Urticaceae	Angiospermae	
Boehmeria siamensis	Urticaceae	Angiospermae	
Boehmeria tricuspis	Urticaceae	Angiospermae	
Chamabainia cuspidata	Urticaceae	Angiospermae	
Elatostema brachyodontum	Urticaceae	Angiospermae	
Elatostema cyrtandrifolium	Urticaceae	Angiospermae	
Elatostema ichangense	Urticaceae	Angiospermae	
Elatostema involucratum	Urticaceae	Angiospermae	
Elatostema retrohirtum	Urticaceae	Angiospermae	
Elatostema schizocephalum	Urticaceae	Angiospermae	
Elatostema stewardii	Urticaceae	Angiospermae	
Girardinia suborbiculata subsp. triloba	Urticaceae	Angiospermae	
Gonostegia hirta	Urticaceae	Angiospermae	AM
Laportea cuspidata	Urticaceae	Angiospermae	
Laportea violacea	Urticaceae	Angiospermae	
Nanocnide lobata	Urticaceae	Angiospermae	
Oreocnide frutescens	Urticaceae	Angiospermae	NM
Oreocnide obovata var. paradoxa	Urticaceae	Angiospermae	
Parietaria micrantha	Urticaceae	Angiospermae	
Pellionia radicans	Urticaceae	Angiospermae	
Pilea cavaleriei	Urticaceae	Angiospermae	

Pilea japonica	Urticaceae	Angiospermae	
Pilea longicaulis	Urticaceae	Angiospermae	
Pilea notata	Urticaceae	Angiospermae	AM
Pilea peploides var. major	Urticaceae	Angiospermae	
Pilea plataniflora	Urticaceae	Angiospermae	
Pilea pumila	Urticaceae	Angiospermae	AM+NM
Pilea swinglei	Urticaceae	Angiospermae	
Pilea verrucosa	Urticaceae	Angiospermae	
Urtica angustifolia	Urticaceae	Angiospermae	
Urtica cannabina	Urticaceae	Angiospermae	
Urtica fissa	Urticaceae	Angiospermae	AM
Urtica laetevirens subsp. laetevirens	Urticaceae	Angiospermae	
Urtica triangularis	Urticaceae	Angiospermae	
Urtica triangularis subsp. pinnatifida	Urticaceae	Angiospermae	
Patrinia heterophylla	Valerianaceae	Angiospermae	AM
Patrinia monandra	Valerianaceae	Angiospermae	
Patrinia punctiflora	Valerianaceae	Angiospermae	
Patrinia punctiflora var. robusta	Valerianaceae	Angiospermae	
Patrinia scabiosaefolia	Valerianaceae	Angiospermae	AM
Patrinia villosa	Valerianaceae	Angiospermae	AM
Valeriana flagellifera	Valerianaceae	Angiospermae	
Valeriana jatamansi	Valerianaceae	Angiospermae	AM
Valeriana officinalis	Valerianaceae	Angiospermae	AM+NM
Avicennia marina	Verbenaceae	Angiospermae	AM+NM
Callicarpa arborea	Verbenaceae	Angiospermae	
Callicarpa bodinieri	Verbenaceae	Angiospermae	
Callicarpa cathayana	Verbenaceae	Angiospermae	AM
Callicarpa dichotoma	Verbenaceae	Angiospermae	
Callicarpa formosana	Verbenaceae	Angiospermae	
Callicarpa giraldii	Verbenaceae	Angiospermae	
Callicarpa kochiana	Verbenaceae	Angiospermae	
Callicarpa longissima	Verbenaceae	Angiospermae	
Callicarpa macrophylla	Verbenaceae	Angiospermae	

Callicarpa nudiflora	Verbenaceae	Angiospermae	
Callicarpa rubella	Verbenaceae	Angiospermae	
Callicarpa rubella var. rubella f. crenata	Verbenaceae	Angiospermae	
Callicarpa yunnanensis	Verbenaceae	Angiospermae	
Caryopteris incana	Verbenaceae	Angiospermae	
Caryopteris mongholica	Verbenaceae	Angiospermae	AM
Caryopteris nepetaefolia	Verbenaceae	Angiospermae	
Caryopteris paniculata	Verbenaceae	Angiospermae	
Caryopteris terniflora	Verbenaceae	Angiospermae	
Clerodendrum bungei	Verbenaceae	Angiospermae	
Clerodendrum canescens	Verbenaceae	Angiospermae	
Clerodendrum cyrtophyllum	Verbenaceae	Angiospermae	AM
Clerodendrum fortunatum	Verbenaceae	Angiospermae	
Clerodendrum indicum	Verbenaceae	Angiospermae	
Clerodendrum inerme	Verbenaceae	Angiospermae	AM
Clerodendrum japonicum	Verbenaceae	Angiospermae	AM
Clerodendrum kwangtungense	Verbenaceae	Angiospermae	
Clerodendrum lindleyi	Verbenaceae	Angiospermae	
Clerodendrum mandarinorum	Verbenaceae	Angiospermae	
Clerodendrum philippinum	Verbenaceae	Angiospermae	
Clerodendrum philippinum var. simplex	Verbenaceae	Angiospermae	
Clerodendrum serratum	Verbenaceae	Angiospermae	
Clerodendrum serratum var. amplexifolium	Verbenaceae	Angiospermae	
Clerodendrum serratum var. herbaceum	Verbenaceae	Angiospermae	
Clerodendrum serratum var. wallichii	Verbenaceae	Angiospermae	
Clinacanthus nutans	Verbenaceae	Angiospermae	
Duranta repens	Verbenaceae	Angiospermae	AM
Lantana camara	Verbenaceae	Angiospermae	AM
Phyla nodiflora	Verbenaceae	Angiospermae	AM
Premna crassa	Verbenaceae	Angiospermae	
Premna henryana	Verbenaceae	Angiospermae	
Premna ligustroides	Verbenaceae	Angiospermae	
Premna microphylla	Verbenaceae	Angiospermae	

Premna puberula	Verbenaceae	Angiospermae	
Premna sunyiensis	Verbenaceae	Angiospermae	
Premna szemaoensis	Verbenaceae	Angiospermae	
Premna urticifolia	Verbenaceae	Angiospermae	
Pygmaeopremna herbacea	Verbenaceae	Angiospermae	
Sphenodesme pentandra var. pentandra	Verbenaceae	Angiospermae	
Stachytarpheta jamaicensis	Verbenaceae	Angiospermae	AM
Tectona grandis	Verbenaceae	Angiospermae	AM+NM
Verbena officinalis	Verbenaceae	Angiospermae	AM
Vitex canescens	Verbenaceae	Angiospermae	
Vitex negundo	Verbenaceae	Angiospermae	AM+NM
Vitex negundo var. cannabifolia	Verbenaceae	Angiospermae	AM+NM
Vitex negundo var. heterophylla	Verbenaceae	Angiospermae	AM+NM
Vitex peduncularis	Verbenaceae	Angiospermae	
Vitex quinata var. puberula	Verbenaceae	Angiospermae	AM
Vitex trifolia	Verbenaceae	Angiospermae	AM
Vitex trifolia var. simplicifolia	Verbenaceae	Angiospermae	AM
Viola acuminata	Violaceae	Angiospermae	NM
Viola acuminata var. pilifera	Violaceae	Angiospermae	NM
Viola betonicifolia	Violaceae	Angiospermae	
Viola betonicifolia subsp. jaunsariensis	Violaceae	Angiospermae	
Viola biflora	Violaceae	Angiospermae	AM
Viola collina	Violaceae	Angiospermae	AM
Viola delavayi	Violaceae	Angiospermae	
Viola diffusa	Violaceae	Angiospermae	AM
Viola diffusa var. brevibarbata	Violaceae	Angiospermae	AM
Viola grypoceras	Violaceae	Angiospermae	AM
Viola inconspicua	Violaceae	Angiospermae	
Viola mandshurica	Violaceae	Angiospermae	AM
Viola moupinensis	Violaceae	Angiospermae	
Viola patrinii	Violaceae	Angiospermae	AM
Viola philippica	Violaceae	Angiospermae	AM
Viola prioantha	Violaceae	Angiospermae	

Viola prionantha var. prionantha	Violaceae	Angiospermae	
Viola rockiana	Violaceae	Angiospermae	
Viola verecunda	Violaceae	Angiospermae	
Ampelopsis heterophylla var. brevipedunculata	Vitaceae	Angiospermae	AM
Ampelopsis japonica	Vitaceae	Angiospermae	
Cayratia corniculata	Vitaceae	Angiospermae	
Cayratia japonica	Vitaceae	Angiospermae	AM
Parthenocissus tricuspidata	Vitaceae	Angiospermae	AM
Tetrastigma hemsleyanum	Vitaceae	Angiospermae	AM
Tetrastigma obtectum	Vitaceae	Angiospermae	
Tetrastigma planicaule	Vitaceae	Angiospermae	AM
Vitis balanseana	Vitaceae	Angiospermae	
Vitis davidii	Vitaceae	Angiospermae	
Vitis flexuosa	Vitaceae	Angiospermae	AM+ECM+ERM
Vitis romanetii Romanet du Caillaud	Vitaceae	Angiospermae	
Alpinia blepharocalyx	Zingiberaceae	Angiospermae	
Alpinia chinensis	Zingiberaceae	Angiospermae	NM
Alpinia conchigera	Zingiberaceae	Angiospermae	AM
Alpinia galanga	Zingiberaceae	Angiospermae	AM+NM
Alpinia japonica	Zingiberaceae	Angiospermae	AM
Alpinia nigra	Zingiberaceae	Angiospermae	AM
Alpinia officinarum	Zingiberaceae	Angiospermae	AM
Alpinia oxyphylla	Zingiberaceae	Angiospermae	
Alpinia psilogyna	Zingiberaceae	Angiospermae	
Alpinia stachyoides	Zingiberaceae	Angiospermae	
Alpinia zerumbet	Zingiberaceae	Angiospermae	AM
Amomum aurantiacum	Zingiberaceae	Angiospermae	
Amomum compactum	Zingiberaceae	Angiospermae	
Amomum kravanh	Zingiberaceae	Angiospermae	
Amomum kwangsiense	Zingiberaceae	Angiospermae	
Amomum maximum	Zingiberaceae	Angiospermae	AM
Amomum microcarpum	Zingiberaceae	Angiospermae	
Amomum muricarpum	Zingiberaceae	Angiospermae	

Amomum subulatum	Zingiberaceae	Angiospermae	AM
Amomum thyrsoideum	Zingiberaceae	Angiospermae	
Amomum tsaoko	Zingiberaceae	Angiospermae	
Amomum villosum	Zingiberaceae	Angiospermae	AM
Amomum villosum var. nanum	Zingiberaceae	Angiospermae	AM
Amomum villosum var. xanthioides	Zingiberaceae	Angiospermae	AM
Caulokaempferia coenobialis	Zingiberaceae	Angiospermae	
Costus speciosus	Zingiberaceae	Angiospermae	AM
Costus tonkinensis	Zingiberaceae	Angiospermae	AM
Curcuma aromatica	Zingiberaceae	Angiospermae	AM
Curcuma caesia	Zingiberaceae	Angiospermae	AM
Curcuma elata	Zingiberaceae	Angiospermae	AM
Curcuma kwangsiensis	Zingiberaceae	Angiospermae	
Curcuma longa	Zingiberaceae	Angiospermae	AM
Curcuma viridiflora	Zingiberaceae	Angiospermae	
Curcuma zedoaria	Zingiberaceae	Angiospermae	AM
Hedychium coronarium	Zingiberaceae	Angiospermae	AM
Hedychium spicatum	Zingiberaceae	Angiospermae	
Hedychium villosum	Zingiberaceae	Angiospermae	
Kaempferia rotunda	Zingiberaceae	Angiospermae	AM
Stahlianthus involucratus	Zingiberaceae	Angiospermae	
Zingiber corallinum	Zingiberaceae	Angiospermae	
Zingiber mioga	Zingiberaceae	Angiospermae	AM
Zingiber officinale	Zingiberaceae	Angiospermae	AM
Zingiber zerumbet	Zingiberaceae	Angiospermae	AM
Nitraria sibirica	Zygophyllaceae	Angiospermae	NM
Peganum harmala	Zygophyllaceae	Angiospermae	AM+NM
Peganum nigellastrum	Zygophyllaceae	Angiospermae	AM
Tribulus terrester	Zygophyllaceae	Angiospermae	AM
Agathis dammara	Araucariaceae	Gymnospermae	AM
Cephalotaxus fortunei	Cephalotaxaceae	Gymnospermae	AM+ECM
Cephalotaxus hainanensis	Cephalotaxaceae	Gymnospermae	AM
Cephalotaxus lanceolata	Cephalotaxaceae	Gymnospermae	

Cephalotaxus mannii	Cephalotaxaceae	Gymnospermae	
Cephalotaxus oliveri	Cephalotaxaceae	Gymnospermae	
Cephalotaxus sinensis	Cephalotaxaceae	Gymnospermae	AM
Juniperus communis	Cupressaceae	Gymnospermae	AM+ECM+NM
Juniperus rigida	Cupressaceae	Gymnospermae	AM+ECM+NM
Platycladus orientalis	Cupressaceae	Gymnospermae	AM+ECM+NM
Sabina chinensis	Cupressaceae	Gymnospermae	ECM
Cycas revoluta	Cycadaceae	Gymnospermae	AM
Ephedra equisetina	Ephedraceae	Gymnospermae	AM
Ephedra intermedia	Ephedraceae	Gymnospermae	AM
Ephedra likiangensis	Ephedraceae	Gymnospermae	
Ephedra minuta	Ephedraceae	Gymnospermae	
Ephedra monosperma	Ephedraceae	Gymnospermae	AM
Ephedra sinica	Ephedraceae	Gymnospermae	AM
Ginkgo biloba	Ginkgoaceae	Gymnospermae	AM+NM
Pinus koraiensis	Pinaceae	Gymnospermae	ECM+NM
Pinus massoniana	Pinaceae	Gymnospermae	AM+ECM+NM
Pinus massoniana var. hainanensis	Pinaceae	Gymnospermae	AM+ECM+NM
Pinus tabulaeformis	Pinaceae	Gymnospermae	AM+ECM+NM
Pseudolarix amabilis	Pinaceae	Gymnospermae	
Taxus cuspidata	Taxaceae	Gymnospermae	AM
Adiantum capillus-veneris f. dissectum	Adiantaceae	Pteridophyta	AM+NM
Adiantum flabellulatum	Adiantaceae	Pteridophyta	AM+NM
Adiantum pedatum	Adiantaceae	Pteridophyta	AM+NM
Angiopteris officinalis	Angiopteridaceae	Pteridophyta	
Angiopteris petiolulata	Angiopteridaceae	Pteridophyta	
Ceterach officinarum	Aspleniaceae	Pteridophyta	NM
Botrychium officinale	Botrychiaceae	Pteridophyta	
Botrychium ternatum	Botrychiaceae	Pteridophyta	AM
Botrychium virginianum	Botrychiaceae	Pteridophyta	AM
Davallia mariesii	Davalliaceae	Pteridophyta	AM
Humata tyermanni	Davalliaceae	Pteridophyta	
Drynaria delavayi	Drynariaceae	Pteridophyta	

Drynaria propinqua	Drynariaceae	Pteridophyta	AM+NM
Drynaria roosii	Drynariaceae	Pteridophyta	
Dryopteris crassirhizoma	Dryopteridaceae	Pteridophyta	
Dryopteris lacera	Dryopteridaceae	Pteridophyta	AM
Vandenboschia naseana	Hymenophyllaceae	Pteridophyta	
Stenoloma chusanum	Lindsaeaceae	Pteridophyta	
Lygodium japonicum	Lygodiaceae	Pteridophyta	AM+NM
Marsilea quadrifolia	Marsileaceae	Pteridophyta	AM+NM
Nephrolepis auriculata	Nephrolepidaceae	Pteridophyta	AM
Ophioglossum thermale	Ophioglossaceae	Pteridophyta	AM
Ophioglossum vulgatum	Ophioglossaceae	Pteridophyta	AM
Ceratopteris pteridoides	Parkeriaceae	Pteridophyta	
Ceratopteris thalictroides	Parkeriaceae	Pteridophyta	AM+NM
Lepidogrammitis diversa	Polypodiaceae	Pteridophyta	
Lepidogrammitis drymoglossoides	Polypodiaceae	Pteridophyta	
Microsorum fortunei	Polypodiaceae	Pteridophyta	
Pyrrosia calvata	Polypodiaceae	Pteridophyta	
Pyrrosia lingua	Polypodiaceae	Pteridophyta	NM
Pyrrosia petiolosa	Polypodiaceae	Pteridophyta	NM
Pteris ensiformis	Pteridaceae	Pteridophyta	AM+NM
Pteris ensiformis var. merrilli	Pteridaceae	Pteridophyta	AM+NM
Pteris multifida	Pteridaceae	Pteridophyta	AM
Pteridium aquilinum	Pteridiaceae	Pteridophyta	AM+NM
Pteridium aquilinum var. latiusculum	Pteridiaceae	Pteridophyta	AM+NM
Salvinia natans	Salviniaceae	Pteridophyta	AM+NM
Schizaea digitata	Schizaeaceae	Pteridophyta	
Onychium japonicum	Sinopteridaceae	Pteridophyta	AM
Onychium japonicum var. lucidum	Sinopteridaceae	Pteridophyta	AM+NM
Pronephrium penangianum	Thelypteridaceae	Pteridophyta	
Pseudocyclosorus falcilobus	Thelypteridaceae	Pteridophyta	
