# Supplementary material for: A Database on Mycorrhizal Traits of Chinese Medicinal Plants
Source: Front Plant Sci. 2022 Mar 1;13:840343. doi: 10.3389/fpls.2022.840343 (PMC8921535; doi:10.3389/fpls.2022.840343)
Supplement: Supplementary file 3 [file Data_Sheet_3.PDF]

OM:obligately mycorrhizal;FM:facultatively mycorrhizal;NM:Non-mycorrhizal

Species	Family	Phylum	Mycorrhizal status
Adhatoda vasica	Acanthaceae	Angiospermae	OM
Barleria cristata	Acanthaceae	Angiospermae	OM
Barleria lupulina	Acanthaceae	Angiospermae	OM
Hygrophila salicifolia	Acanthaceae	Angiospermae	OM
Rhinacanthus nasutus	Acanthaceae	Angiospermae	OM
Mollugo cerviana	Aizoaceae	Angiospermae	OM
Alangium platanifolium	Alangiaceae	Angiospermae	OM
Alangium salviifolium	Alangiaceae	Angiospermae	OM
Alisma canaliculatum	Alismataceae	Angiospermae	OM
Aerva sanguinolenta	Amaranthaceae	Angiospermae	OM
Amaranthus lividus	Amaranthaceae	Angiospermae	OM
Celosia cristata	Amaranthaceae	Angiospermae	OM
Cyathula prostrata	Amaranthaceae	Angiospermae	OM
Agave sisalana	Amaryllidaceae	Angiospermae	OM
Crinum asiaticum	Amaryllidaceae	Angiospermae	OM
Crinum asiaticum var. sinicum	Amaryllidaceae	Angiospermae	OM
Narcissus tazetta var. chinensis	Amaryllidaceae	Angiospermae	OM
Choerospondias axillaris var. axillaris	Anacardiaceae	Angiospermae	OM
Pistacia vera	Anacardiaceae	Angiospermae	OM
Pistacia weinmannifolia	Anacardiaceae	Angiospermae	OM
Rhus chinensis	Anacardiaceae	Angiospermae	OM
Toxicodendron succedaneum	Anacardiaceae	Angiospermae	OM
Annona squamosa	Annonaceae	Angiospermae	OM
Desmos chinensis	Annonaceae	Angiospermae	OM
Alstonia scholaris	Apocynaceae	Angiospermae	OM
Apocynum venetum	Apocynaceae	Angiospermae	OM
Apocynum venetum var. ellipticifolium	Apocynaceae	Angiospermae	OM
Cerbera manghas	Apocynaceae	Angiospermae	OM
Ecdysanthera rosea	Apocynaceae	Angiospermae	OM
Melodinus fusiformis	Apocynaceae	Angiospermae	OM
Rauvolfia tetraphylla	Apocynaceae	Angiospermae	OM

Rauvolfia verticillata	Apocynaceae	Angiospermae	OM
Rauvolfia verticillata var. hainanensis	Apocynaceae	Angiospermae	OM
Rauvolfia vomitoria	Apocynaceae	Angiospermae	OM
Rauvolfia yunnanensis	Apocynaceae	Angiospermae	OM
Trachelospermum jasminoides	Apocynaceae	Angiospermae	OM
Trachelospermum jasminoides var. heterophyllum	Apocynaceae	Angiospermae	OM
Wrightia pubescens	Apocynaceae	Angiospermae	OM
Ilex asprella	Aquifoliaceae	Angiospermae	OM
Ilex cornuta	Aquifoliaceae	Angiospermae	OM
Ilex latifolia	Aquifoliaceae	Angiospermae	OM
Acorus tatarinowii	Araceae	Angiospermae	OM
Aglaonema modestum	Araceae	Angiospermae	OM
Amorphophallus rivieri	Araceae	Angiospermae	OM
Arisaema erubescens	Araceae	Angiospermae	OM
Arisaema flavum	Araceae	Angiospermae	OM
Caladium bicolor	Araceae	Angiospermae	OM
Pinellia ternata	Araceae	Angiospermae	OM
Rhaphidophora decursiva	Araceae	Angiospermae	OM
Rhaphidophora hongkongensis	Araceae	Angiospermae	OM
Schismatoglottis calyptrata	Araceae	Angiospermae	OM
Acanthopanax gracilistylus	Araliaceae	Angiospermae	OM
Acanthopanax senticosus	Araliaceae	Angiospermae	OM
Acanthopanax sessiliflorus	Araliaceae	Angiospermae	OM
Aralia cordata	Araliaceae	Angiospermae	OM
Hedera nepalensis var. sinensis	Araliaceae	Angiospermae	OM
Panax ginseng	Araliaceae	Angiospermae	OM
Tetrapanax papyrifer	Araliaceae	Angiospermae	OM
Aristolochia debilis	Aristolochiaceae	Angiospermae	OM
Aristolochia mollissima	Aristolochiaceae	Angiospermae	OM
Asarum caulescens	Aristolochiaceae	Angiospermae	OM
Asarum sieboldii	Aristolochiaceae	Angiospermae	OM
Asarum sieboldii f. seoulense	Aristolochiaceae	Angiospermae	OM
Calotropis gigantea	Asclepiadaceae	Angiospermae	OM

Cryptolepis buchananii	Asclepiadaceae	Angiospermae	OM
Cynanchum auriculatum	Asclepiadaceae	Angiospermae	OM
Cynanchum sibiricum	Asclepiadaceae	Angiospermae	OM
Gomphocarpus fruticosus	Asclepiadaceae	Angiospermae	OM
Hoya carnosa	Asclepiadaceae	Angiospermae	OM
Hoya carnosa var. marmorata	Asclepiadaceae	Angiospermae	OM
Marsdenia tomentosa	Asclepiadaceae	Angiospermae	OM
Metaplexis japonica	Asclepiadaceae	Angiospermae	OM
Periploca sepium	Asclepiadaceae	Angiospermae	OM
Tylophora arenicola	Asclepiadaceae	Angiospermae	OM
Impatiens chinensis	Balsaminaceae	Angiospermae	OM
Begonia maculata	Begoniaceae	Angiospermae	OM
Berberis poiretii	Berberidaceae	Angiospermae	OM
Berberis sargentiana	Berberidaceae	Angiospermae	OM
Berberis sibirica	Berberidaceae	Angiospermae	OM
Berberis thunbergii	Berberidaceae	Angiospermae	OM
Dysosma versipellis	Berberidaceae	Angiospermae	OM
Sinopodophyllum hexandrum	Berberidaceae	Angiospermae	OM
Campsis grandiflora	Bignoniaceae	Angiospermae	OM
Catalpa bungei	Bignoniaceae	Angiospermae	OM
Incarvillea arguta	Bignoniaceae	Angiospermae	OM
Incarvillea younghusbandii	Bignoniaceae	Angiospermae	OM
Millingtonia hortensis	Bignoniaceae	Angiospermae	OM
Oroxylum indicum	Bignoniaceae	Angiospermae	OM
Radermachera sinica	Bignoniaceae	Angiospermae	OM
Bixa orellana	Bixaceae	Angiospermae	OM
Bombax malabaricum	Bombacaceae	Angiospermae	OM
Cynoglossum officinale	Boraginaceae	Angiospermae	OM
Cynoglossum zeylanicum	Boraginaceae	Angiospermae	OM
Heliotropium indicum	Boraginaceae	Angiospermae	OM
Trigonotis peduncularis	Boraginaceae	Angiospermae	OM
Canarium album	Burseraceae	Angiospermae	OM
Canarium pimela	Burseraceae	Angiospermae	OM

Opuntia monacantha	Cactaceae	Angiospermae	OM
Opuntia stricta var. dillenii	Cactaceae	Angiospermae	OM
Chimonanthus nitens	Calycanthaceae	Angiospermae	OM
Chimonanthus praecox	Calycanthaceae	Angiospermae	OM
Platycodon grandiflorus	Campanulaceae	Angiospermae	OM
Canna indica	Cannaceae	Angiospermae	OM
Lonicera confusa	Caprifoliaceae	Angiospermae	OM
Lonicera hispida	Caprifoliaceae	Angiospermae	OM
Sambucus adnata	Caprifoliaceae	Angiospermae	OM
Carica papaya	Caricaceae	Angiospermae	OM
Silene conoidea	Caryophyllaceae	Angiospermae	OM
Silene fortunei	Caryophyllaceae	Angiospermae	OM
Silene viscidula	Caryophyllaceae	Angiospermae	OM
Casuarina equisetifolia	Casuarinaceae	Angiospermae	OM
Celastrus orbiculatus	Celastraceae	Angiospermae	OM
Euonymus laxiflorus	Celastraceae	Angiospermae	OM
Atriplex centralasiatica	Chenopodiaceae	Angiospermae	OM
Kochia scoparia	Chenopodiaceae	Angiospermae	OM
Chloranthus japonicus	ChloranthaceaeOrchidacea	Angiospermae	OM
Chloranthus multistachys	ChloranthaceaeOrchidacea	Angiospermae	OM
Chloranthus serratus	ChloranthaceaeOrchidacea	Angiospermae	OM
Chloranthus spicatus	ChloranthaceaeOrchidacea	Angiospermae	OM
Terminalia bellirica	Combretaceae	Angiospermae	OM
Terminalia catappa	Combretaceae	Angiospermae	OM
Terminalia chebula	Combretaceae	Angiospermae	OM
Ageratum houstonianum	Compositae	Angiospermae	OM
Anaphalis lactea	Compositae	Angiospermae	OM
Antennaria dioica	Compositae	Angiospermae	OM
Artemisia anethifolia	Compositae	Angiospermae	OM
Artemisia annua	Compositae	Angiospermae	OM
Artemisia argyi	Compositae	Angiospermae	OM
Artemisia argyi var. gracilis	Compositae	Angiospermae	OM
Artemisia brachyloba	Compositae	Angiospermae	OM

Artemisia carvifolia	Compositae	Angiospermae	OM
Artemisia dracunculus	Compositae	Angiospermae	OM
Artemisia dracunculus var. turkestanica	Compositae	Angiospermae	OM
Artemisia dubia	Compositae	Angiospermae	OM
Artemisia dubia var. subdigitata	Compositae	Angiospermae	OM
Artemisia eriopoda	Compositae	Angiospermae	OM
Artemisia giraldii	Compositae	Angiospermae	OM
Artemisia japonica	Compositae	Angiospermae	OM
Artemisia japonica var. hainanensis	Compositae	Angiospermae	OM
Artemisia lavandulaefolia	Compositae	Angiospermae	OM
Artemisia leucophylla	Compositae	Angiospermae	OM
Artemisia littoricola	Compositae	Angiospermae	OM
Artemisia mongolica	Compositae	Angiospermae	OM
Artemisia princeps	Compositae	Angiospermae	OM
Artemisia roxburghiana	Compositae	Angiospermae	OM
Artemisia rubripes	Compositae	Angiospermae	OM
Artemisia rupestris	Compositae	Angiospermae	OM
Artemisia sacrorum	Compositae	Angiospermae	OM
Artemisia sieversiana	Compositae	Angiospermae	OM
Artemisia verlotorum	Compositae	Angiospermae	OM
Artemisia vestita	Compositae	Angiospermae	OM
Artemisia wellbyi	Compositae	Angiospermae	OM
Aster ageratoides	Compositae	Angiospermae	OM
Aster ageratoides var. lasiocladus	Compositae	Angiospermae	OM
Atractylodes lancea	Compositae	Angiospermae	OM
Atractylodes macrocephala	Compositae	Angiospermae	OM
Bidens bipinnata	Compositae	Angiospermae	OM
Bidens frondosa	Compositae	Angiospermae	OM
Bidens parviflora	Compositae	Angiospermae	OM
Blumea balsamifera	Compositae	Angiospermae	OM
Carpesium abrotanoides	Compositae	Angiospermae	OM
Carpesium divaricatum	Compositae	Angiospermae	OM
Carpesium lipskyi	Compositae	Angiospermae	OM

Carthamus tinctorius	Compositae	Angiospermae	OM
Centipeda minima	Compositae	Angiospermae	OM
Conyza blinii	Compositae	Angiospermae	OM
Conyza canadensis	Compositae	Angiospermae	OM
Conyza japonica	Compositae	Angiospermae	OM
Crassocephalum crepidioides	Compositae	Angiospermae	OM
Dahlia pinnata	Compositae	Angiospermae	OM
Dendranthema indicum	Compositae	Angiospermae	OM
Doellingeria scaber	Compositae	Angiospermae	OM
Eclipta prostrata	Compositae	Angiospermae	OM
Elephantopus scaber	Compositae	Angiospermae	OM
Erigeron annuus	Compositae	Angiospermae	OM
Erigeron breviscapus	Compositae	Angiospermae	OM
Eupatorium chinense	Compositae	Angiospermae	OM
Eupatorium odoratum	Compositae	Angiospermae	OM
Farfugium japonicum	Compositae	Angiospermae	OM
Galinsoga parviflora	Compositae	Angiospermae	OM
Glossogyne tenuifolia	Compositae	Angiospermae	OM
Gnaphalium affine	Compositae	Angiospermae	OM
Helianthus annuus	Compositae	Angiospermae	OM
Helianthus tuberosus	Compositae	Angiospermae	OM
Helichrysum arenarium	Compositae	Angiospermae	OM
Inula helenium	Compositae	Angiospermae	OM
Inula helianthus-aquatica	Compositae	Angiospermae	OM
Inula japonica	Compositae	Angiospermae	OM
Inula salicina	Compositae	Angiospermae	OM
Ixeris polycephala	Compositae	Angiospermae	OM
Kalimeris indica	Compositae	Angiospermae	OM
Leontopodium japonicum	Compositae	Angiospermae	OM
Leontopodium leontopodioides	Compositae	Angiospermae	OM
Ligularia sibirica	Compositae	Angiospermae	OM
Petasites japonicus	Compositae	Angiospermae	OM
Pulicaria dysenterica	Compositae	Angiospermae	OM

Pyrethrum cinerariifolium	Compositae	Angiospermae	OM
Saussurea costus	Compositae	Angiospermae	OM
Saussurea gossypiphora	Compositae	Angiospermae	OM
Scorzonera muriculata	Compositae	Angiospermae	OM
Seriphidium transiliense	Compositae	Angiospermae	OM
Siegesbeckia orientalis	Compositae	Angiospermae	OM
Solidago virgaurea	Compositae	Angiospermae	OM
Spilanthes callimorpha	Compositae	Angiospermae	OM
Taraxacum mongolicum	Compositae	Angiospermae	OM
Taraxacum platypecidum	Compositae	Angiospermae	OM
Vernonia anthelmintica	Compositae	Angiospermae	OM
Vernonia esculenta	Compositae	Angiospermae	OM
Xanthium sibiricum	Compositae	Angiospermae	OM
Rourea microphylla	Connaraceae	Angiospermae	OM
Calystegia hederacea	Convolvulaceae	Angiospermae	OM
Calystegia sepium	Convolvulaceae	Angiospermae	OM
Cuscuta australis	Convolvulaceae	Angiospermae	OM
Cuscuta campestris	Convolvulaceae	Angiospermae	OM
Dichondra repens	Convolvulaceae	Angiospermae	OM
Evolvulus alsinoides var. decumbens	Convolvulaceae	Angiospermae	OM
Merremia umbellata subsp. orientalis	Convolvulaceae	Angiospermae	OM
Pharbitis nil	Convolvulaceae	Angiospermae	OM
Porana racemosa	Convolvulaceae	Angiospermae	OM
Coriaria nepalensis	Coriariaceae	Angiospermae	OM
Cornus officinalis	Cornaceae	Angiospermae	OM
Hylotelephium erythrostictum	Crassulaceae	Angiospermae	OM
Orostachys fimbriatus	Crassulaceae	Angiospermae	OM
Sedum aizoon	Crassulaceae	Angiospermae	OM
Sedum major	Crassulaceae	Angiospermae	OM
Sedum sarmentosum	Crassulaceae	Angiospermae	OM
Brassica campestris	Cruciferae	Angiospermae	OM
Cardamine griffithii	Cruciferae	Angiospermae	OM
Cardamine macrophylla	Cruciferae	Angiospermae	OM

Cardamine macrophylla var. polyphylla	Cruciferae	Angiospermae	OM
Actinostemma tenerum	Cucurbitaceae	Angiospermae	OM
Benincasa hispida	Cucurbitaceae	Angiospermae	OM
Citrullus lanatus	Cucurbitaceae	Angiospermae	OM
Cucumis sativus	Cucurbitaceae	Angiospermae	OM
Cucurbita moschata	Cucurbitaceae	Angiospermae	OM
Gynostemma pentaphyllum	Cucurbitaceae	Angiospermae	OM
Lagenaria siceraria	Cucurbitaceae	Angiospermae	OM
Lagenaria siceraria var. microcarpa	Cucurbitaceae	Angiospermae	OM
Luffa cylindrica	Cucurbitaceae	Angiospermae	OM
Siraitia grosvenorii	Cucurbitaceae	Angiospermae	OM
Trichosanthes cucumerina	Cucurbitaceae	Angiospermae	OM
Dioscorea alata	Dioscoreaceae	Angiospermae	OM
Dioscorea bulbifera	Dioscoreaceae	Angiospermae	OM
Dioscorea deltoidea	Dioscoreaceae	Angiospermae	OM
Dioscorea japonica	Dioscoreaceae	Angiospermae	OM
Dioscorea nipponica	Dioscoreaceae	Angiospermae	OM
Dioscorea opposita	Dioscoreaceae	Angiospermae	OM
Dioscorea tokoro	Dioscoreaceae	Angiospermae	OM
Dioscorea zingiberensis	Dioscoreaceae	Angiospermae	OM
Dipsacus asperoides	Dipsacaceae	Angiospermae	OM
Dipsacus japonicus	Dipsacaceae	Angiospermae	OM
Dipterocarpus retusus	Dipterocarpaceae	Angiospermae	OM
Dipterocarpus turbinatus	Dipterocarpaceae	Angiospermae	OM
Diospyros cathayensis	Ebenaceae	Angiospermae	OM
Diospyros lotus	Ebenaceae	Angiospermae	OM
Diospyros maritima	Ebenaceae	Angiospermae	OM
Diospyros morrisiana	Ebenaceae	Angiospermae	OM
Elaeagnus glabra	Elaeagnaceae	Angiospermae	OM
Elaeagnus mollis	Elaeagnaceae	Angiospermae	OM
Elaeagnus pungens	Elaeagnaceae	Angiospermae	OM
Elaeagnus umbellata	Elaeagnaceae	Angiospermae	OM
Hippophae thibetana	Elaeagnaceae	Angiospermae	OM

Elaeocarpus prunifolioides	Elaeocarpaceae	Angiospermae	OM
Cassiope fastigiata	Ericaceae	Angiospermae	OM
Gaultheria forrestii	Ericaceae	Angiospermae	OM
Rhododendron decorum	Ericaceae	Angiospermae	OM
Rhododendron molle	Ericaceae	Angiospermae	OM
Rhododendron ovatum	Ericaceae	Angiospermae	OM
Rhododendron simsii	Ericaceae	Angiospermae	OM
Eucommia ulmoides	Eucommiaceae	Angiospermae	OM
Alchornea trewioides	Euphorbiaceae	Angiospermae	OM
Antidesma bunius	Euphorbiaceae	Angiospermae	OM
Antidesma ghaesembilla	Euphorbiaceae	Angiospermae	OM
Bischofia javanica	Euphorbiaceae	Angiospermae	OM
Breynia fruticosa	Euphorbiaceae	Angiospermae	OM
Croton tiglium	Euphorbiaceae	Angiospermae	OM
Euphorbia antiquorum	Euphorbiaceae	Angiospermae	OM
Euphorbia esula	Euphorbiaceae	Angiospermae	OM
Euphorbia hypericifolia	Euphorbiaceae	Angiospermae	OM
Euphorbia jolkinii	Euphorbiaceae	Angiospermae	OM
Euphorbia lathylris	Euphorbiaceae	Angiospermae	OM
Euphorbia milii	Euphorbiaceae	Angiospermae	OM
Euphorbia milii var. tananarivae	Euphorbiaceae	Angiospermae	OM
Euphorbia pekinensis	Euphorbiaceae	Angiospermae	OM
Euphorbia royleana	Euphorbiaceae	Angiospermae	OM
Flueggea suffruticosa	Euphorbiaceae	Angiospermae	OM
Flueggea virosa	Euphorbiaceae	Angiospermae	OM
Glochidion eriocarpum	Euphorbiaceae	Angiospermae	OM
Glochidion puberum	Euphorbiaceae	Angiospermae	OM
Glochidion zeylanicum	Euphorbiaceae	Angiospermae	OM
Jatropha curcas	Euphorbiaceae	Angiospermae	OM
Phyllanthus urinaria	Euphorbiaceae	Angiospermae	OM
Phyllanthus virgatus	Euphorbiaceae	Angiospermae	OM
Sapium sebiferum	Euphorbiaceae	Angiospermae	OM
Flagellaria indica	Flagellariaceae	Angiospermae	OM

Erodium stephanianum	Geraniaceae	Angiospermae	OM
Geranium carolinianum	Geraniaceae	Angiospermae	OM
Geranium nepalense	Geraniaceae	Angiospermae	OM
Geranium wilfordii	Geraniaceae	Angiospermae	OM
Boea hygrometrica	Gesneriaceae	Angiospermae	OM
Conandron ramondioides	Gesneriaceae	Angiospermae	OM
Corallodiscus flabellatus	Gesneriaceae	Angiospermae	OM
Lysionotus pauciflorus	Gesneriaceae	Angiospermae	OM
Lysionotus pauciflorus var. latifolius	Gesneriaceae	Angiospermae	OM
Paraboea dictyoneura	Gesneriaceae	Angiospermae	OM
Bambusa pervariabilis	Gramineae	Angiospermae	OM
Bambusa pervariabilis × textilis	Gramineae	Angiospermae	OM
Bambusa textilis	Gramineae	Angiospermae	OM
Bambusa textilis var. persistens	Gramineae	Angiospermae	OM
Cymbopogon jwarancusa	Gramineae	Angiospermae	OM
Cymbopogon nardus	Gramineae	Angiospermae	OM
Eragrostis ferruginea	Gramineae	Angiospermae	OM
Eragrostis tenella	Gramineae	Angiospermae	OM
Miscanthus floridulus	Gramineae	Angiospermae	OM
Phyllostachys nigra var. henonis	Gramineae	Angiospermae	OM
Saccharum officinarum	Gramineae	Angiospermae	OM
Setaria geniculata	Gramineae	Angiospermae	OM
Setaria italica	Gramineae	Angiospermae	OM
Calophyllum inophyllum	Guttiferae	Angiospermae	OM
Cratoxylum cochinchinense	Guttiferae	Angiospermae	OM
Cratoxylum formosum subsp. pruniflorum	Guttiferae	Angiospermae	OM
Garcinia multiflora	Guttiferae	Angiospermae	OM
Hypericum attenuatum	Guttiferae	Angiospermae	OM
Hypericum japonicum	Guttiferae	Angiospermae	OM
Aesculus chinensis	Hippocastanaceae	Angiospermae	OM
Salacia prinoides	Hippocrateaceae	Angiospermae	OM
Pittosporopsis kerrii	Icacinaceae	Angiospermae	OM
Belamcanda chinensis	Iridaceae	Angiospermae	OM

Crocus sativus	Iridaceae	Angiospermae	OM
Iris lactea var. chinensis	Iridaceae	Angiospermae	OM
Iris tectorum	Iridaceae	Angiospermae	OM
Agastache rugosa	Labiatae	Angiospermae	OM
Ajuga decumbens	Labiatae	Angiospermae	OM
Amethystea caerulea	Labiatae	Angiospermae	OM
Dracocephalum heterophyllum	Labiatae	Angiospermae	OM
Dracocephalum integrifolium	Labiatae	Angiospermae	OM
Elsholtzia blanda	Labiatae	Angiospermae	OM
Elsholtzia ciliata	Labiatae	Angiospermae	OM
Elsholtzia communis	Labiatae	Angiospermae	OM
Elsholtzia cypriani	Labiatae	Angiospermae	OM
Elsholtzia densa	Labiatae	Angiospermae	OM
Elsholtzia rugulosa	Labiatae	Angiospermae	OM
Elsholtzia strobilifera	Labiatae	Angiospermae	OM
Glechoma longituba	Labiatae	Angiospermae	OM
Gomphostemma microdon	Labiatae	Angiospermae	OM
Hyptis suaveolens	Labiatae	Angiospermae	OM
Lamiophlomis rotata	Labiatae	Angiospermae	OM
Lamium barbatum	Labiatae	Angiospermae	OM
Leonurus artemisia var. albiflorus	Labiatae	Angiospermae	OM
Leonurus artemisia var. artemisia	Labiatae	Angiospermae	OM
Marrubium vulgare	Labiatae	Angiospermae	OM
Melissa officinalis	Labiatae	Angiospermae	OM
Mentha haplocalyx	Labiatae	Angiospermae	OM
Mosla dianthera	Labiatae	Angiospermae	OM
Mosla scabra	Labiatae	Angiospermae	OM
Nepeta cataria	Labiatae	Angiospermae	OM
Ocimum sanctum	Labiatae	Angiospermae	OM
Origanum vulgare	Labiatae	Angiospermae	OM
Panzeria alaschanica	Labiatae	Angiospermae	OM
Paraphlomis javanica var. coronata	Labiatae	Angiospermae	OM
Perilla frutescens	Labiatae	Angiospermae	OM

Phlomis umbrosa	Labiatae	Angiospermae	OM
Phlomis umbrosa var. australis	Labiatae	Angiospermae	OM
Pogostemon cablin	Labiatae	Angiospermae	OM
Rabdosia amethystoides	Labiatae	Angiospermae	OM
Rabdosia sculponeata	Labiatae	Angiospermae	OM
Salvia chinensis	Labiatae	Angiospermae	OM
Salvia miltiorrhiza	Labiatae	Angiospermae	OM
Salvia officinalis	Labiatae	Angiospermae	OM
Salvia przewalskii	Labiatae	Angiospermae	OM
Schizonepeta tenuifolia	Labiatae	Angiospermae	OM
Scutellaria barbata	Labiatae	Angiospermae	OM
Scutellaria discolor	Labiatae	Angiospermae	OM
Scutellaria discolor var. hirta	Labiatae	Angiospermae	OM
Scutellaria scordifolia	Labiatae	Angiospermae	OM
Teucrium japonicum	Labiatae	Angiospermae	OM
Teucrium quadrifarium	Labiatae	Angiospermae	OM
Teucrium scordium	Labiatae	Angiospermae	OM
Akebia quinata	Lardizabalaceae	Angiospermae	OM
Akebia trifoliata	Lardizabalaceae	Angiospermae	OM
Akebia trifoliata subsp. australis	Lardizabalaceae	Angiospermae	OM
Cinnamomum austrosinense	Lauraceae	Angiospermae	OM
Cinnamomum burmannii	Lauraceae	Angiospermae	OM
Cinnamomum camphora	Lauraceae	Angiospermae	OM
Cinnamomum camphora var. linaloolifera	Lauraceae	Angiospermae	OM
Cinnamomum cassia	Lauraceae	Angiospermae	OM
Cinnamomum glanduliferum	Lauraceae	Angiospermae	OM
Cinnamomum porrectum	Lauraceae	Angiospermae	OM
Cinnamomum subavenium	Lauraceae	Angiospermae	OM
Cinnamomum tamala	Lauraceae	Angiospermae	OM
Lindera aggregata	Lauraceae	Angiospermae	OM
Lindera aggregata var. playfairii	Lauraceae	Angiospermae	OM
Lindera communis	Lauraceae	Angiospermae	OM
Lindera obtusiloba	Lauraceae	Angiospermae	OM

Lindera reflexa	Lauraceae	Angiospermae	OM
Litsea coreana var. sinensis	Lauraceae	Angiospermae	OM
Litsea cubeba var. cubeba	Lauraceae	Angiospermae	OM
Litsea cubeba var. cubeba f. obtusifolia	Lauraceae	Angiospermae	OM
Litsea glutinosa	Lauraceae	Angiospermae	OM
Litsea monopetala	Lauraceae	Angiospermae	OM
Machilus thunbergii	Lauraceae	Angiospermae	OM
Neocinnamomum delavayi	Lauraceae	Angiospermae	OM
Neolitsea aurata	Lauraceae	Angiospermae	OM
Neolitsea aurata var. chekiangensis	Lauraceae	Angiospermae	OM
Neolitsea cambodiana	Lauraceae	Angiospermae	OM
Sassafras tzumu	Lauraceae	Angiospermae	OM
Barringtonia racemosa	Lecythidaceae	Angiospermae	OM
Abrus precatorius	Leguminosae	Angiospermae	OM
Acacia senegal	Leguminosae	Angiospermae	OM
Aeschynomene indica	Leguminosae	Angiospermae	OM
Afzelia xylocarpa	Leguminosae	Angiospermae	OM
Albizia julibrissin	Leguminosae	Angiospermae	OM
Albizia julibrissin f. rosea	Leguminosae	Angiospermae	OM
Alysicarpus vaginalis	Leguminosae	Angiospermae	OM
Astragalus bhotanensis	Leguminosae	Angiospermae	OM
Astragalus membranaceus var. mongholicus	Leguminosae	Angiospermae	OM
Astragalus scaberrimus	Leguminosae	Angiospermae	OM
Bauhinia aurea	Leguminosae	Angiospermae	OM
Bauhinia purpurea	Leguminosae	Angiospermae	OM
Butea monosperma	Leguminosae	Angiospermae	OM
Caesalpinia decapetala	Leguminosae	Angiospermae	OM
Cajanus scarabaeoides	Leguminosae	Angiospermae	OM
Campylotropis delavayi	Leguminosae	Angiospermae	OM
Campylotropis pinetorum	Leguminosae	Angiospermae	OM
Campylotropis pinetorum subsp. velutina	Leguminosae	Angiospermae	OM
Cassia fistula	Leguminosae	Angiospermae	OM
Cassia occidentalis	Leguminosae	Angiospermae	OM

Cassia sophera	Leguminosae	Angiospermae	OM
Cercis chinensis	Leguminosae	Angiospermae	OM
Christia vespertilionis	Leguminosae	Angiospermae	OM
Crotalaria assamica	Leguminosae	Angiospermae	OM
Crotalaria juncea	Leguminosae	Angiospermae	OM
Crotalaria linifolia	Leguminosae	Angiospermae	OM
Crotalaria medicaginea	Leguminosae	Angiospermae	OM
Crotalaria retusa	Leguminosae	Angiospermae	OM
Crotalaria spectabilis	Leguminosae	Angiospermae	OM
Crotalaria verrucosa	Leguminosae	Angiospermae	OM
Dalbergia hupeana	Leguminosae	Angiospermae	OM
Dalbergia odorifera	Leguminosae	Angiospermae	OM
Dalbergia pinnata	Leguminosae	Angiospermae	OM
Dendrolobium triangulare	Leguminosae	Angiospermae	OM
Desmodium heterocarpon	Leguminosae	Angiospermae	OM
Desmodium microphyllum	Leguminosae	Angiospermae	OM
Desmodium multiflorum	Leguminosae	Angiospermae	OM
Desmodium triflorum	Leguminosae	Angiospermae	OM
Dichrostachys cinerea	Leguminosae	Angiospermae	OM
Flemingia macrophylla	Leguminosae	Angiospermae	OM
Flemingia strobilifera	Leguminosae	Angiospermae	OM
Gleditsia japonica	Leguminosae	Angiospermae	OM
Gleditsia sinensis	Leguminosae	Angiospermae	OM
Glycine max	Leguminosae	Angiospermae	OM
Glycine soja	Leguminosae	Angiospermae	OM
Glycine soja var. albiflora f. angustifolia	Leguminosae	Angiospermae	OM
Glycyrrhiza glabra	Leguminosae	Angiospermae	OM
Glycyrrhiza inflata	Leguminosae	Angiospermae	OM
Glycyrrhiza uralensis	Leguminosae	Angiospermae	OM
Gueldenstaedtia verna subsp. multiflora	Leguminosae	Angiospermae	OM
Gueldenstaedtia verna subsp. multiflora f. multiflora	Leguminosae	Angiospermae	OM
Indigofera amblyantha	Leguminosae	Angiospermae	OM
Indigofera pseudotinctoria	Leguminosae	Angiospermae	OM

Indigofera suffruticosa	Leguminosae	Angiospermae	OM
Indigofera tinctoria	Leguminosae	Angiospermae	OM
Kummerowia striata	Leguminosae	Angiospermae	OM
Lablab purpureus	Leguminosae	Angiospermae	OM
Lespedeza pilosa	Leguminosae	Angiospermae	OM
Mimosa pudica	Leguminosae	Angiospermae	OM
Mucuna sempervirens	Leguminosae	Angiospermae	OM
Oxytropis aciphylla var. aciphylla	Leguminosae	Angiospermae	OM
Oxytropis microphylla	Leguminosae	Angiospermae	OM
Oxytropis racemosa	Leguminosae	Angiospermae	OM
Pachyrhizus erosus	Leguminosae	Angiospermae	OM
Parkinsonia aculeata	Leguminosae	Angiospermae	OM
Pisum sativum	Leguminosae	Angiospermae	OM
Psoralea corylifolia	Leguminosae	Angiospermae	OM
Pterocarpus indicus	Leguminosae	Angiospermae	OM
Pueraria lobata	Leguminosae	Angiospermae	OM
Rhynchosia volubilis	Leguminosae	Angiospermae	OM
Saraca dives	Leguminosae	Angiospermae	OM
Smithia sensitiva	Leguminosae	Angiospermae	OM
Sophora alopecuroides	Leguminosae	Angiospermae	OM
Sophora flavescens	Leguminosae	Angiospermae	OM
Sophora flavescens var. favescens	Leguminosae	Angiospermae	OM
Sophora flavescens var. kronei	Leguminosae	Angiospermae	OM
Sophora japonica	Leguminosae	Angiospermae	OM
Sophora tonkinensis	Leguminosae	Angiospermae	OM
Sphaerophysa salsula	Leguminosae	Angiospermae	OM
Tadehagi triquetrum	Leguminosae	Angiospermae	OM
Thermopsis chinensis	Leguminosae	Angiospermae	OM
Thermopsis lanceolata	Leguminosae	Angiospermae	OM
Trifolium repens	Leguminosae	Angiospermae	OM
Trigonella foenum-graecum	Leguminosae	Angiospermae	OM
Uraria lagopodioides	Leguminosae	Angiospermae	OM
Vicia amoena	Leguminosae	Angiospermae	OM

Vicia faba	Leguminosae	Angiospermae	OM
Vicia hirsuta	Leguminosae	Angiospermae	OM
Vicia sativa	Leguminosae	Angiospermae	OM
Vicia sepium	Leguminosae	Angiospermae	OM
Vicia tetrasperma	Leguminosae	Angiospermae	OM
Vigna radiata	Leguminosae	Angiospermae	OM
Vigna umbellata	Leguminosae	Angiospermae	OM
Vigna vexillata	Leguminosae	Angiospermae	OM
Aletris spicata	Liliaceae	Angiospermae	OM
Allium fistulosum	Liliaceae	Angiospermae	OM
Allium macrostemon	Liliaceae	Angiospermae	OM
Aloe arborescens var. natalensis	Liliaceae	Angiospermae	OM
Anemarrhena asphodeloides	Liliaceae	Angiospermae	OM
Asparagus cochinchinensis	Liliaceae	Angiospermae	OM
Dianella ensifolia	Liliaceae	Angiospermae	OM
Dracaena cochinchinensis	Liliaceae	Angiospermae	OM
Fritillaria cirrhosa	Liliaceae	Angiospermae	OM
Fritillaria karelinii	Liliaceae	Angiospermae	OM
Fritillaria taipaiensis	Liliaceae	Angiospermae	OM
Fritillaria thunbergii	Liliaceae	Angiospermae	OM
Fritillaria thunbergii var. chekiangensis	Liliaceae	Angiospermae	OM
Fritillaria ussuriensis	Liliaceae	Angiospermae	OM
Hemerocallis citrina	Liliaceae	Angiospermae	OM
Hosta plantaginea	Liliaceae	Angiospermae	OM
Hosta ventricosa	Liliaceae	Angiospermae	OM
Iphigenia indica	Liliaceae	Angiospermae	OM
Lilium brownii	Liliaceae	Angiospermae	OM
Lilium brownii var. viridulum	Liliaceae	Angiospermae	OM
Lilium lancifolium	Liliaceae	Angiospermae	OM
Lilium pumilum	Liliaceae	Angiospermae	OM
Lilium speciosum var. gloriosoides	Liliaceae	Angiospermae	OM
Reineckia carnea	Liliaceae	Angiospermae	OM
Rohdea japonica	Liliaceae	Angiospermae	OM

Smilax glabra	Liliaceae	Angiospermae	OM
Reinwardtia indica	Linaceae	Angiospermae	OM
Tirpitzia ovoidea	Linaceae	Angiospermae	OM
Buddleja asiatica	Loganiaceae	Angiospermae	OM
Buddleja lindleyana	Loganiaceae	Angiospermae	OM
Gardneria multiflora	Loganiaceae	Angiospermae	OM
Mitrasacme pygmaea	Loganiaceae	Angiospermae	OM
Strychnos nux-vomica	Loganiaceae	Angiospermae	OM
Taxillus sutchuenensis var. sutchuenensis	Loranthaceae	Angiospermae	OM
Lagerstroemia subcostata	Lythraceae	Angiospermae	OM
Illicium difengpi	Magnoliaceae	Angiospermae	OM
Illicium verum	Magnoliaceae	Angiospermae	OM
Kadsura japonica	Magnoliaceae	Angiospermae	OM
Liriodendron chinense	Magnoliaceae	Angiospermae	OM
Magnolia denudata	Magnoliaceae	Angiospermae	OM
Magnolia grandiflora	Magnoliaceae	Angiospermae	OM
Magnolia liliflora	Magnoliaceae	Angiospermae	OM
Magnolia officinalis	Magnoliaceae	Angiospermae	OM
Magnolia officinalis subsp. biloba	Magnoliaceae	Angiospermae	OM
Magnolia sieboldii	Magnoliaceae	Angiospermae	OM
Mahonia fortunei	Magnoliaceae	Angiospermae	OM
Michelia figo	Magnoliaceae	Angiospermae	OM
Michelia maudiae	Magnoliaceae	Angiospermae	OM
Schisandra sphenanthera	Magnoliaceae	Angiospermae	OM
Abelmoschus moschatus	Malvaceae	Angiospermae	OM
Abutilon theophrasti	Malvaceae	Angiospermae	OM
Althaea officinalis	Malvaceae	Angiospermae	OM
Althaea rosea	Malvaceae	Angiospermae	OM
Hibiscus syriacus	Malvaceae	Angiospermae	OM
Malva sinensis	Malvaceae	Angiospermae	OM
Malva verticillata	Malvaceae	Angiospermae	OM
Malvastrum coromandelianum	Malvaceae	Angiospermae	OM
Sida szechuensis	Malvaceae	Angiospermae	OM

Maranta arundinacea	Marantaceae	Angiospermae	OM
Phrynium capitatum	Marantaceae	Angiospermae	OM
Melastoma candidum	Melastomataceae	Angiospermae	OM
Cipadessa cinerascens	Meliaceae	Angiospermae	OM
Toona sinensis	Meliaceae	Angiospermae	OM
Fibraurea recisa	Menispermaceae	Angiospermae	OM
Sinomenium acutum	Menispermaceae	Angiospermae	OM
Stephania japonica	Menispermaceae	Angiospermae	OM
Stephania tetrandra	Menispermaceae	Angiospermae	OM
Tinospora sinensis	Menispermaceae	Angiospermae	OM
Artocarpus nitidus	Moraceae	Angiospermae	OM
Artocarpus nitidus subsp. lingnanensis	Moraceae	Angiospermae	OM
Cannabis sativa	Moraceae	Angiospermae	OM
Cannabis sativa subsp. indica	Moraceae	Angiospermae	OM
Cudrania cochinchinensis	Moraceae	Angiospermae	OM
Cudrania tricuspidata	Moraceae	Angiospermae	OM
Ficus hirta	Moraceae	Angiospermae	OM
Ficus pumila	Moraceae	Angiospermae	OM
Ficus variolosa	Moraceae	Angiospermae	OM
Humulus scandens	Moraceae	Angiospermae	OM
Musa basjoo	Musaceae	Angiospermae	OM
Myrica rubra	Myricaceae	Angiospermae	OM
Myristica fragrans	Myristicaceae	Angiospermae	OM
Ardisia chinensis	Myrsinaceae	Angiospermae	OM
Ardisia crenata	Myrsinaceae	Angiospermae	OM
Ardisia crispa	Myrsinaceae	Angiospermae	OM
Ardisia crispa var. amplifolia	Myrsinaceae	Angiospermae	OM
Ardisia crispa var. dielsii	Myrsinaceae	Angiospermae	OM
Ardisia japonica	Myrsinaceae	Angiospermae	OM
Ardisia pusilla	Myrsinaceae	Angiospermae	OM
Maesa japonica	Myrsinaceae	Angiospermae	OM
Myrsine africana	Myrsinaceae	Angiospermae	OM
Rhodomyrtus tomentosa	Myrtaceae	Angiospermae	OM

Camptotheca acuminata	Nyssaceae	Angiospermae	OM
Forsythia suspensa	Oleaceae	Angiospermae	OM
Fraxinus americana	Oleaceae	Angiospermae	OM
Fraxinus malacophylla	Oleaceae	Angiospermae	OM
Jasminum laurifolium	Oleaceae	Angiospermae	OM
Ligustrum lucidum	Oleaceae	Angiospermae	OM
Ligustrum sinense	Oleaceae	Angiospermae	OM
Syringa reticulata var. amurensis	Oleaceae	Angiospermae	OM
Ludwigia adscendens	Onagraceae	Angiospermae	OM
Oenothera glazioviana	Onagraceae	Angiospermae	OM
Oenothera rosea	Onagraceae	Angiospermae	OM
Anoectochilus roxburghii	Orchidaceae	Angiospermae	OM
Goodyera repens	Orchidaceae	Angiospermae	OM
Gymnadenia conopsea	Orchidaceae	Angiospermae	OM
Habenaria dentata	Orchidaceae	Angiospermae	OM
Herminium monorchis	Orchidaceae	Angiospermae	OM
Ludisia discolor	Orchidaceae	Angiospermae	OM
Spiranthes sinensis	Orchidaceae	Angiospermae	OM
Oxalis corniculata	Oxalidaceae	Angiospermae	OM
Oxalis corymbosa	Oxalidaceae	Angiospermae	OM
Cocos nucifera	Palmae	Angiospermae	OM
Livistona chinensis	Palmae	Angiospermae	OM
Rhapis excelsa	Palmae	Angiospermae	OM
Trachycarpus fortunei	Palmae	Angiospermae	OM
Corydalis yanhusuo	Papaveraceae	Angiospermae	OM
Dicentra spectabilis	Papaveraceae	Angiospermae	OM
Dicranostigma leptopodum	Papaveraceae	Angiospermae	OM
Hylomecon japonica	Papaveraceae	Angiospermae	OM
Hylomecon japonica var. subincisa	Papaveraceae	Angiospermae	OM
Hypecoum erectum	Papaveraceae	Angiospermae	OM
Macleaya cordata	Papaveraceae	Angiospermae	OM
Passiflora coerulea	Passifloraceae	Angiospermae	OM
Passiflora edulis	Passifloraceae	Angiospermae	OM

Passiflora foetida	Passifloraceae	Angiospermae	OM
Sesamum indicum	Pedaliaceae	Angiospermae	OM
Phryma leptostachya subsp. asiatica	Phrymaceae	Angiospermae	OM
Phytolacca acinosa	Phytolaccaceae	Angiospermae	OM
Peperomia tetraphylla	Piperaceae	Angiospermae	OM
Piper betle	Piperaceae	Angiospermae	OM
Piper flaviflorum	Piperaceae	Angiospermae	OM
Piper longum	Piperaceae	Angiospermae	OM
Piper sarmentosum	Piperaceae	Angiospermae	OM
Limonium gmelinii	Plumbaginaceae	Angiospermae	OM
Plumbago auriculata	Plumbaginaceae	Angiospermae	OM
Plumbago zeylanica	Plumbaginaceae	Angiospermae	OM
Polygala hybrida	Polygalaceae	Angiospermae	OM
Polygala sibirica	Polygalaceae	Angiospermae	OM
Polygala sibirica var. megalopha	Polygalaceae	Angiospermae	OM
Polygala tatarinowii	Polygalaceae	Angiospermae	OM
Polygala tenuifolia	Polygalaceae	Angiospermae	OM
Fagopyrum dibotrys	Polygonaceae	Angiospermae	OM
Fagopyrum tataricum	Polygonaceae	Angiospermae	OM
Fallopia multiflora	Polygonaceae	Angiospermae	OM
Fallopia multiflora var. ciliinerve	Polygonaceae	Angiospermae	OM
Polygonum amplexicaule	Polygonaceae	Angiospermae	OM
Polygonum amplexicaule var. sinense	Polygonaceae	Angiospermae	OM
Polygonum chinense	Polygonaceae	Angiospermae	OM
Polygonum viviparum	Polygonaceae	Angiospermae	OM
Rheum palmatum	Polygonaceae	Angiospermae	OM
Rumex nepalensis	Polygonaceae	Angiospermae	OM
Talinum paniculatum	Portulacaceae	Angiospermae	OM
Lysimachia barystachys	Primulaceae	Angiospermae	OM
Lysimachia christinae	Primulaceae	Angiospermae	OM
Lysimachia clethroides	Primulaceae	Angiospermae	OM
Lysimachia congestiflora	Primulaceae	Angiospermae	OM
Lysimachia fortunei	Primulaceae	Angiospermae	OM

Lysimachia klattiana	Primulaceae	Angiospermae	OM
Lysimachia lobelioides	Primulaceae	Angiospermae	OM
Primula atrodentata	Primulaceae	Angiospermae	OM
Punica granatum	Punicaceae	Angiospermae	OM
Pyrola calliantha var. calliantha	Pyrolaceae	Angiospermae	OM
Aconitum chasmanthum	Ranunculaceae	Angiospermae	OM
Aconitum coreanum	Ranunculaceae	Angiospermae	OM
Aconitum gymnandrum	Ranunculaceae	Angiospermae	OM
Aconitum rotundifolium	Ranunculaceae	Angiospermae	OM
Anemone altaica	Ranunculaceae	Angiospermae	OM
Anemone hupehensis	Ranunculaceae	Angiospermae	OM
Anemone hupehensis var. hupehensis f. alba	Ranunculaceae	Angiospermae	OM
Anemone hupehensis var. japonica	Ranunculaceae	Angiospermae	OM
Anemone tomentosa	Ranunculaceae	Angiospermae	OM
Aquilegia incurvata	Ranunculaceae	Angiospermae	OM
Clematis apiifolia	Ranunculaceae	Angiospermae	OM
Clematis apiifolia var. obtusidentata	Ranunculaceae	Angiospermae	OM
Clematis florida	Ranunculaceae	Angiospermae	OM
Clematis terniflora var. mandshurica	Ranunculaceae	Angiospermae	OM
Coptis chinensis	Ranunculaceae	Angiospermae	OM
Coptis chinensis var. brevisepala	Ranunculaceae	Angiospermae	OM
Coptis deltoidea	Ranunculaceae	Angiospermae	OM
Hepatica nobilis var. asiatica	Ranunculaceae	Angiospermae	OM
Paeonia emodi	Ranunculaceae	Angiospermae	OM
Paeonia lactiflora	Ranunculaceae	Angiospermae	OM
Paeonia suffruticosa	Ranunculaceae	Angiospermae	OM
Paeonia suffruticosa var. papaveracea	Ranunculaceae	Angiospermae	OM
Pulsatilla campanella	Ranunculaceae	Angiospermae	OM
Pulsatilla cernua	Ranunculaceae	Angiospermae	OM
Pulsatilla chinensis	Ranunculaceae	Angiospermae	OM
Ranunculus arvensis	Ranunculaceae	Angiospermae	OM
Ranunculus cantoniensis	Ranunculaceae	Angiospermae	OM
Ranunculus japonicus	Ranunculaceae	Angiospermae	OM

Ranunculus sieboldii	Ranunculaceae	Angiospermae	OM
Semiaquilegia adoxoides	Ranunculaceae	Angiospermae	OM
Thalictrum alpinum var. elatum	Ranunculaceae	Angiospermae	OM
Thalictrum aquilegifolium var. sibiricum	Ranunculaceae	Angiospermae	OM
Thalictrum delavayi	Ranunculaceae	Angiospermae	OM
Thalictrum foetidum	Ranunculaceae	Angiospermae	OM
Thalictrum minus	Ranunculaceae	Angiospermae	OM
Thalictrum minus var. hypoleucum	Ranunculaceae	Angiospermae	OM
Thalictrum przewalskii	Ranunculaceae	Angiospermae	OM
Thalictrum simplex var. brevipes	Ranunculaceae	Angiospermae	OM
Trollius asiaticus	Ranunculaceae	Angiospermae	OM
Berchemia polyphylla	Rhamnaceae	Angiospermae	OM
Berchemia polyphylla var. leioclada	Rhamnaceae	Angiospermae	OM
Rhamnus frangula	Rhamnaceae	Angiospermae	OM
Rhamnus globosa	Rhamnaceae	Angiospermae	OM
Sageretia thea	Rhamnaceae	Angiospermae	OM
Ziziphus mauritiana	Rhamnaceae	Angiospermae	OM
Agrimonia pilosa var. nepalensis	Rosaceae	Angiospermae	OM
Amygdalus communis	Rosaceae	Angiospermae	OM
Amygdalus mongolica	Rosaceae	Angiospermae	OM
Amygdalus pedunculata	Rosaceae	Angiospermae	OM
Amygdalus persica	Rosaceae	Angiospermae	OM
Armeniaca mume	Rosaceae	Angiospermae	OM
Armeniaca sibirica	Rosaceae	Angiospermae	OM
Armeniaca vulgaris	Rosaceae	Angiospermae	OM
Armeniaca vulgaris var. ansu	Rosaceae	Angiospermae	OM
Cerasus humilis	Rosaceae	Angiospermae	OM
Chaenomeles sinensis	Rosaceae	Angiospermae	OM
Chaenomeles speciosa	Rosaceae	Angiospermae	OM
Chamaerhodos erecta	Rosaceae	Angiospermae	OM
Cotoneaster multiflorus	Rosaceae	Angiospermae	OM
Crataegus cuneata	Rosaceae	Angiospermae	OM
Cydonia oblonga	Rosaceae	Angiospermae	OM

Duchesnea chrysantha	Rosaceae	Angiospermae	OM
Duchesnea indica	Rosaceae	Angiospermae	OM
Eriobotrya japonica	Rosaceae	Angiospermae	OM
Geum aleppicum	Rosaceae	Angiospermae	OM
Photinia parvifolia	Rosaceae	Angiospermae	OM
Photinia serrulata var. serrulata	Rosaceae	Angiospermae	OM
Photinia villosa	Rosaceae	Angiospermae	OM
Potentilla anserina	Rosaceae	Angiospermae	OM
Potentilla bifurca	Rosaceae	Angiospermae	OM
Potentilla discolor	Rosaceae	Angiospermae	OM
Potentilla fruticosa	Rosaceae	Angiospermae	OM
Potentilla griffithii var. velutina	Rosaceae	Angiospermae	OM
Potentilla kleiniana	Rosaceae	Angiospermae	OM
Potentilla reptans var. sericophylla	Rosaceae	Angiospermae	OM
Potentilla tanacetifolia	Rosaceae	Angiospermae	OM
Prinsepia utilis	Rosaceae	Angiospermae	OM
Pyrus betulifolia	Rosaceae	Angiospermae	OM
Rhodotypos scandens	Rosaceae	Angiospermae	OM
Rosa banksiae var. normalis	Rosaceae	Angiospermae	OM
Rosa chinensis	Rosaceae	Angiospermae	OM
Rosa laevigata	Rosaceae	Angiospermae	OM
Rosa maximowicziana	Rosaceae	Angiospermae	OM
Rosa roxburghii	Rosaceae	Angiospermae	OM
Rubus corchorifolius	Rosaceae	Angiospermae	OM
Rubus coreanus var. coreanus	Rosaceae	Angiospermae	OM
Rubus ellipticus var. obcordatus	Rosaceae	Angiospermae	OM
Rubus lambertianus	Rosaceae	Angiospermae	OM
Rubus parvifolius	Rosaceae	Angiospermae	OM
Rubus phoenicolasius	Rosaceae	Angiospermae	OM
Rubus pungens	Rosaceae	Angiospermae	OM
Rubus trianthus	Rosaceae	Angiospermae	OM
Sorbus pohuashanensis	Rosaceae	Angiospermae	OM
Spiraea blumei	Rosaceae	Angiospermae	OM

Catunaregam spinosa	Rubiaceae	Angiospermae	OM
Cinchona ledgeriana	*Rubiaceae*	*Angiospermae*	OM
Galium bungei	Rubiaceae	Angiospermae	OM
Hedyotis auricularia	Rubiaceae	Angiospermae	OM
Hymenodictyon orixense	Rubiaceae	Angiospermae	OM
Morinda citrifolia	Rubiaceae	Angiospermae	OM
Mussaenda pubescens	Rubiaceae	Angiospermae	OM
Psychotria calocarpa	Rubiaceae	Angiospermae	OM
Wendlandia uvariifolia	Rubiaceae	Angiospermae	OM
Boenninghausenia albiflora	Rutaceae	Angiospermae	OM
Boenninghausenia sessilicarpa	Rutaceae	Angiospermae	OM
Citrus aurantium	Rutaceae	Angiospermae	OM
Citrus medica var. sarcodactylis	Rutaceae	Angiospermae	OM
Evodia lepta	Rutaceae	Angiospermae	OM
Fortunella hindsii	Rutaceae	Angiospermae	OM
Micromelum integerrimum	Rutaceae	Angiospermae	OM
Ptelea trifoliata	Rutaceae	Angiospermae	OM
Ruta graveolens	Rutaceae	Angiospermae	OM
Zanthoxylum ailanthoides var. ailanthoides	Rutaceae	Angiospermae	OM
Zanthoxylum armatum var. armatum	Rutaceae	Angiospermae	OM
Zanthoxylum echinocarpum var. echinocarpum	Rutaceae	Angiospermae	OM
Sabia japonica	Sabiaceae	Angiospermae	OM
Populus tomentosa	Salicaceae	Angiospermae	OM
Salix matsudana	Salicaceae	Angiospermae	OM
Thesium chinense var. chinense	Santalaceae	Angiospermae	OM
Koelreuteria paniculata	Sapindaceae	Angiospermae	OM
Nephelium chryseum	Sapindaceae	Angiospermae	OM
Sapindus mukorossi	Sapindaceae	Angiospermae	OM
Chrysophyllum lanceolatum var. stellatocarpon	Sapotaceae	Angiospermae	OM
Manilkara hexandra	Sapotaceae	Angiospermae	OM
Manilkara zapota	Sapotaceae	Angiospermae	OM
Planchonella obovata	Sapotaceae	Angiospermae	OM
Houttuynia cordata	Saururaceae	Angiospermae	OM

Hydrangea macrophylla	Saxifragaceae	Angiospermae	OM
Tiarella polyphylla	Saxifragaceae	Angiospermae	OM
Centranthera cochinchinensis var. nepalensis	Scrophulariaceae	Angiospermae	OM
Scrophularia ningpoensis	Scrophulariaceae	Angiospermae	OM
Veronica linariifolia subsp. dilatata	Scrophulariaceae	Angiospermae	OM
Veronica peregrina	Scrophulariaceae	Angiospermae	OM
Veronicastrum axillare	Scrophulariaceae	Angiospermae	OM
Brucea javanica	Simaroubaceae	Angiospermae	OM
Lycium barbarum	Solanaceae	Angiospermae	OM
Lycium chinense	Solanaceae	Angiospermae	OM
Solanum indicum	Solanaceae	Angiospermae	OM
Solanum lyratum	Solanaceae	Angiospermae	OM
Solanum photeinocarpum	*Solanaceae*	*Angiospermae*	OM
Solanum surattense	Solanaceae	Angiospermae	OM
Solanum verbascifolium	Solanaceae	Angiospermae	OM
Sparganium stoloniferum	Sparganiaceae	Angiospermae	OM
Euscaphis japonica	Staphyleaceae	Angiospermae	OM
Croomia japonica	Stemonaceae	Angiospermae	OM
Stemona japonica	Stemonaceae	Angiospermae	OM
Firmiana platanifolia	Sterculiaceae	Angiospermae	OM
Helicteres isora	Sterculiaceae	Angiospermae	OM
Pterospermum heterophyllum	Sterculiaceae	Angiospermae	OM
Symplocos congesta	Symplocaceae	Angiospermae	OM
Symplocos glauca	Symplocaceae	Angiospermae	OM
Symplocos lancifolia	Symplocaceae	Angiospermae	OM
Symplocos laurina	Symplocaceae	Angiospermae	OM
Symplocos paniculata	Symplocaceae	Angiospermae	OM
Symplocos racemosa	Symplocaceae	Angiospermae	OM
Symplocos sumuntia	Symplocaceae	Angiospermae	OM
Tamarix chinensis	Tamaricaceae	Angiospermae	OM
Eurya japonica	Theaceae	Angiospermae	OM
Aquilaria sinensis	Thymelaeaceae	Angiospermae	OM
Stellera chamaejasme	Thymelaeaceae	Angiospermae	OM

Wikstroemia nutans	Thymelaeaceae	Angiospermae	OM
Corchorus aestuans	Tiliaceae	Angiospermae	OM
Microcos paniculata	Tiliaceae	Angiospermae	OM
Ulmus macrocarpa	Ulmaceae	Angiospermae	OM
Zelkova serrata	Ulmaceae	Angiospermae	OM
Anethum graveolens	Umbelliferae	Angiospermae	OM
Angelica dahurica	Umbelliferae	Angiospermae	OM
Angelica sinensis	Umbelliferae	Angiospermae	OM
Bupleurum chinense	Umbelliferae	Angiospermae	OM
Bupleurum scorzonerifolium	Umbelliferae	Angiospermae	OM
Coriandrum sativum	Umbelliferae	Angiospermae	OM
Ferula ferulaeoides	Umbelliferae	Angiospermae	OM
Ferula fukanensis	Umbelliferae	Angiospermae	OM
Ferula lehmannii	Umbelliferae	Angiospermae	OM
Ferula sinkiangensis	Umbelliferae	Angiospermae	OM
Foeniculum vulgare	Umbelliferae	Angiospermae	OM
Glehnia littoralis	Umbelliferae	Angiospermae	OM
Levisticum officinale	Umbelliferae	Angiospermae	OM
Ligusticum chuanxiong	Umbelliferae	Angiospermae	OM
Peucedanum praeruptorum	Umbelliferae	Angiospermae	OM
Saposhnikovia divaricata	Umbelliferae	Angiospermae	OM
Torilis japonica	Umbelliferae	Angiospermae	OM
Boehmeria nivea	Urticaceae	Angiospermae	OM
Boehmeria nivea var. nipononivea	Urticaceae	Angiospermae	OM
Boehmeria nivea var. tenacissima	Urticaceae	Angiospermae	OM
Gonostegia hirta	Urticaceae	Angiospermae	OM
Pilea notata	Urticaceae	Angiospermae	OM
Urtica fissa	Urticaceae	Angiospermae	OM
Patrinia heterophylla	Valerianaceae	Angiospermae	OM
Patrinia scabiosaefolia	Valerianaceae	Angiospermae	OM
Patrinia villosa	Valerianaceae	Angiospermae	OM
Valeriana jatamansi	Valerianaceae	Angiospermae	OM
Callicarpa cathayana	Verbenaceae	Angiospermae	OM

Caryopteris mongholica	Verbenaceae	Angiospermae	OM
Clerodendrum cyrtophyllum	Verbenaceae	Angiospermae	OM
Clerodendrum inerme	Verbenaceae	Angiospermae	OM
Clerodendrum japonicum	Verbenaceae	Angiospermae	OM
Duranta repens	Verbenaceae	Angiospermae	OM
Lantana camara	Verbenaceae	Angiospermae	OM
Phyla nodiflora	Verbenaceae	Angiospermae	OM
Stachytarpheta jamaicensis	Verbenaceae	Angiospermae	OM
Verbena officinalis	Verbenaceae	Angiospermae	OM
Vitex quinata var. puberula	Verbenaceae	Angiospermae	OM
Vitex trifolia	Verbenaceae	Angiospermae	OM
Vitex trifolia var. simplicifolia	Verbenaceae	Angiospermae	OM
Viola biflora	Violaceae	Angiospermae	OM
Viola collina	Violaceae	Angiospermae	OM
Viola diffusa	Violaceae	Angiospermae	OM
Viola diffusa var. brevibarbata	Violaceae	Angiospermae	OM
Viola grypoceras	Violaceae	Angiospermae	OM
Viola mandshurica	Violaceae	Angiospermae	OM
Viola patrinii	Violaceae	Angiospermae	OM
Viola philippica	Violaceae	Angiospermae	OM
Ampelopsis heterophylla var. brevipedunculata	Vitaceae	Angiospermae	OM
Cayratia japonica	Vitaceae	Angiospermae	OM
Parthenocissus tricuspidata	Vitaceae	Angiospermae	OM
Tetrastigma hemsleyanum	Vitaceae	Angiospermae	OM
Tetrastigma planicaule	Vitaceae	Angiospermae	OM
Vitis flexuosa	Vitaceae	Angiospermae	OM
Alpinia conchigera	Zingiberaceae	Angiospermae	OM
Alpinia japonica	Zingiberaceae	Angiospermae	OM
Alpinia nigra	Zingiberaceae	Angiospermae	OM
Alpinia officinarum	Zingiberaceae	Angiospermae	OM
Alpinia zerumbet	Zingiberaceae	Angiospermae	OM
Amomum maximum	Zingiberaceae	Angiospermae	OM
Amomum subulatum	Zingiberaceae	Angiospermae	OM

Amomum villosum	Zingiberaceae	Angiospermae	OM
Amomum villosum var. nanum	Zingiberaceae	Angiospermae	OM
Amomum villosum var. xanthioides	Zingiberaceae	Angiospermae	OM
Costus speciosus	Zingiberaceae	Angiospermae	OM
Costus tonkinensis	Zingiberaceae	Angiospermae	OM
Curcuma aromatica	Zingiberaceae	Angiospermae	OM
Curcuma caesia	Zingiberaceae	Angiospermae	OM
Curcuma elata	Zingiberaceae	Angiospermae	OM
Curcuma longa	Zingiberaceae	Angiospermae	OM
Curcuma zedoaria	Zingiberaceae	Angiospermae	OM
Hedychium coronarium	Zingiberaceae	Angiospermae	OM
Kaempferia rotunda	Zingiberaceae	Angiospermae	OM
Zingiber mioga	Zingiberaceae	Angiospermae	OM
Zingiber officinale	Zingiberaceae	Angiospermae	OM
Zingiber zerumbet	Zingiberaceae	Angiospermae	OM
Peganum nigellastrum	Zygophyllaceae	Angiospermae	OM
Tribulus terrester	Zygophyllaceae	Angiospermae	OM
Agathis dammara	Araucariaceae	Gymnospermae	OM
Cephalotaxus fortunei	Cephalotaxaceae	Gymnospermae	OM
Cephalotaxus hainanensis	Cephalotaxaceae	Gymnospermae	OM
Cephalotaxus sinensis	Cephalotaxaceae	Gymnospermae	OM
Sabina chinensis	Cupressaceae	Gymnospermae	OM
Cycas revoluta	Cycadaceae	Gymnospermae	OM
Ephedra equisetina	Ephedraceae	Gymnospermae	OM
Ephedra intermedia	Ephedraceae	Gymnospermae	OM
Ephedra monosperma	Ephedraceae	Gymnospermae	OM
Ephedra sinica	Ephedraceae	Gymnospermae	OM
Taxus cuspidata	Taxaceae	Gymnospermae	OM
Botrychium ternatum	Botrychiaceae	Pteridophyta	OM
Botrychium virginianum	Botrychiaceae	Pteridophyta	OM
Davallia mariesii	Davalliaceae	Pteridophyta	OM
Dryopteris lacera	Dryopteridaceae	Pteridophyta	OM
Nephrolepis auriculata	Nephrolepidaceae	Pteridophyta	OM

Ophioglossum thermale	Ophioglossaceae	Pteridophyta	OM
Ophioglossum vulgatum	Ophioglossaceae	Pteridophyta	OM
Pteris multifida	Pteridaceae	Pteridophyta	OM
Onychium japonicum	Sinopteridaceae	Pteridophyta	OM
Andrographis paniculata	Acanthaceae	Angiospermae	FM
Actinidia polygama	Actinidiaceae	Angiospermae	FM
Tetragonia tetragonioides	Aizoaceae	Angiospermae	FM
Alangium chinense	Alangiaceae	Angiospermae	FM
Alisma plantago-aquatica	Alismataceae	Angiospermae	FM
Achyranthes aspera	Amaranthaceae	Angiospermae	FM
Achyranthes bidentata	Amaranthaceae	Angiospermae	FM
Alternanthera philoxeroides	Amaranthaceae	Angiospermae	FM
Alternanthera sessilis	Amaranthaceae	Angiospermae	FM
Amaranthus caudatus	Amaranthaceae	Angiospermae	FM
Amaranthus retroflexus	Amaranthaceae	Angiospermae	FM
Amaranthus spinosus	Amaranthaceae	Angiospermae	FM
Amaranthus tricolor	Amaranthaceae	Angiospermae	FM
Amaranthus viridis	Amaranthaceae	Angiospermae	FM
Celosia argentea	Amaranthaceae	Angiospermae	FM
Agave americana	Amaryllidaceae	Angiospermae	FM
Curculigo orchioides	Amaryllidaceae	Angiospermae	FM
Anacardium occidentale	Anacardiaceae	Angiospermae	FM
Mangifera indica	Anacardiaceae	Angiospermae	FM
Artabotrys hexapetalus	Annonaceae	Angiospermae	FM
Catharanthus roseus	Apocynaceae	Angiospermae	FM
Ichnocarpus frutescens	Apocynaceae	Angiospermae	FM
Nerium oleander	Apocynaceae	Angiospermae	FM
Rauvolfia serpentina	Apocynaceae	Angiospermae	FM
Ilex microcca f. pilosa	Aquifoliaceae	Angiospermae	FM
Acorus calamus var. verus	Araceae	Angiospermae	FM
Aglaonema tenuipes	Araceae	Angiospermae	FM
Alocasia macrorrhiza	Araceae	Angiospermae	FM
Colocasia esculenta	Araceae	Angiospermae	FM

Kalopanax septemlobus	Araliaceae	Angiospermae	FM
Asclepias curassavica	Asclepiadaceae	Angiospermae	FM
Calotropis procera	Asclepiadaceae	Angiospermae	FM
Gymnema sylvestre	Asclepiadaceae	Angiospermae	FM
Impatiens balsamina	Balsaminaceae	Angiospermae	FM
Berberis amurensis	Berberidaceae	Angiospermae	FM
Nandina domestica	Berberidaceae	Angiospermae	FM
Lithospermum officinale	Boraginaceae	Angiospermae	FM
Cleome gynandra	Capparaceae	Angiospermae	FM
Cleome viscosa	Capparaceae	Angiospermae	FM
Agrostemma githago	Caryophyllaceae	Angiospermae	FM
Arenaria serpyllifolia	Caryophyllaceae	Angiospermae	FM
Dianthus chinensis	Caryophyllaceae	Angiospermae	FM
Dianthus chinensis var. longisquama	Caryophyllaceae	Angiospermae	FM
Dianthus chinensis var. versicolor	Caryophyllaceae	Angiospermae	FM
Myosoton aquaticum	Caryophyllaceae	Angiospermae	FM
Stellaria media	Caryophyllaceae	Angiospermae	FM
Stellaria uliginosa	Caryophyllaceae	Angiospermae	FM
Euonymus alatus	Celastraceae	Angiospermae	FM
Ceratophyllum demersum	Ceratophyllaceae	Angiospermae	FM
Chenopodium album	Chenopodiaceae	Angiospermae	FM
Chenopodium ambrosioides	Chenopodiaceae	Angiospermae	FM
Sarcandra glabra	ChloranthaceaeOrchidacea	Angiospermae	FM
Commelina bengalensis	Commelinaceae	Angiospermae	FM
Commelina communis	Commelinaceae	Angiospermae	FM
Commelina diffusa	Commelinaceae	Angiospermae	FM
Cyanotis cristata	Commelinaceae	Angiospermae	FM
Ageratum conyzoides	Compositae	Angiospermae	FM
Artemisia absinthium	Compositae	Angiospermae	FM
Artemisia capillaris	Compositae	Angiospermae	FM
Artemisia frigida	Compositae	Angiospermae	FM
Artemisia ordosica	Compositae	Angiospermae	FM
Artemisia sphaerocephala	Compositae	Angiospermae	FM

Artemisia vulgaris	Compositae	Angiospermae	FM
Bidens biternata	Compositae	Angiospermae	FM
Bidens pilosa	Compositae	Angiospermae	FM
Bidens pilosa var. radiata	Compositae	Angiospermae	FM
Bidens tripartita	Compositae	Angiospermae	FM
Emilia sonchifolia	Compositae	Angiospermae	FM
Inula britannica	Compositae	Angiospermae	FM
Matricaria recutita	Compositae	Angiospermae	FM
Pentanema indicum	Compositae	Angiospermae	FM
Pentanema indicum var. hypoleucum	Compositae	Angiospermae	FM
Sonchus oleraceus	Compositae	Angiospermae	FM
Tussilago farfara	Compositae	Angiospermae	FM
Convolvulus arvensis	Convolvulaceae	Angiospermae	FM
Evolvulus alsinoides	Convolvulaceae	Angiospermae	FM
Ipomoea aquatica	Convolvulaceae	Angiospermae	FM
Ipomoea pes-caprae	Convolvulaceae	Angiospermae	FM
Operculina turpethum	Convolvulaceae	Angiospermae	FM
Helwingia japonica	Cornaceae	Angiospermae	FM
Armoracia rusticana	Cruciferae	Angiospermae	FM
Brassica juncea	Cruciferae	Angiospermae	FM
Brassica juncea var. gracilis	Cruciferae	Angiospermae	FM
Capsella bursa-pastoris	Cruciferae	Angiospermae	FM
Cardamine flexuosa	Cruciferae	Angiospermae	FM
Cardamine hirsuta	Cruciferae	Angiospermae	FM
Cardamine impatiens	Cruciferae	Angiospermae	FM
Cochlearia officinalis	Cruciferae	Angiospermae	FM
Isatis tinctoria	Cruciferae	Angiospermae	FM
Lepidium apetalum	Cruciferae	Angiospermae	FM
Lepidium sativum	Cruciferae	Angiospermae	FM
Lepidium virginicum	Cruciferae	Angiospermae	FM
Raphanus sativus	Cruciferae	Angiospermae	FM
Raphanus sativus var. longipinnatus	Cruciferae	Angiospermae	FM
Rorippa indica	Cruciferae	Angiospermae	FM

Sinapis alba	Cruciferae	Angiospermae	FM
Thlaspi arvense	Cruciferae	Angiospermae	FM
Cucumis melo	Cucurbitaceae	Angiospermae	FM
Momordica charantia	Cucurbitaceae	Angiospermae	FM
Cyperus rotundus	Cyperaceae	Angiospermae	FM
Ottelia alismoides	Dioscoreaceae	Angiospermae	FM
Drosera rotundifolia	Droseraceae	Angiospermae	FM
Diospyros kaki	Ebenaceae	Angiospermae	FM
Elaeagnus angustifolia	Elaeagnaceae	Angiospermae	FM
Empetrum nigrum var. japonicum	Empetraceae	Angiospermae	FM
Vaccinium vitis-idaea	Ericaceae	Angiospermae	FM
Euphorbia helioscopia	Euphorbiaceae	Angiospermae	FM
Euphorbia humifusa	Euphorbiaceae	Angiospermae	FM
Euphorbia peplus	Euphorbiaceae	Angiospermae	FM
Euphorbia thymifolia	Euphorbiaceae	Angiospermae	FM
Phyllanthus emblica	Euphorbiaceae	Angiospermae	FM
Phyllanthus niruri	Euphorbiaceae	Angiospermae	FM
Phyllanthus reticulatus	Euphorbiaceae	Angiospermae	FM
Ricinus communis	Euphorbiaceae	Angiospermae	FM
Quercus dentata	Fagaceae	Angiospermae	FM
Quercus mongolica	Fagaceae	Angiospermae	FM
Quercus mongolica var. grosseserrata	Fagaceae	Angiospermae	FM
Apluda mutica	Gramineae	Angiospermae	FM
Cymbopogon citratus	Gramineae	Angiospermae	FM
Eleusine indica	Gramineae	Angiospermae	FM
Eragrostis pilosa	Gramineae	Angiospermae	FM
Hierochloe odorata var. pubescens	Gramineae	Angiospermae	FM
Lophatherum gracile	Gramineae	Angiospermae	FM
Phragmites australis	Gramineae	Angiospermae	FM
Pogonatherum crinitum	Gramineae	Angiospermae	FM
Setaria viridis	Gramineae	Angiospermae	FM
Garcinia xanthochymus	Guttiferae	Angiospermae	FM
Hypericum monogynum	Guttiferae	Angiospermae	FM

Liquidambar formosana	Hamamelidaceae	Angiospermae	FM
Loropetalum chinense	Hamamelidaceae	Angiospermae	FM
Engelhardtia roxburghiana	Juglandaceae	Angiospermae	FM
Platycarya strobilacea	Juglandaceae	Angiospermae	FM
Juncus effusus	Juncaceae	Angiospermae	FM
Glechoma hederacea	Labiatae	Angiospermae	FM
Lamium album	Labiatae	Angiospermae	FM
Mentha spicata	Labiatae	Angiospermae	FM
Ocimum americanum	Labiatae	Angiospermae	FM
Ocimum basilicum	Labiatae	Angiospermae	FM
Ocimum basilicum var. pilosum	Labiatae	Angiospermae	FM
Prunella vulgaris	Labiatae	Angiospermae	FM
Litsea verticillata	Lauraceae	Angiospermae	FM
Persea americana	Lauraceae	Angiospermae	FM
Acacia farnesiana	Leguminosae	Angiospermae	FM
Astragalus adsurgens	Leguminosae	Angiospermae	FM
Bauhinia variegata	Leguminosae	Angiospermae	FM
Cassia mimosoides	Leguminosae	Angiospermae	FM
Coronilla varia	Leguminosae	Angiospermae	FM
Erythrina variegata	Leguminosae	Angiospermae	FM
Maackia amurensis	Leguminosae	Angiospermae	FM
Maackia amurensis var. buergeri	Leguminosae	Angiospermae	FM
Sesbania grandiflora	Leguminosae	Angiospermae	FM
Sesbania sesban	Leguminosae	Angiospermae	FM
Tamarindus indica	Leguminosae	Angiospermae	FM
Zornia gibbosa	Leguminosae	Angiospermae	FM
Spirodela polyrrhiza	Lemnaceae	Angiospermae	FM
Aloe vera var. chinensis	Liliaceae	Angiospermae	FM
Convallaria majalis	Liliaceae	Angiospermae	FM
Ophiopogon japonicus	Liliaceae	Angiospermae	FM
Polygonatum odoratum	Liliaceae	Angiospermae	FM
Linum usitatissimum	Linaceae	Angiospermae	FM
Buddleja davidii	Loganiaceae	Angiospermae	FM

Lagerstroemia indica	Lythraceae	Angiospermae	FM
Lagerstroemia indica f. alba	Lythraceae	Angiospermae	FM
Lagerstroemia speciosa	Lythraceae	Angiospermae	FM
Lawsonia inermis	Lythraceae	Angiospermae	FM
Lythrum salicaria	Lythraceae	Angiospermae	FM
Woodfordia fruticosa	Lythraceae	Angiospermae	FM
Schisandra chinensis	Magnoliaceae	Angiospermae	FM
Abutilon indicum	Malvaceae	Angiospermae	FM
Hibiscus mutabilis	Malvaceae	Angiospermae	FM
Hibiscus mutabilis f. mutablis	Malvaceae	Angiospermae	FM
Sida acuta	Malvaceae	Angiospermae	FM
Urena lobata	Malvaceae	Angiospermae	FM
Melia azedarach	Meliaceae	Angiospermae	FM
Ficus carica	Moraceae	Angiospermae	FM
Morus alba	Moraceae	Angiospermae	FM
Ardisia quinquegona	Myrsinaceae	Angiospermae	FM
Eucalyptus robusta	Myrtaceae	Angiospermae	FM
Boerhavia diffusa	Nyctaginaceae	Angiospermae	FM
Mirabilis jalapa	Nyctaginaceae	Angiospermae	FM
Nelumbo nucifera	Nymphaeaceae	Angiospermae	FM
Fraxinus chinensis	Oleaceae	Angiospermae	FM
Fraxinus pennsylvanica	Oleaceae	Angiospermae	FM
Fraxinus pennsylvanica var. subintegerrima	Oleaceae	Angiospermae	FM
Fraxinus rhynchophylla	Oleaceae	Angiospermae	FM
Jasminum sambac	Oleaceae	Angiospermae	FM
Epilobium angustifolium	Onagraceae	Angiospermae	FM
Epilobium hirsutum	Onagraceae	Angiospermae	FM
Epilobium palustre	Onagraceae	Angiospermae	FM
Ludwigia hyssopifolia	Onagraceae	Angiospermae	FM
Averrhoa carambola	Oxalidaceae	Angiospermae	FM
Biophytum sensitivum	Oxalidaceae	Angiospermae	FM
Oxalis acetosella	Oxalidaceae	Angiospermae	FM
Oxalis acetosella subsp. griffithii	Oxalidaceae	Angiospermae	FM

Areca catechu	Palmae	Angiospermae	FM
Pandanus tectorius	Pandanaceae	Angiospermae	FM
Chelidonium majus	Papaveraceae	Angiospermae	FM
Papaver rhoeas	Papaveraceae	Angiospermae	FM
Papaver somniferum	Papaveraceae	Angiospermae	FM
Philydrum lanuginosum	Philydraceae	Angiospermae	FM
Piper nigrum	Piperaceae	Angiospermae	FM
Fagopyrum esculentum	Polygonaceae	Angiospermae	FM
Polygonum aviculare	Polygonaceae	Angiospermae	FM
Polygonum bistorta	Polygonaceae	Angiospermae	FM
Polygonum capitatum	Polygonaceae	Angiospermae	FM
Pongamia pinnata	Polygonaceae	Angiospermae	FM
Reynoutria japonica	Polygonaceae	Angiospermae	FM
Rumex acetosa	Polygonaceae	Angiospermae	FM
Rumex japonicus	Polygonaceae	Angiospermae	FM
Eichhornia crassipes	Pontederiaceae	Angiospermae	FM
Portulaca oleracea	Portulacaceae	Angiospermae	FM
Portulaca pilosa	Portulacaceae	Angiospermae	FM
Caltha palustris	Ranunculaceae	Angiospermae	FM
Caltha palustris var. sibirica	Ranunculaceae	Angiospermae	FM
Paeonia obovata	Ranunculaceae	Angiospermae	FM
Paeonia obovata var. willmottiae	Ranunculaceae	Angiospermae	FM
Ranunculus lingua	Ranunculaceae	Angiospermae	FM
Ranunculus sceleratus	Ranunculaceae	Angiospermae	FM
Trollius chinensis	Ranunculaceae	Angiospermae	FM
Hovenia acerba	Rhamnaceae	Angiospermae	FM
Rhamnella franguloides	Rhamnaceae	Angiospermae	FM
Rhamnus cathartica	Rhamnaceae	Angiospermae	FM
Rhamnus davurica	Rhamnaceae	Angiospermae	FM
Ziziphus jujuba	Rhamnaceae	Angiospermae	FM
Ziziphus jujuba var. spinosa	Rhamnaceae	Angiospermae	FM
Ceriops tagal	Rhizophoraceae	Angiospermae	FM
Rhizophora mucronata	Rhizophoraceae	Angiospermae	FM

Crataegus pinnatifida	Rosaceae	Angiospermae	FM
Kerria japonica	Rosaceae	Angiospermae	FM
Kerria japonica f. pleniflora	Rosaceae	Angiospermae	FM
Photinia glabra	Rosaceae	Angiospermae	FM
Pyrus ussuriensis	Rosaceae	Angiospermae	FM
Rosa davurica	Rosaceae	Angiospermae	FM
Rosa davurica var. glabra	Rosaceae	Angiospermae	FM
Rosa multiflora var. cathayensis	Rosaceae	Angiospermae	FM
Rosa rugosa	Rosaceae	Angiospermae	FM
Rubus idaeus	Rosaceae	Angiospermae	FM
Rubus peltatus	Rosaceae	Angiospermae	FM
Sanguisorba officinalis	Rosaceae	Angiospermae	FM
Sanguisorba officinalis var. longifolia	Rosaceae	Angiospermae	FM
Sibbaldia procumbens var. aphanopetala	Rosaceae	Angiospermae	FM
Galium aparine	Rubiaceae	Angiospermae	FM
Galium verum	Rubiaceae	Angiospermae	FM
Gardenia jasminoides	Rubiaceae	Angiospermae	FM
Pavetta hongkongensis	Rubiaceae	Angiospermae	FM
Psychotria serpens	Rubiaceae	Angiospermae	FM
Aegle marmelos	Rutaceae	Angiospermae	FM
Orixa japonica	Rutaceae	Angiospermae	FM
Phellodendron amurense	Rutaceae	Angiospermae	FM
Populus × canadensis	Salicaceae	Angiospermae	FM
Populus davidiana	Salicaceae	Angiospermae	FM
Populus nigra	Salicaceae	Angiospermae	FM
Santalum album	Santalaceae	Angiospermae	FM
Cardiospermum halicacabum	Sapindaceae	Angiospermae	FM
Dimocarpus longan	Sapindaceae	Angiospermae	FM
Dimocarpus longan var. obtusus	Sapindaceae	Angiospermae	FM
Bacopa monnieri	Scrophulariaceae	Angiospermae	FM
Linaria vulgaris subsp. sinensis	Scrophulariaceae	Angiospermae	FM
Striga asiatica	Scrophulariaceae	Angiospermae	FM
Ailanthus altissima	Simaroubaceae	Angiospermae	FM

Solanum dulcamara	Solanaceae	Angiospermae	FM
Solanum melongena	Solanaceae	Angiospermae	FM
Solanum melongena var. esculentum	Solanaceae	Angiospermae	FM
Solanum melongena var. serpentinum	Solanaceae	Angiospermae	FM
Solanum nigrum	*Solanaceae*	*Angiospermae*	FM
Typha angustifolia	Typhaceae	Angiospermae	FM
Typha latifolia	Typhaceae	Angiospermae	FM
Typha orientalis	Typhaceae	Angiospermae	FM
Ulmus parvifolia	Ulmaceae	Angiospermae	FM
Ulmus pumila	Ulmaceae	Angiospermae	FM
Anthriscus sylvestris	Umbelliferae	Angiospermae	FM
Centella asiatica	Umbelliferae	Angiospermae	FM
Conium maculatum	Umbelliferae	Angiospermae	FM
Daucus carota	Umbelliferae	Angiospermae	FM
Oenanthe javanica	Umbelliferae	Angiospermae	FM
Pilea pumila	Urticaceae	Angiospermae	FM
Valeriana officinalis	Valerianaceae	Angiospermae	FM
Avicennia marina	Verbenaceae	Angiospermae	FM
Tectona grandis	Verbenaceae	Angiospermae	FM
Vitex negundo	Verbenaceae	Angiospermae	FM
Vitex negundo var. cannabifolia	Verbenaceae	Angiospermae	FM
Vitex negundo var. heterophylla	Verbenaceae	Angiospermae	FM
Alpinia galanga	Zingiberaceae	Angiospermae	FM
Peganum harmala	Zygophyllaceae	Angiospermae	FM
Juniperus communis	Cupressaceae	Gymnospermae	FM
Juniperus rigida	Cupressaceae	Gymnospermae	FM
Platycladus orientalis	Cupressaceae	Gymnospermae	FM
Ginkgo biloba	Ginkgoaceae	Gymnospermae	FM
Pinus koraiensis	Pinaceae	Gymnospermae	FM
Pinus massoniana	Pinaceae	Gymnospermae	FM
Pinus massoniana var. hainanensis	Pinaceae	Gymnospermae	FM
Pinus tabulaeformis	Pinaceae	Gymnospermae	FM
Adiantum capillus-veneris f. dissectum	Adiantaceae	Pteridophyta	FM

Adiantum flabellulatum	Adiantaceae	Pteridophyta	FM
Adiantum pedatum	Adiantaceae	Pteridophyta	FM
Drynaria propinqua	Drynariaceae	Pteridophyta	FM
Lygodium japonicum	Lygodiaceae	Pteridophyta	FM
Marsilea quadrifolia	Marsileaceae	Pteridophyta	FM
Ceratopteris thalictroides	Parkeriaceae	Pteridophyta	FM
Pteris ensiformis	Pteridaceae	Pteridophyta	FM
Pteris ensiformis var. merrilli	Pteridaceae	Pteridophyta	FM
Pteridium aquilinum	Pteridiaceae	Pteridophyta	FM
Pteridium aquilinum var. latiusculum	Pteridiaceae	Pteridophyta	FM
Salvinia natans	Salviniaceae	Pteridophyta	FM
Onychium japonicum var. lucidum	Sinopteridaceae	Pteridophyta	FM
Mollugo stricta	Aizoaceae	Angiospermae	NM
Sagittaria pygmaea	Alismataceae	Angiospermae	NM
Fissistigma glaucescens	Annonaceae	Angiospermae	NM
Acorus gramineus	Araceae	Angiospermae	NM
Colocasia antiquorum	Araceae	Angiospermae	NM
Lasia spinosa	Araceae	Angiospermae	NM
Pistia stratiotes	Araceae	Angiospermae	NM
Basella alba	Basellaceae	Angiospermae	NM
Kigelia africana	Bignoniaceae	Angiospermae	NM
Dianthus superbus	Caryophyllaceae	Angiospermae	NM
Gypsophila paniculata	Caryophyllaceae	Angiospermae	NM
Pseudostellaria heterophylla	Caryophyllaceae	Angiospermae	NM
Sagina japonica	Caryophyllaceae	Angiospermae	NM
Saponaria officinalis	Caryophyllaceae	Angiospermae	NM
Anabasis aphylla	Chenopodiaceae	Angiospermae	NM
Chenopodium hybridum	Chenopodiaceae	Angiospermae	NM
Salsola collina	Chenopodiaceae	Angiospermae	NM
Murdannia nudiflora	Commelinaceae	Angiospermae	NM
Pollia japonica	Commelinaceae	Angiospermae	NM
Artemisia sacrorum var. incana	Compositae	Angiospermae	NM
Artemisia sacrorum var. messerschmidtiana	Compositae	Angiospermae	NM

Conyza bonariensis	Compositae	Angiospermae	NM
Erycibe obtusifolia	Convolvulaceae	Angiospermae	NM
Ipomoea cairica	Convolvulaceae	Angiospermae	NM
Arabis flagellosa	Cruciferae	Angiospermae	NM
Descurainia sophia	Cruciferae	Angiospermae	NM
Draba oreades	Cruciferae	Angiospermae	NM
Eruca sativa	Cruciferae	Angiospermae	NM
Erysimum cheiranthoides	Cruciferae	Angiospermae	NM
Lepidium latifolium	Cruciferae	Angiospermae	NM
Lepidium latifolium var. affine	Cruciferae	Angiospermae	NM
Nasturtium officinale	Cruciferae	Angiospermae	NM
Drosera peltata	Droseraceae	Angiospermae	NM
Drosera peltata var. multisepala	Droseraceae	Angiospermae	NM
Breynia vitis-idaea	Euphorbiaceae	Angiospermae	NM
Euphorbia pulcherrima	Euphorbiaceae	Angiospermae	NM
Halenia corniculata	Gentianaceae	Angiospermae	NM
Cymbopogon flexuosus	Gramineae	Angiospermae	NM
Calophyllum membranaceum	Guttiferae	Angiospermae	NM
Myriophyllum spicatum	Haloragidaceae	Angiospermae	NM
Comanthosphace ningpoensis	Labiatae	Angiospermae	NM
Leucas martinicensis	Labiatae	Angiospermae	NM
Scutellaria baicalensis	Labiatae	Angiospermae	NM
Teucrium viscidum	Labiatae	Angiospermae	NM
Lindera chunii	Lauraceae	Angiospermae	NM
Bauhinia tomentosa	Leguminosae	Angiospermae	NM
Codariocalyx motorius	Leguminosae	Angiospermae	NM
Gleditsia fera	Leguminosae	Angiospermae	NM
Lemna minor	Lemnaceae	Angiospermae	NM
Cordyline fruticosa	Liliaceae	Angiospermae	NM
Polygonatum sibiricum	Liliaceae	Angiospermae	NM
Melastoma affine	Melastomataceae	Angiospermae	NM
Melia toosendan	Meliaceae	Angiospermae	NM
Euryale ferox	Nymphaeaceae	Angiospermae	NM

Jasminum pentaneurum	Oleaceae	Angiospermae	NM
Pandanus furcatus	Pandanaceae	Angiospermae	NM
Corydalis schanginii	Papaveraceae	Angiospermae	NM
Phytolacca americana	Phytolaccaceae	Angiospermae	NM
Piper hancei	Piperaceae	Angiospermae	NM
Polygonum hydropiper	Polygonaceae	Angiospermae	NM
Portulaca grandiflora	Portulacaceae	Angiospermae	NM
Portulaca quadrifida	Portulacaceae	Angiospermae	NM
Potamogeton pectinatus	Potamogetonaceae	Angiospermae	NM
Aconitum soongaricum	Ranunculaceae	Angiospermae	NM
Anemone rivularis	Ranunculaceae	Angiospermae	NM
Anemone rivularis var. flore-minore	Ranunculaceae	Angiospermae	NM
Anemone vitifolia	Ranunculaceae	Angiospermae	NM
Thalictrum petaloideum	Ranunculaceae	Angiospermae	NM
Rhamnus ussuriensis	Rhamnaceae	Angiospermae	NM
Potentilla chinensis	Rosaceae	Angiospermae	NM
Rubus crataegifolius	Rosaceae	Angiospermae	NM
Psychotria prainii	Rubiaceae	Angiospermae	NM
Tarenna mollissima	Rubiaceae	Angiospermae	NM
Koelreuteria bipinnata	Sapindaceae	Angiospermae	NM
Lindernia antipoda	Scrophulariaceae	Angiospermae	NM
Solanum torvum	Solanaceae	Angiospermae	NM
Tacca chantrieri	Taccaceae	Angiospermae	NM
Gironniera subaequalis	Ulmaceae	Angiospermae	NM
Ulmus gaussenii	Ulmaceae	Angiospermae	NM
Cryptotaenia japonica	Umbelliferae	Angiospermae	NM
Hydrocotyle sibthorpioides	Umbelliferae	Angiospermae	NM
Hydrocotyle sibthorpioides var. batrachium	Umbelliferae	Angiospermae	NM
Oreocnide frutescens	Urticaceae	Angiospermae	NM
Viola acuminata	Violaceae	Angiospermae	NM
Viola acuminata var. pilifera	Violaceae	Angiospermae	NM
Alpinia chinensis	Zingiberaceae	Angiospermae	NM
Nitraria sibirica	Zygophyllaceae	Angiospermae	NM

Ceterach officinarum	Aspleniaceae	Pteridophyta	NM
Pyrrosia lingua	Polypodiaceae	Pteridophyta	NM
Pyrrosia petiolosa	Polypodiaceae	Pteridophyta	NM
