# Supplementary material for: A Database on Mycorrhizal Traits of Chinese Medicinal Plants
Source: Front Plant Sci. 2022 Mar 1;13:840343. doi: 10.3389/fpls.2022.840343 (PMC8921535; doi:10.3389/fpls.2022.840343)
Supplement: Supplementary file 4 [file Data_Sheet_4.PDF]

References

Akhmetzhanova, A, A., N. A. Soudzilovskaia, V. G. Onipchenko, W. K. Cornwell, J. Cornelissen. 2012. A rediscovered treasure: mycorrhizal intensity database for 3000 vascular plant species across the former soviet union. Ecology 93:689.

Al-Yahya'ei, M. N., F. Oehl, M. Vallino, E. Lumini, D. Redecker, A. Wiemken, and P. Bonfante. 2011. Unique arbuscular mycorrhizal fungal communities uncovered in date palm plantations and surrounding desert habitats of Southern Arabia. Mycorrhiza 21:195-209.

Andrade, D. J., O. B. Weber, L. A. Crisostomo, M. I. B. Loiola, A. S. Quaresma, R. O. Ferreira, M. C. Pagano, and J. O. Saggin Junior. 2017. Incidence and diversity of arbuscular mycorrhizal fungi and successor herbaceous plants in an agro-system irrigated with produced water. Symbiosis 71:223-232.

Anene, A, and S. Declerck. 2016. Combination of crotalaria spectabilis with rhizophagus irregularis mucl41833 decreases the impact of radopholus similis in banana. Applied Soil Ecology 106:11-17.

BACK, Marina, Martinello, ALTMANN, Taís, SOUZA, Paulo, Vitor, Dutra, and de. 2016. Influência de fungos micorrízicos arbusculares no desenvolvimento vegetativo de porta-enxertos de citros. Pesqui. Agropecu. Trop Pesquisa Agropecuária Tropical 46(4): 407-412.

Baek, Y. H., J. A. Baik, Y. J. Lee, K. N. Yu, K. S. B., J. S. Lee, and M. H. Chiang. 2009. Effect of Mycorrhiza on Plnt Growth and Drought Resistance in Ardisia pusilla. Journal of Bio-Environment Control 18:132-136.

Ban, Y., Z. Xu, H. Zhang, H. Chen, and M. Tang. 2015. Soil chemistry properties, translocation of heavy metals, and mycorrhizal fungi associated with six plant species growing on lead-zinc mine tailings. Annals of Microbiology 65:503-515.

Bao, Y. Y., F. Sun, and W. Yan. 2005. Preliminary Study on Arbuscular Mycorrhizaes and Their Morphological Types of Common Plants in Inner Mongolia Desert Region. Journal of Arid Land Resources and Environment 19:180-184.

Bao, Y, Y., W. Yan, and M. Q. Zhang. 2007. Arbuscular mycorrhizal fungi associated with common plants in grassland of Inner Mongolia. Mycosystema 26:51-58.

Barnes, C. J., C. Maldonado, T. G. Froslev, A. Antonelli, and N. Ronsted. 2016. Unexpectedly High Beta-Diversity of Root-Associated Fungal Communities in the Bolivian Andes. Frontiers in Microbiology 7:1377.

Birhane, E. B. E., T. W. Kuyper, F. J. Sterck, K. Gebrehiwot, and F. Bongers. 2015. Arbuscular mycorrhiza and water and nutrient supply differently impact seedling performance of dry woodland species with different acquisition strategies. Transactions of the Botanical Society of Edinburgh 8:387-399.

Cai, B. P. 2009. Diversity of arbuscular mycorrhizal fungi associated with Prunus mume in China. PhD thesis, Beijing Forestry University, Beijing, China.

Cai, X. B., Y. L. Peng, and J. P. Gai. 2010. Ecological distribution of arbuscular mycorrhizal fungi in alpine grasslands of Tibet Plateau. Chinese Journal of Applied Ecology 21:2635-2644.

Chandra, K., N. Kumar, and G. Chand. 2010. Studies on mycorrhizal inoculation on dry matter yield and root colonization of some medicinal plants grown in stress and forest soils. Journal of Environmental Biology 31:975-979.

Chandran, P., and V. P. Potty. 2010. In vitro Co-culture of VAM Fungi Glomus microcarpum in Ri T-DNA Transformed Hairy Roots of Vigna vexillata. Biotechnology 3:333-338.

Chang, S. S., C. N . Wang, S. Wang, J. H. Zhu, and K. J. Mao. 2016. Effects of inoculating different kinds of AMF on growth of seedlings in Diospyros lotus. Nonwood Forest Research 2:79-85.

Chatterjee, S., and S. Dutta. 2010. A survey on vam association in three different species of cassia and determination of antimicrobial property of these phytoextracts. Journal of Medicinal Plant Research 4:286-292.

Chen, H., and Y. Guo. 1996. Mycorrhizal survey of broadleaf forest in mountainous area of southern Shaanxi. Journal of Northwest Forestry University 11:99-103.

Chen, S. M., C. Y. Liu, X. X. Zhang, E. T. Cai, and Q. S. Wu. 2018. Mycorrhizal Development Status of Four Turfgrass Plants and Their Main Soil Influence Factors. Journal of Henan Agricultural Sciences 47:63-66.

Chen, T., Z. B. Nan, K. Paul, T. Y. Duan, S. Hui, J. F. Wang, C. H. Li, and F. J. Hou. 2018. Effects of interspecific competition on plant-soil feedbacks generated by long-term grazing. Soil Biology & Biochemistry 126:133-134.

Chen, W.Y., Z. T. Mai, K. L. Cai, Y. L. Chen, and W. J. Hong. 2017. Study on promotion effects of different Arbuscular mycorrhizas fungi on rare tree species. Guangdong Agricultural Sciences 44:13-19.

Chen, X., J. J. Tang, Z. G. Fang, and K. Shimizu. 2004. Effects of weed communities with various species numbers on soil features in a subtropical orchard ecosystem. Agriculture Ecosystems & Environment 102:377-388.

Chen, Y., L. Wang, F. Ma, X. F. Jiang, and J. Dong. 2014. Role of Arbuscular Mycorrhizal Fungi on Iris. Journal of Agricultural Resources and Environment 3:265-272.

Chen, Y. L., J. Luo, K. L. Cai, W. Y. Chen, Z. T. Mai, and W. J. Hong. 2018. Effects of Inoculation with Arbuscular Mycorrhizal Fungi on the Ab-sorption of Nutrients of Aquilaria sinensis and Dalbergia odorifera. Chinese Journal of Tropical Crops 39:2355-2362.

Chen, Y.Y., G. S. Zhu, T. F. Mao, and Z. Y. Liu. 2008. A Preliminary Study on Habenaria dentate Endophytic Fungi. Guizhou Agricultural Sciences 36:12-13+1.

de Oliveira, P. T. F., E. L. Dos Santos, W. A. V. da Silva, M. R. A. Ferreira, L. A. L. Soares, F. A. da Silva, and F. S. B. da Silva. 2019. Production of biomolecules of interest to the anxiolytic herbal medicine industry in yellow passionfruit leaves (Passiflora edulis f. flavicarpa) promoted by mycorrhizal inoculation. Journal of the ence of Food and Agriculture 99:3716-3720.

Deniau, M., V. Jung, C. Le Lann, H. Kellner, B. Béchade, T. Morra, A. Prinzing, and A. Bennett. 2017. Janzen–Connell patterns can be induced by fungal-driven decomposition and offset by ectomycorrhizal fungi accumulated under a

closely related canopy. Functional Ecology 32:785-798.
Dong, L., and C. X. Guo. 2011. Advances in the Study of Mycorrhiza Fungi Diversity of Ornamental Plants. Journal of Qingdao Agricultural University(Natural Science) 28:174-180.
Dong, S., Z. Tian, P. J. Chen, S. K. Rajendran, S. C. Hui, D. Cai, O. Ralf, and Y. K. Wun. 2013. The maturation zone is an important target of piriformospora indica in chinese cabbage roots. Journal of Experimental Botany 14:4529-4540.
Druille, M., M. N. Cabello, P. A. García Parisi, and M. Omacini. 2015. Glyphosate vulnerability explains changes in root-symbionts propagules viability in pampean grasslands. Agriculture Ecosystems & Environment 202:48-55.
Feng, X. X. 2011. Tempo-spatial dynamics in arbuscular mycorrhizal microecology of sophora davidii on the loess plateau. Master's thesis, Northwest A&F University, Yangling Shaanxi, China.
Fujiyoshi, M., A. Kagawa, T. Nakatsubo, and T. Masuzawa. 2006. Effects of arbuscular mycorrhizal fungi and soil developmental stages on herbaceous plants growing in the early stage of primary succession on mount Fuji. Ecological Research 21:278-284.
Gai, S. S. 2017. Linkage Between Shurb Communities and Arbuscular Mycorrhizal Fungi Communities In Karst Peak-Cluster Depression Area of Southwest China. Master's thesis, Guangxi Normal University, Guilin, Guangxi, China.
Gao, A.X., and X. L. He. 2007. Ecological study on AM fungi around roots of medicinal plants in the middle area of Hebei Province. Agricultural Research in the Arid Areas 25:196-201.
Ghanbari, J., G. Khajoei-Nejad, S. V. Van Ruth, and S. Aghighi. 2019. The possibility for improvement of flowering, corm properties, bioactive compounds, and antioxidant activity in saffron (crocus sativus l.) by different nutritional regimes. Industrial Crops & Products 135:301-310.
Gong, B., X. Wu, T. Wei, R. Q. Liao, B. W. Su, J. J. Song, G. H. Jiang, and K. X. Zhu. 2017. Isolation,identification and antitumor activity of endophytic fungi in Taxilli herba from Salix babylonica in Guangxi. Guihaia 37:634-641.
Gong, M. Q, F. Z. Wang, and Y. Chen. 1994. Study on VA Mycorrhizal of Rattan Species. Forest Research 4:359-363.
Gong, X. F, Y. Zhu, Y. N. Peng, Z. Y. Wang, H. Yang, Z. X. Guo, and J. P. Zhou. 2017. Dynamics of arbuscular mycorrhizal fungi distributions, soil nutrients and enzyme activities in rhizosphere soil at different growth stages of Angelica sinensis. Microbiology 44:2596-2605.
Guo, J. 2019. Effect of Nitrongen and Phosphorus Fertilization on Root Traits and Nutrient Uptake of Sorbus pohuashanensis and Acanthopanax sessiliflorus. PhD thesis, Northeast Normal University, Changchun, Jilin, China.
Guo, X., and J. Gong. 2014. Differential effects of abiotic factors and host plant traits on diversity and community composition of root-colonizing arbuscular mycorrhizal fungi in a salt-stressed ecosystem. Mycorrhiza 24:79-94.
Halder, M., P. P. Dhar, J. C. Joardar, M. S. Amin, M. Amin, M. H. Kobir, A. K. Dey,

and M. Rahman. 2018. Effects of edaphoclimatic factors on arbuscular mycorrhiza fungi colonization in chittagong bcsir reserve forest, bangladesh. Bangladesh Journal of Botany 47:97-104.

Han, B. Y., K. D. Wang, N. Zhou, and B. Jiang. 2013. Effects of Arbuscular Mycorrhizal Fungi on Terpenoids in Isodon adenantha. Journal of Chinese Medicinal Materials 36:191-195.

Han, Q., J. Huang, D. Long, X. Wang, and J. Liu. 2017. Diversity and community structure of ectomycorrhizal fungi associated with larix chinensis across the alpine treeline ecotone of taibai mountain. Mycorrhiza 27:1-11.

Han, Y, Z. 2012. Selection of Better Rhizobia Strains for Dolichos lablab Infection and their Inoculation Effects. PhD thesis, Southwest University, Chongqing, China.

Harley, J. L., and E. Harley. 1987. A check-list of mycorrhiza in the british flora. New Phytologist 105:1-102.

Hempel, S., L. Gotzenberger, I. Kuhn, S. G. Michalski, M. C. Rillig, M. Zobel, M. Moora. 2013. Mycorrhizas in the Central European flora: relationships with plant life history traits and ecology. Ecology 94:1389-1399.

Hu, J., C. Luo., and T. Wu. 2015. Infection Characteristics and Species Identification of Arbuscular Mycorrhizal Fungi in the Rhizosphere of Rosa roxburghii Tratt. Guizhou Agricultural Sciences 43:82-86.

Huang. W. L., X. J. Fan, Z. Y. Yan, T. T. Ma, and X. L. Meng. 2012. Diversity Study of Arbuscular Mycorrhizal Fungi of Coptis deltoidea. Journal of Chinese Medicinal Materials 35:689-693.

Huang, X. L. 2017. Study on the Diversity of Artificial Cultivation Arbuscular Mycorrhiza of Blumea. Journal of the Qiannan Normal College for Nationaliti 37:4-8.

Huda, S.M.S., M. B. Uddin, M. M. Haque, M. A. U. Mridha, and M. K. Bhuiyan. 2006. Horizontal distribution of ectomycorrhizal infection in Dipterocarpus turbinatus plantations of Bangladesh. Journal of Forestry Research 17:49-51+90.

Ishii, T., I. Matsumoto, Y. H. Shrestha, L. S. Wamocho, and K. Kadoya. 1998. Observation of Vesicular-Arbuscular Mycorrhizal Formation in the Roots of Some Seasonal Weeds Proliferated in Citrus Orchards. Engei Gakkai zasshi 67:556-558.

Jambhulkar, H. P., and K. M. Suresh. 2019. Eco-restoration approach for mine spoil overburden dump through biotechnological route. Environmental monitoring and assessment 191:1-16.

Jiang. P., and M. Y. Wang. 2012. Colonization rate and diversity of AM fungi in the rhizosphere of seven medicinal plants in Xiamen. Acta Ecologica Sinica 32:4043-4051.

Jiang. P. 2012. The Resources and Species diversity Research on AM Fungi occurring in medicinal plants of South Fujian. Master’s thesis, Huaqiao University, Quanzhou, Fujian, China.

Jiang. W., G. Gou, and Y. Ding. 2013. Influences of arbuscular mycorrhizal fungi on

growth and mineral element absorption of chenglu hybrid bamboo seedlings. Pakistan Journal of Botany 45:303-310.

Joshee. N., S. R. Mentreddy, and A. K. Yadav. 2007. Mycorrhizal fungi and growth and development of micropropagated scutellaria integrifolia plants. Industrial Crops & Products 25:169-177.

Lai. Z. Z. 2011. The Preliminary Studies on Endophyte of Lonicra confusa DC. Master's thesis, Guangzhou University of Chinese Medicine, Guangzhou, Guangdong Province, China.

Li. J. 2010. Enhancement of Paxillus involutus and Hydrophilic Polymers on Salt Tolerance of Tree Species. PhD thesis, Beijing Forestry University, Beijing, China.

LI. J. 2014. Effects of clonal integration on microbial processes and community composition in the rhizosphere of Glechoma longituba. Master's thesis, Chengdu University of Technology, Chengdu, Sichuan, China.

Li. J. H., S. Xie, G. T. W. Wilson, A. B. Cobb, S. M. Tang, L. Z. Guo, K. Wang, and B. Deng. 2019. Plant–microbial interactions facilitate grassland species coexistence at the community level. Oikos 129:1-11.

Li, J. S. 2016. Studying on between hyphal infevtion characteristics of AMF and phylogeny of plant. Master's thesis, Lanzhou University, Lanzhou City, Gansu, China.

Li, X. F. 2016. Urban plants Mycorrhizal and its AM fungi diversity in soil-A Case Study of Inner Mongolia University campus. Master's thesis, Inner Mongolia University, Huhhot, Inner Mongolia, China.

Liang, C. C., Y. P. Xiao, and Z. W. Zhao. 2007. Arbuscular Mycorrhiza and Dark Septate Endophytes an Abandoned Lead-Zinc Mine in Huize, Yunnan, China. Chinese Journal of Applied & Environmental Biology 13:811-817.

Liang, Y. M., Y. R. Su, X. Y. He, and X. B. Chen. 2018. Structure Analysis of Arbuscular Mycorrhizal in Roots from Different Shrubs in Karst Regions. Environmental Science 39:5657-5664.

Lin, Q. H., X. P. Zeng, N. Zhang, Z. H. Huang, and W. N. Huang. 2003. The Isolation and Identification of VA Mycorrhizal Fungi of Nonleguminous Tree of Nitrogen Fixation in Fujian. Journal of Fujian College of Forestry 23:270-273.

Liu, L., T. Zhang, F. S. Gilliam, P. Gundersen, W. Zhang, H. Chen, and J. M. Mo. 2013. Interactive effects of nitrogen and phosphorus on soil microbial communities in a tropical forest. Plos One 8:e61188.

Liu, L.Q., and C. Q. Yang. 2011. Distribution and Infection Situations of Mycorrhizal Fungi in 13 Traditional Chinese Medicines from Chongqing. Journal of Anhui Agricultural Sciences 39:10837-10838.

Liu, X. Y. 2020. The mycorrhizal structure types and AMF diverisity of common plants of liliaceaein Inner Mongolia grassland. Master's thesis, Inner Mongolia University, Huhhot, Inner Mongolia, China.

Long, J. 2014. The Studies on the Constituents of the Root of Anemone tomentosa and Secondary Metabolites Bioactivty of Endophytic Fungi. Master's thesis, Lanzhou University of Technology, Lanzhou, Gansu, China.

Lu, X., W. Y. Hu, B. Huang, Y. Li, Y. Q. Zu,NF. D. Zhan, R. X. Qi, and Y. He. 2017. Effects of Arbuscular Mycorrhizal Fungi (AMF) on Cd Absorption and Accumulation in Maize andSonchus asperL. Hill Using Intercropping System. Soils 49:111-117.

Lu, Y. D., H. J. Zhao, X. Q. Tian, K. N. Xue, and X. Y. Wu. 2007. Mycorrhizal Dependency of Ten Seedling Species for Landscaping and the Inoculation Effects. Journal of Tropical and Subtropical Botany 15:237-243.

Lu, Y. Q., S. X. Chang, H. X. Xing, Y. Li, C. J. Xu, and Z. Wang. 2015. Symbiosis Effect Arbuscular Mycorrhizal Fungi on Plants in Coal Mine Areas. Northern Horticulture 5:78-82.

Lumini, E., J. Pan, F. Magurno, C. Huang, and A. Tedeschi. 2020. Native arbuscular mycorrhizal fungi characterization from saline lands in arid oases, northwest china. Journal of Fungi — Open Access Mycology Journal 6:80.

Luo, J., Z. Y. Yan, X. H. Guo, and Y. L. Wang. 2007. Isolation,identification and the antibacterial activity of endophytic fungi in Euphorbia nematocypha Hand.-Mazz. West China Journal of Pharmaceutical Sciences 22:380-385.

Luo, Q. 2016. Study on the Effect of ERM Fungi on Introduction and Heat-Resistance of Rhododendron molle. Master’s thesis, South China Agricultural University, Guangzhou, Guangdong Province, China.

Luo, X. 2015. Diversity of arbuscular mycorrhizal fungi associated with common wild plants in the drawdown zone of Three Gorges Reservoir. Master’s thesis, Southwest University, Chongqing, China.

Ma, L. M. 2015. Survey on the diversities of Arbuscular mycorrhizal(AM) Fungi and its host plants in wetlands of Beijing area. Master’s thesis, Beijing University of Chemical Technology, Beijing, China.

Ma, W.W., B. W. Deng, and W. Q. Chen. 2016. Preliminary screening of endophyte fungi with functional enzyme activity in Corydalis yanhusuo. Journal of Shaanxi Shaanxi University of Technology(Natural Science Edution) 32:65-72.

Mccain, K., G. T. W. Wilson, and J. W. Blair. 2011. Mycorrhizal suppression alters plant productivity and forb establishment in a grass-dominated prairie restoration. Plant Ecology 212:1675-1685.

Meng, X. Y. 2007. Diversity and Infection of Arbuscular Mycorrhizaes Plants in the lower reaches of Tarim River. Master’s thesis, Xinjiang Agricultural University, Urumqi, Xinjiang, China.

Mo, H. Z., and X. Y. Zhuang. 2013. Study on AMF and Some Mycorrhizal Plants of Three Plants Communities in the Limestone Area in Qingyuan, Guangdong. Guangdong Forestry Science and Technology 29:9-15.

Moyer, J.R., M. J. Clapperton, and A. L. Boswall. 2003. Method and time of alfalfa termination affects cereal growth and weed populations. Canadian Journal of Plant Science 83:969-976.

Mu, D. Y., G. Z. Lu, X. S. Sun, N. Wang, and Z. H. Zhao. 2013. Fungal diversity in rhizosphere soil of medicinal plants in Heilongjiang Province. Acta Ecologica Sinica 33:229-237.

Mu. J. 2012. Seasonal variation of arbuscular mycorrhizal fungi in endangered plants Cricaeaster agrestis and Sinopodophyllum hexandrum roots. PhD thesis, Lanzhou University, Lanzhou City, Gansu, China.

Nash, J., R. Laushman, and C. Schadt. 2020. Ectomycorrhizal fungal diversity interacts with soil nutrients to predict plant growth despite weak plant-soil feedbacks. Plant and Soil 453:445-458.

Niu, ZC. 2007. The resource and lead-resistance of arbuscular mycorrhizal fungi at lead and zinc diggings in the Qinglin muntans. Master’s thesis, Northwest A&F University, Yangling Shaanxi, China.

Peng, S. M., B. L. Wang, J. Lu, Z. S. Ding, X. F. Yuan. 2014. Diversity and Structure of Endophyte in Vegetative Organs of Fritillia thunbergii. Journal of Zhejiang Chinese Medical University 38:1091-1097+1102.

Punyalue, A., S. Jamjod, and B. Rerkasem. 2018. Intercropping maize with legumes for sustainable highland maize production. Mountain Research & Development 38:35-44.

Qi, H. Y., Q. P. Zhang, M. Li, Y. Zhang, Q. C. Wan, and Y. Z. Chen. 2014. Effects of arbuscular mycorrhizal preparation on the growth of okra seedlings. Jiangsu Agricultural Sciences 42:138-139.

Qi, Y. L. 2012. Reserch of arbuscular mycorrhizal fungi diversity and distribution character in different forest types in GuLuBan of Daqing Mountain. Master’s thesis, Inner Mongolia University, Huhhot, Inner Mongolia, China.

Qin, M., G. Shi, J. Miranda, Y. Liu, and Q. Zhang. 2019. Revegetation differentially influences microbial trophic groups in a qinghai-tibetan alpine steppe ecosystem. Journal of Basic Microbiology 59:1-12.

Qin, Y. 2014. Research on the effects of inoculation mycorrhizal fungus to cultivate Artemisia and Artemisinin content. Master’s thesis, Northeast Forestry University, Haerbin, Heilongjiang, China.

Radhika, K. P., and B. F. Rodrigues. 2010. Arbuscular mycorrhizal fungal diversity in some commonly occurring medicinal plants of western ghats,goa region. Journal of Forestry Research 1:45-52.

Rashid, A., N. Ayub, T. Ahmad, J. Gul, and A. G. Khan. 2009. Phytoaccumulation prospects of cadmium and zinc by mycorrhizal plant species growing in industrially polluted soils. Environ Geochem Health 31:91-98.

Rudawska, M., R. Wilgan, D. Janowski, M. Iwański, and T. Leski. 2018. Shifts in taxonomical and functional structure of ectomycorrhizal fungal community of Scots pine (Pinus sylvestris L.) underpinned by partner tree ageing. Pedobiologia 71:20-30.

Shafique, H. A., R. Noreen, V. Sultana, J. Ara, and S. Ehteshamul-Haque. 2015. Effect of endophytic pseudomonas aeruginosa and trichoderma harzianum on soil-borne diseases, mycorrhizae and induction of systemic resistance in okra grown in soil amended with Vernonia anthelmintica (L.) seed's powder. Pakistan Journal of Botany 47:2421-2426.

Shan, B. Q., Y. T. Zhang, X. Li, and M. X. Bai. 2017. Symbiotic retationship between Artemisia species and arbuscular mycorrhiza fungi in crude oil contaminated

soil. Environmental Pollution & Control 39:54-59.

Shi, G. X., S. J. Jiang, J. J. Luo, H. K. Zhou, and H. F. Feng. 2017. Relationships between plant phylogeny and arbuscular mycorrhizal fungal colonization in an alpine meadow ecosystem. Acta Ecologica Sinica 37:3628-3635.

Shi, L. 2007. A Dissertation for the Degree of M.Science Ecological and Physiological studies of AM Fungi from Astragalus membranaceus var. mongholicus. Master's thesis, Hebei University, Baoding, Hebei, China.

Shukla, A., D. Vyas, and J. Anuradha. 2013. Soil depth: an overriding factor for distribution of arbuscular mycorrhizal fungi. Journal of Soil Science & Plant Nutrition 13:23-33.

Sidhoum, W., and Z. Fortas. 2019. The beneficial role of indigenous arbuscular mycorrhizal fungi in phytoremediation of wetland plants and tolerance to metal stress. Archives of Environmental Protection 45:103-114.

Song, H. X., and Z. C. Zhong. 2009. Species diversity of amf community colonized in herbages roots in calcareous soil and purple soil. Ying Yong Sheng Tai Xue Bao 20:1857-1862.

Soudzilovskaia, N. A., S. Vaessen, M. Barcelo, J. He, S. Rahimlou, K. Abarenkov, M. C. Brundrett, S. I. F. Gomes, V. Merckx, and L. Tedersoo. 2020. FungalRoot: global online database of plant mycorrhizal associations. New Phytologist 227:955-966.

Štajerová, K., M. Šmilauerová, and P. Šmilauer. 2009. Arbuscular mycorrhizal symbiosis of herbaceous invasive neophytes in the czech republic. Preslia 81:341-355.

Stevens, K. J., M. R. Wellner, and M. F. Acevedo. 2010. Dark septate endophyte and arbuscular mycorrhizal status of vegetation colonizing a bottomland hardwood forest after a 100 year flood. AQUATIC BOTANY 92:105-111.

Straker, C. J., I. M. Weiersbye, and E. T. F. Witkowski. (2007). Arbuscular mycorrhiza status of gold and uranium tailings and surrounding soils of south africa's deep level gold mines: I. root colonization and spore levels. South African Journal of Botany 73:218-225.

Sun, J. Q. 2007. Diversity and Ecological Distribution of Endophytic Fungi of Common Medicinal Plants in North China. PhD thesis, Northeast Forestry University, Haerbin, Heilongjiang, China.

Sun, L., K. Pei., W. Fang, Q. Ding, Y. Bing, G. Bo, Z. Yu, L. Yu, and K. Ma. 2012. Different distribution patterns between putative ercoid mycorrhizal and other fungal assemblages in roots of rhododendron decorum in the southwest of china. Plos One 7:e49867.

Sun, L. 2014. Studies on the population distribution pattern of poisonous plants and its correlation with plateau pika. AM fungi in the Tibetan Plateau alpine medow. Master's thesis, Lanzhou University, Lanzhou City, Gansu, China.

Sun, W. B. 2018. Studies on the Endophytic Fungi from Illicium difengpi and Their Antimicrobial Ingredients. Master's thesis, Guangxi Normal University, Guilin, Guangxi, China.

Sun, Z., S. J. Wang, X. Wang, W. T. Zhou, and H. B. Ma. 2020. Advances in Research

on Relationship Between Casuarina equisetifolia and Root Microorganisms. World Forestry Research 33:25-30.

Talukder, M. R., A. Sarkar, and M. Rashid. 2019. The role of arbuscular mycorrhizal fungi in the bioprotection of ash gourd (benincasa hispida) against damping off disease. Japanese Journal of Applied Entomology and Zoology 4:704-712.

Tan, S. Y., Q. Y. Jiang, Z. Feng, H. Liu, Y. T. Wang, S. S. Li, Z. H. Ye, Y. X. Jing, Y. Jiang, and L. H. Wang. 2015. Effect of inoculation with glomus versiforme on cadmium accumulation, antioxidant activities and phytochelatins of solanum photeinocarpum. Plos One 10:e0132347.

Tan, X. M., L. Y. Yu, Y. Q. Zhou, X. L. Zhou, and Y. Wei. 2013. Microscopic observation on mycorrhiza of rare herb Dysosma versipellis. China Journal of Chinese Materia Medica 38:4044-4046.

Tebuqin, and Y. Y. Bao. 2015. Colonization characteristics of AMF in common Mongolian medicinal plants of Horqin sandy land. Inner Mongolia Agricultural Science And Technology 43:25-28.

Tsetsegmaa, M. 2018. Study on the common plants Mycorrhizal Structure and Rhizosphere AMF Diversity of Different plant communities in central province, Mongolia. PhD thesis, Inner Mongolia University, Huhhot, Inner Mongolia, China.

Tsuruta, J., M. Okumura, N. Makita, Y. Kosugi, T. Miyama, T. Kume, and S. Tohno. 2018. A comparison of the biogenic volatile organic compound emissions from the fine roots of 15 tree species in Japan and Taiwan. Journal of Forest Research 23:242-251.

Valverde-Barrantes, O. J., G. T. Freschet, C. Roumet, and C. B. Blackwood. 2017. A worldview of root traits: the influence of ancestry, growth form, climate and mycorrhizal association on the functional trait variation of fine-root tissues in seed plants. New Phytologist 215:1562-1573.

Wang, AR. 2014. The Research on Isolation and Identification of Chloranthus multistachys Endophytic Fungi and Their Metabolites. Master’s thesis, Southwest University, Chongqing, China.

Wang, B., and Y. L. Qiu. 2006. Phylogenetic distribution and evolution of mycorrhizas in land plants. Mycorrhiza 16:299-363.

Wang, G. J. 2005. The symbiotic diversity of Arbuscular Mycorrhizal in Salinized Leymus chinensis Grassland in western Jilin Province. Master’s thesis, Northeast Normal University, Changchun, Jilin, China.

Wang, J. G. 2011. Study on the AMF Diversity and AM Improved the Drought Resistance of Amygdalus monglica. Inner Mongolia Agricultural University, Master’s thesis, Inner Mongolia University, Huhhot, Inner Mongolia, China.

Wang, M. Y., P. Jiang, and F. J. Liu. 2014. Colonisation and Distribution of AM Fungi in the Rhizosphere of 28 Medicinal Plants in Quanzhou Area. Journal of Huaqiao University(Natural Science 35:299-304.

Wang, M. Y., P. Jiang, and J. F. Liu. 2014. Colonisation and Distribution of AM Fungi in the Rhizosphere of 28 Medicinal Plants in Quanzhou Area. Journal of Huaqiao University(Natural Science) 35:299-304.

Wang, M. Y., Z. K. Xi, and R. J. Liu. 2004. Taxonomic Characteristics and Distribution of Species and Genera in Gigaporaceae of AM Fungi. Journal of Fungal Research 2:6-11.

Wang, Q., X. L. He, and T. S. Chen. 2009. Study on the Annual Change of AM Fungi's Colonization and Spore Density in the Rhizospere of Pueraria lobata. Chinese Agricultural Science Bulletin 25:222-224.

Wang, X. W. 2013. Studies on internation of nitrogen and phosphorus absorption and medicinal plant by arbuscular mycorrhizal fungus. Master's thesis, Zhejiang Normal University, Hangzhou, Zhejiang, China.

Wang, Y., S. Chen, S. J. Qi, X. J. Wu, and L. J. Wang. 2017. Isolation and Identification of Fungi in Roots of Pyrola calliantha. Guizhou Agricultural Sciences 45:112-116.

Wang, Y. J., J. L. Cui, H. Su, and L. Fan. 2009. Inhibitory Activity Screening of Endophytic Fungi from Polygala tenuifolia Willd. Microbiology 36:404-411.

Wang, Y. Y., Z. M. Diao, and K. L. Chen. 2015. Opportunity and Challenge of Qinghai Prataculture Sustainable Development. Qinghai Prataculture 24:35-43.

Wang, Z. 2010. The study of the rhizosphere niche characteristics of the dominant plant species in Karst Mountain. Master's thesis, Nanjing Forestry University, Nanjing, Jiangsu, China.

Wang, Z. G., Y. L. Bi, B. Jiang, Y. Zhakypbek, S. P. Peng, W. W. Liu, and H. Liu. 2016. Arbuscular mycorrhizal fungi enhance soil carbon sequestration in the coalfields, northwest China. Rep 6:34336.

Wu, C. H., J. J. Tang, X. Chen, J. Chen, R. Y. Yang, and Q. Q. Jiang. 2005. Mycorrhizal colonization and lead uptake of weeds in lead-polluted soil. Acta Ecologica Sinica 25:1325-1330.

Wu, J. R., Y. Wang, C. Y. Zhao, Q. H. Lu, Z. Gao, F. Wang, and H. C. Ma. 2014. AMF isolated from Bombacaceae plants in Dry and Hot Valley of Yunnan. Journal of Northwest A&F University (Nat. Sci. Ed.) 42:205-210.

Wu, L. Q. 2005. Study on the Interaction Between the Endophytic Fungi and Saussurea involucrate Kar. et Kir. PhD thesis, Peking Union Medical College, Beijing, China.

Wu, S., Z. Xin., B. Chen, Z. Wu, and Y. Wang. (2015). Chromium immobilization by extraradical mycelium of arbuscular mycorrhiza contributes to plant chromium tolerance. Environmental and Experimental Botany 122:10-18.

Wu, Y. 2013. Colonization and Spore Densities of AMF in Mangroves Located in Different Regions of China. Master's thesis, Sun Yat-sen University, Guangzhou, Guangdong, China.

Wu, Q. S., A. K. Srivastava, and Y. Li. 2015. Effects of mycorrhizal symbiosis on growth behavior and carbohydrate metabolism of trifoliate orange under different substrate p levels. Journal of Plant Growth Regulation 34:499-508.

Xie, H. R. 2017. The Diversity and Biocontrol Effects on Bacterial Will of Endophytic Fungi from patchouli. Master's thesis, Guangdong Pharmaceutical University, Guangzhou, Guangdong Province, China.

Xu, J. 2013. The distribution of arbuscular mycorrhizal fungi and the relationship with soil factore in lead-zine mine area. Master's thesis, Northwest A&F University, Yangling Shaanxi, China.

Xue, J. J., F. Qin, Z. B. Fang, Z. M. Qin, and L. B. Deng. 2005. Study on stock of Siraitia grosvenorii. Journal of Guangxi Agricultural and Biological Science 24:51-53.

Xue, K. N., Y. D. Lu, H. J. Zhao, and R. Z. Luo. 2006. The Field Effect of Vesicular Arbuscular Mycorrhiza on Pterospermum heterophyllum. Guangxi Forestry Science 35:133-135.

Yan, W., and Y. Y. Bao. 2004. Arbuscular mycorrhizae and their structural types on common plants in grasslands of mid-western Inner Mongolia. Chinese Biodiversity 12(5): 501-508.

Yang, D. J., J. Qiu, Y. B. Jing, Y. F. Gen, F. Zhang, J. H. Mao, and K. F. Zhang. 2015. Effects of Arbuscular Mycorrhizal Fungi Inoculation on Seedlings of Pterocarpus indicus. Journal of West China Forestry Science 44:30-35.

Yang, L. X., Y. D. Xie, Z. Q. He, and W. Y. Yan. 2020. Effects of selenium and arbuscular mycorrhizal fungi on the growth and quality of Talinum paniculatum(Jacq.)Gaertn under cadmium stress. Journal of Agro-Environment Science 39(5): 982-988.

Yang, M., J. Zhang, D. Q. Zhang, J. He, J. S. Qi, and N. Zhou. 2018. Effect of Arbuscular Mycorrhizal on Yield and Secondary Metabolites of Rheum palmatum. Chinese Journal of Experimental Traditional Medical Formulae 24:33-37.

Yang, R., S. Li, Z. Qin, X. Cai, X. Li, P. Christie, J. Zhang, G. Feng, J. Gai. 2018. Importance of AM fungi and local adaptation in plant response to environmental change: Field evidence at contrasting elevations. Fungal Ecology 34:59-66.

Yang, R,, S. Zan, J. Tang, C. Xin, and Q. Zhang. 2010. Variation in community structure of arbuscular mycorrhizal fungi associated with a cu tolerant plant—elsholtzia splendens. Applied Soil Ecology 44:191-197.

Yang, W. W., D. Q. Guo, W. G. Cao, X. J. Pan, Y. B. Xue, J. Zhang, and N. Zhou. 2020. Effects of 27 Strains of Arbuscular Mycorrhizal Fungi Inoculation on Physiology and Biochemistry and Major Components of Terpenoids in Potted Saussurea costus. Chinese Journal of Tropical Crops 41:1822-1830.

Yang, Y.H., Y. N. Chen, W. H. Li. 2008. Arbuscular mycorrhizal fungi infection and environmental influencing factors in desert riparian forest: a case study in the lower reaches of Tarim River. Progress in Natural Science 18:397-405.

Yao, Q., H. H. Zhu, D. Wang, and L. Q. Li. 2006. Differential growth response of dominant and subordinate plant species to AM fungi in subtropical grasslands. Acta Ecologica Sinica 26:2288-2293.

Yao, Y. Q. 2017. The diversity, antimicrobial activity and secondary metabolites of endophytic fungi in sophora tonkinensis gagnep. PhD thesis, Guangxi University, Nanning, Guangxi, China.

Yi, X. H., M. Q. Zhu, Z. H. Wang, X. L. Fang, and X. Zhang. 2008. A Preliminary

Study on Population and Distribution of Endophytic Fungi from Pyrethrum cinerariifolium. Journal of Fungal Research 6:78-82.

Yu. Y., L. Zhao., M. Cui, Y. Xiao, and S. Han. 2020. Exploring slope spatial heterogeneity by nitrogen transfer and arbuscular mycorrhizal community. Journal of Soils and Sediments 20:3569-3579.

Yuan, L. H., and W. K. Wang. 2011. Influence of AM Fungion Seedling Growth and Photosythsis of Elaeagnus mollis. Journal of Northwest Forestry University 26:33-35+127.

Yuan, Y., M. V. Kleunen, and J. Li. 2021. A parasite indirectly affects nutrient distribution by common mycorrhizal networks between host and neighboring plants. Ecology 102:e03339.

Yuan, Y., R. Zheng, and L. Chun. 2017. AMF Community Structure in Rhizosphere Soil of Amygdalus pedunculata Pall. of Different Slopes in Daqingshan Mountains. Northern Horticulture 22:102-108.

Yuan, Z. M. 2019. Infect of AMF of main afforestation tree species in coastal areas of Northern Jiangsu and its correlation with edaphic factors. Master’s thesis, Nanjing Forestry University, Nanjing, Jiangsu, China.

Zaman, P., A. K. Roy, N. S. Khanum, N. Absar, and T. Yeasmin. 2008. Arbuscular mycorrhizal status of medicinal plants in Rajshahi University Campus. Mycosystema 27:543-553.

Zhang, F. F., K. Zhou, Y. J. Zhao, F. X. Xie, and L. Li. 2012. Status of VA mycorrhizal fungi resources in saline-alkaline soil of Tianjin. Agricultural Research in the Arid Areas 30:149-153.

Zhang, J. H.,B. B. Feng, and C. S. Wu. 2020. Effects of Arbuscular Mycorrhizal Fungi on Growth and Q-Markers of Fritillaria Taipaiensis. Chinese Journal of Information on Traditional Chinese Medicine 27:88-93.

Zhang, M. C., Y. S. Li, M. Wang, E. Q. Tang, X. Q. Shan. 1998. Observation of vesicular-arbuscular mycorrhizas in asarum heterotropoides fr. Schmidt var.mandshuricum(maxim.) kitag., lonicera edulis turcz. And actinidia arguta planch. Journal of Jilin Agricultural University 10:61-64+95-96.

Zhang, M. F., and Z. X. Li. 1995. Investigation on the Resources of Vesicular-Arbuscular Mycorrhiza. Journal of Guangxi Agricultural University 14:129-136.

Zhang, R. F., P. Q. Li, J. L. Zhao, T. J. Shan, C. H. Yin, and L. G. Zhou. 2010. Endophytic Fungi from Dioscorea zingiberensis and Their Effects on the Growth and Diosgenin Production of the Host Plant Cultures. Natural Product Research and Development 22:11-15.

Zhang, X. T. 2010. Diversity of plant root fungi in xishuangbanna tropical rain forest. Master’s thesis, Yunnan University, Kunming, Yunnan, China.

Zhang, Y., L. D. Guo, and R. J. Liu. 2004. Survey of arbuscular mycorrhizal fungi in deforested and natural forest land in the subtropical region of Dujiangyan, southwest China. Plant and Soil 261:257-263.

Zhang, Y. 2012. Relationship between ectomycorrhizal fungi and subtropical forest succession in Gutianshan. Master’s thesis, Qiqihar University, Qiqihar,

Heilongjiang, China.
Zhao, D. D., C. C. Liang, and Z. W. Zhao. 2006. Arbuscular Mycorrhizas in the Tributary Dry-hot Valleys (Puduhe and Xiaojiang) of Jinsha River. Acta Botanica Yunnanica 28:250-256.
Zhao, H. J., Y. D. Lu, X. P. Zhang, A. H. Yin, and J. D. Tan. 2008. The growth effect of VA mycroohiza fungiI on saraca dives plerre and vatica astrotricha. Journal of Inner Mongolia Agricultural University(Natural Science Edition) 29:80-83.
Zhao, J., and X. L. He. 2010. Resource and distrubition of AM fungi in the rhizosphere of medicinal plants in Anguo city of Hebei province. Journal of Agricultural University of Hebei 33:39-44.
Zhao, J. L., X. Q. Chen, X. Y. Gu, and B. Liu. 2012. Relationship between AM Fungi of New Eight Medicinal Plants and Soil Factors in Anguo of Hebei. Journal of Henan Agricultural Sciences 41:87-91.
Zhao, X., Z. L. Wu, H. Zhang, X. Z. Yang, C. Han, and J. Gao. 2020. Arbuscular mycorrhizal fungi infection rates of flora of thr Fengfeng mining area coal gob piles and influence on plant Cd content. Acta Prataculturae Sinica 29:78-87.
Zhao, Z., K. X. Ma, and A. A. Duan. 1994. Study of negative associations among vaand ectomycorrhizae of populous tomentosa. Scientia Silvae Sinicae 30:111-116.
Zheng, W. J., and M. Y. Wang. 2015. Influence of Uranium in Pteris vittata L. Inoculated by Arbuscular Mycorrhizal Fungus. Environmental Science 8:3004-3010.
Zhou, L. S., and S. X. Guo. 2013. Molecular diversity of arbuscular mycorrhizal fungi in wild and cultured Gynostemma pentaphyllum roots in Xishuangbanna, Southwest China. Chinese Journal of Applied Ecology 24:2503-2510.
Zhou, L. S., X. Zeng, and S. X. Guo. 2019. Characterization of Arbuscular Mycorrhizal Fungus Communities of Aquilaria sinensis Roots from Yunnan Province. Chinese Pharmaceutical Journal 54:867-873.
Zhou, Z. 2013. Arbuscular mycorrhizal and zinc fertilizer promote apricot growth and nutrient absorption. China Fruit News 30:77.
Zhou, Z. Q., Y. N. Hu, Y. L. Peng, M. L. Sun, Y. H. Zhang, and D. Liu. 2015. Effects of three Arbuscular Mycorrhizas on Different Provenances of Amur Cork Seedings. Bulletin of Botanical Research 35:92-100.
Zhu, D. L. 2008. Studies on Biological Characteristics of the Genus Artemisia L. from Xiaowutai Mountains of Hebei. Master’s thesis, Hebei University, Baoding, Hebei, China.
Zhu, J., X. J. Li, L. Sun, S. X. Guo, and J. Chen. 2015. Ecological distribution and diversity of medical Ferula species produced in Xinjiang. China Journal of Chinese Materia Medica 42:356-361.
Zubek, S., J. B. »Aszkowski, and W. Buchwald. 2012. Fungal root endophyte associations of medicinal plants. Nova Hedwigia 94:525-540.
